# Supplementary material for: A unique Pd-catalysed Heck arylation as a remote trigger for cyclopropane selective ring-opening
Source: Nat Commun. 2017 Feb 7;8:14200. doi: 10.1038/ncomms14200 (PMC5309700; doi:10.1038/ncomms14200)
Supplement: Supplementary Information — Supplementary figures, supplementary tables, supplementary note, supplementary methods and supplementary references. [file ncomms14200-s1.pdf]

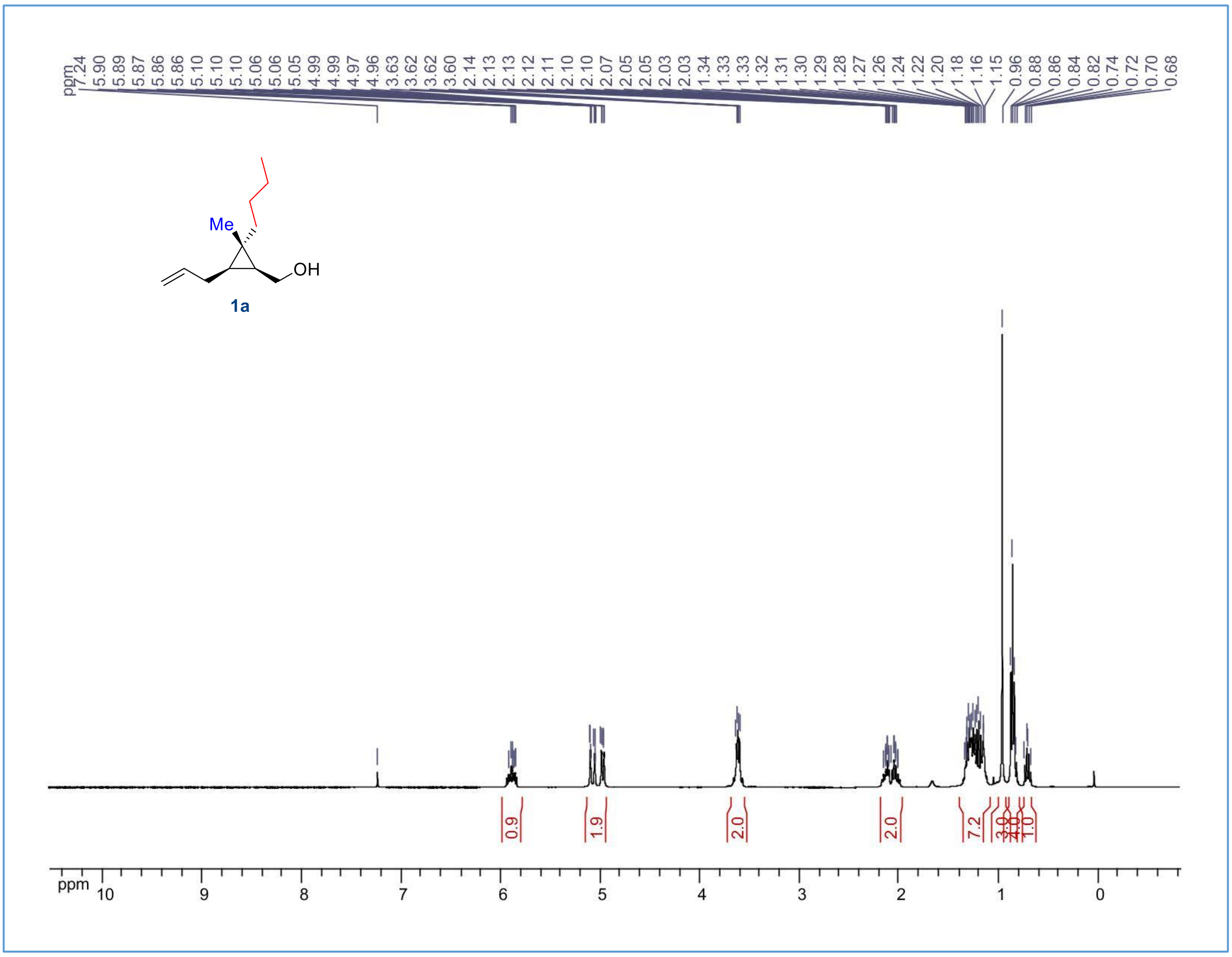

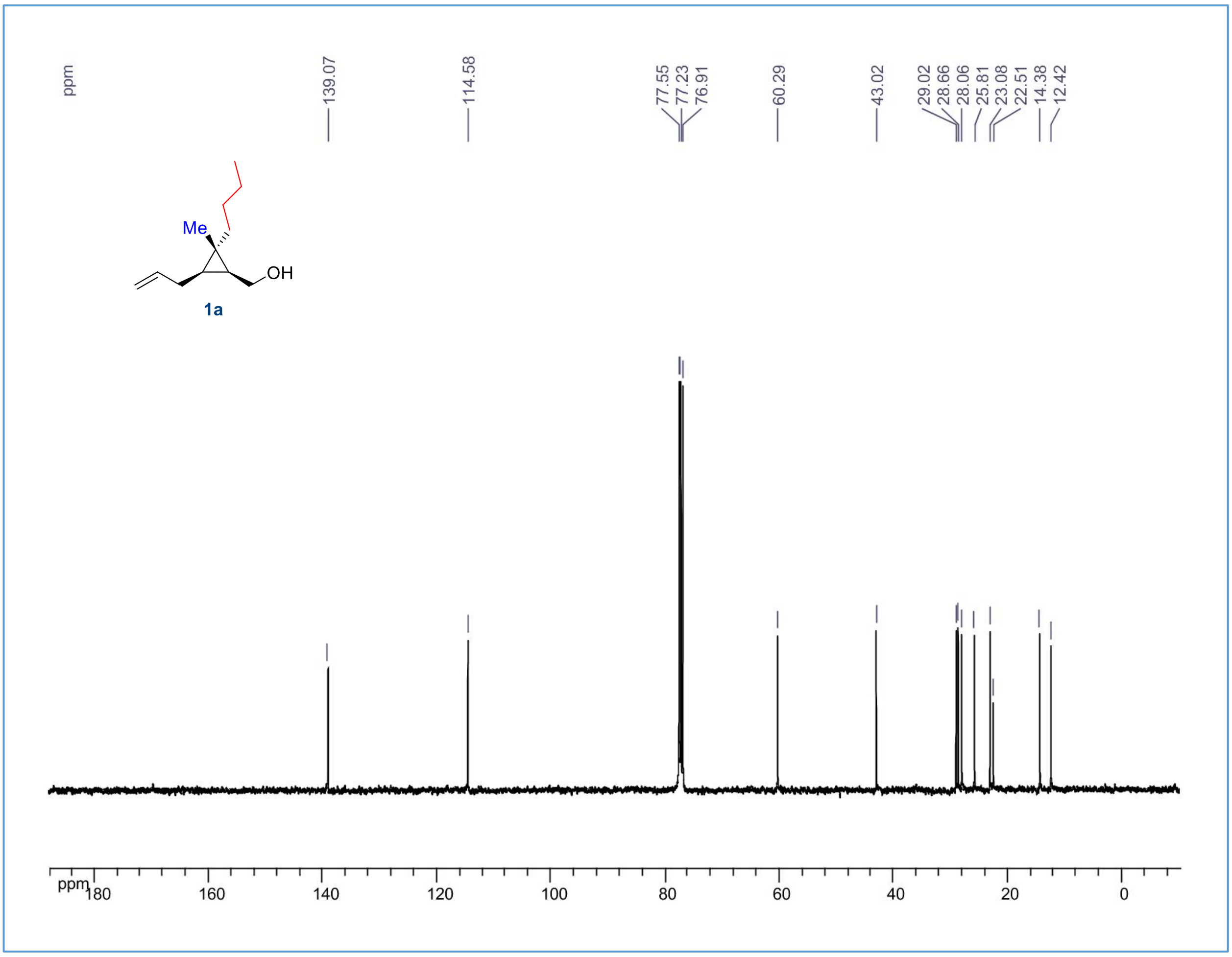

Supplementary Figure 1. ^{1}H and ^{13}C NMR spectra of compound **1a**

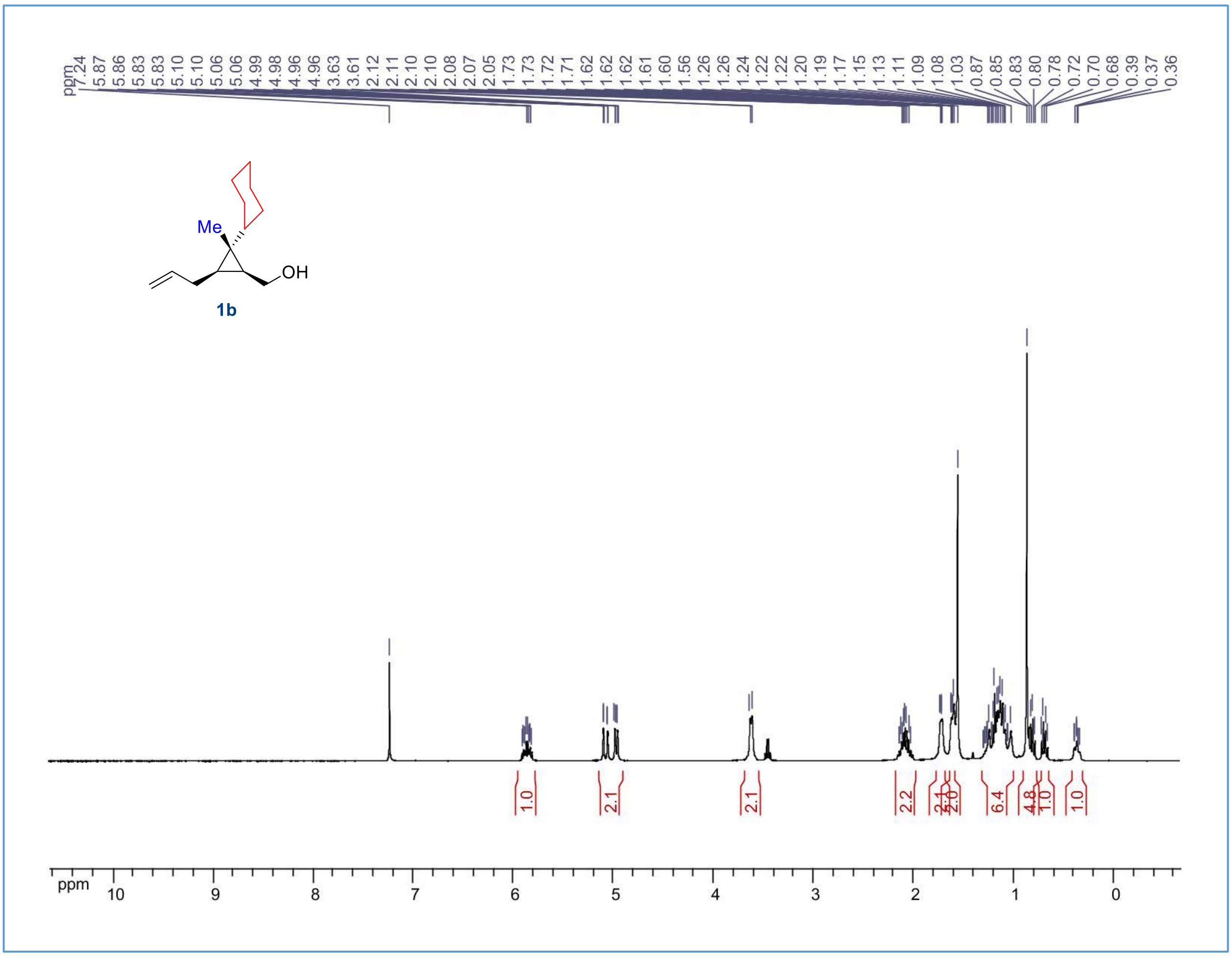

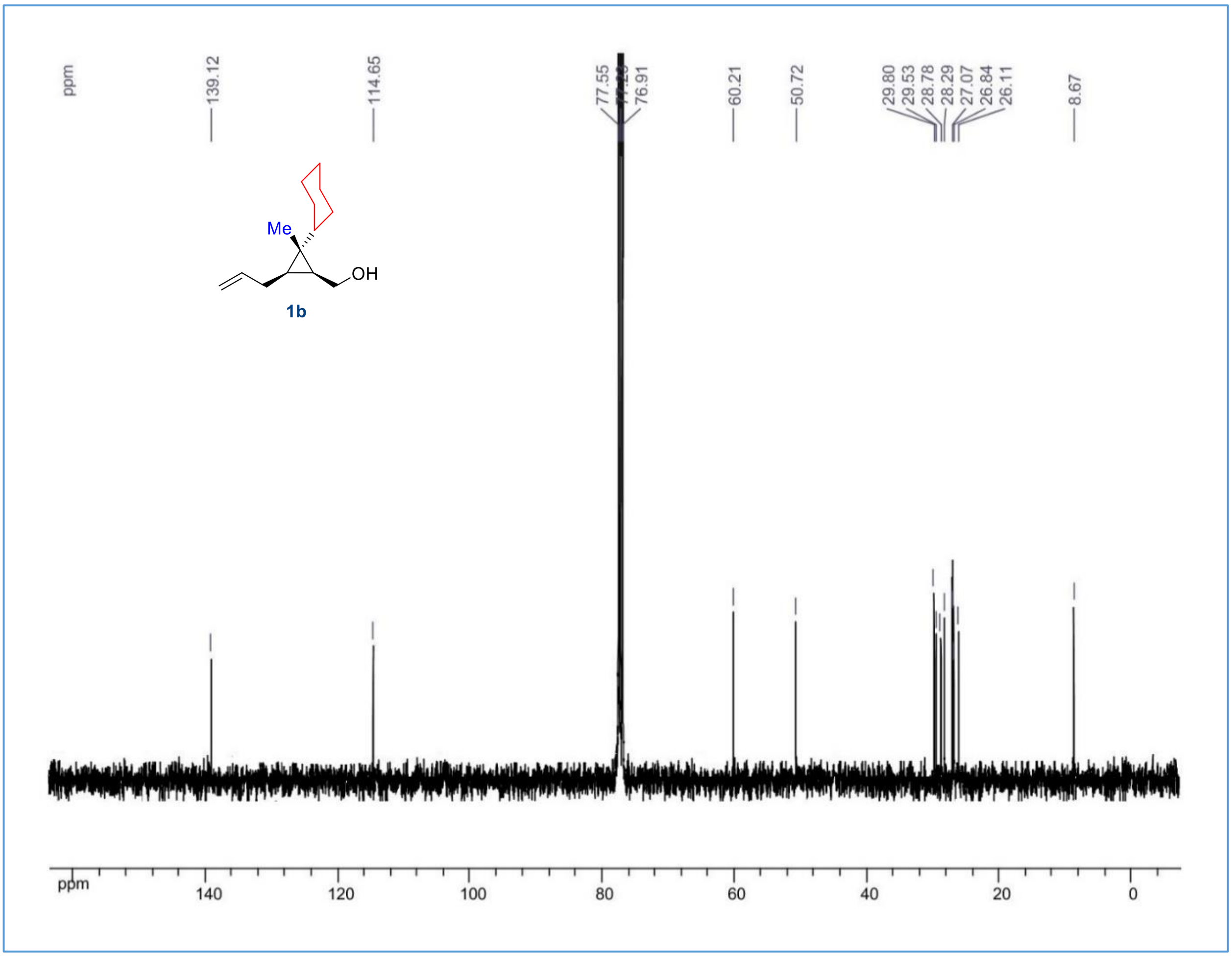

Supplementary Figure 2. ^{1}H and ^{13}C NMR spectra of compound **1b**

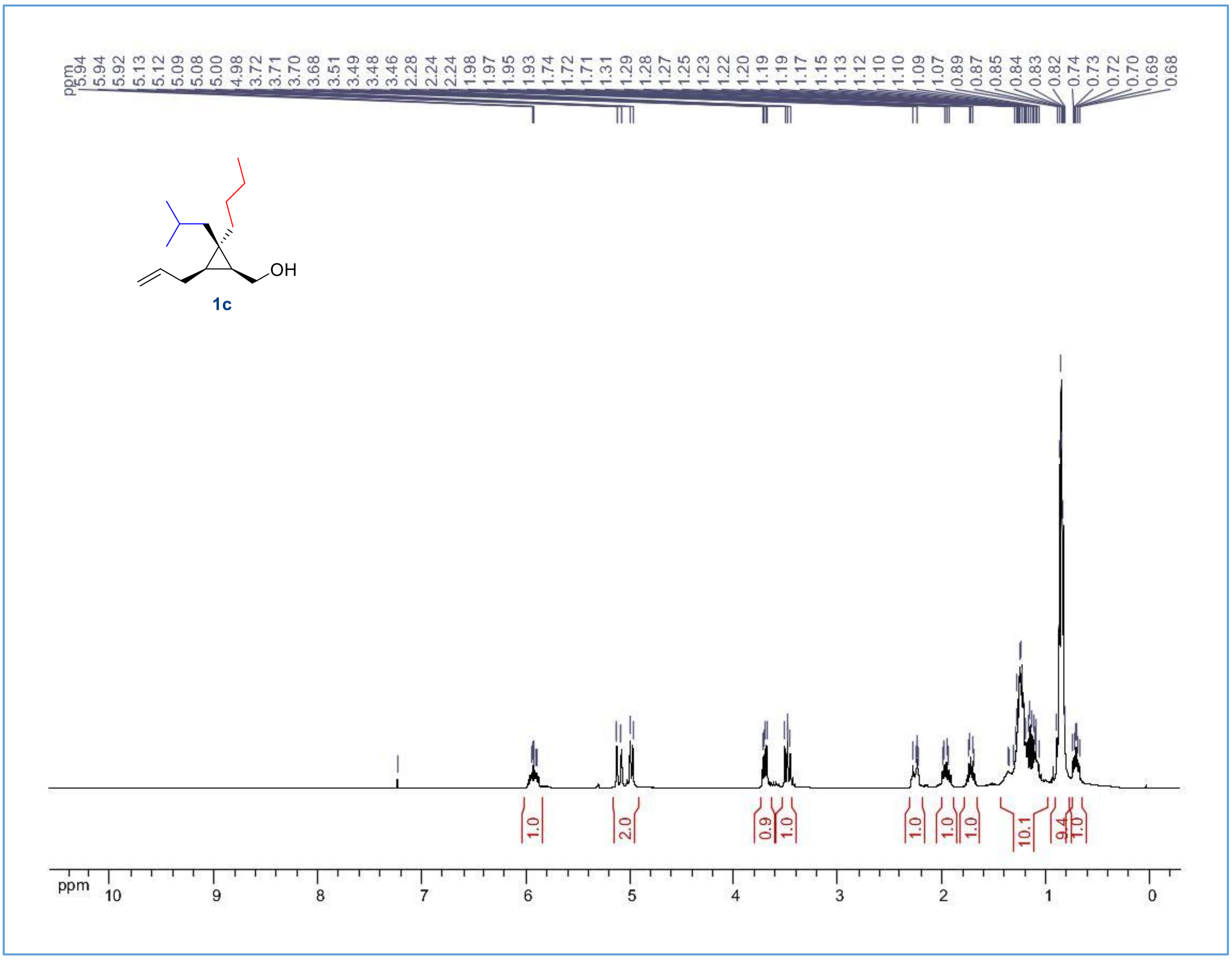

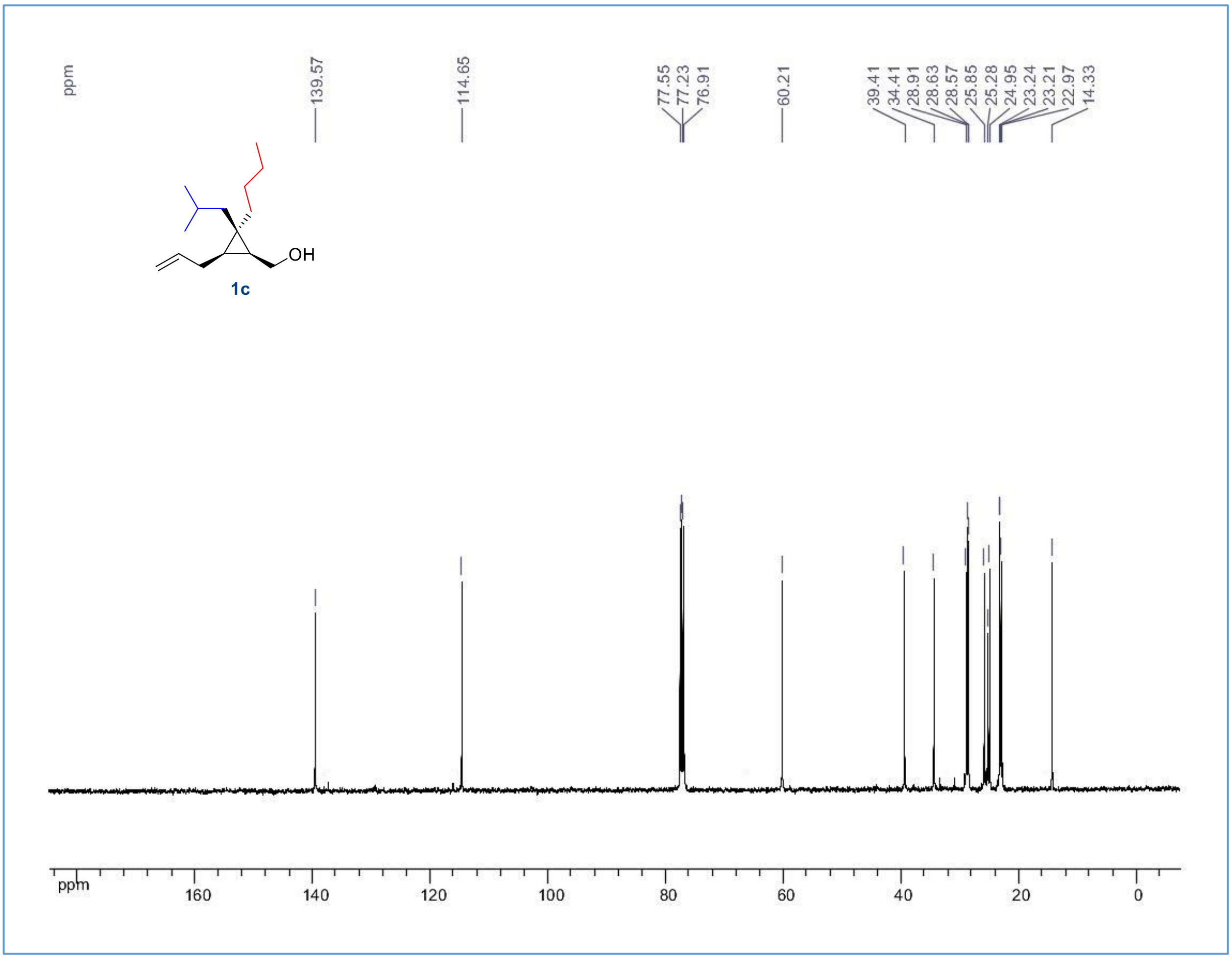

Supplementary Figure 3. ^{1}H and ^{13}C NMR spectra of compound **1c**

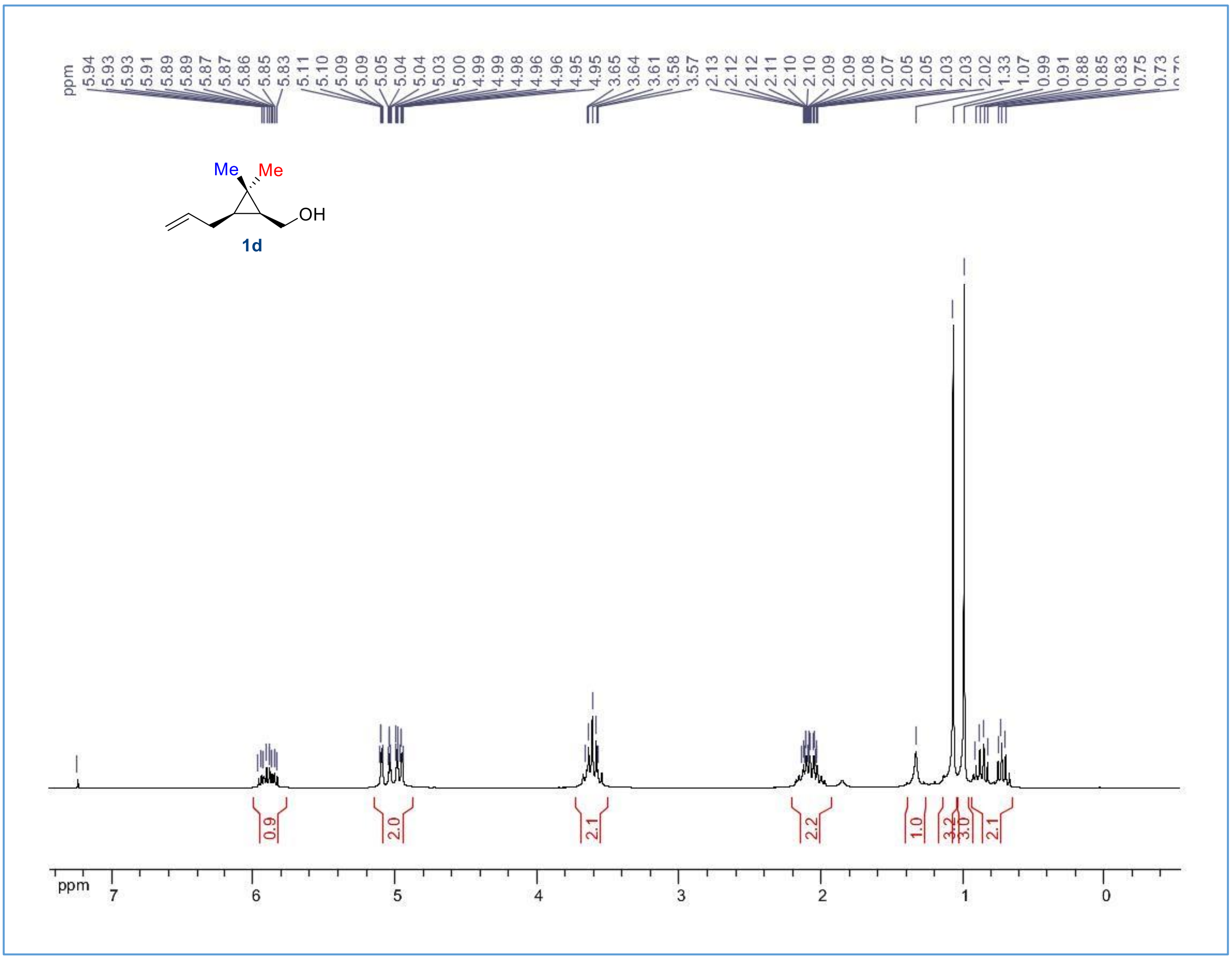

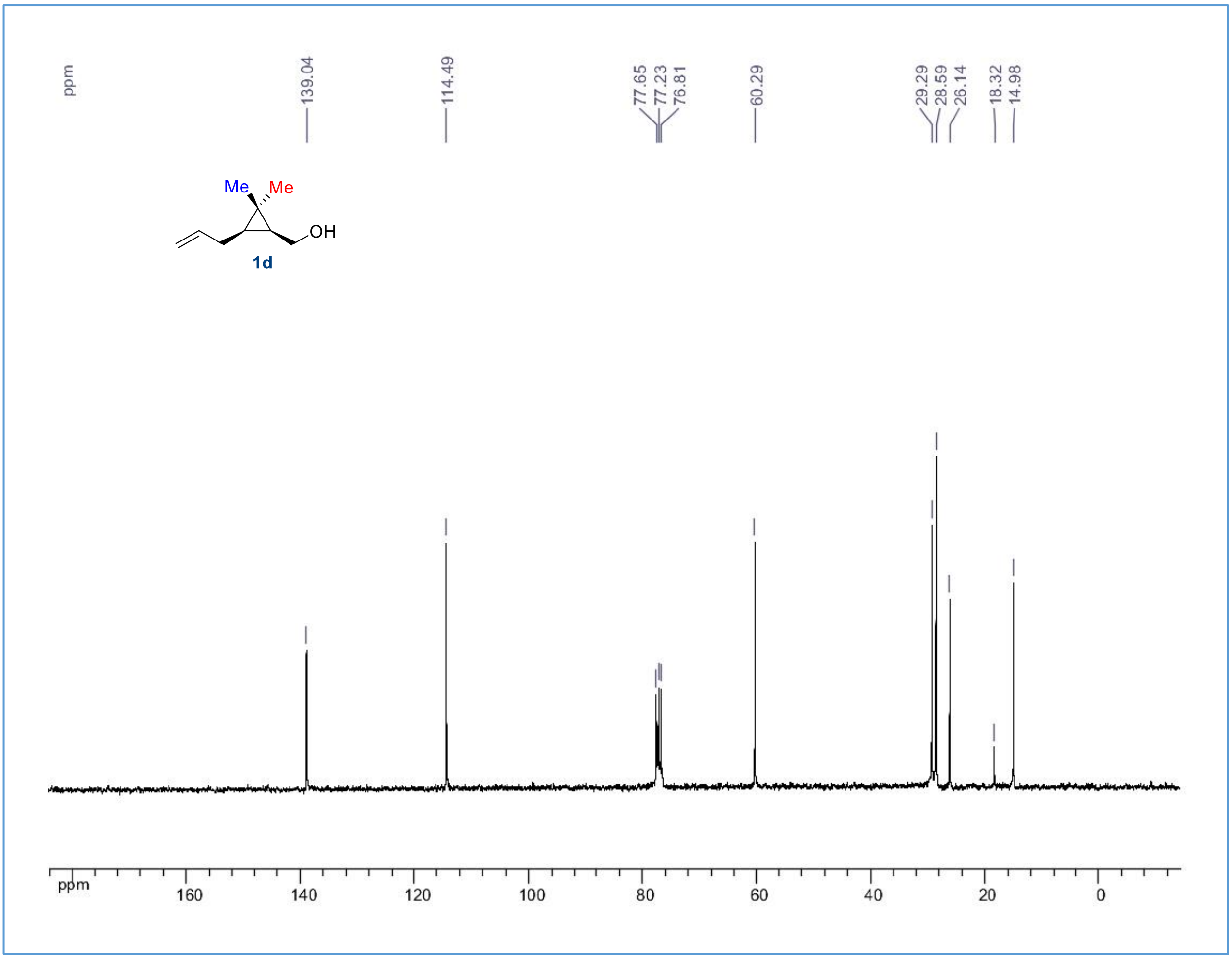

Supplementary Figure 4. ^{1}H and ^{13}C NMR spectra of compound **1d**

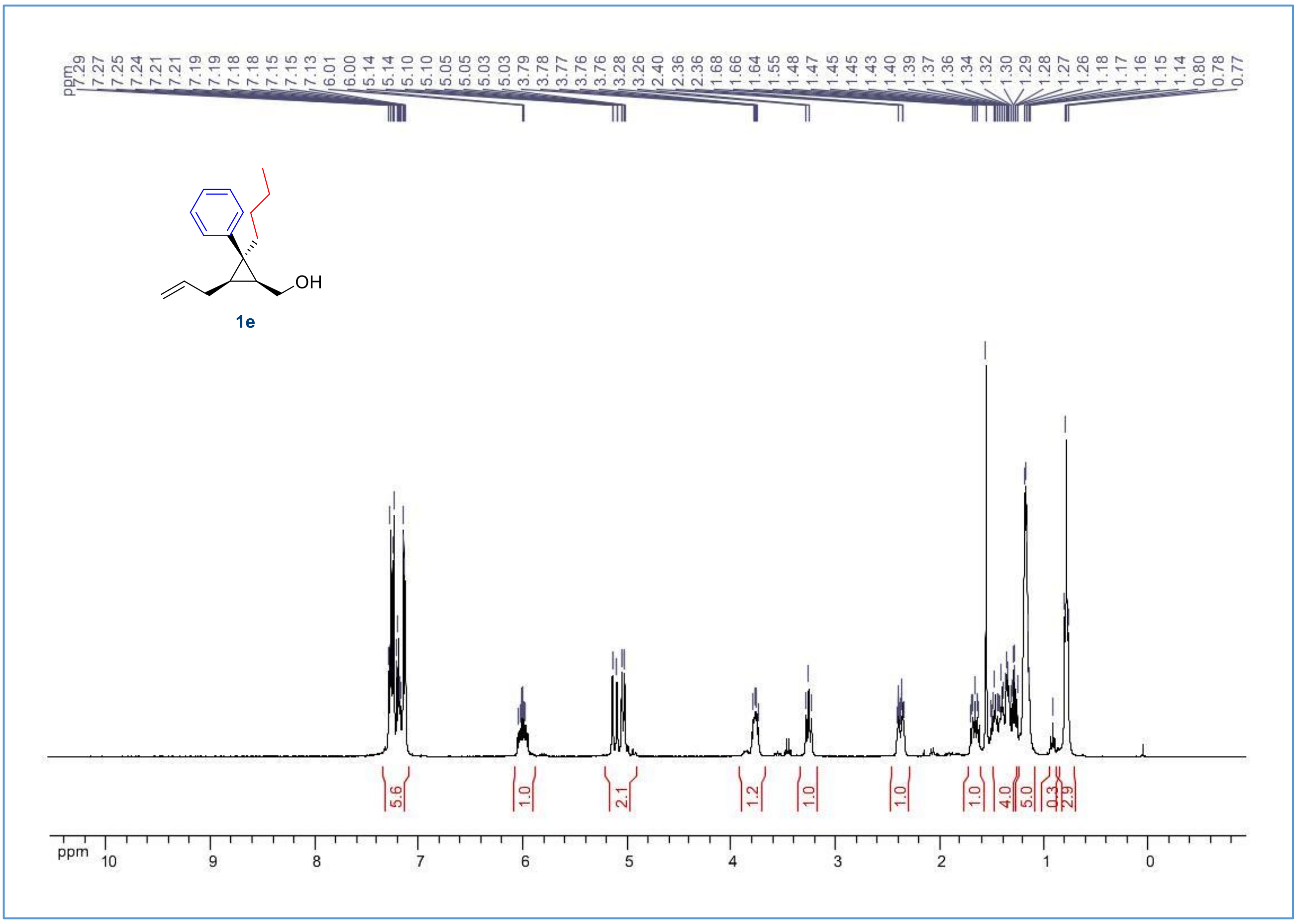

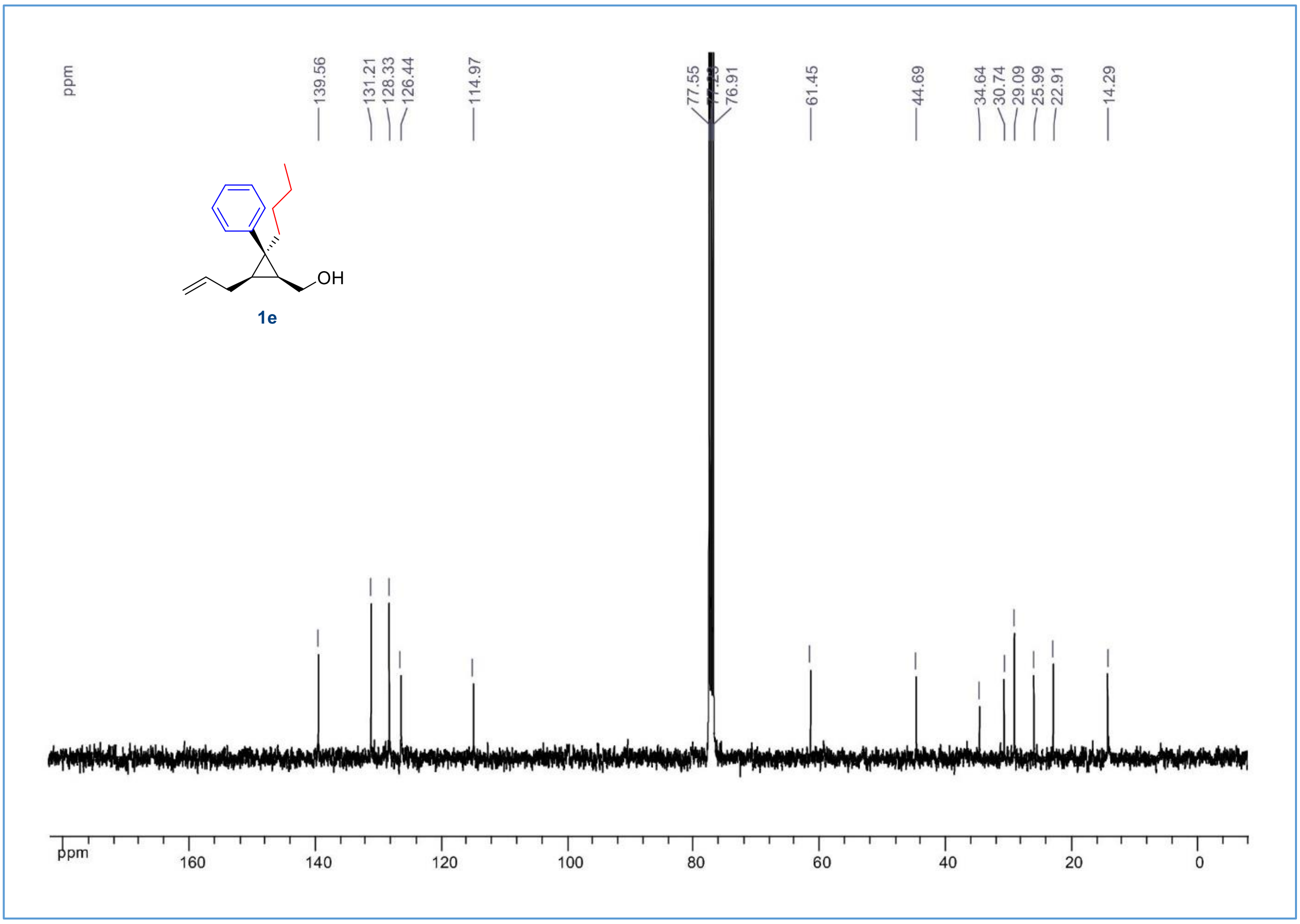

Supplementary Figure 5. ^{1}H and ^{13}C NMR spectra of compound **1e**

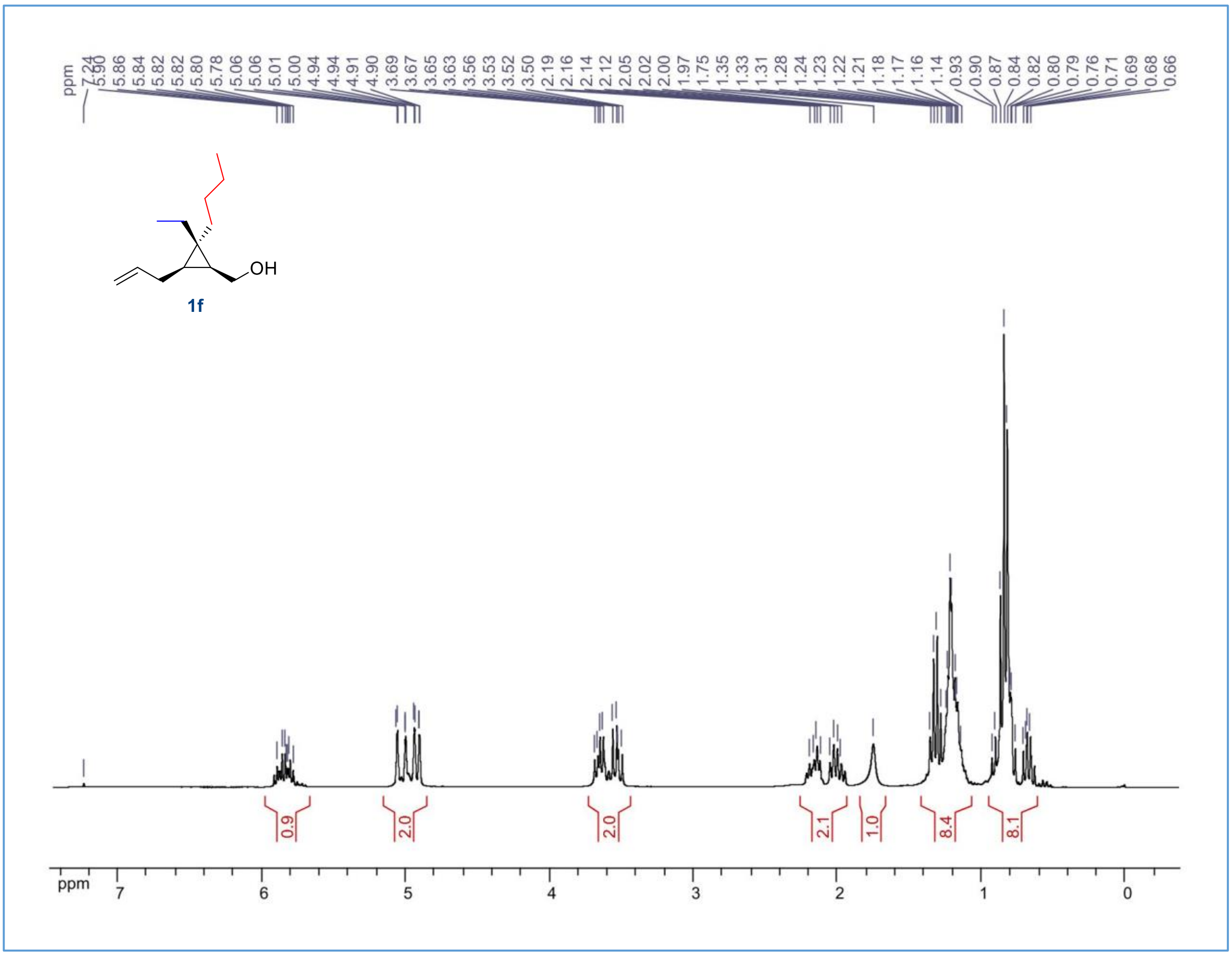

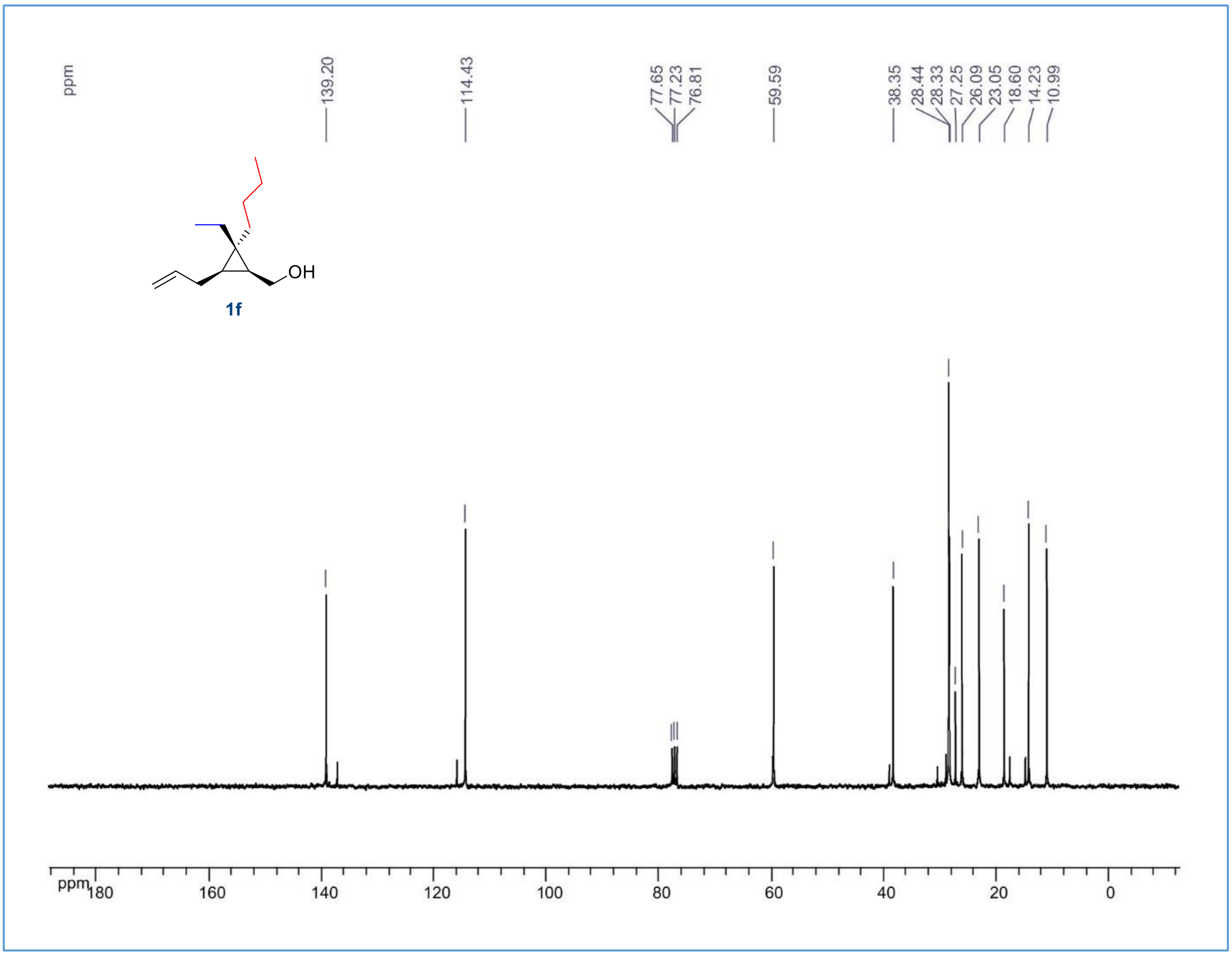

Supplementary Figure 6. ^{1}H and ^{13}C NMR spectra of compound **1f**

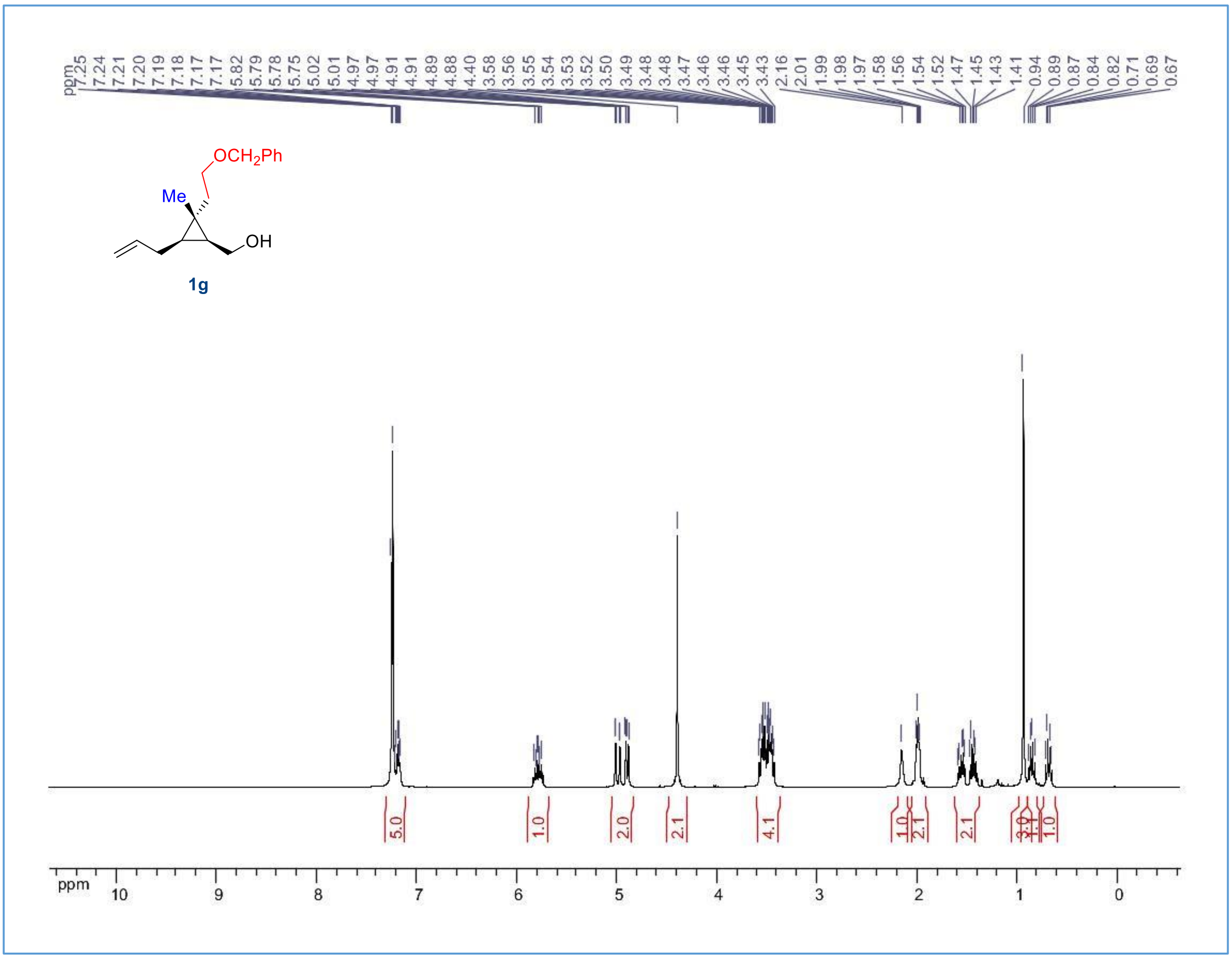

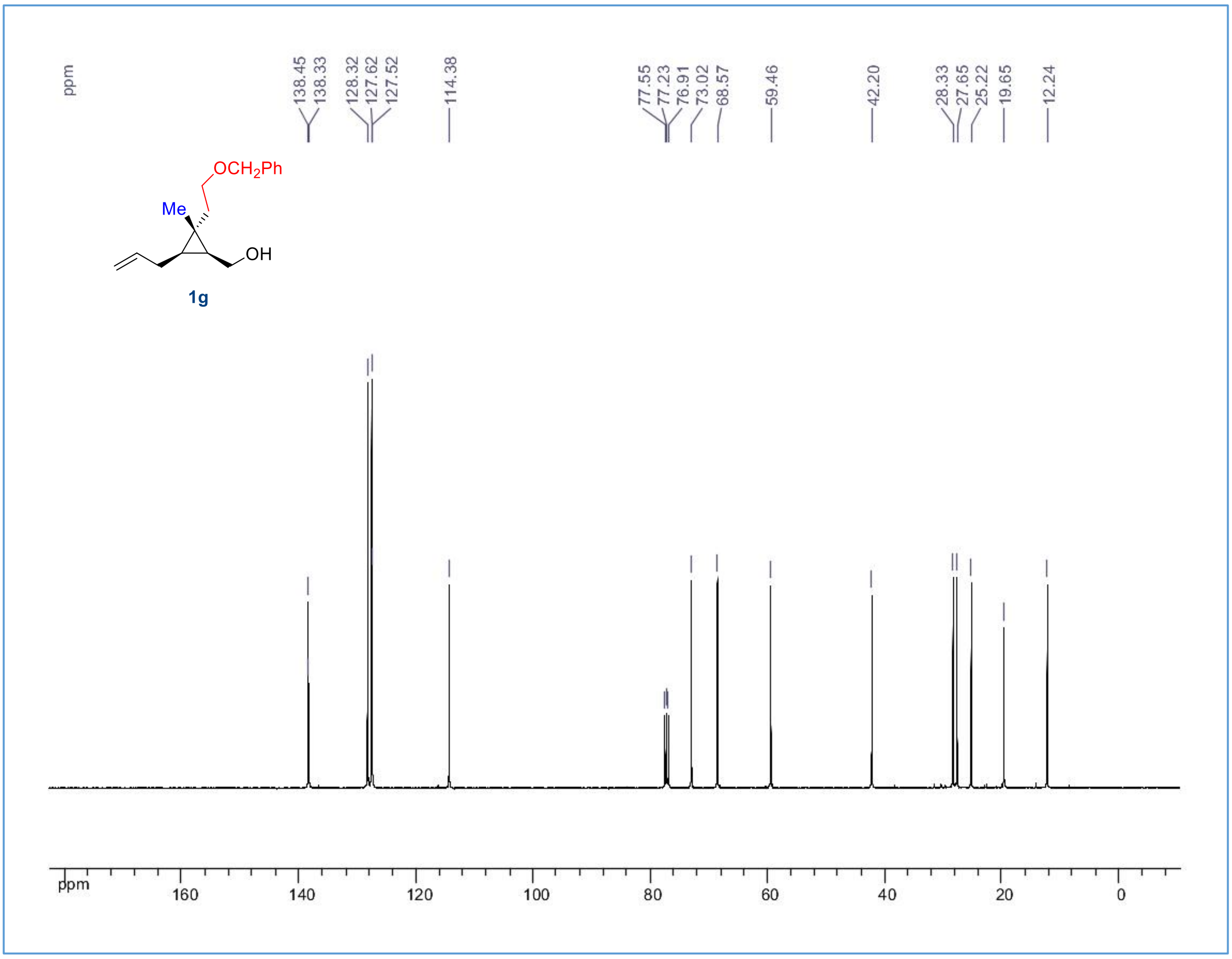

Supplementary Figure 7. ^{1}H and ^{13}C NMR spectra of compound **1g**

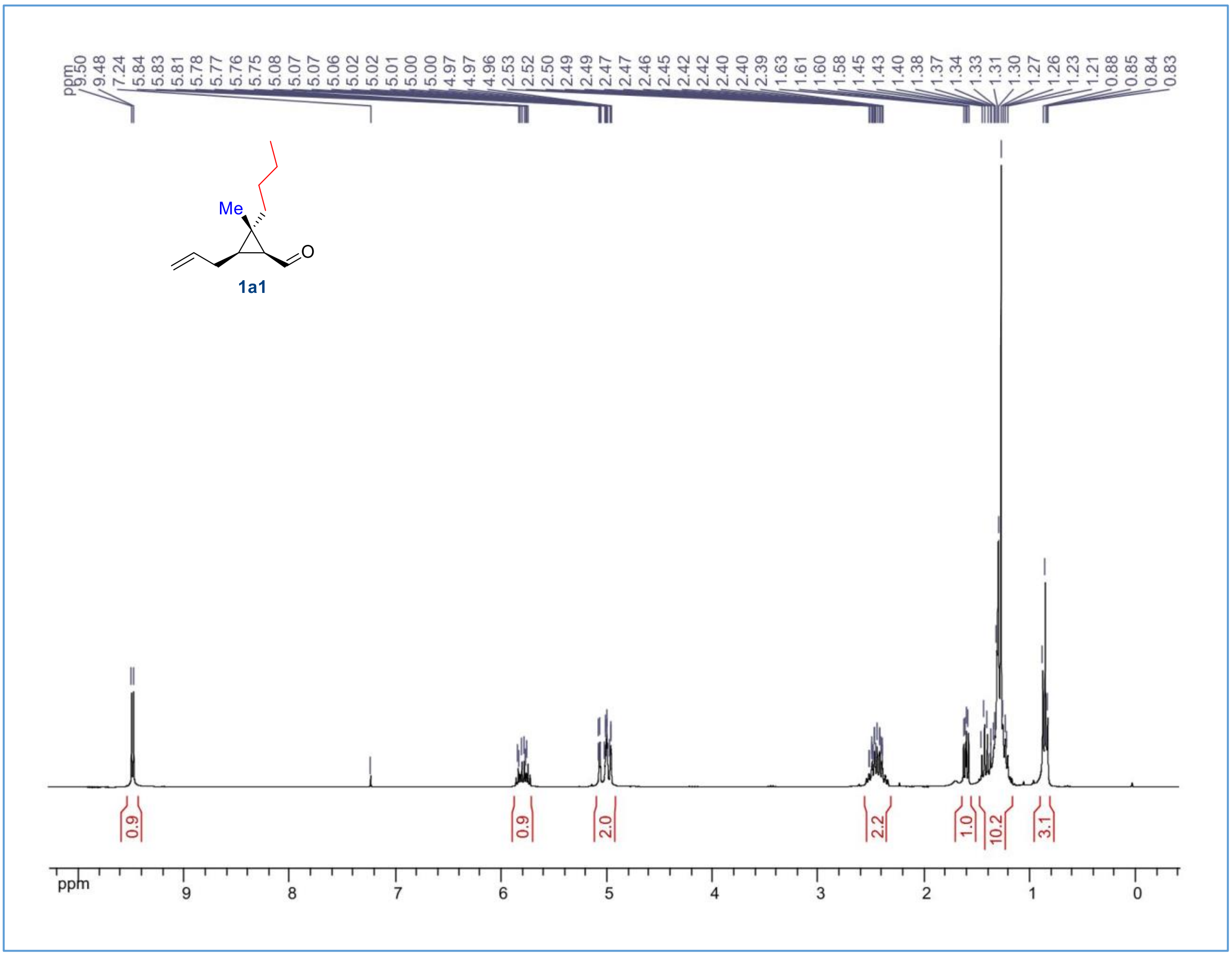

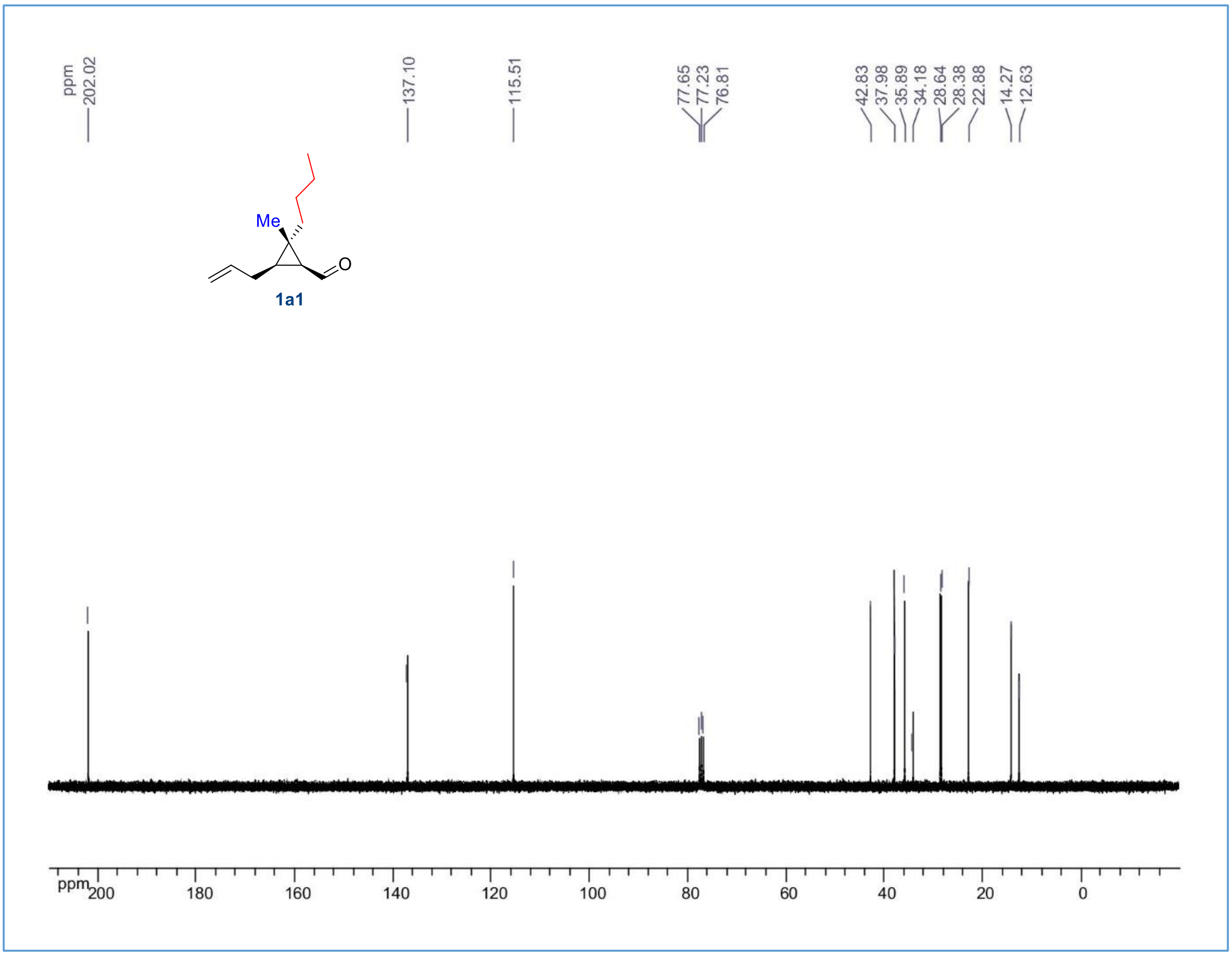

Supplementary Figure 8. ^{1}H and ^{13}C NMR spectra of compound **1a1**

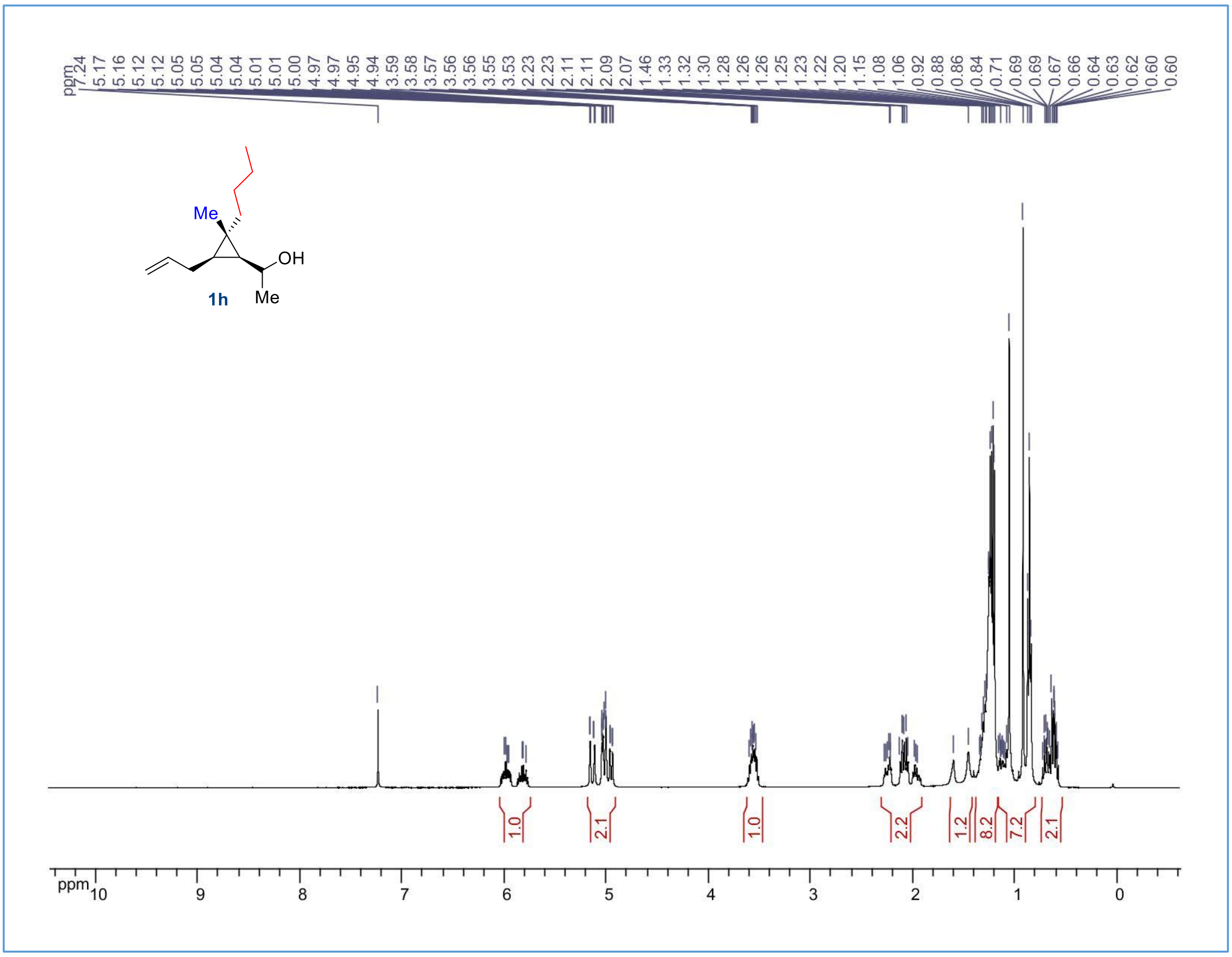

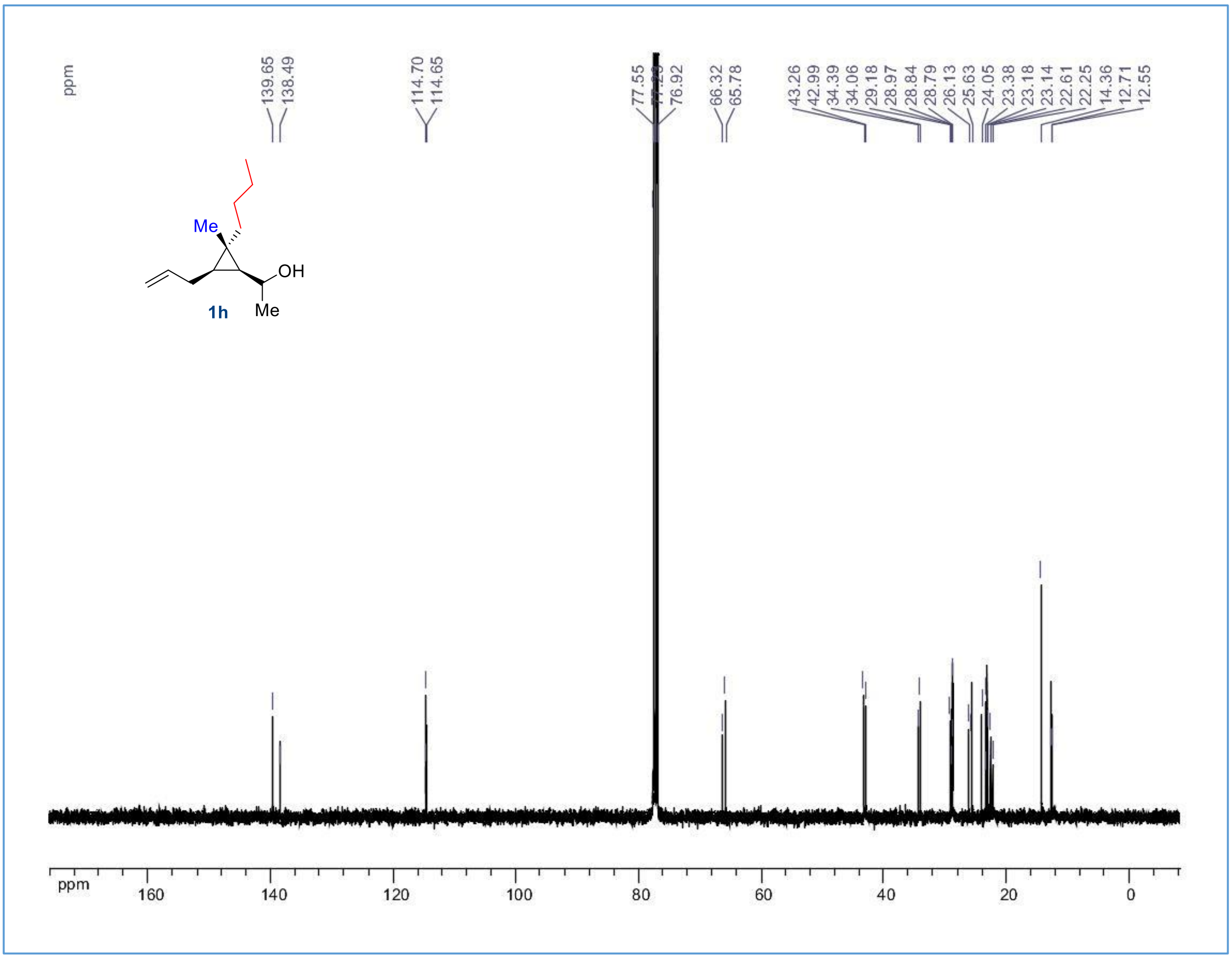

Supplementary Figure 9. ^{1}H and ^{13}C NMR spectra of compound **1h**

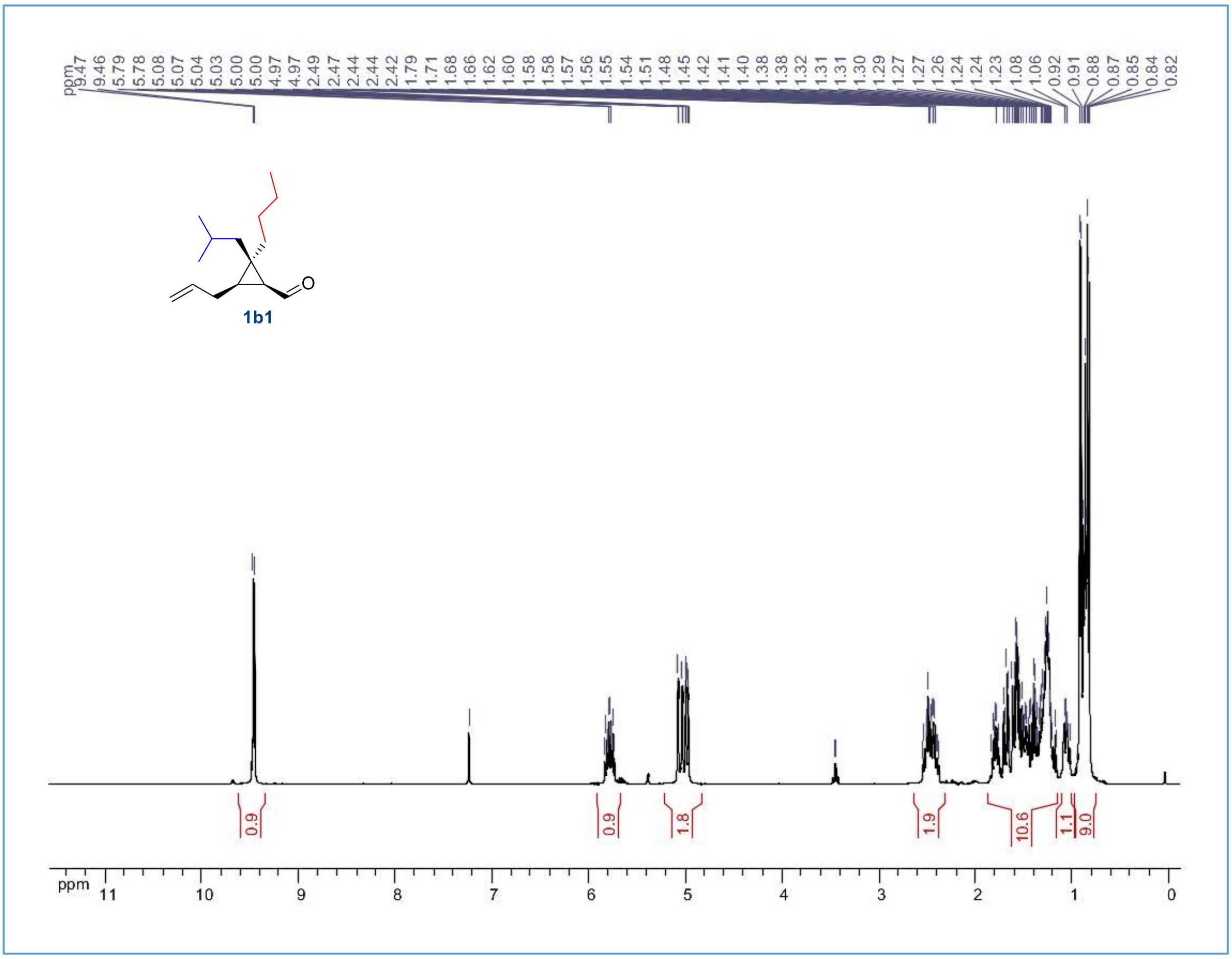

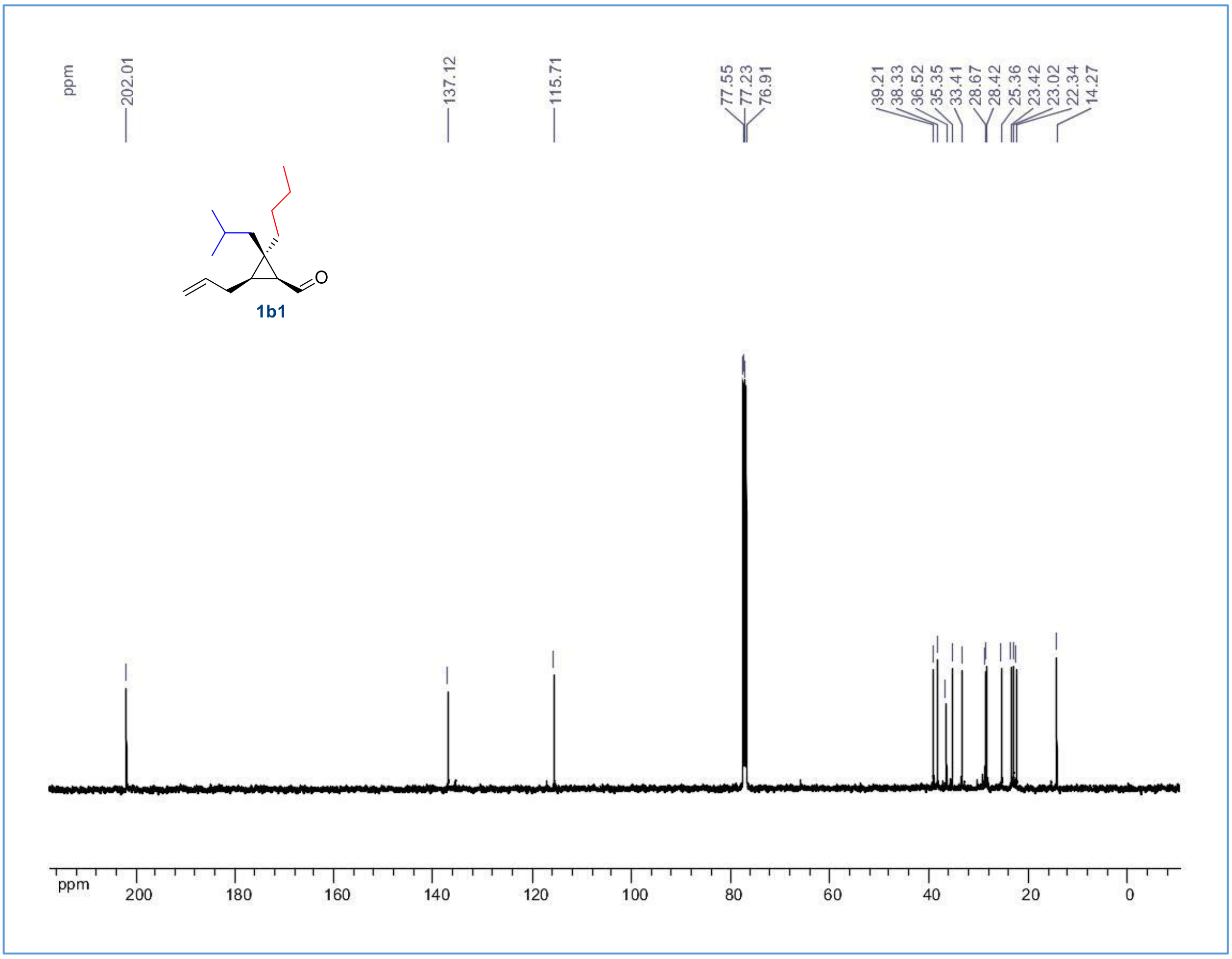

Supplementary Figure 10. ^{1}H and ^{13}C NMR spectra of compound **1b1**

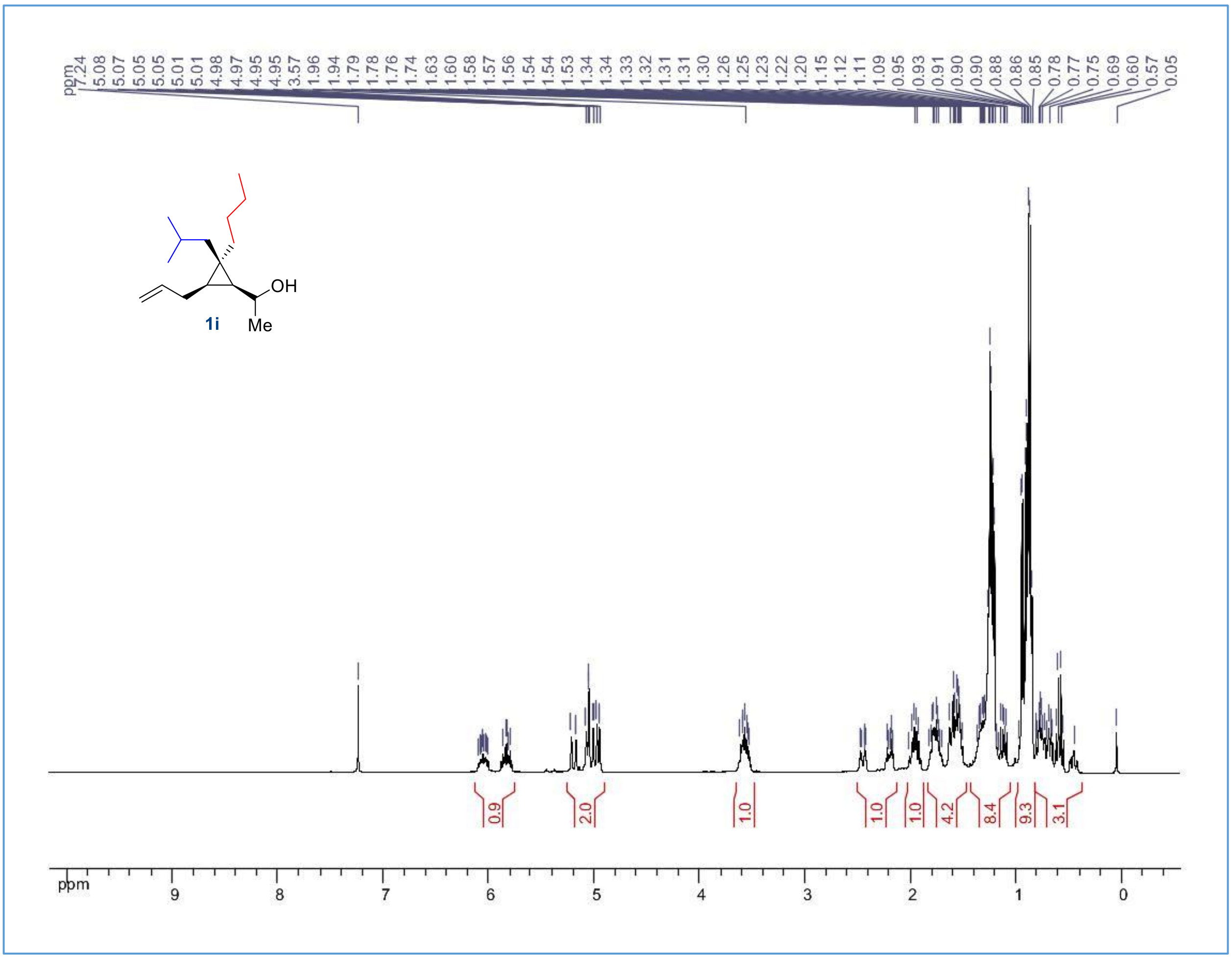

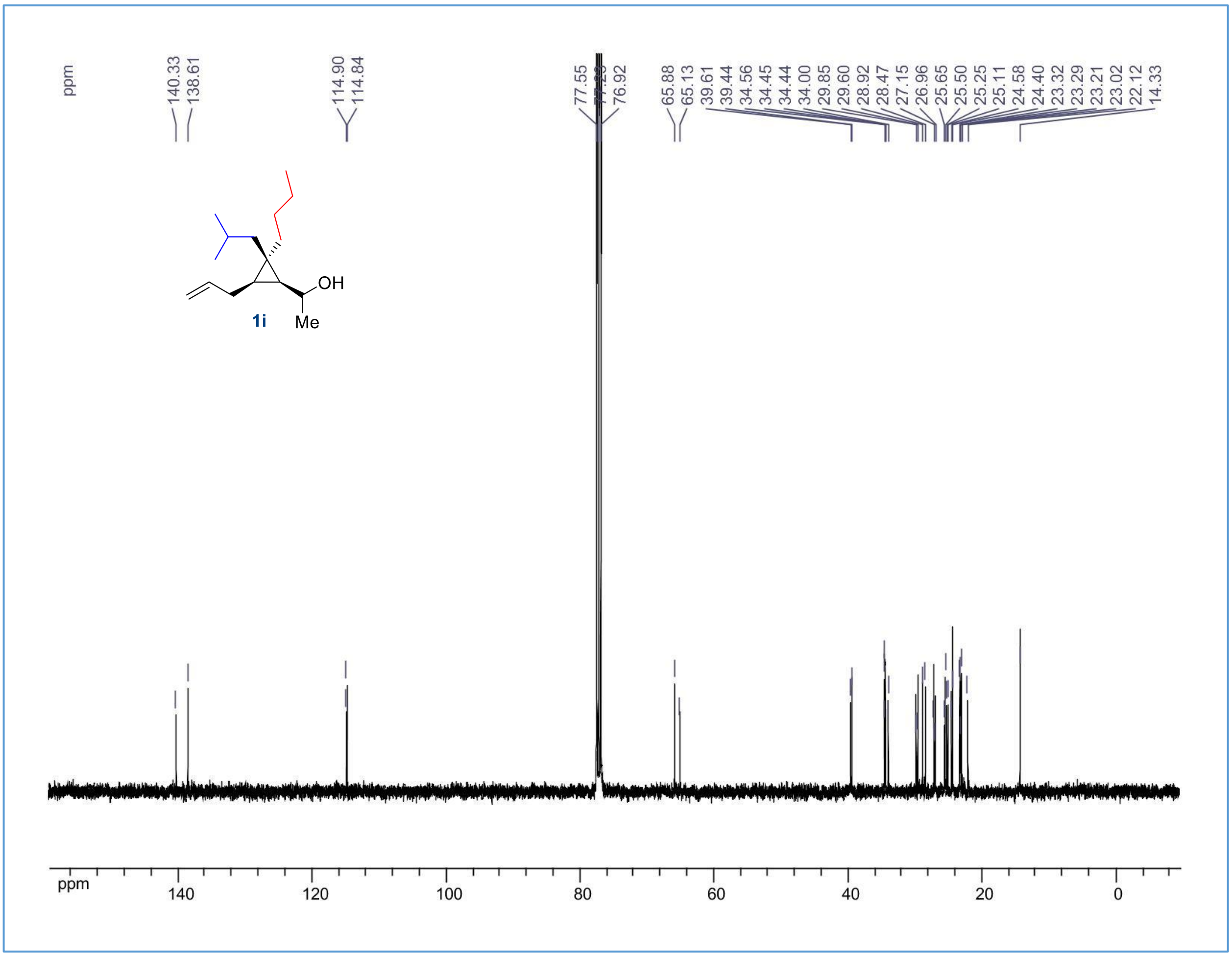

Supplementary Figure 11. ^{1}H and ^{13}C NMR spectra of compound **1i**

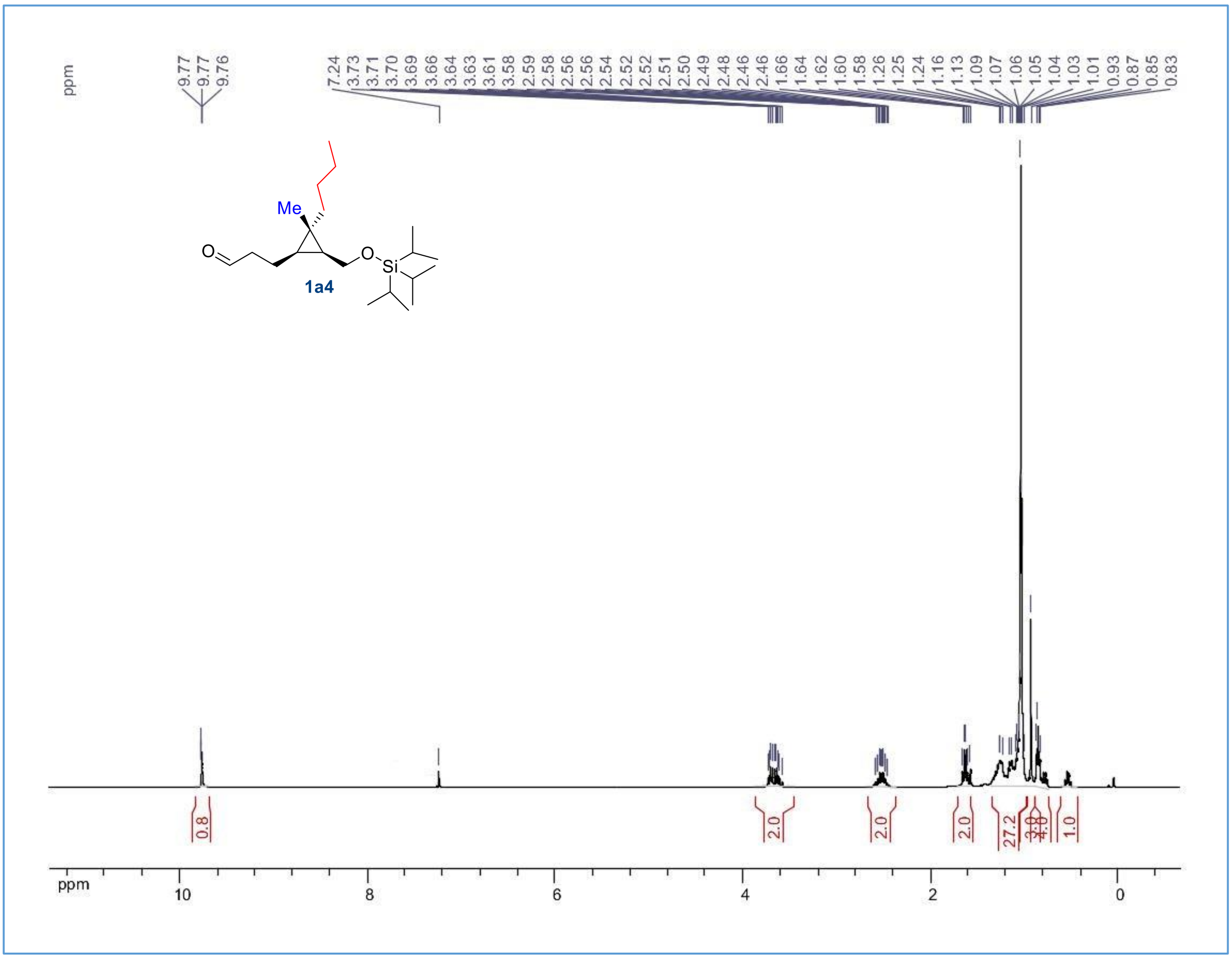

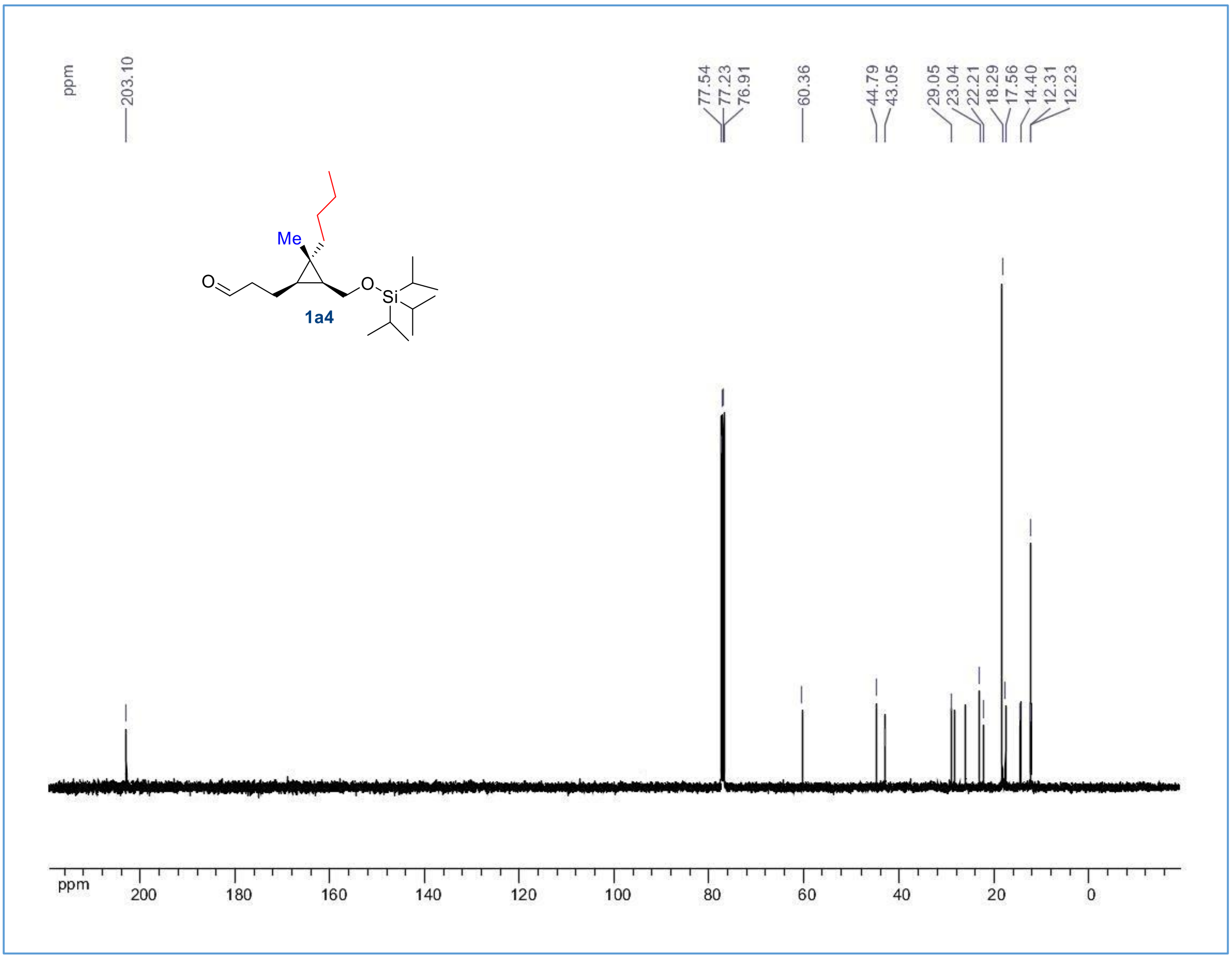

Supplementary Figure 12. ^{1}H and ^{13}C NMR spectra of compound **1a4**

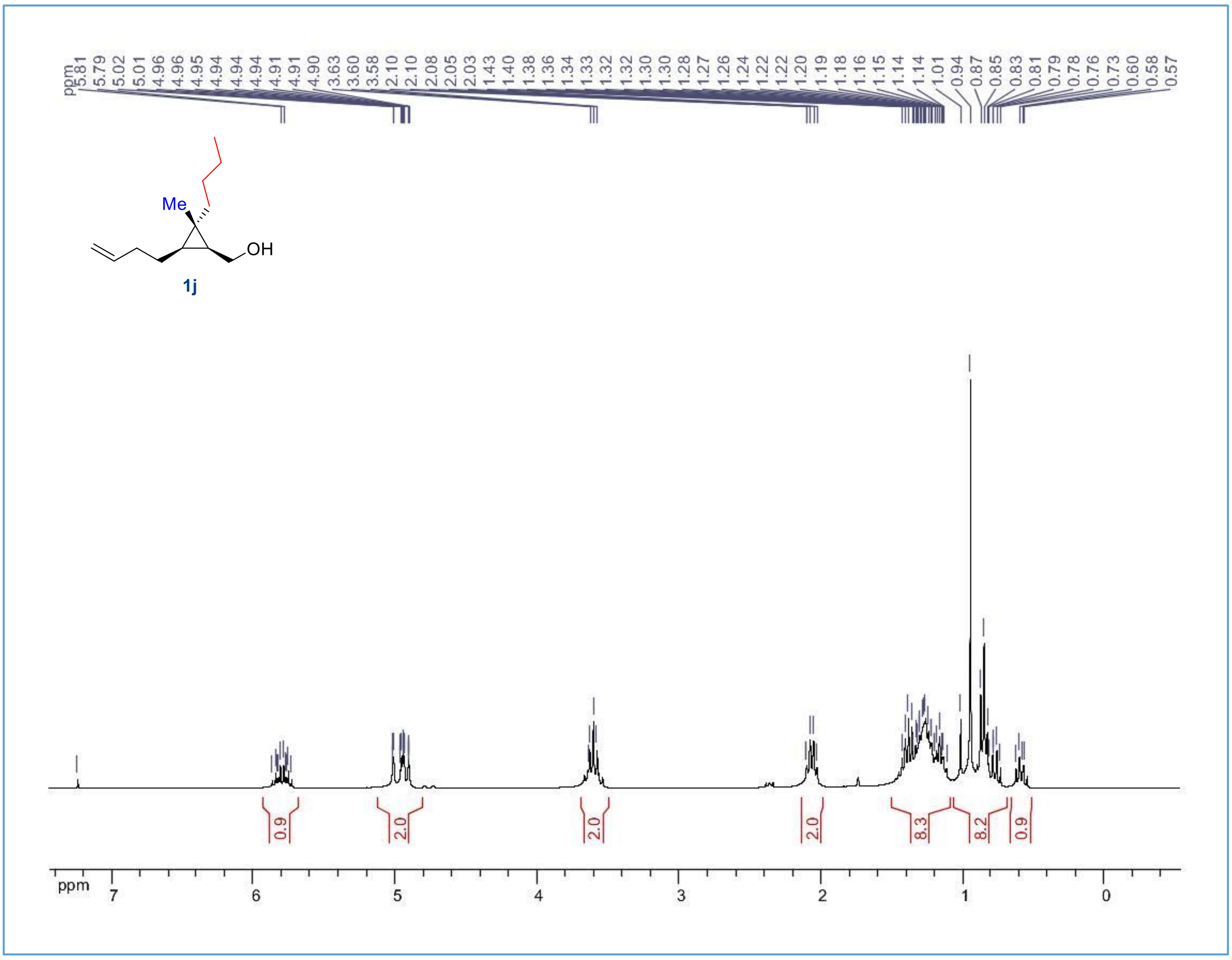

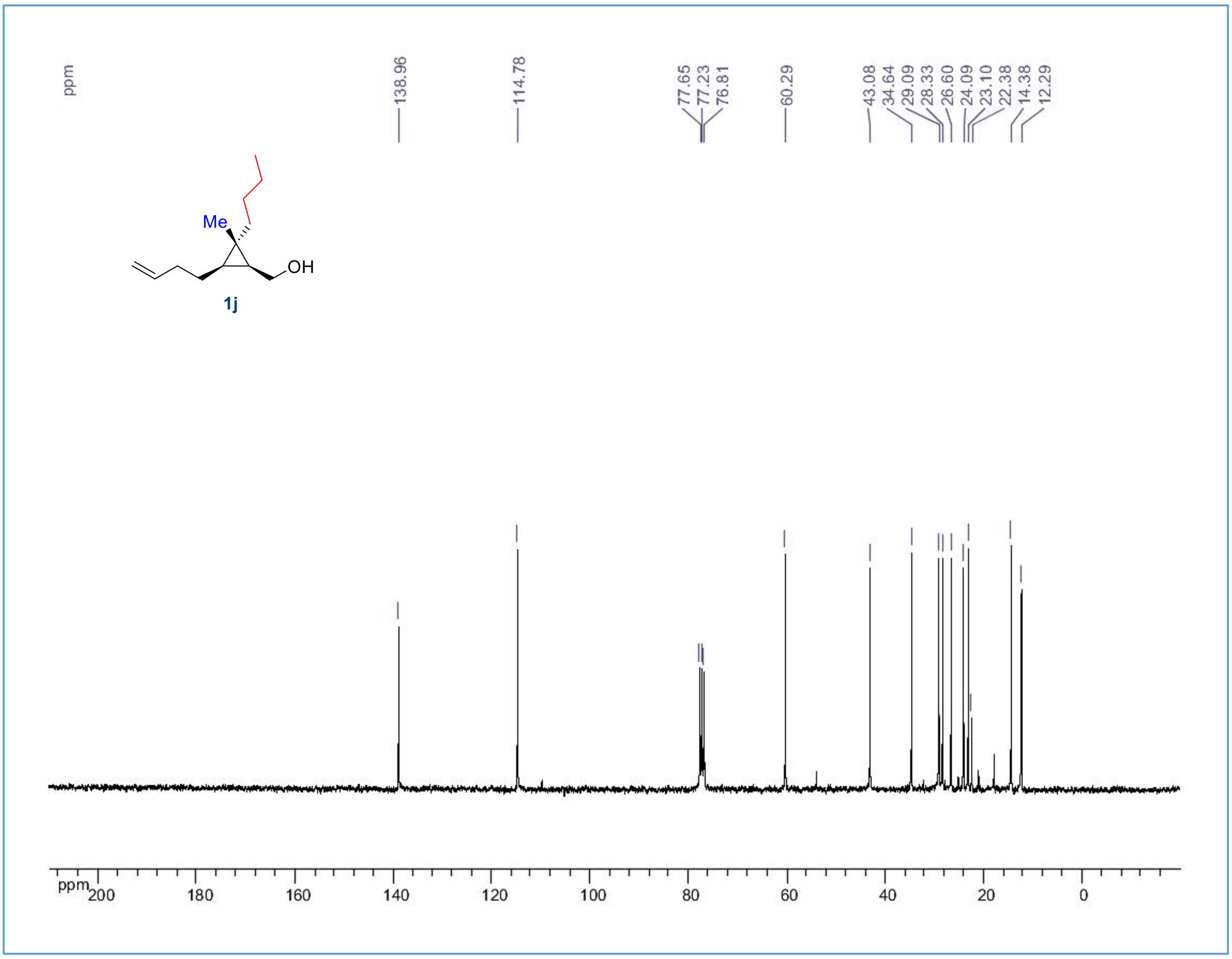

Supplementary Figure 13. ^{1}H and ^{13}C NMR spectra of compound **1j**

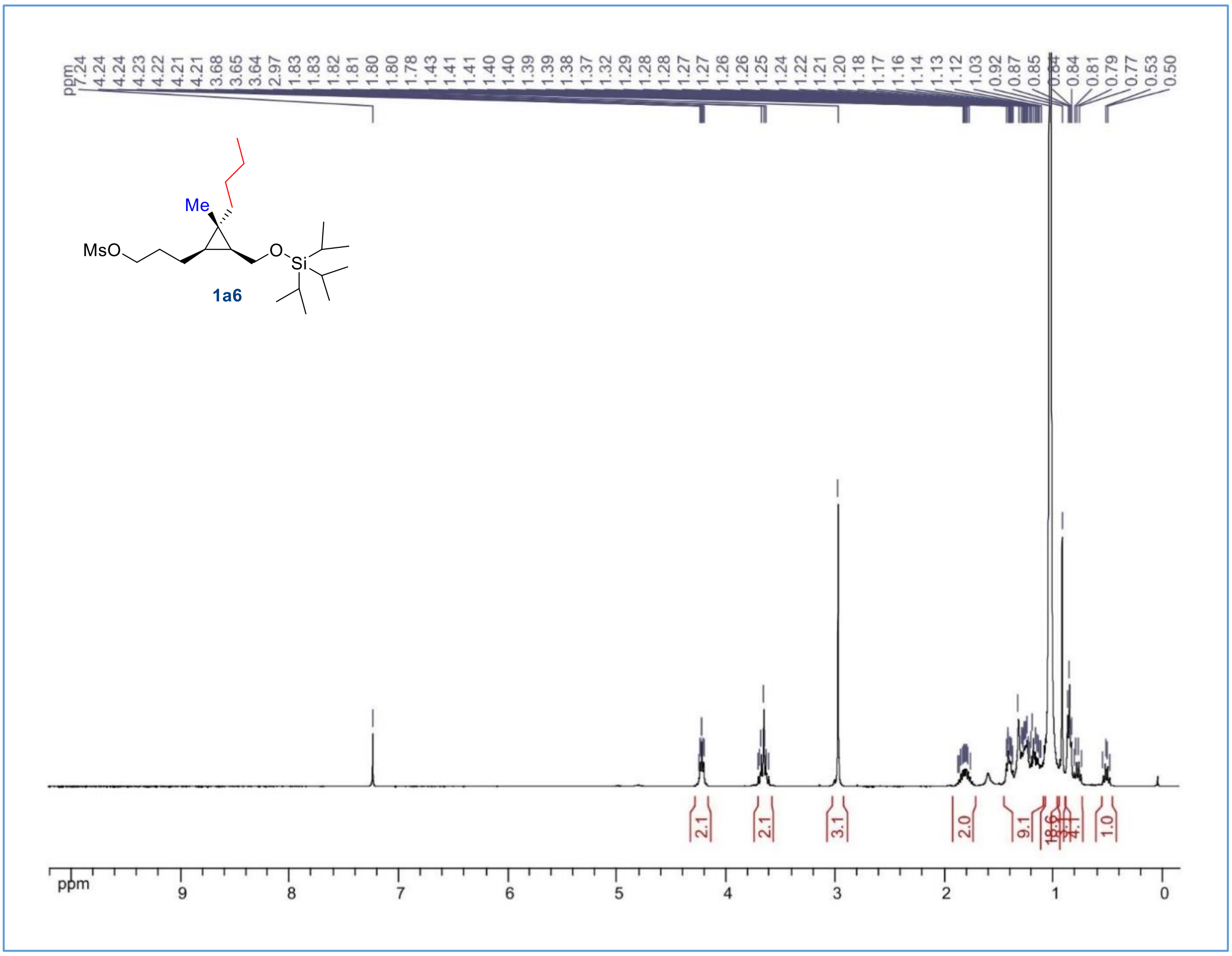

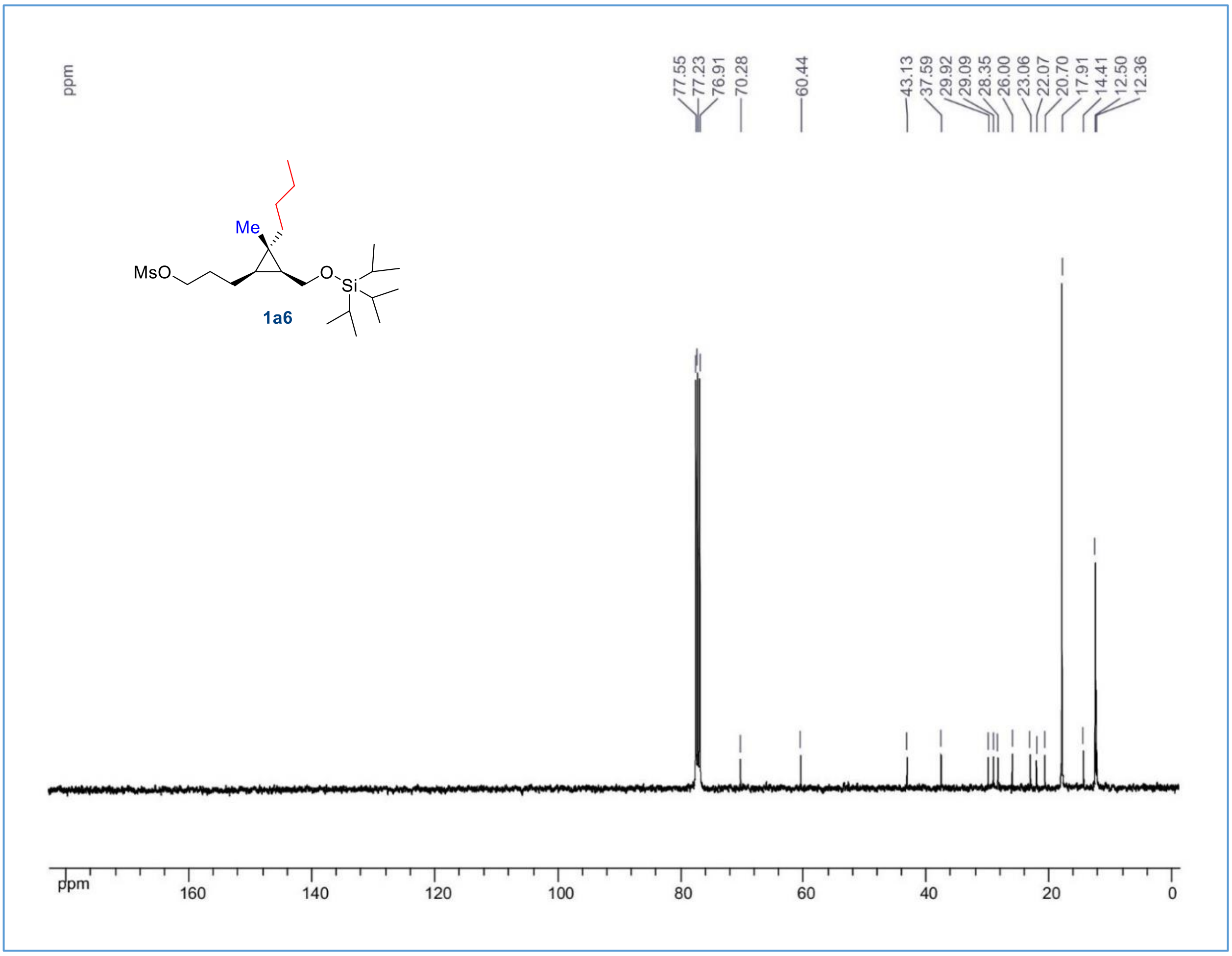

Supplementary Figure 14. ^{1}H and ^{13}C NMR spectra of compound **1a6**

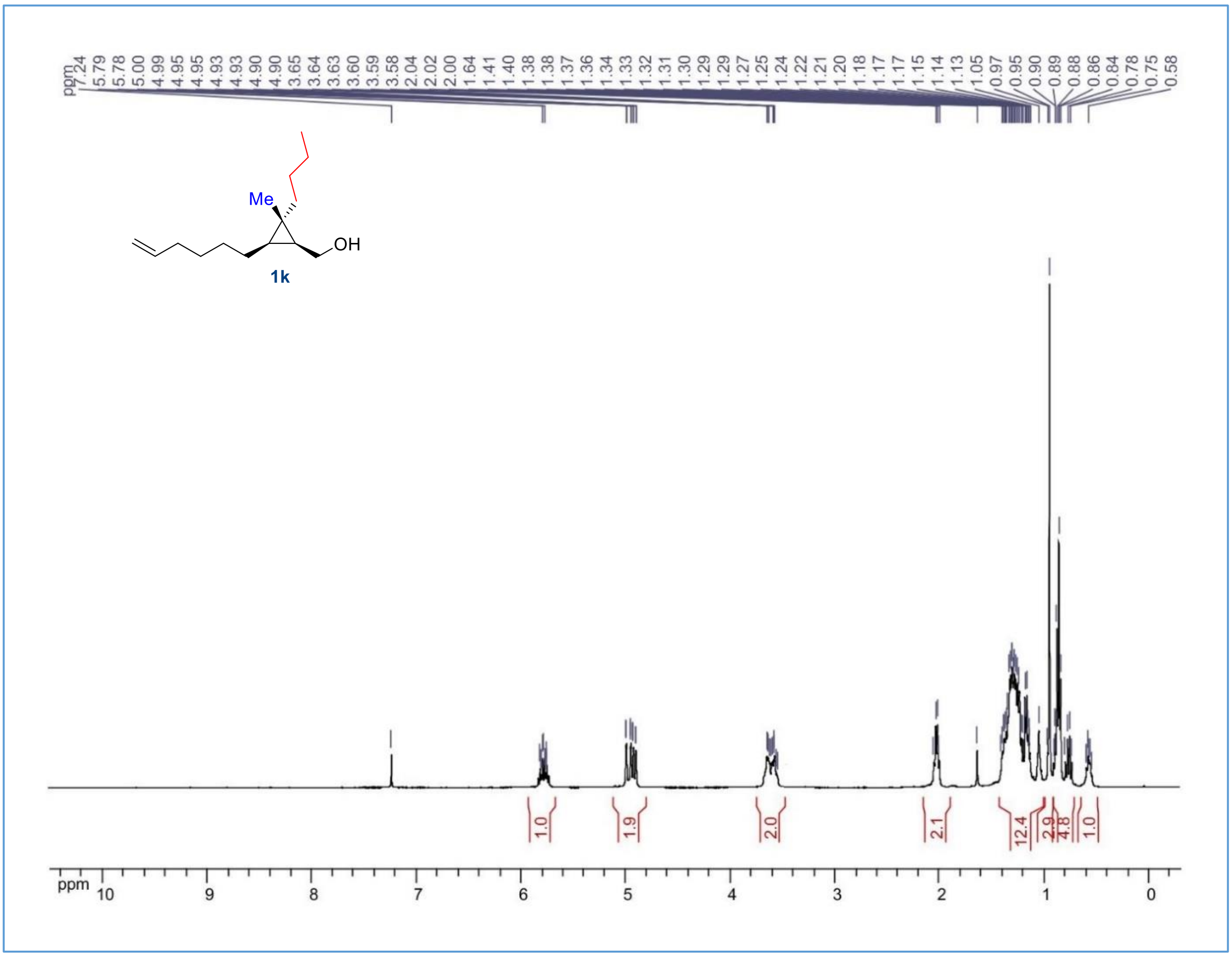

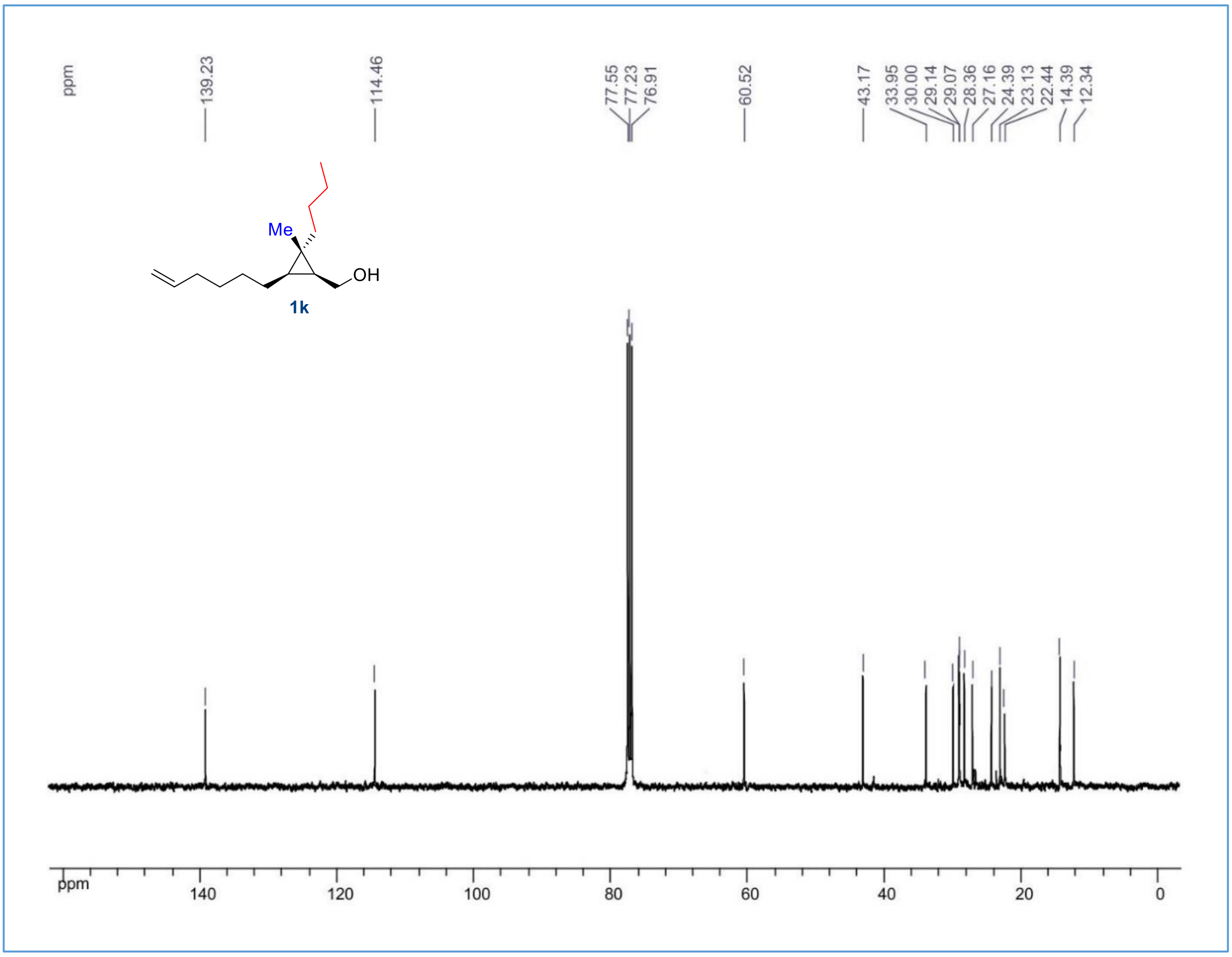

Supplementary Figure 15. ^{1}H and ^{13}C NMR spectra of compound **1k**

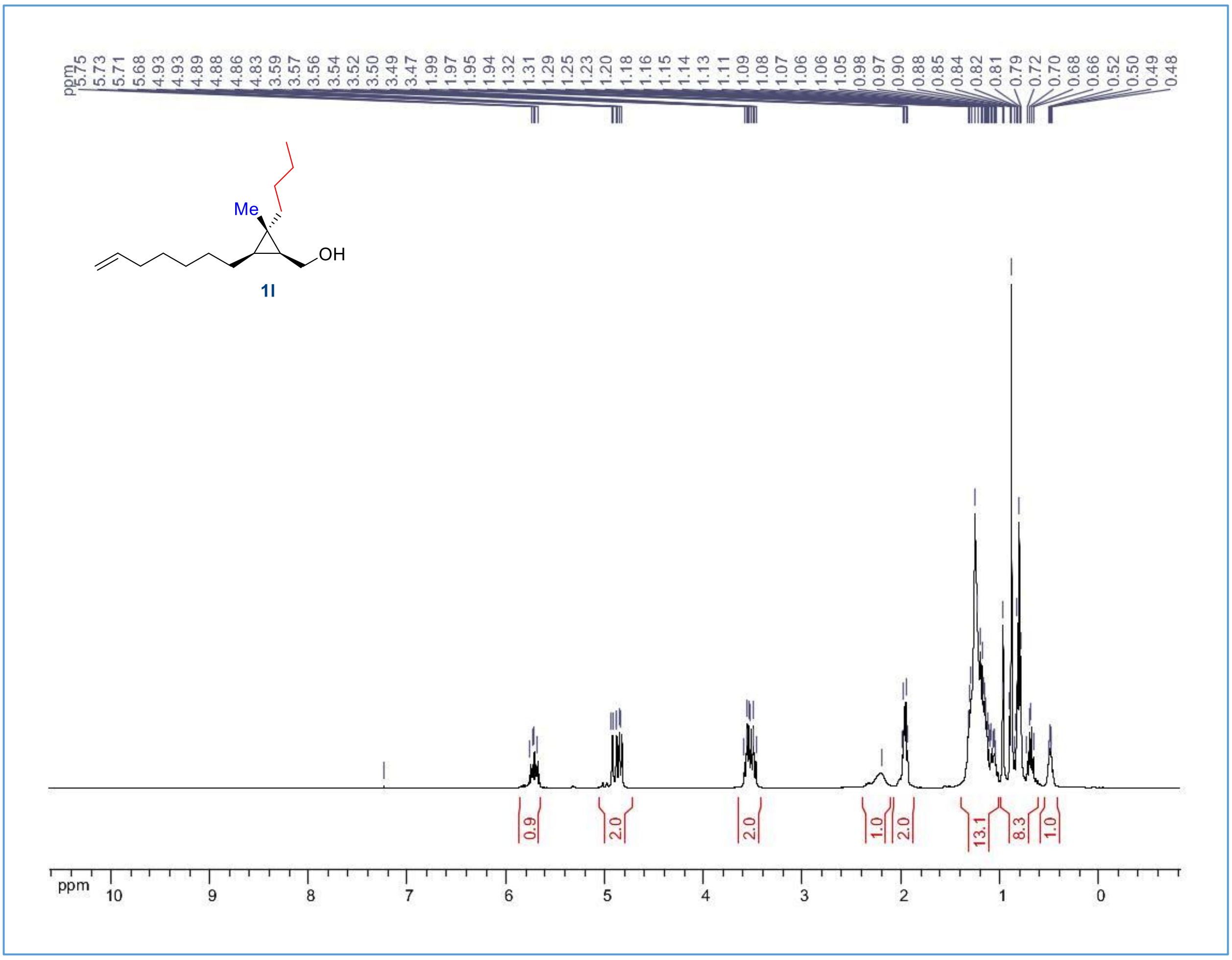

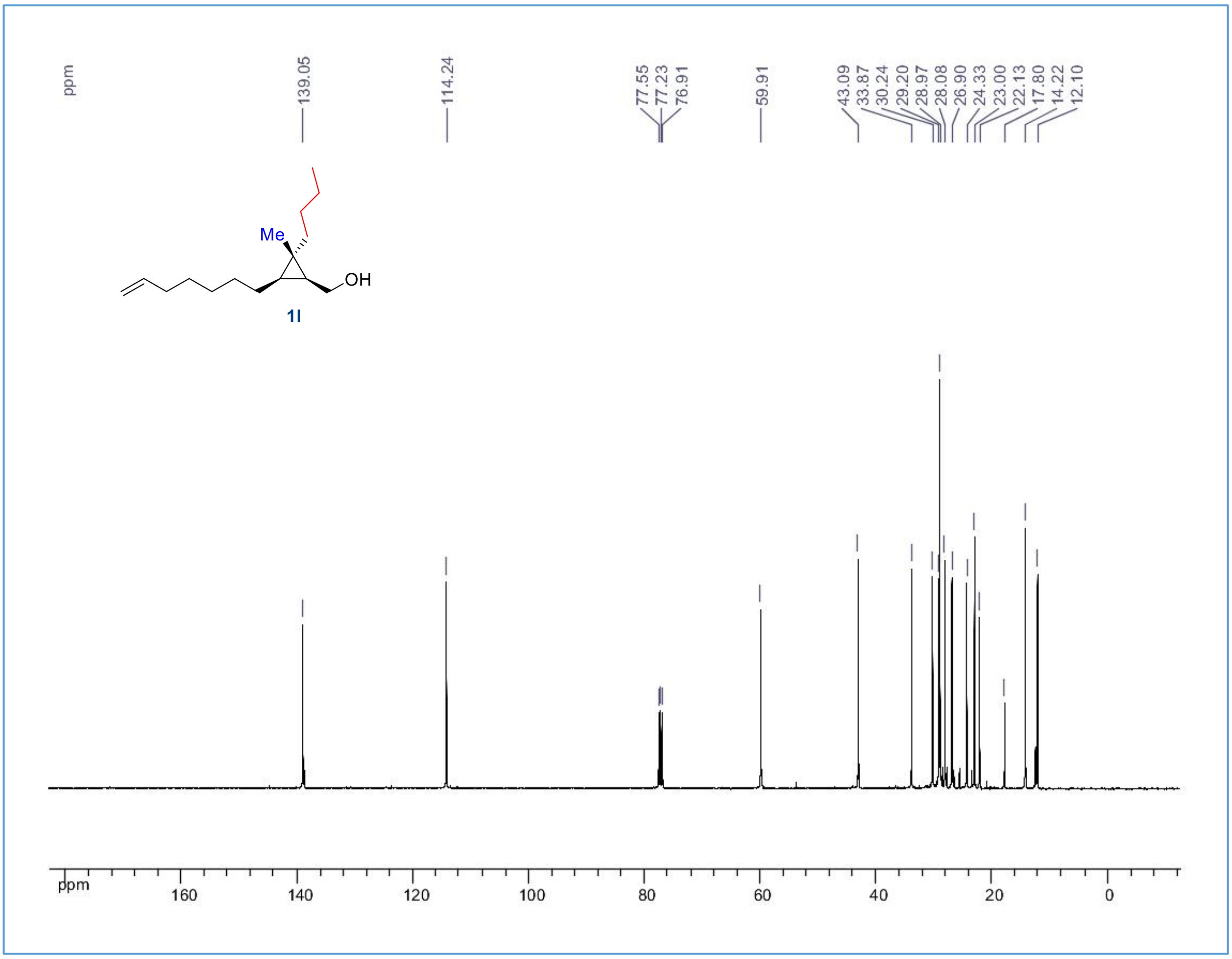

Supplementary Figure 16. ^{1}H and ^{13}C NMR spectra of compound **1l**

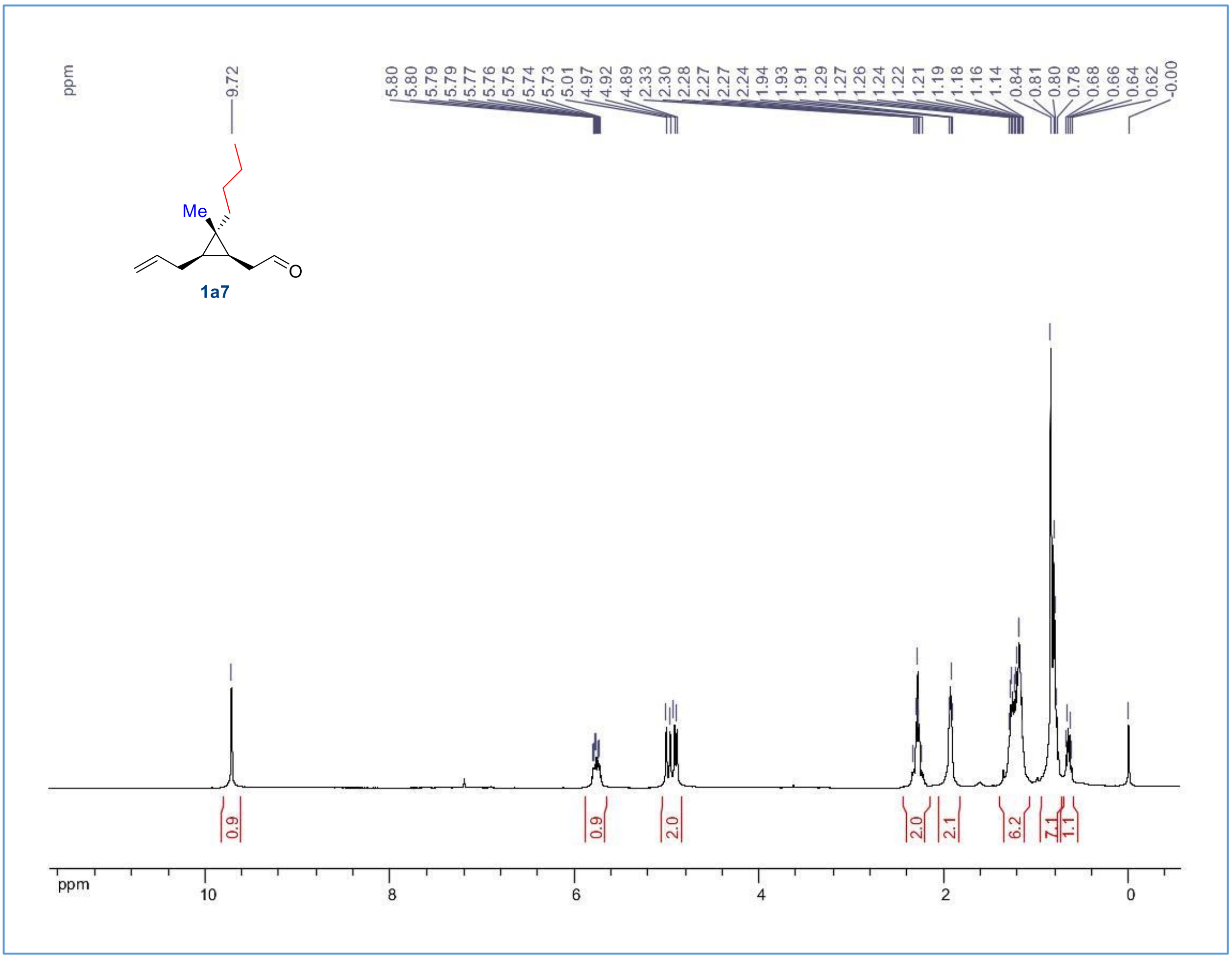

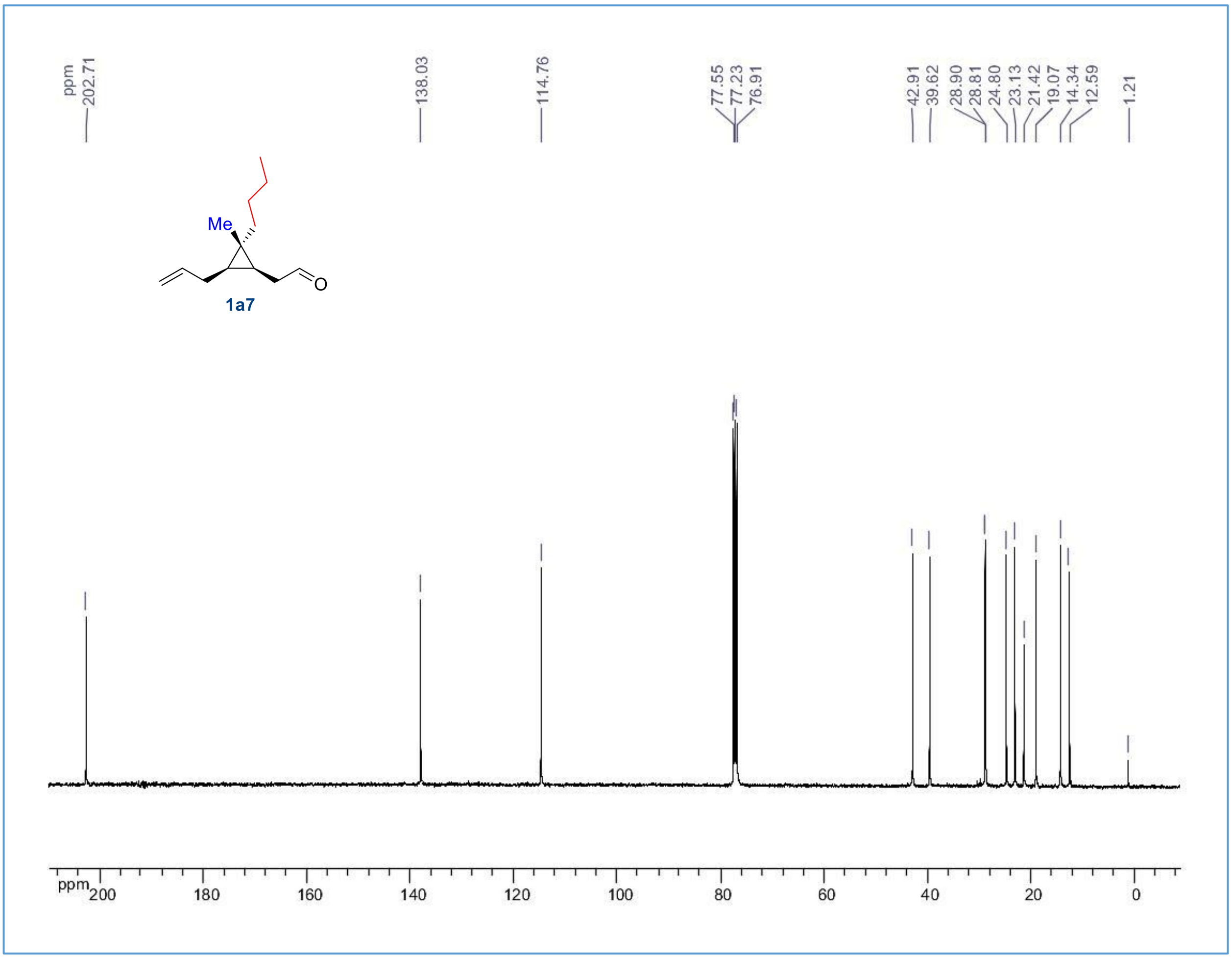

Supplementary Figure 17. ^{1}H and ^{13}C NMR spectra of compound **1a7**

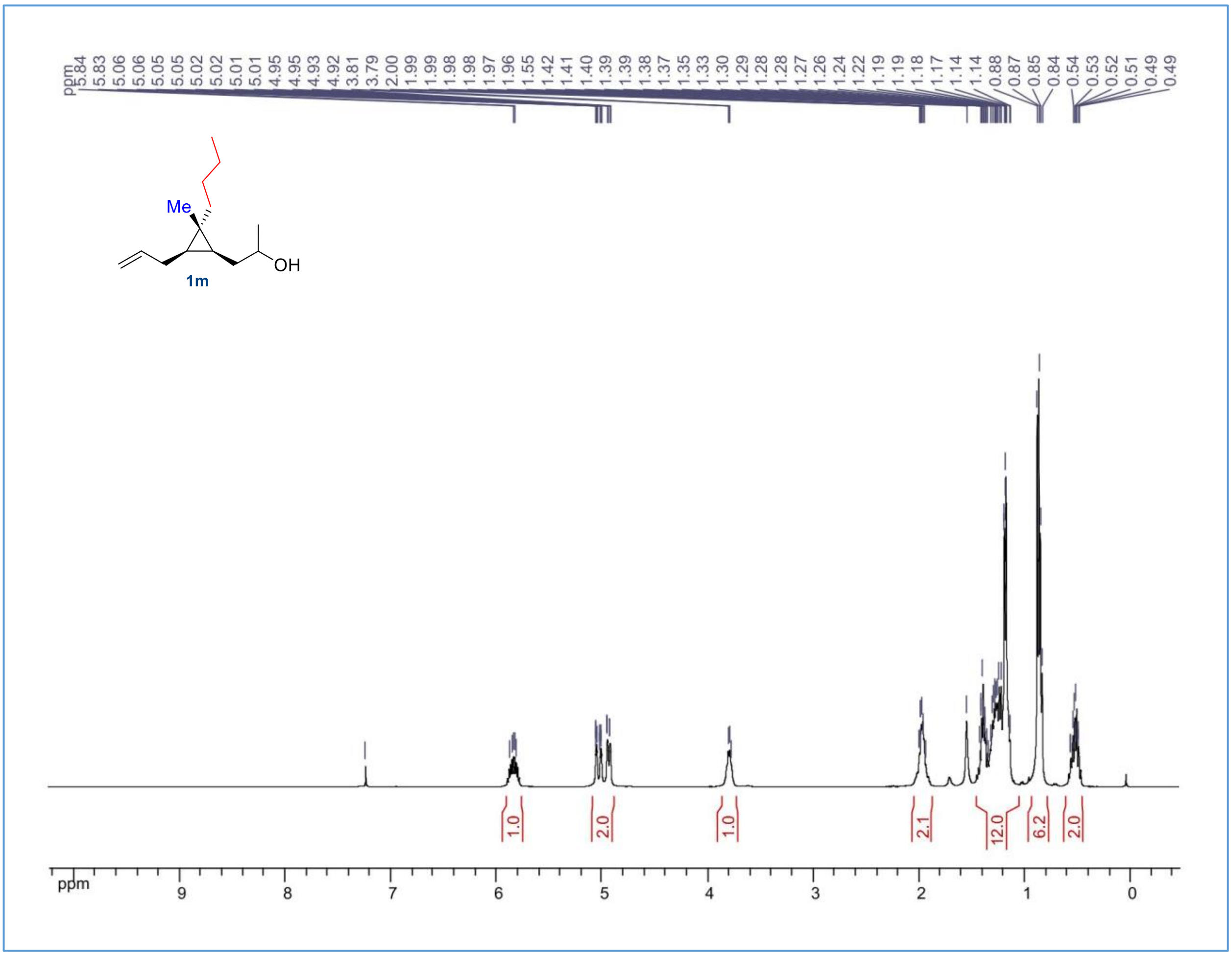

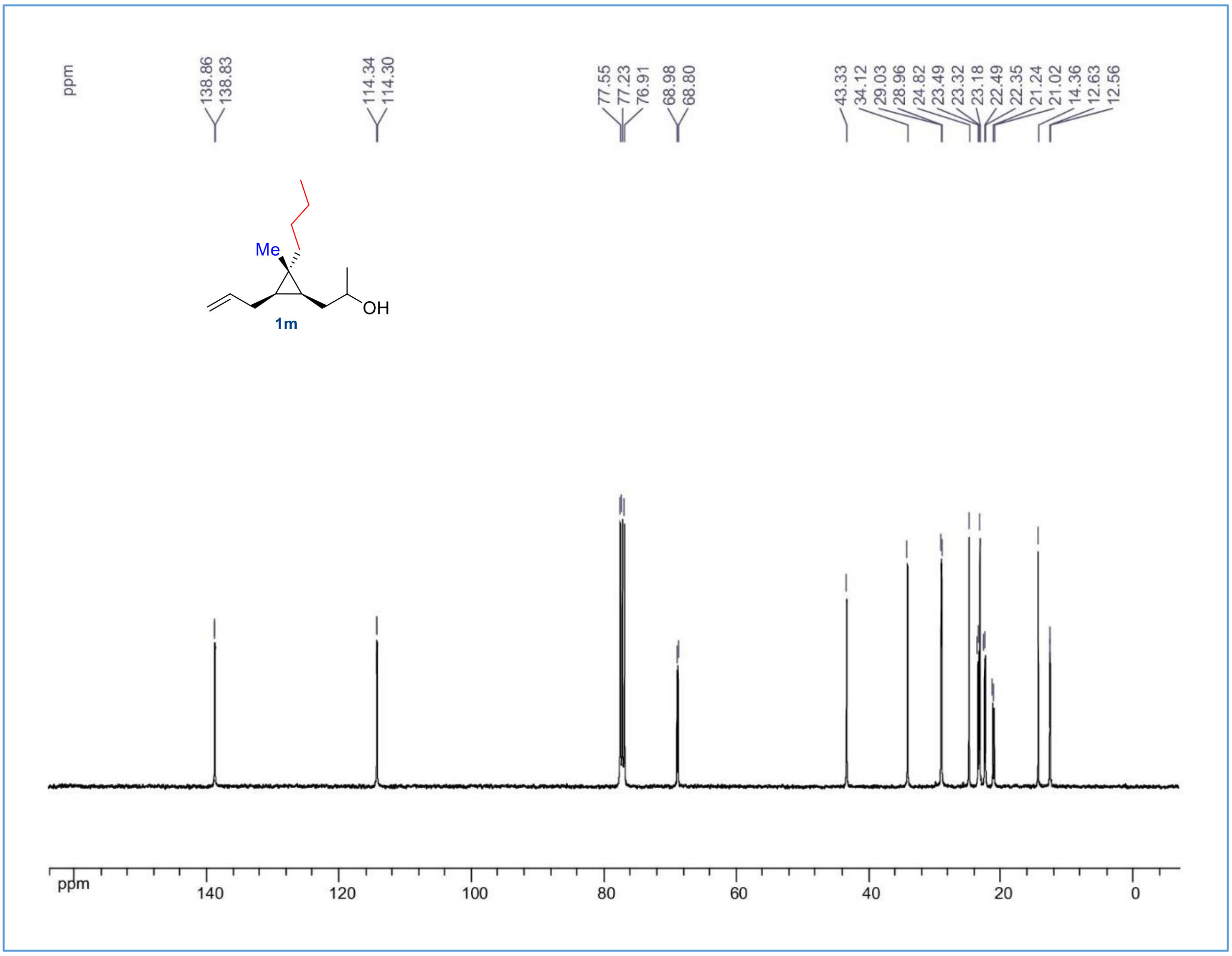

Supplementary Figure 18. ^{1}H and ^{13}C NMR spectra of compound **1m**

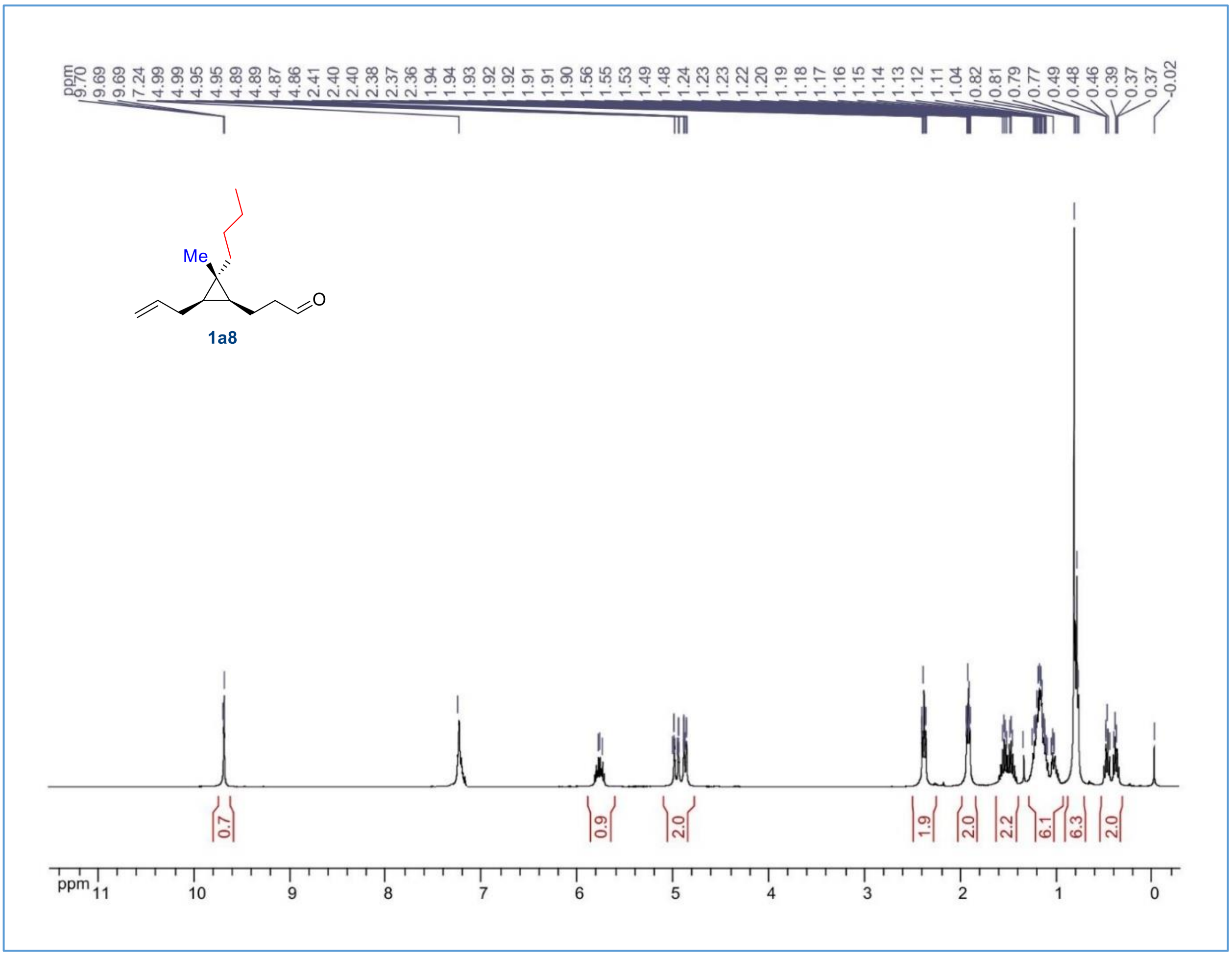

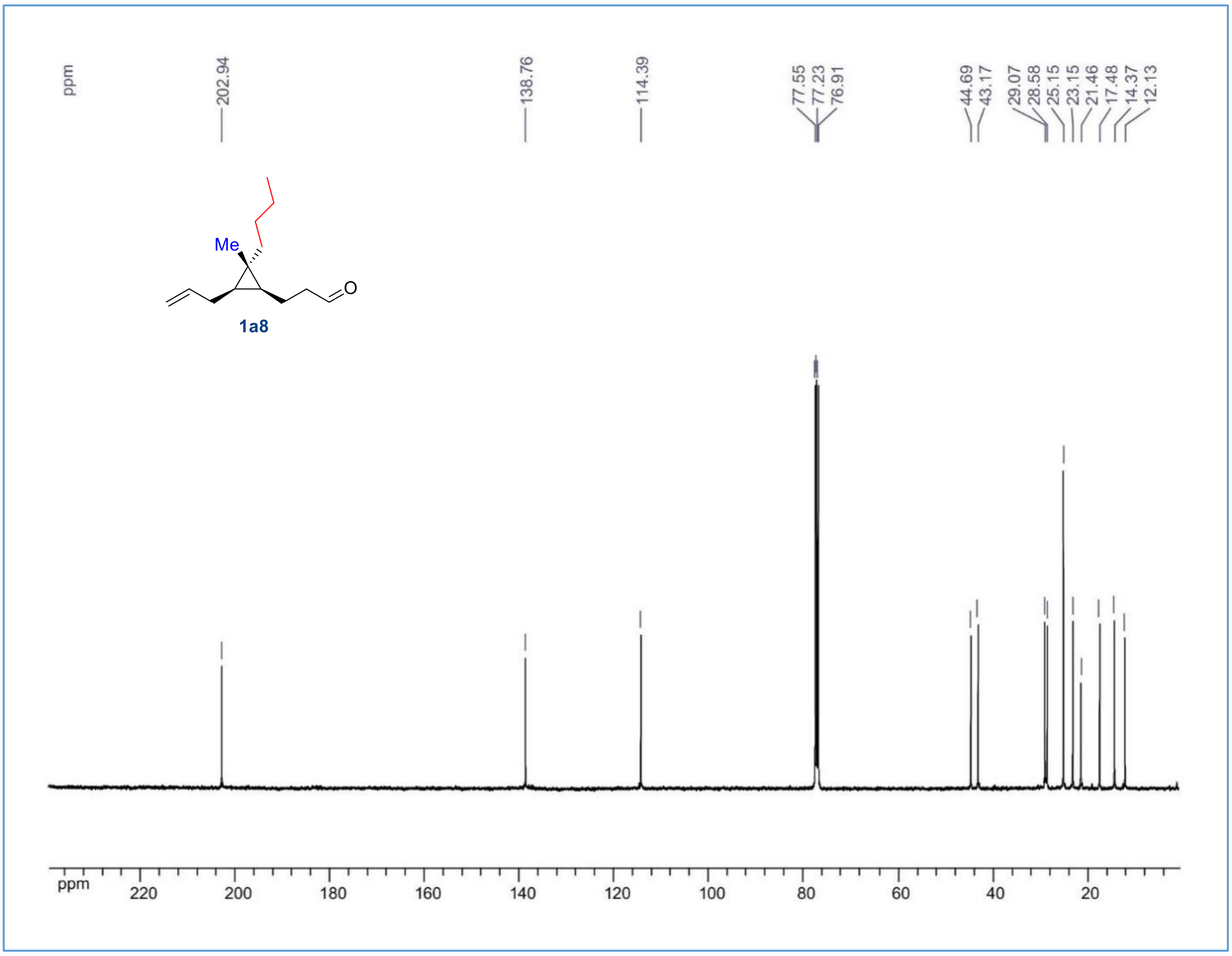

Supplementary Figure 19. ^{1}H and ^{13}C NMR spectra of compound **1a8**

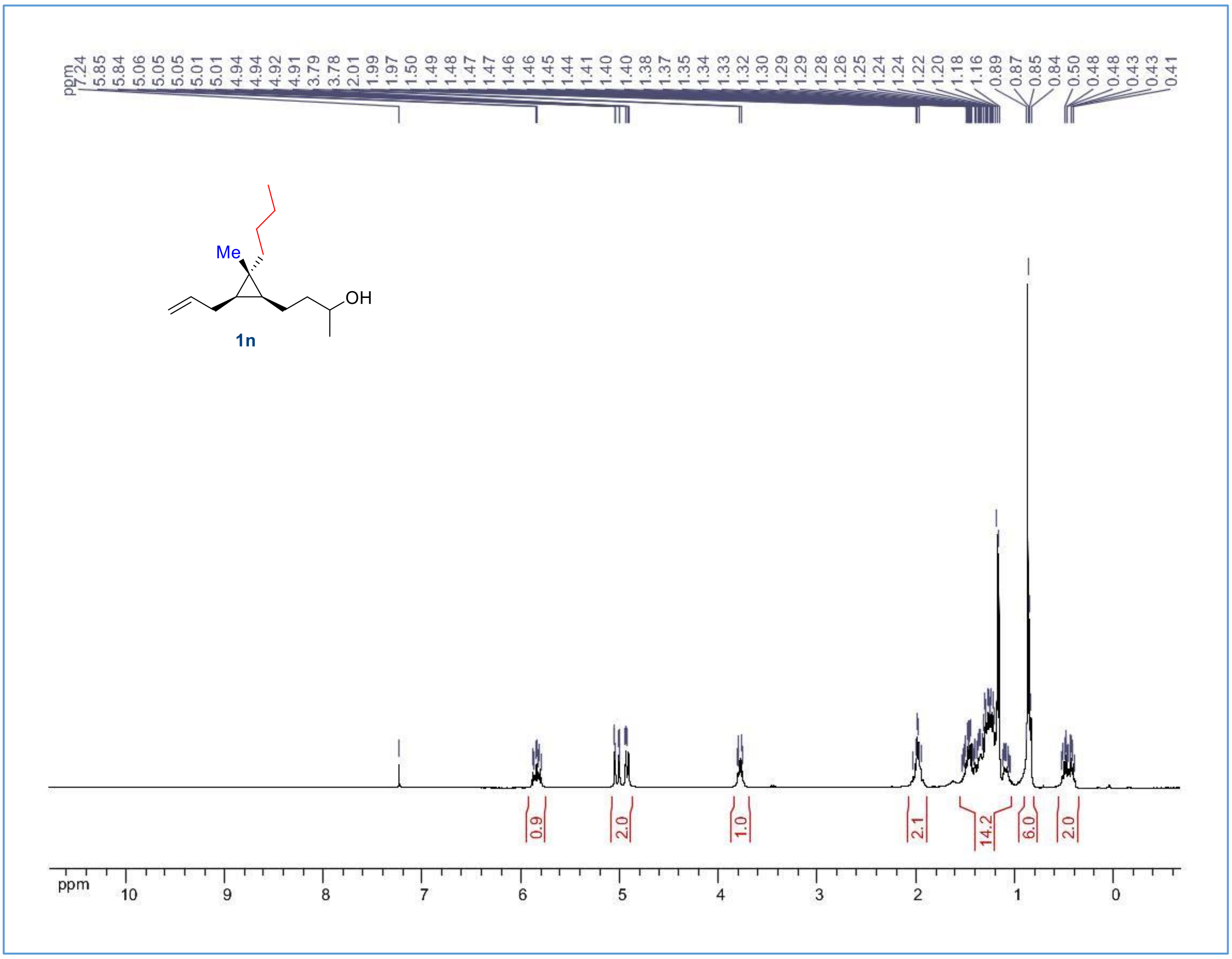

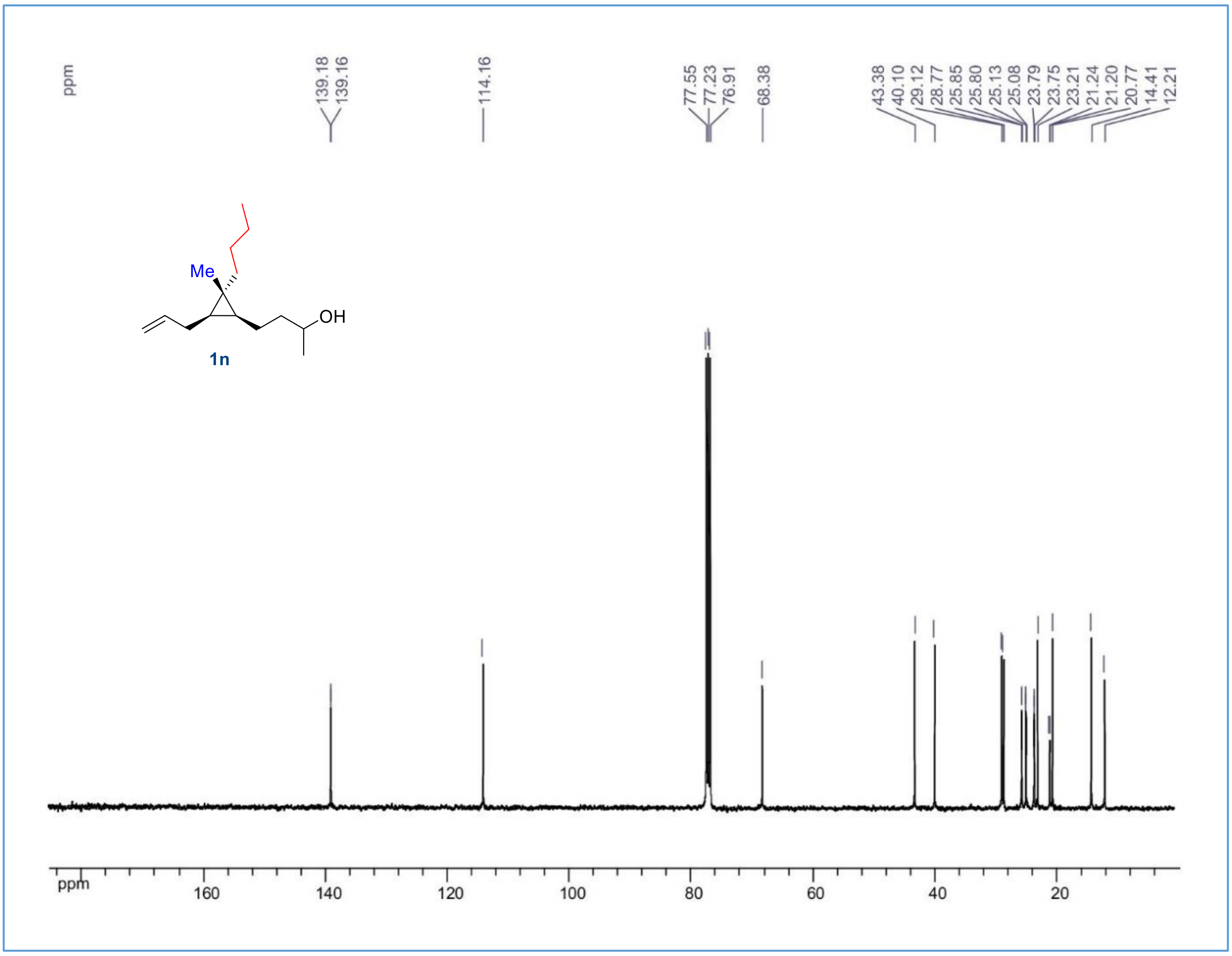

Supplementary Figure 20. ^{1}H and ^{13}C NMR spectra of compound **1n**

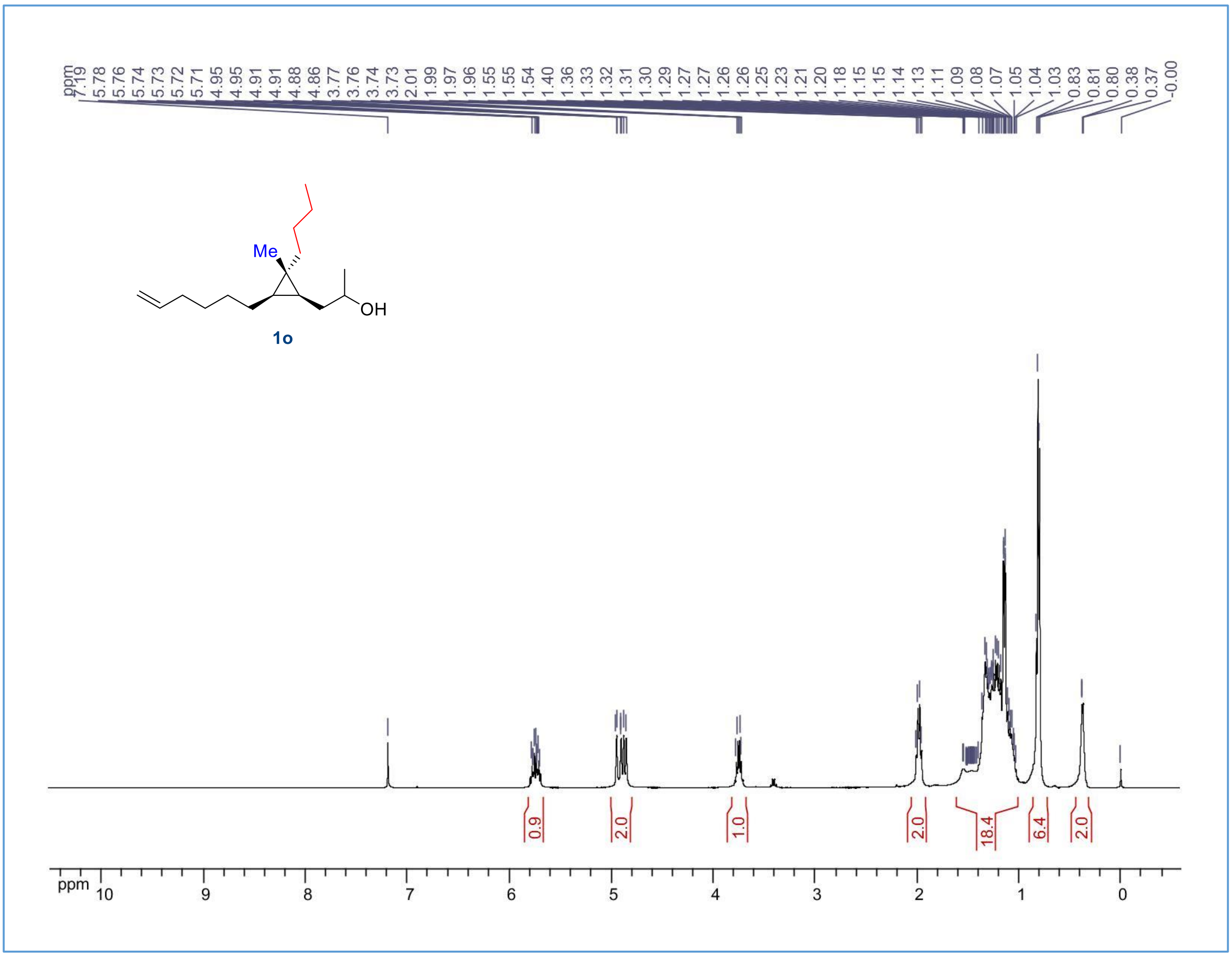

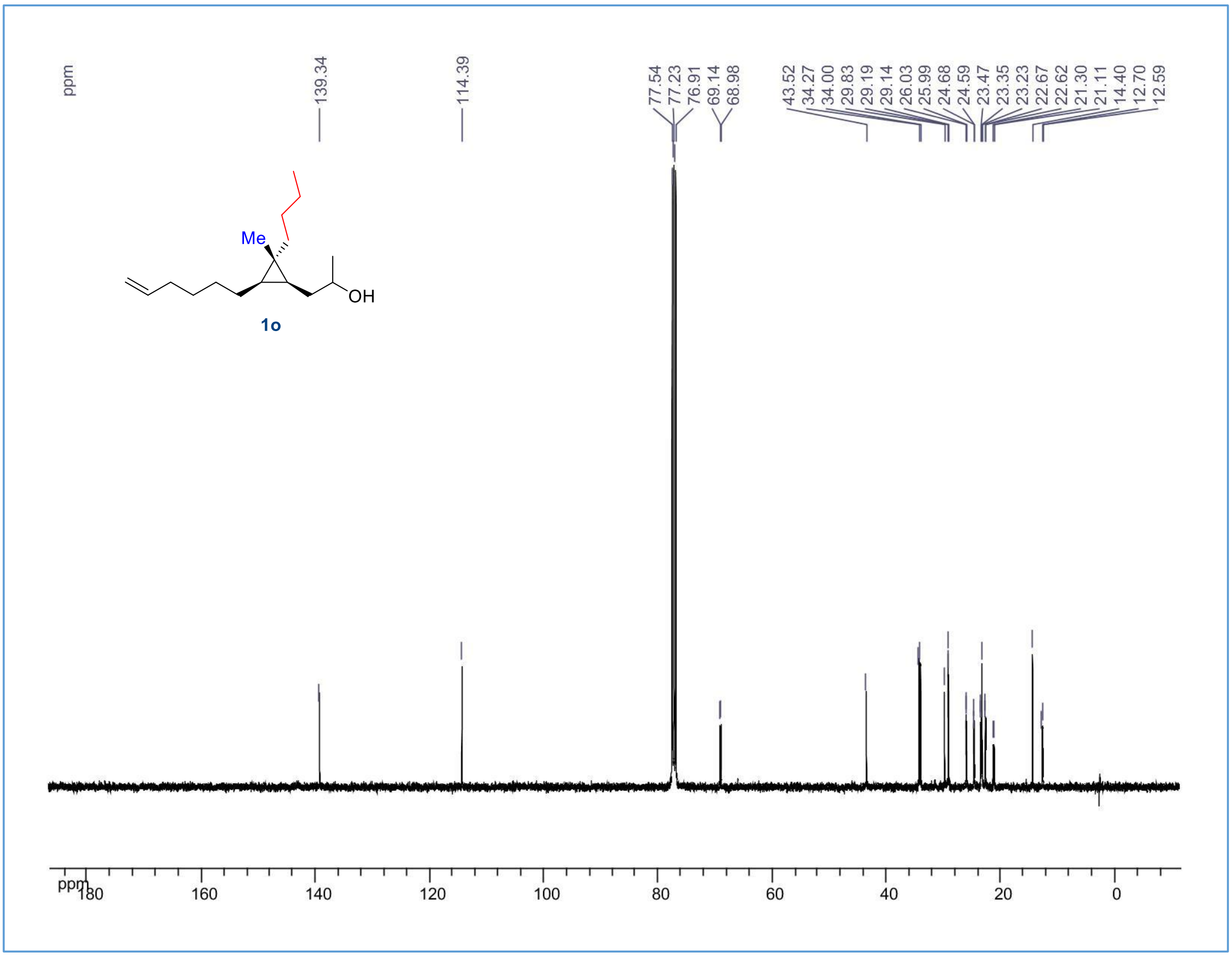

Supplementary Figure 21. ^{1}H and ^{13}C NMR spectra of compound **1o**

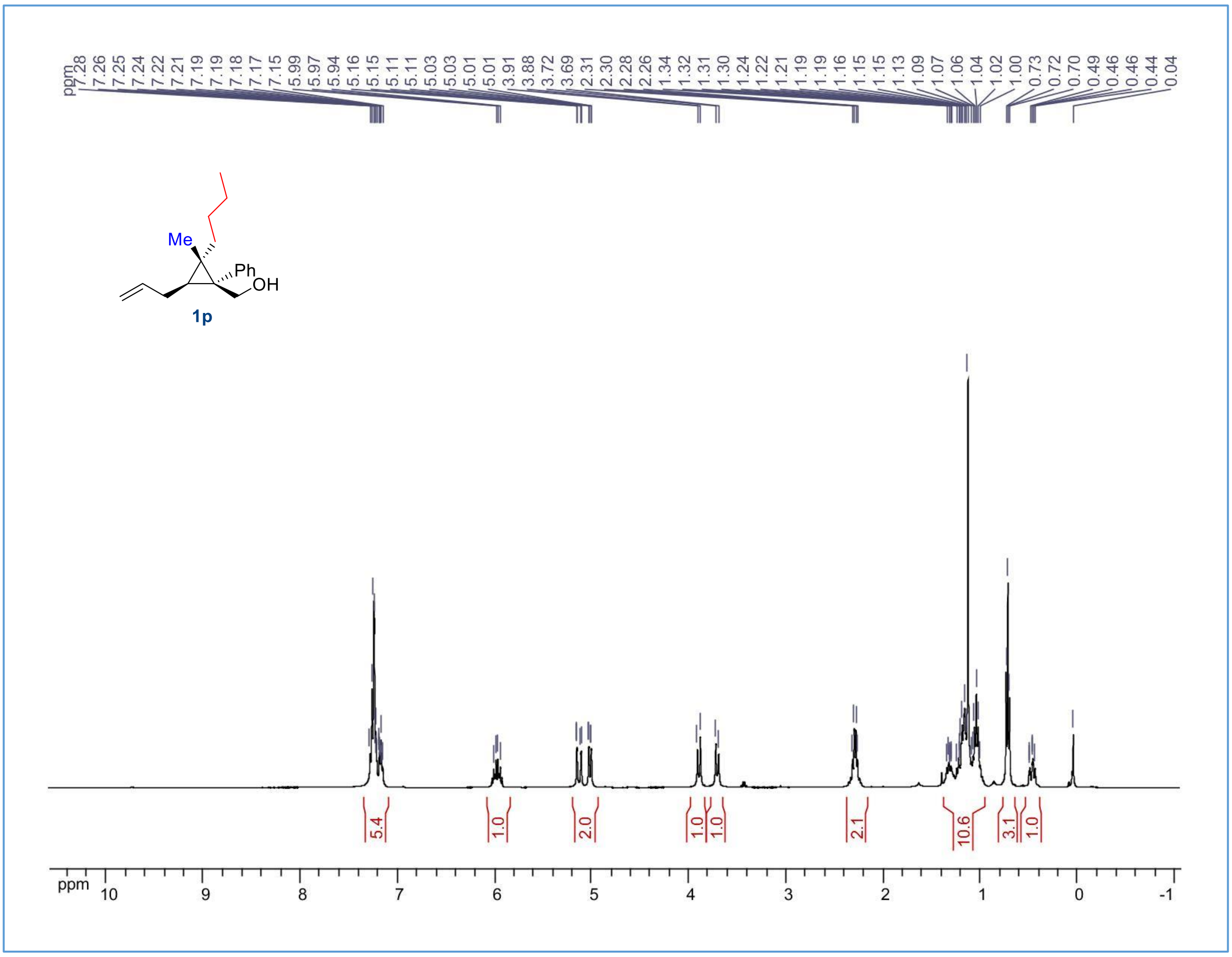

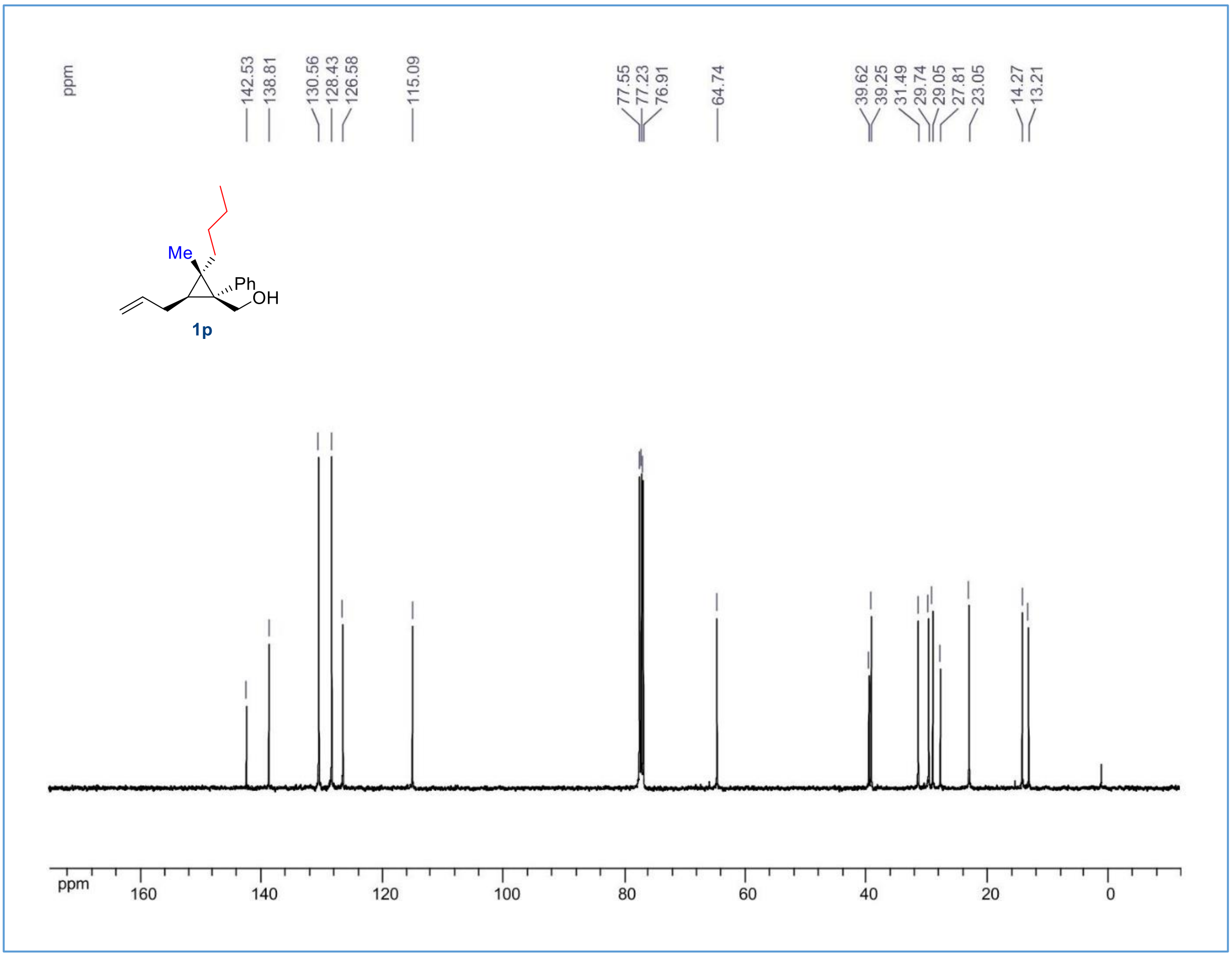

Supplementary Figure 22. ^{1}H and ^{13}C NMR spectra of compound **1p**

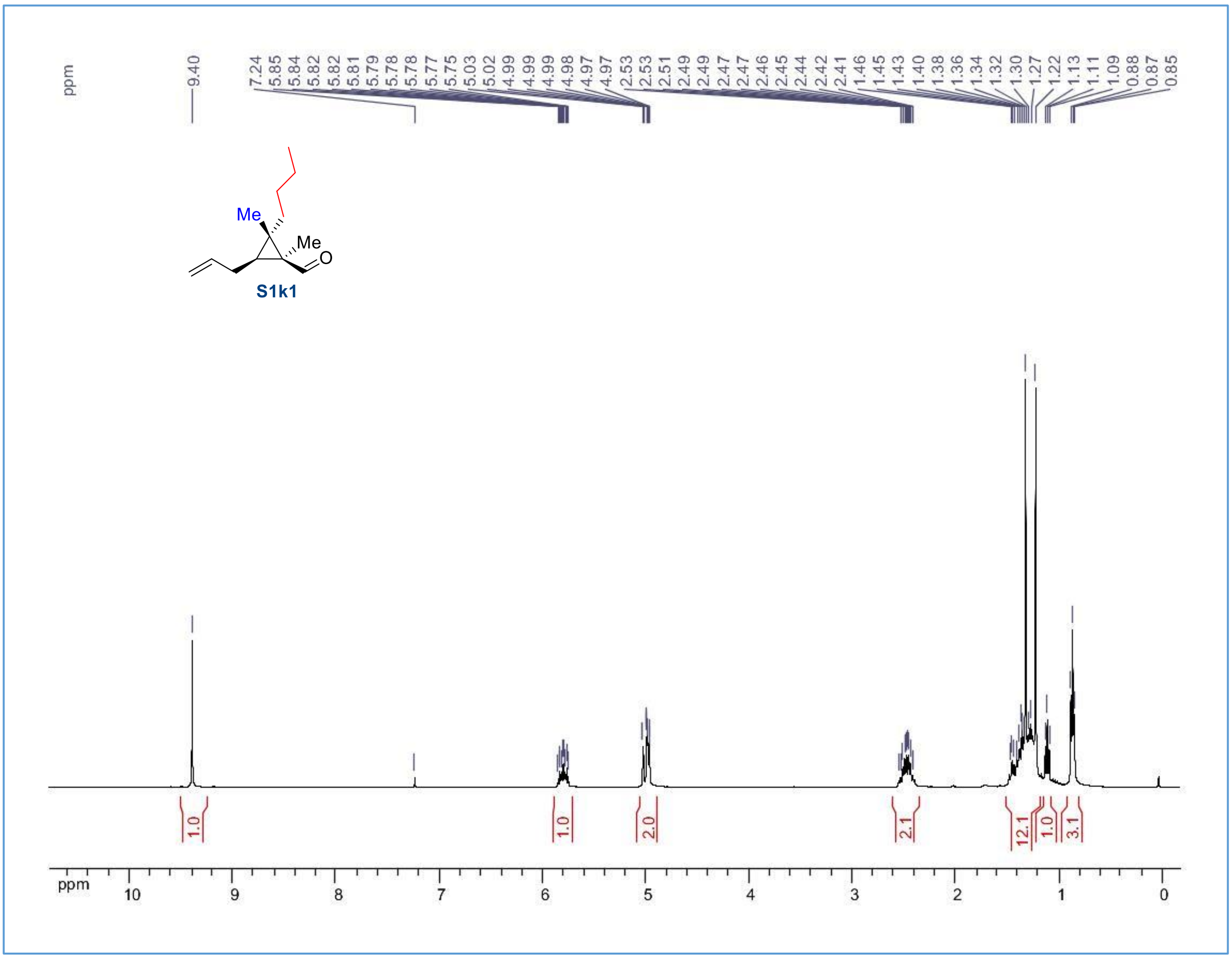

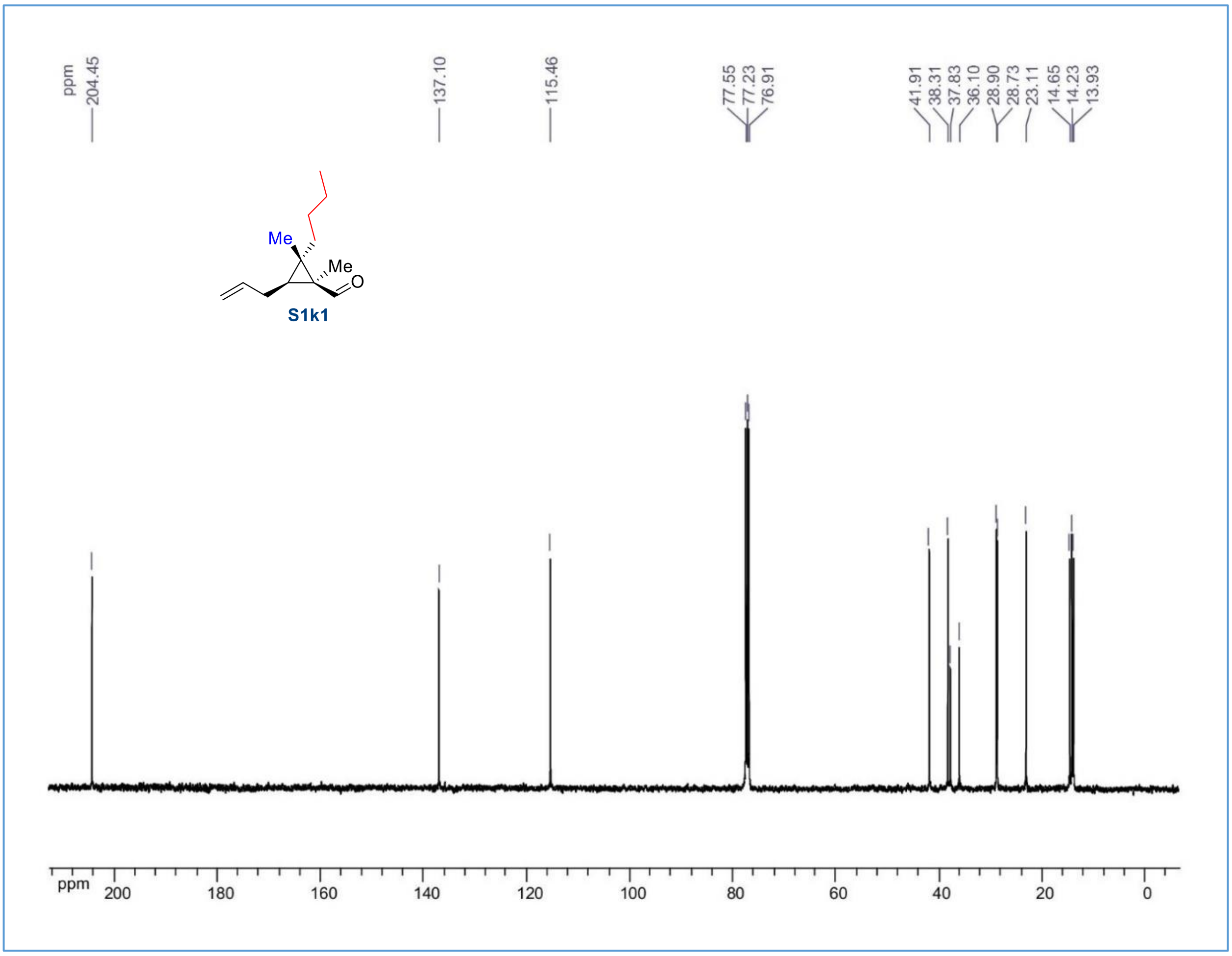

Supplementary Figure 23. ^{1}H and ^{13}C NMR spectra of compound **S1k1**

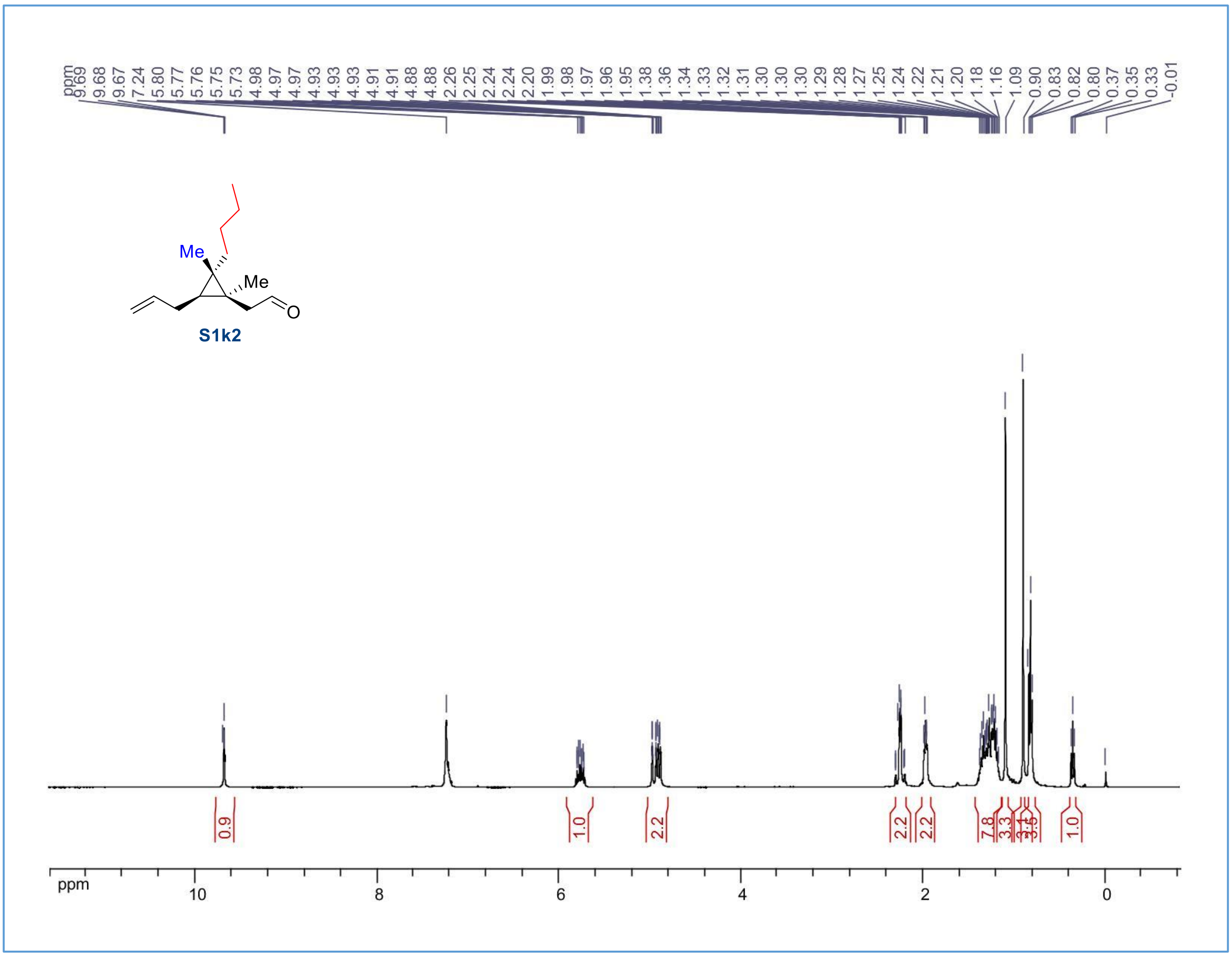

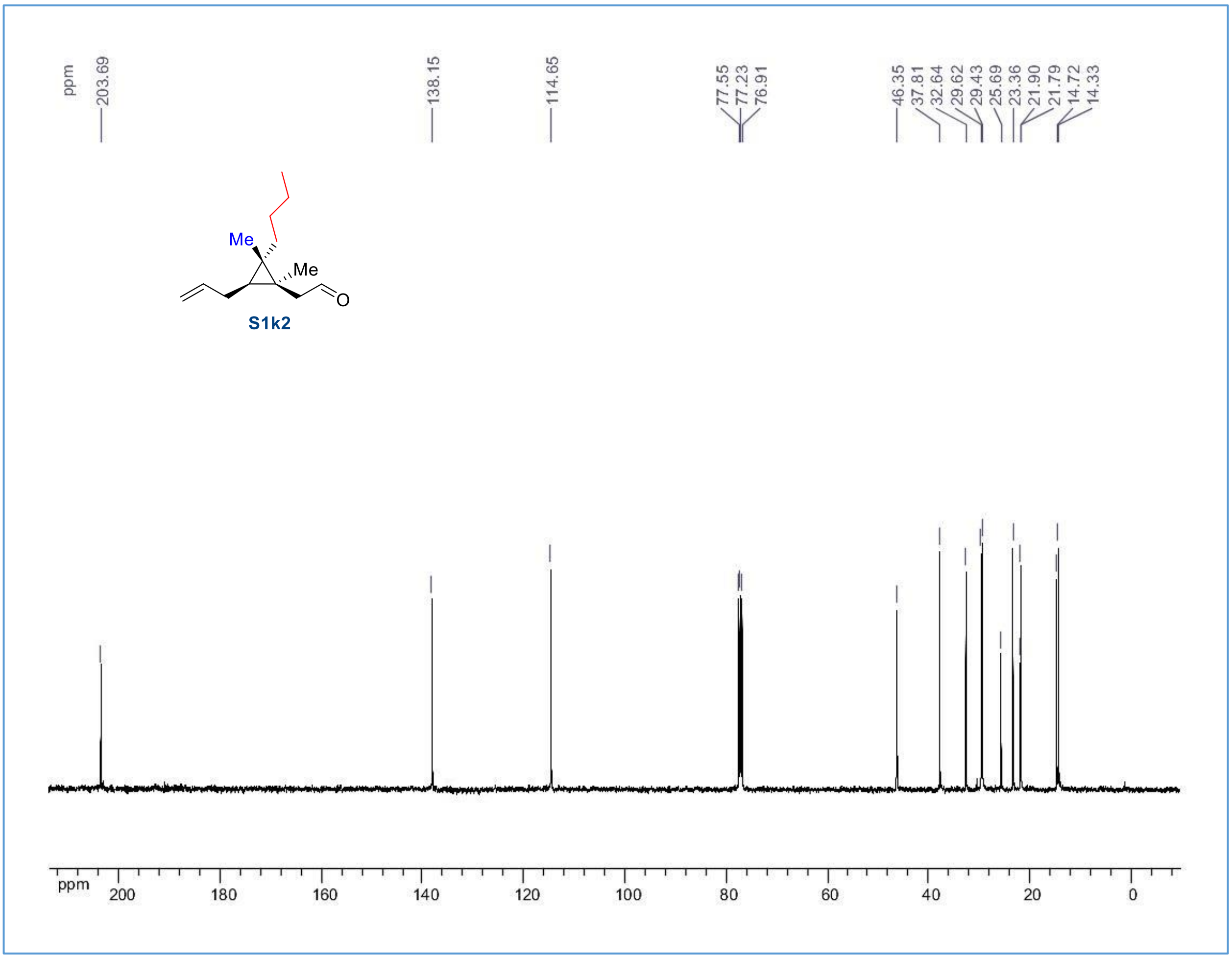

Supplementary Figure 24. ^{1}H and ^{13}C NMR spectra of compound **S1k2**

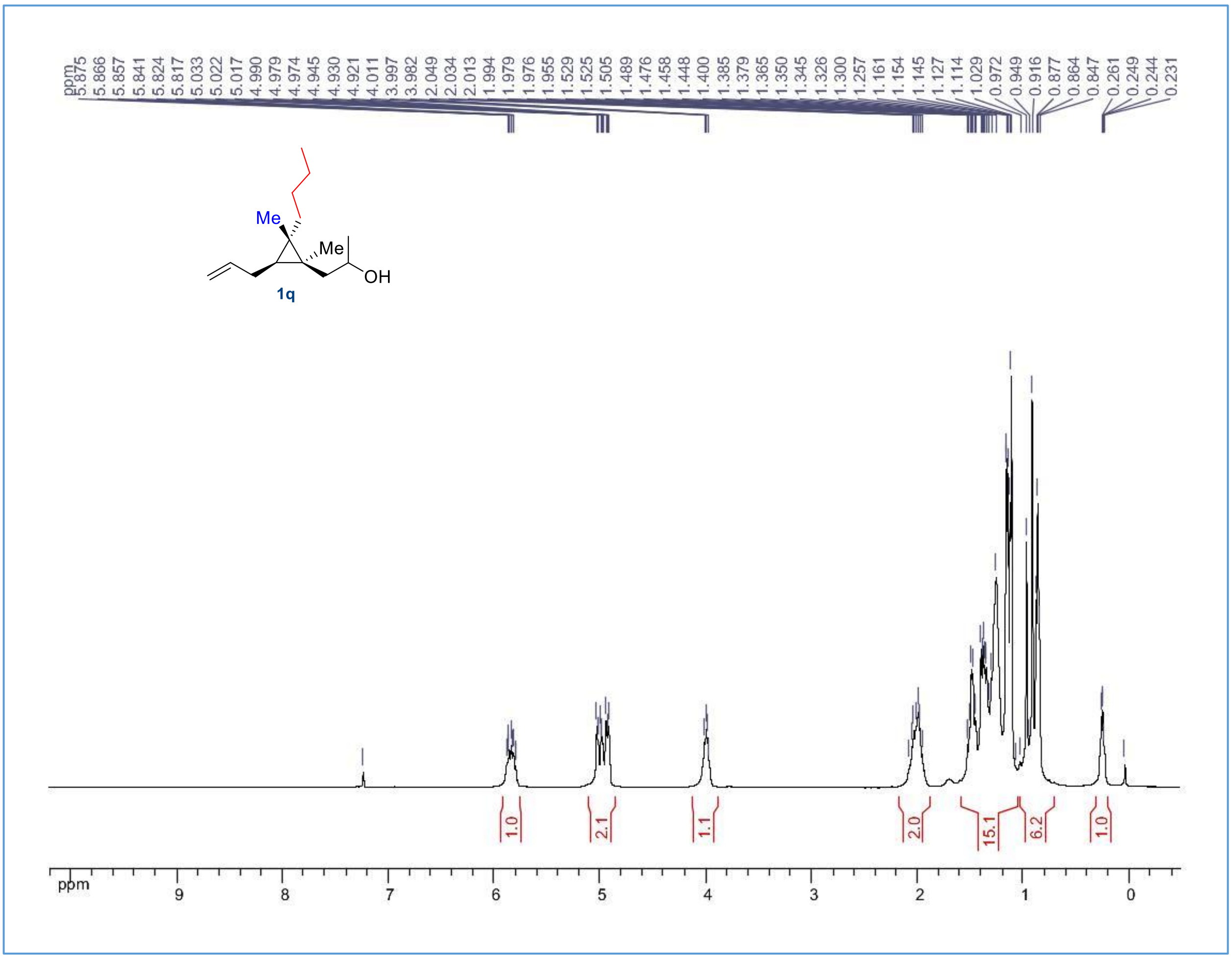

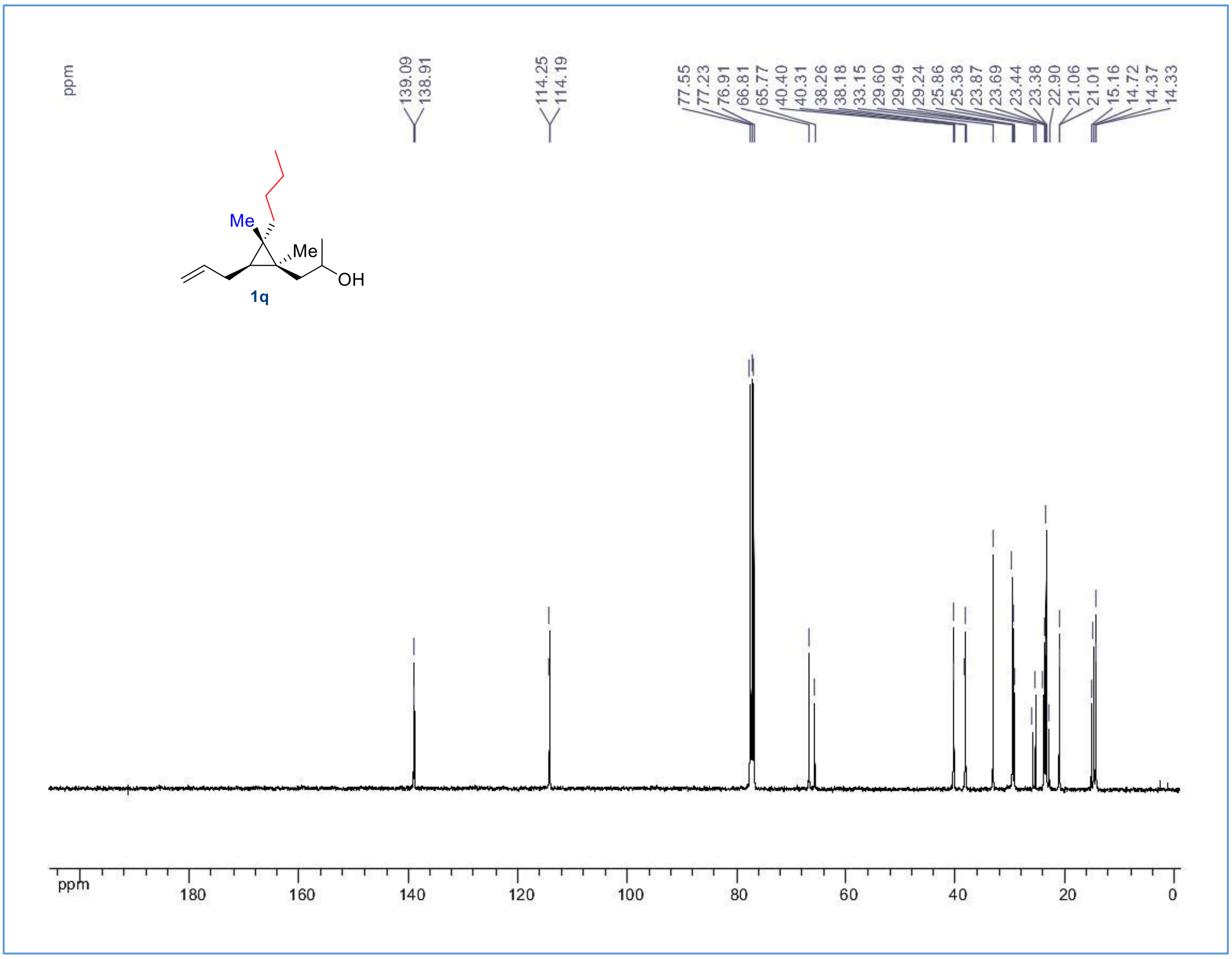

Supplementary Figure 25. ^{1}H and ^{13}C NMR spectra of compound **1q**

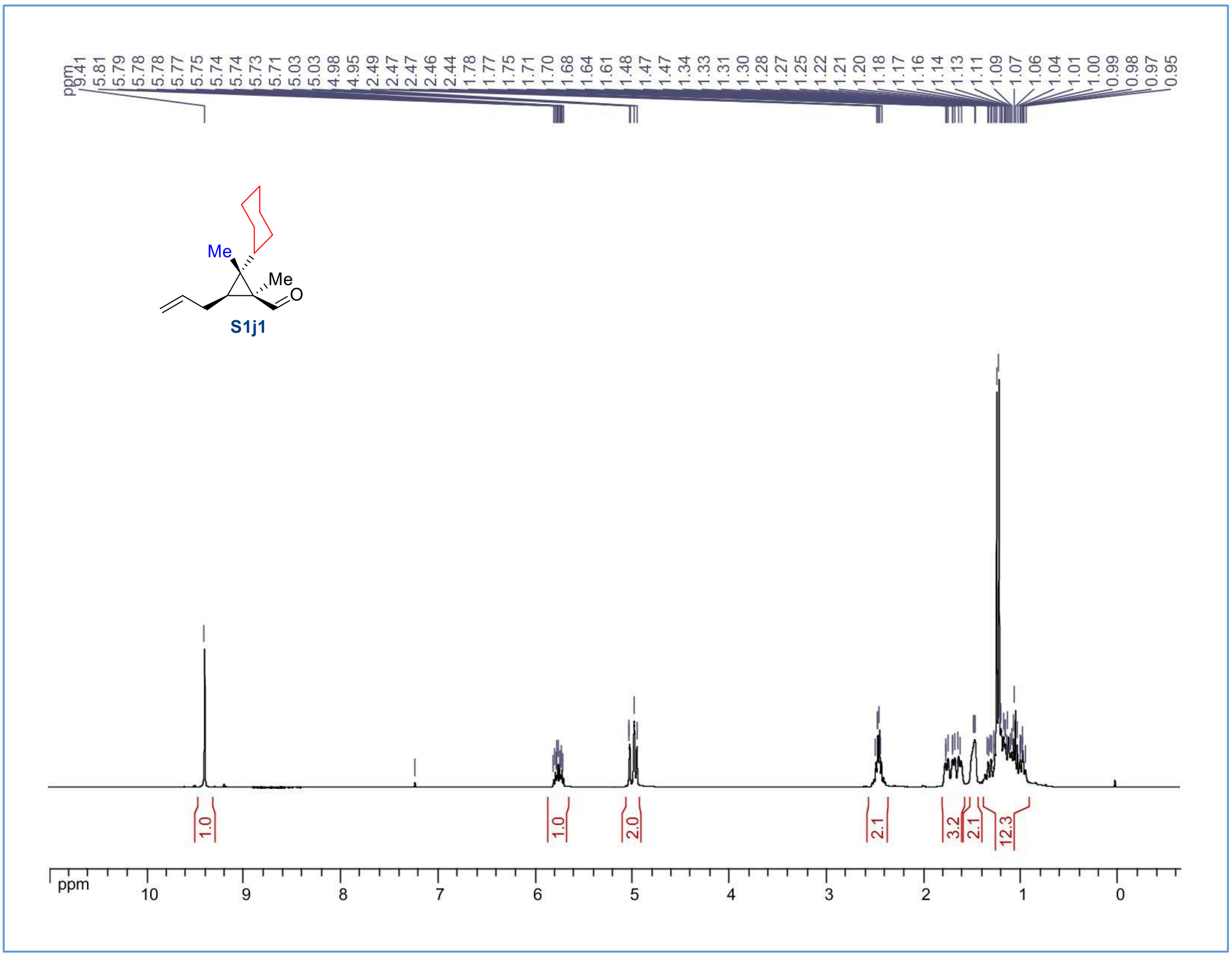

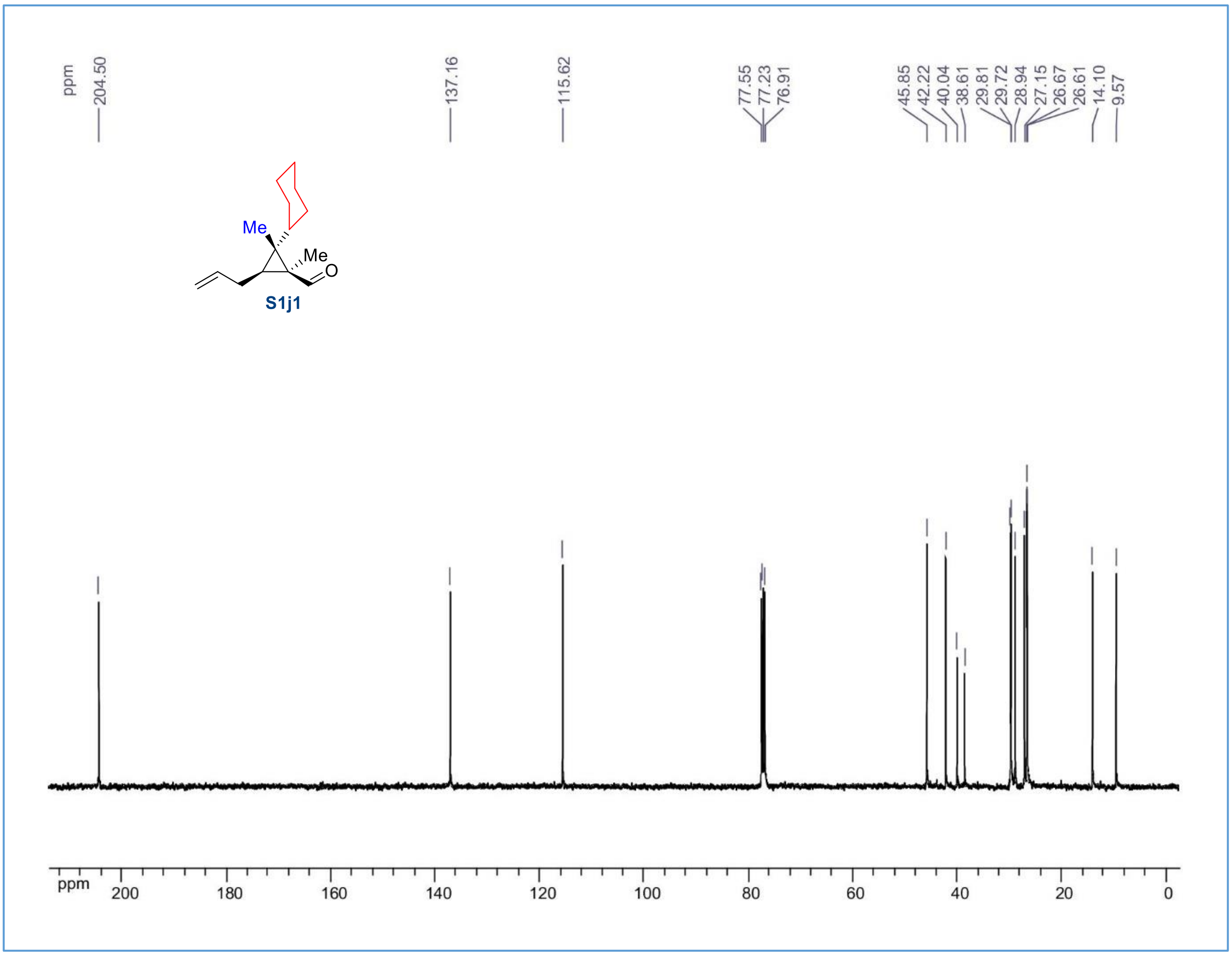

Supplementary Figure 26. ^{1}H and ^{13}C NMR spectra of compound **S1j1**

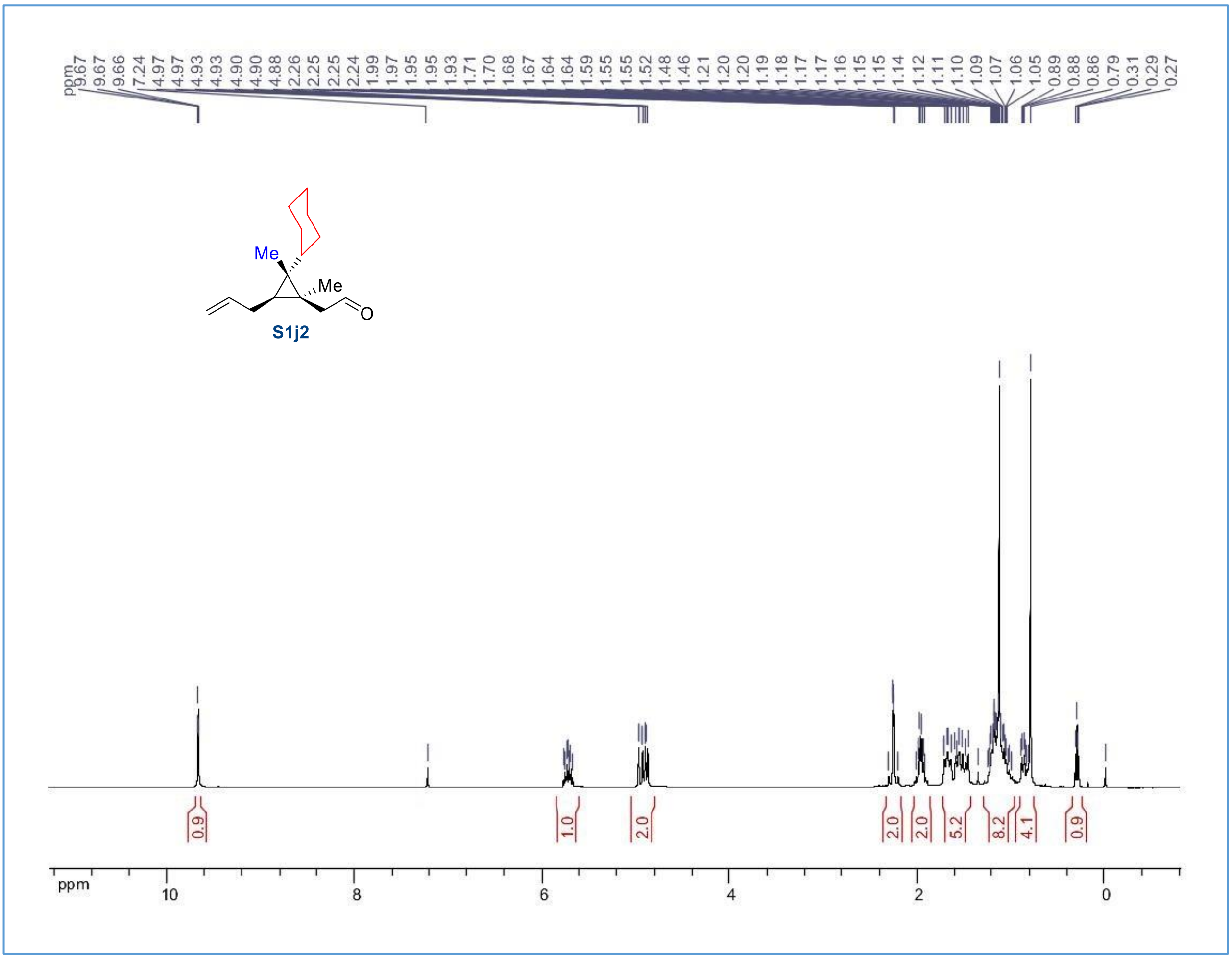

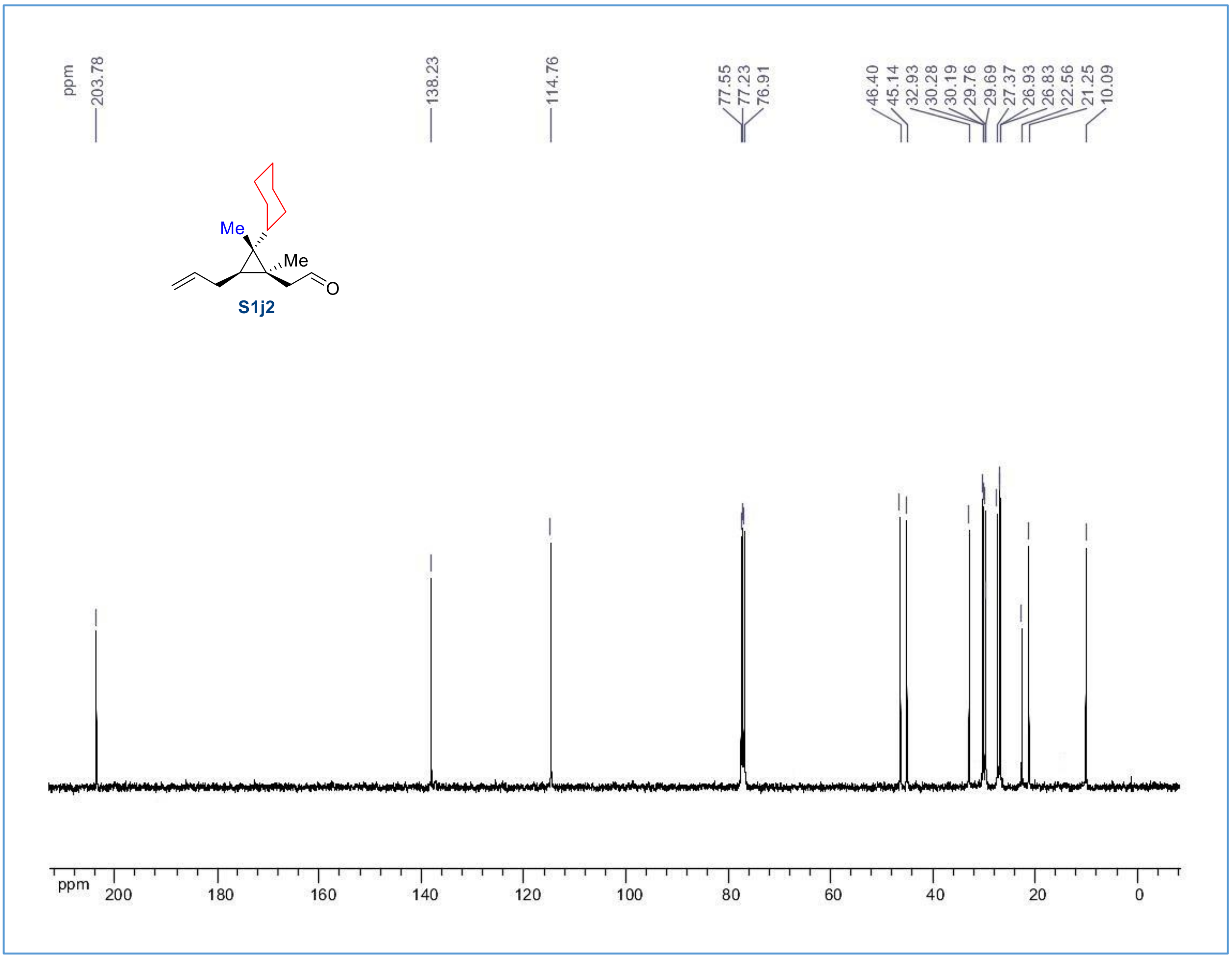

Supplementary Figure 27. ^{1}H and ^{13}C NMR spectra of compound **S1j2**

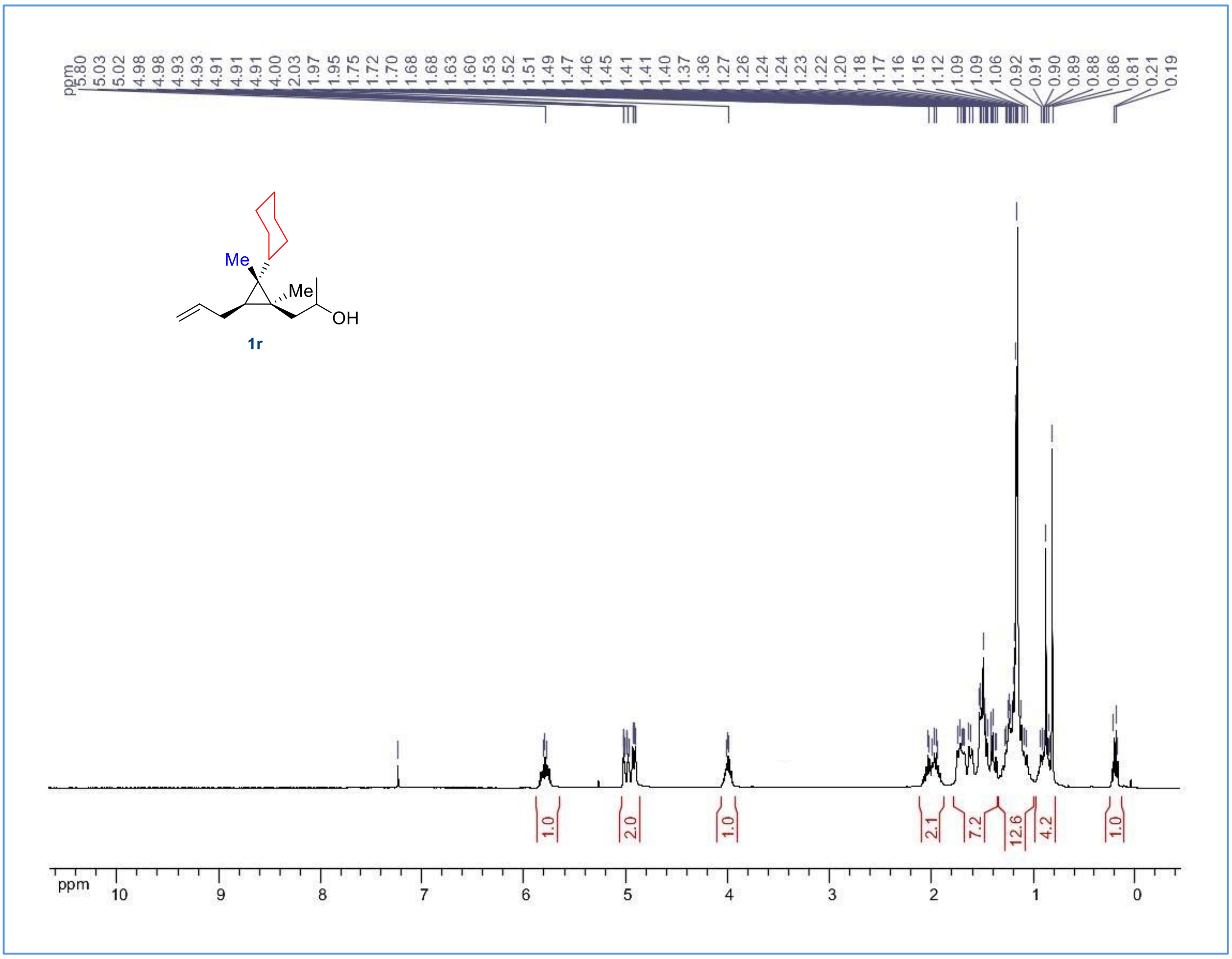

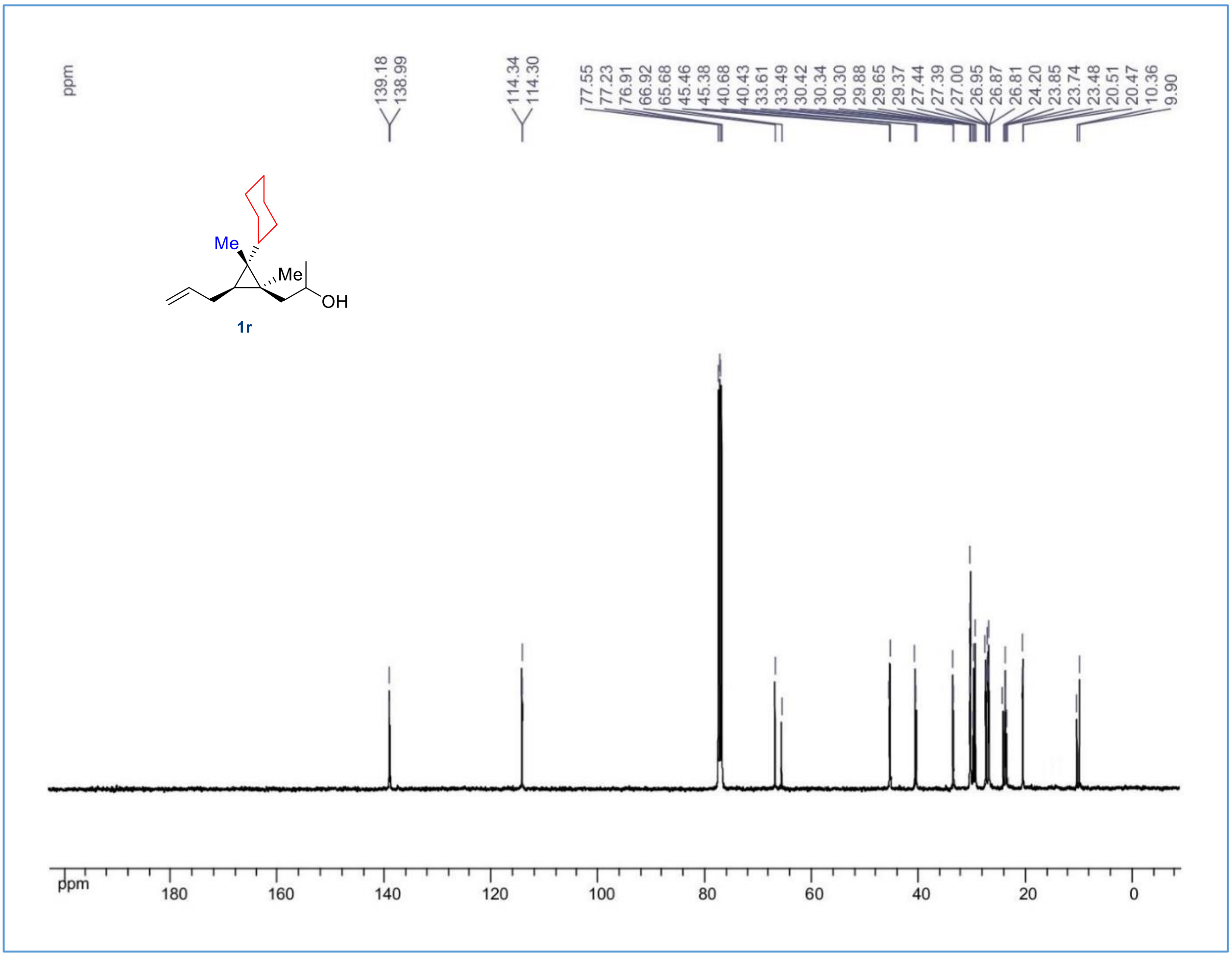

Supplementary Figure 28. ^{1}H and ^{13}C NMR spectra of compound **1r**

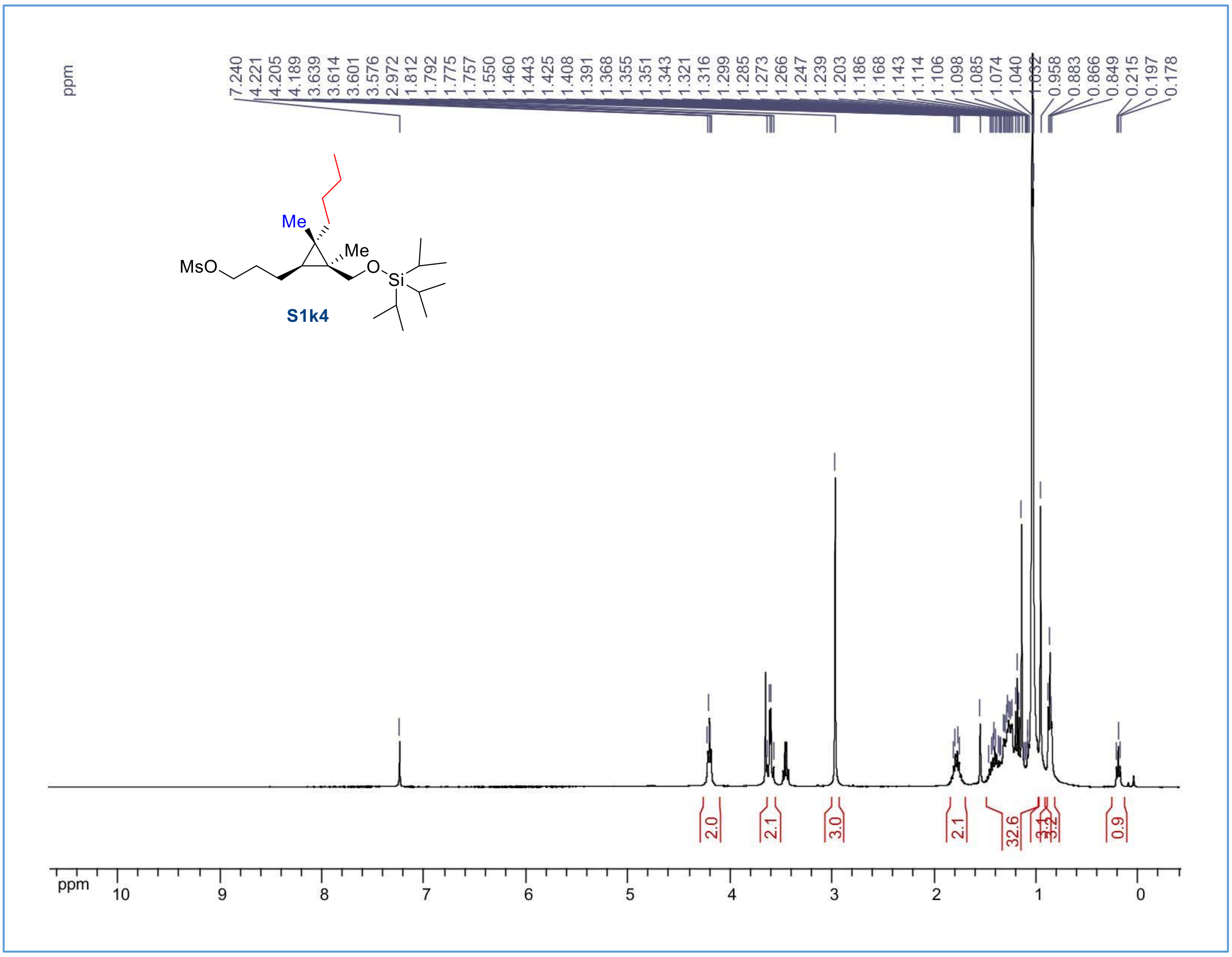

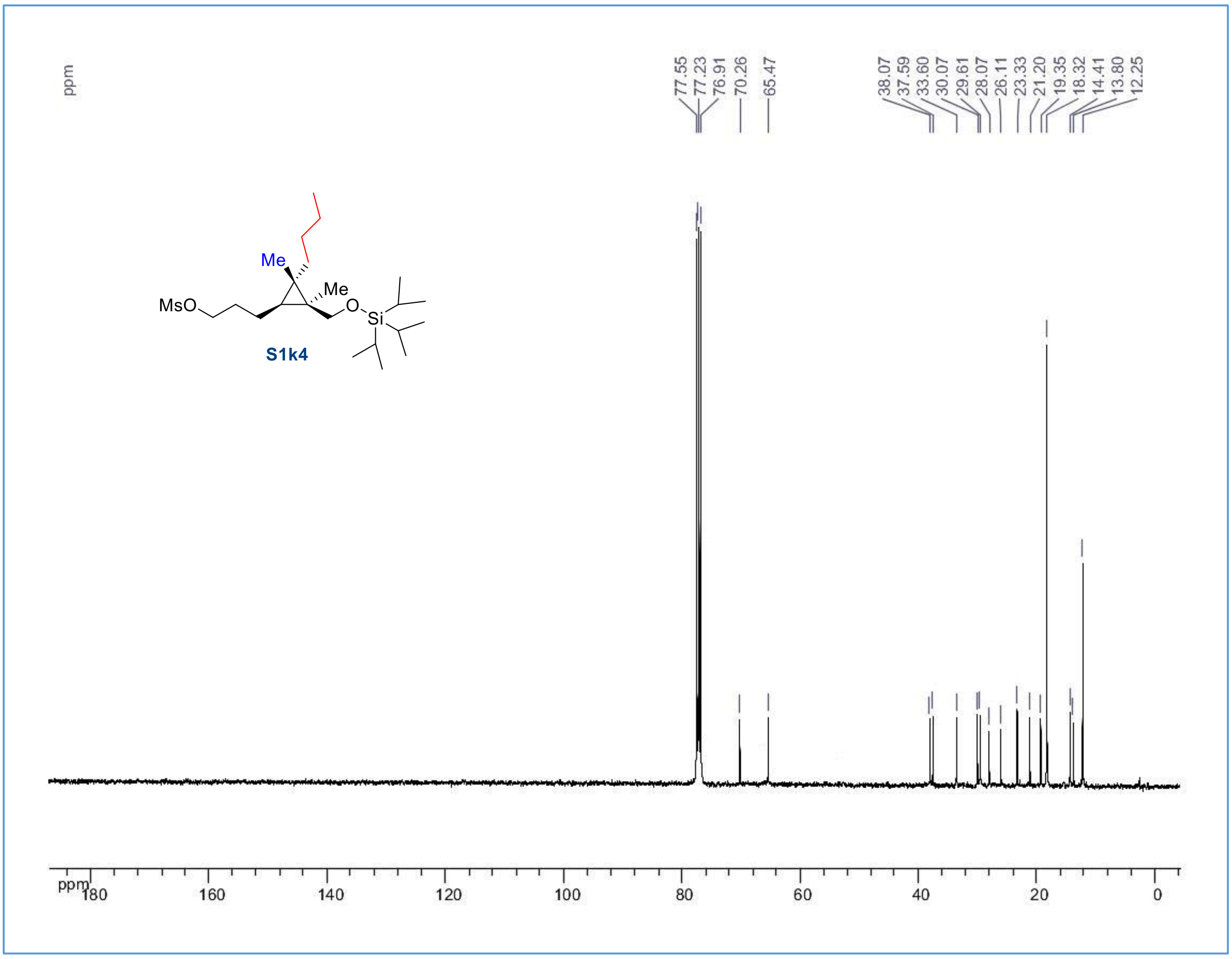

Supplementary Figure 29. ^{1}H and ^{13}C NMR spectra of compound **S1k4**

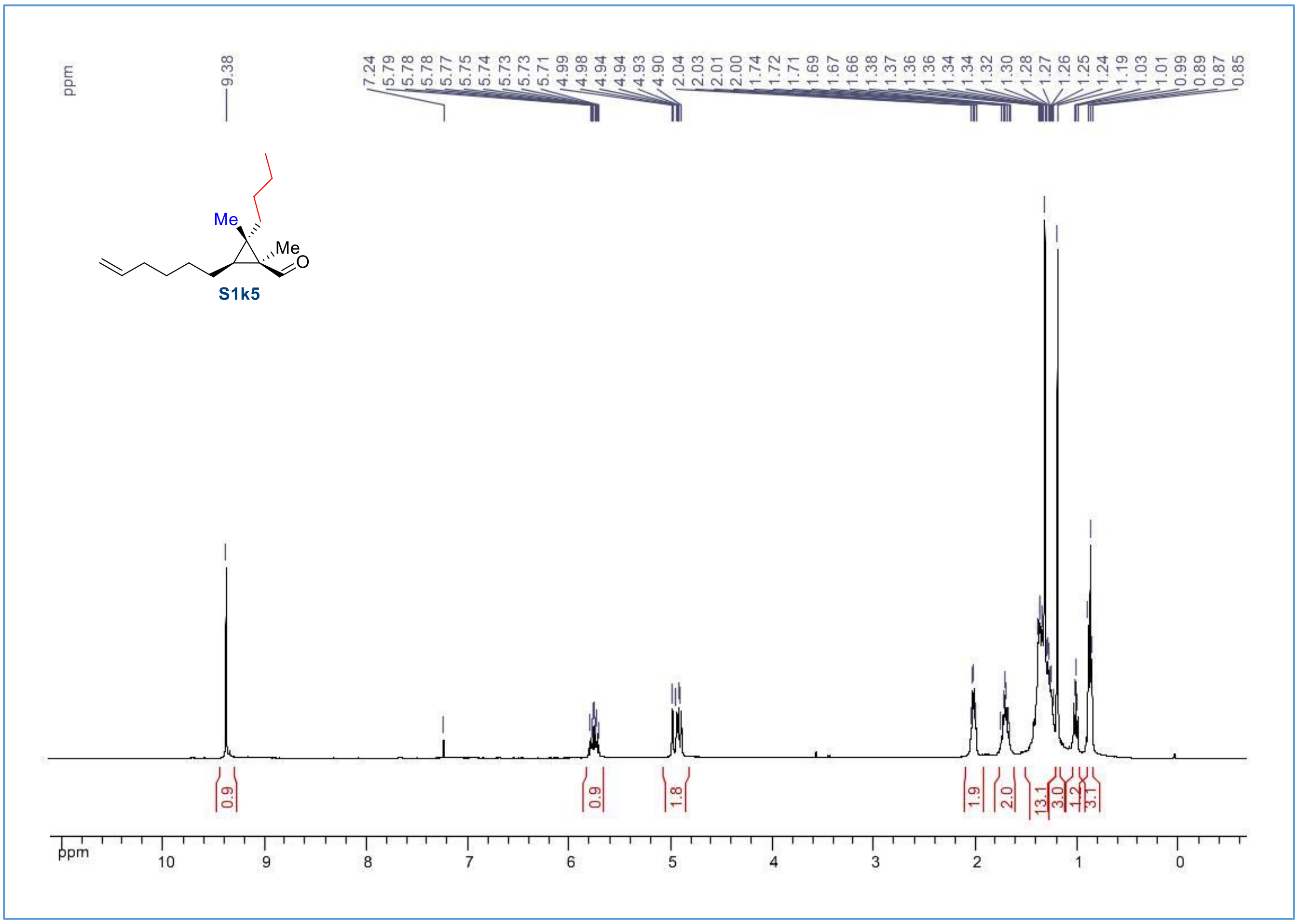

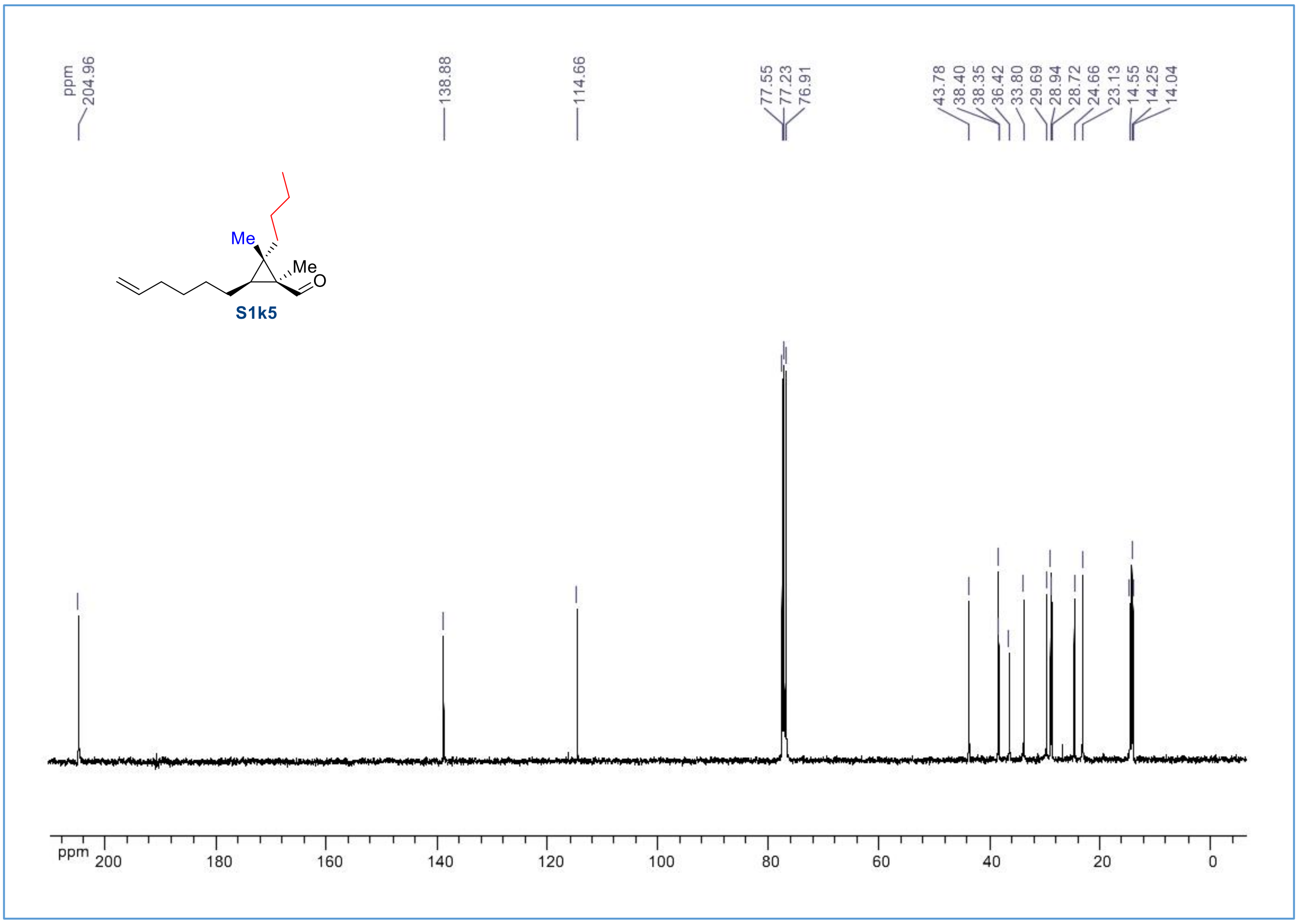

Supplementary Figure 30. ^{1}H and ^{13}C NMR spectra of compound **S1k5**

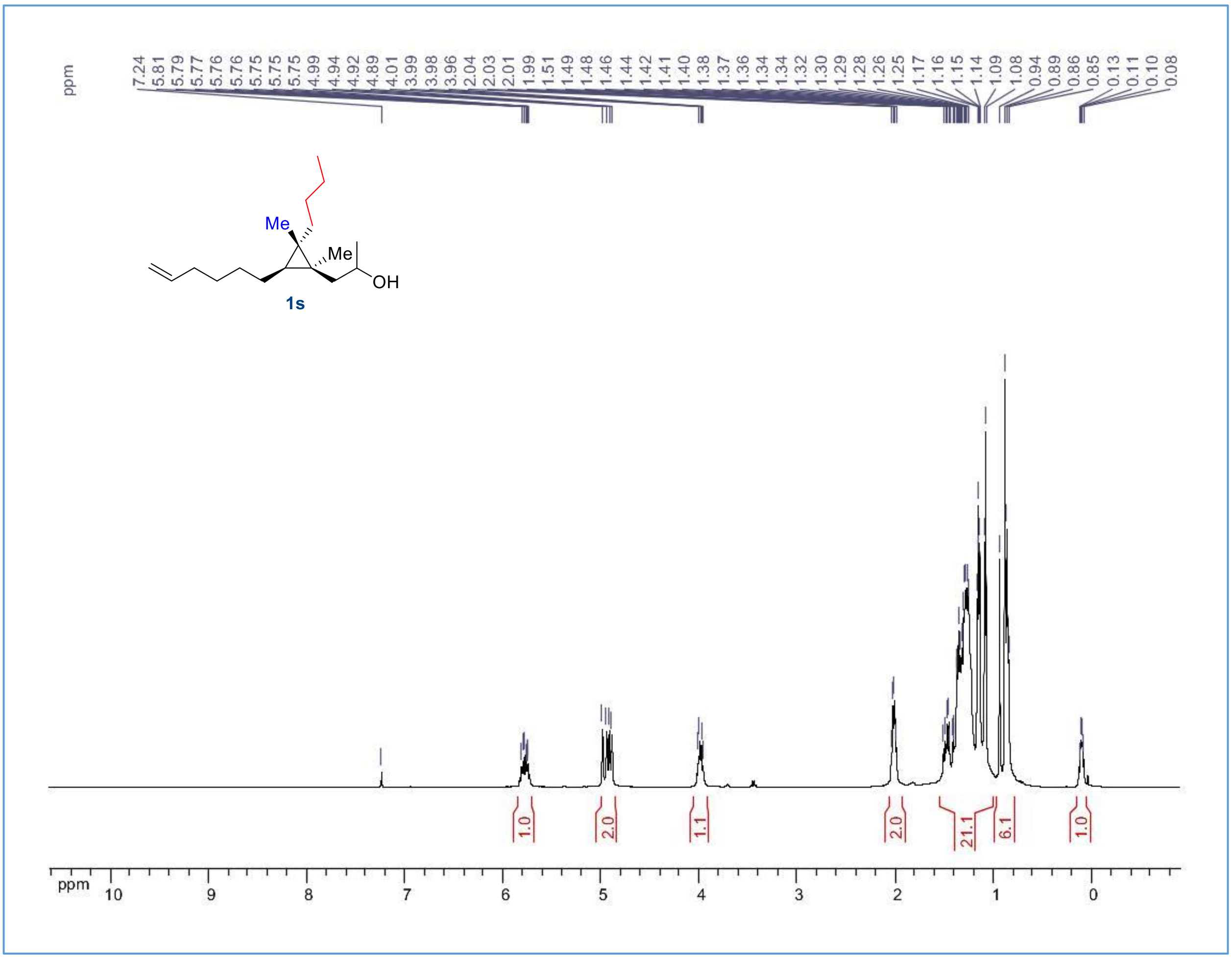

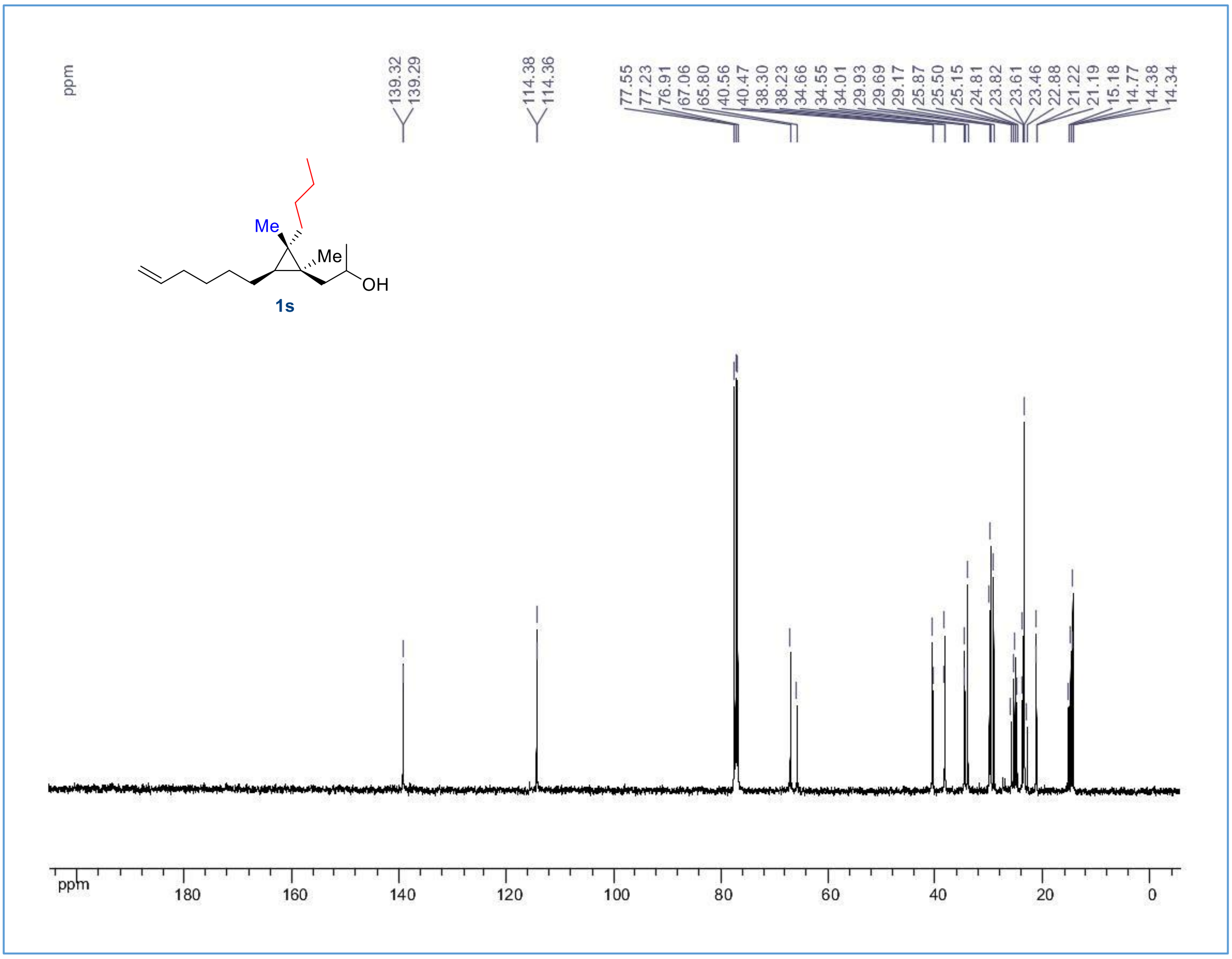

Supplementary Figure 31. ^{1}H and ^{13}C NMR spectra of compound **1s**

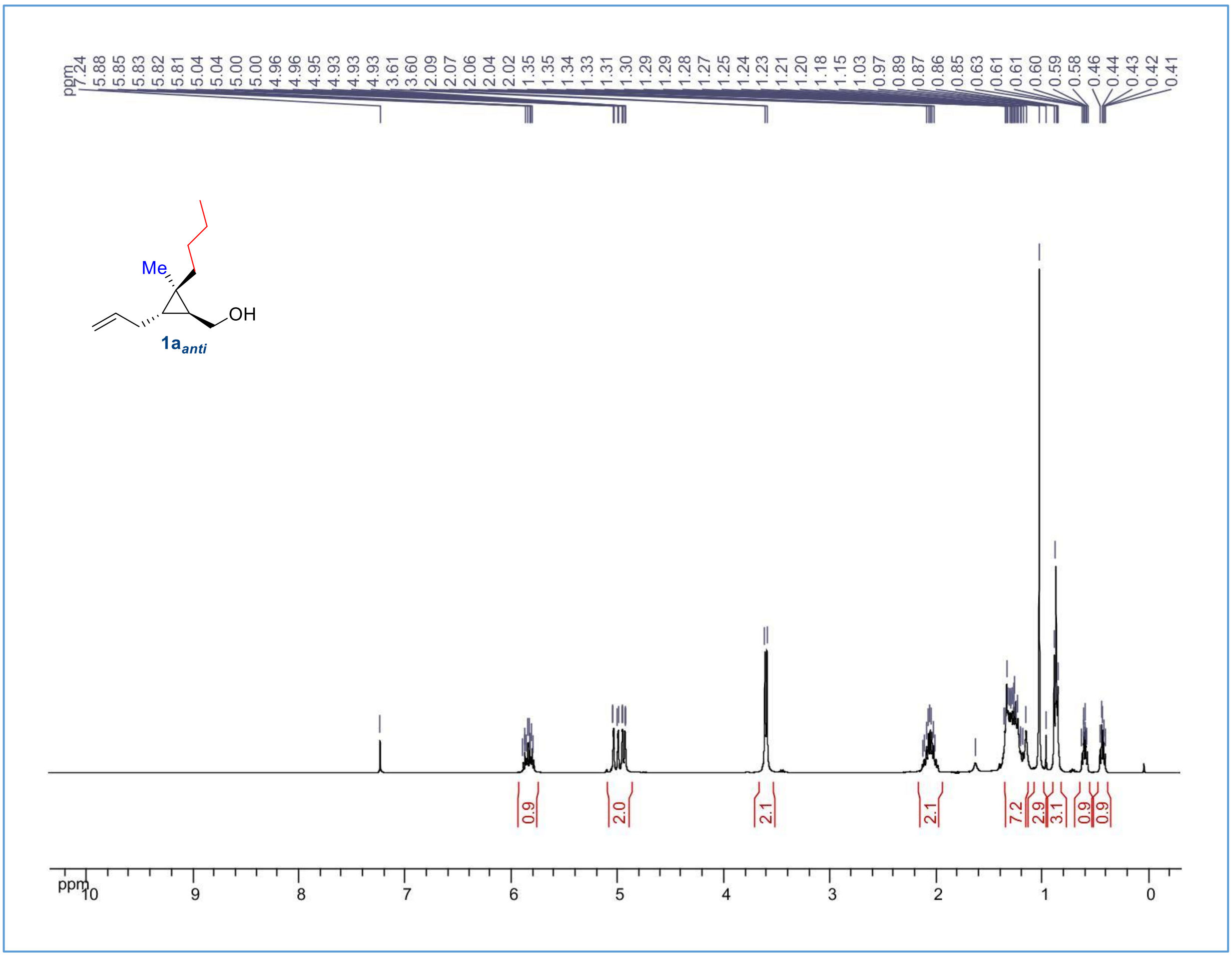

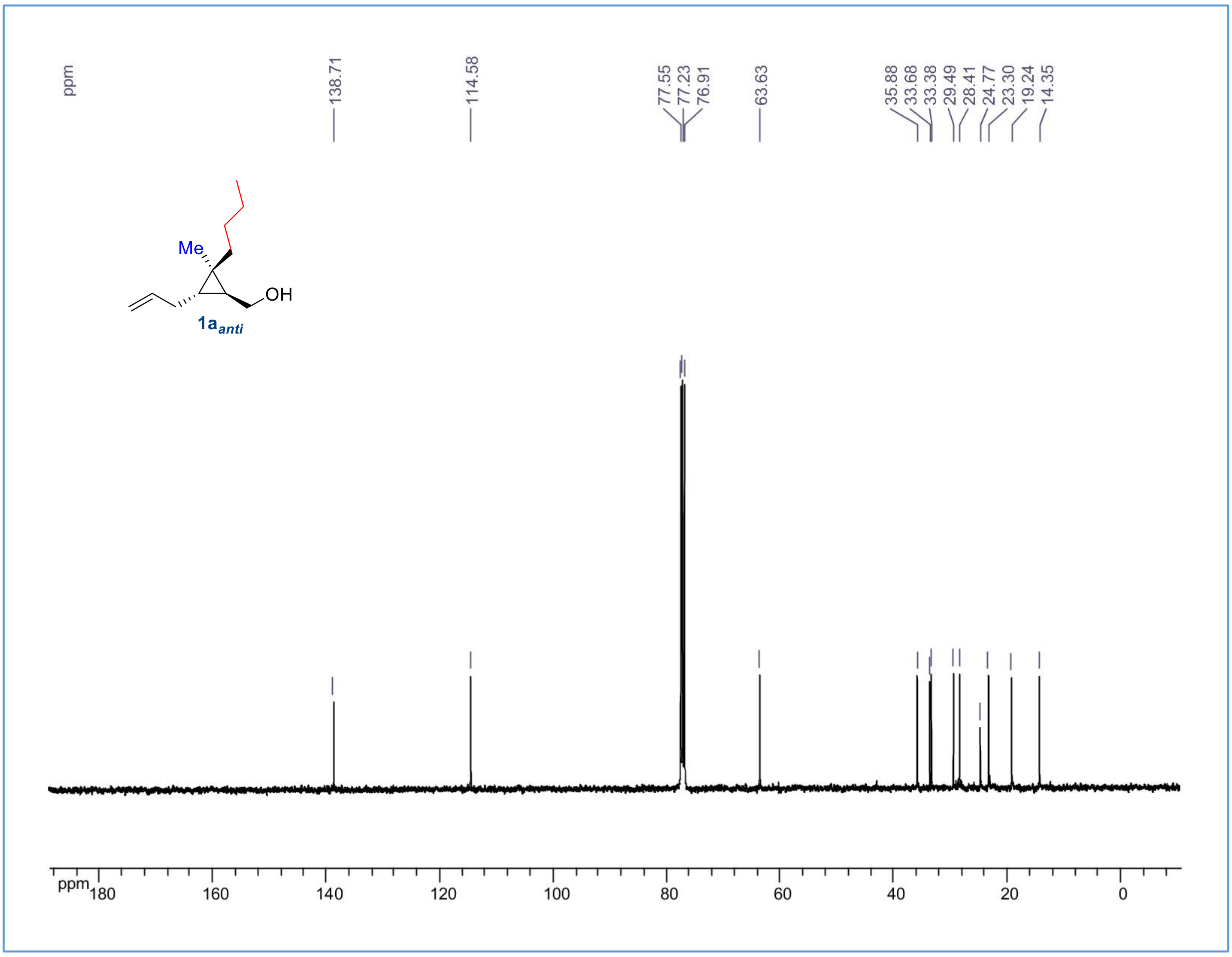

Supplementary Figure 32. ^{1}H and ^{13}C NMR spectra of compound **1a*$_{anti}$***

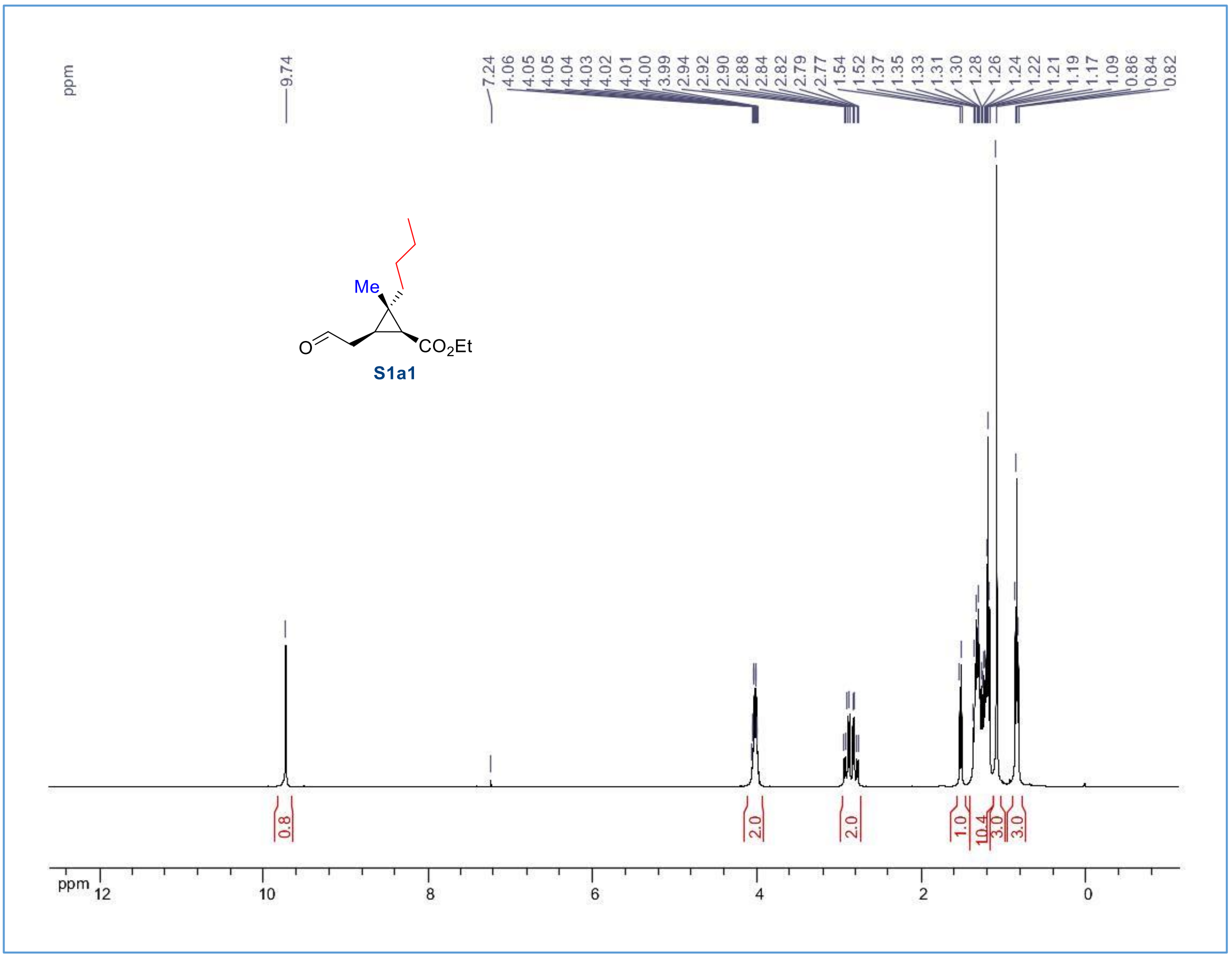

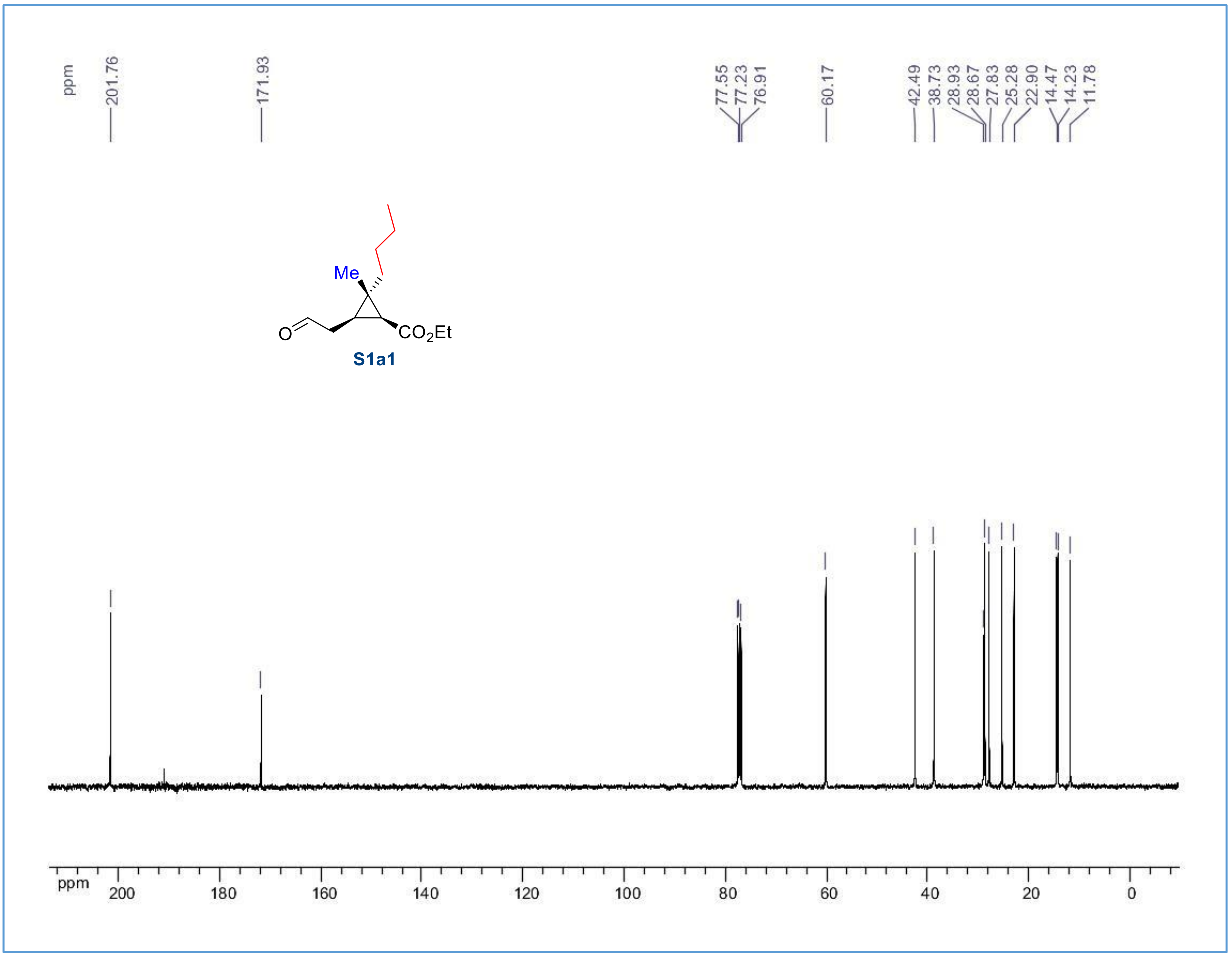

Supplementary Figure 33. ^{1}H and ^{13}C NMR spectra of compound **S1a1**

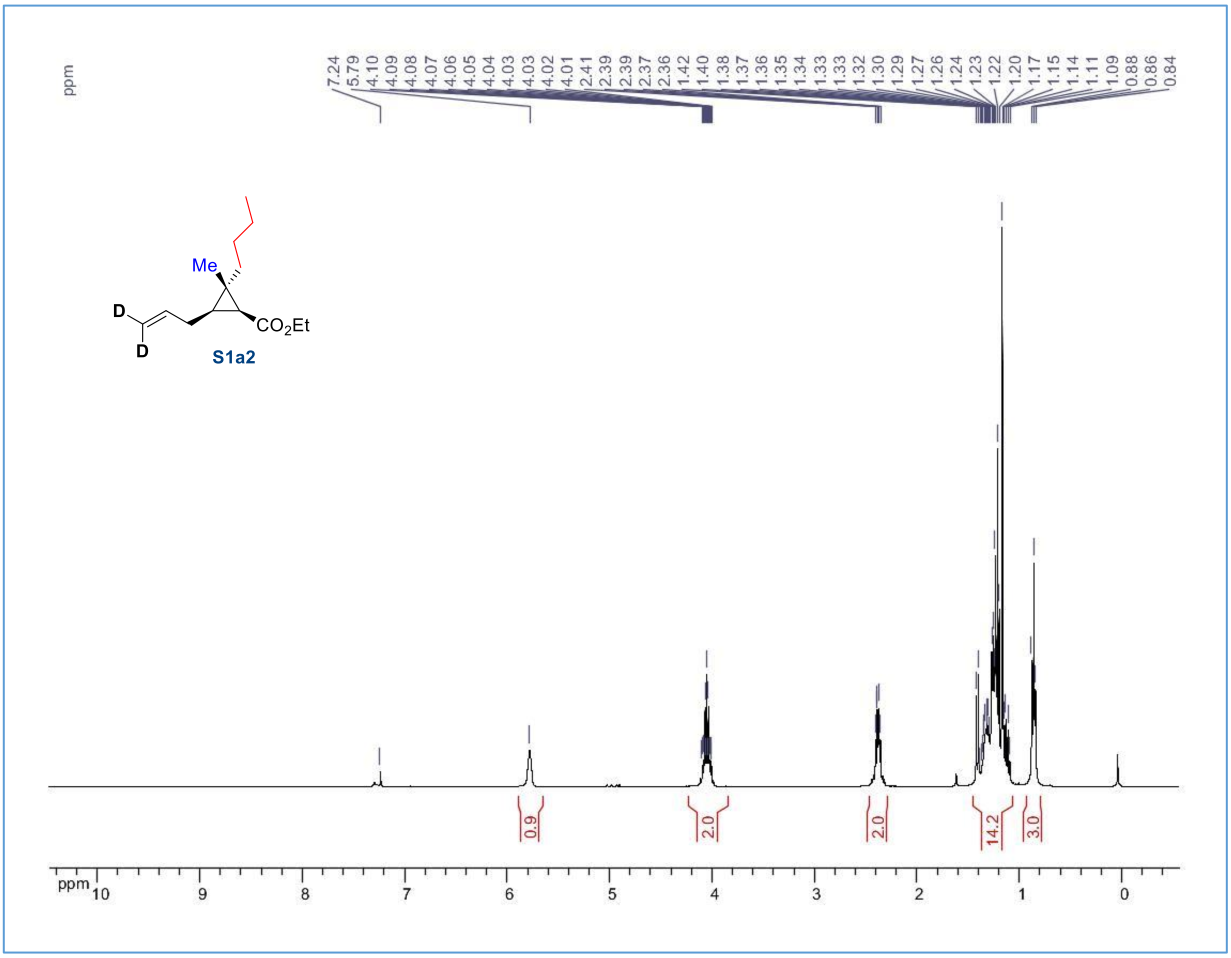

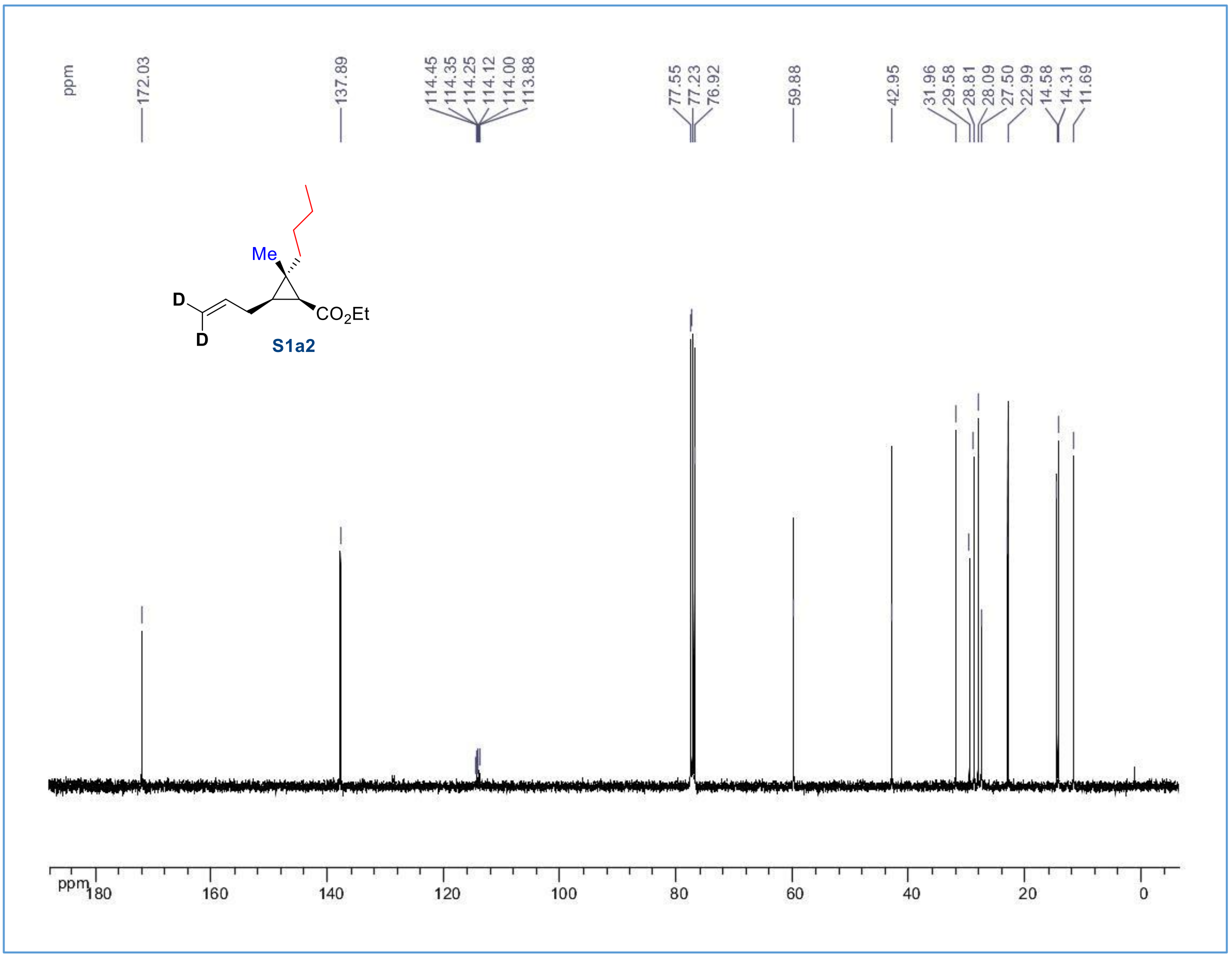

Supplementary Figure 34. ^{1}H and ^{13}C NMR spectra of compound **S1a2**

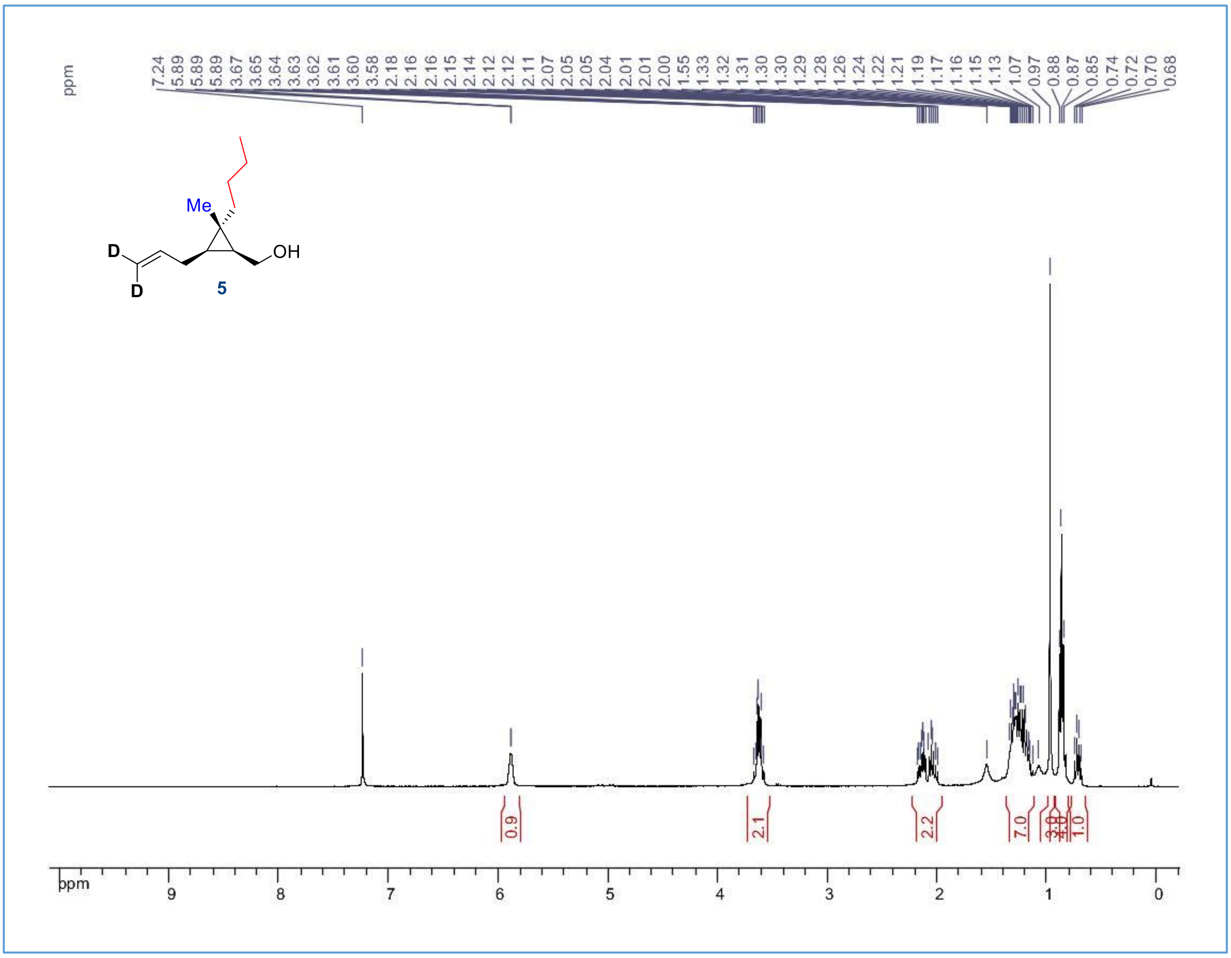

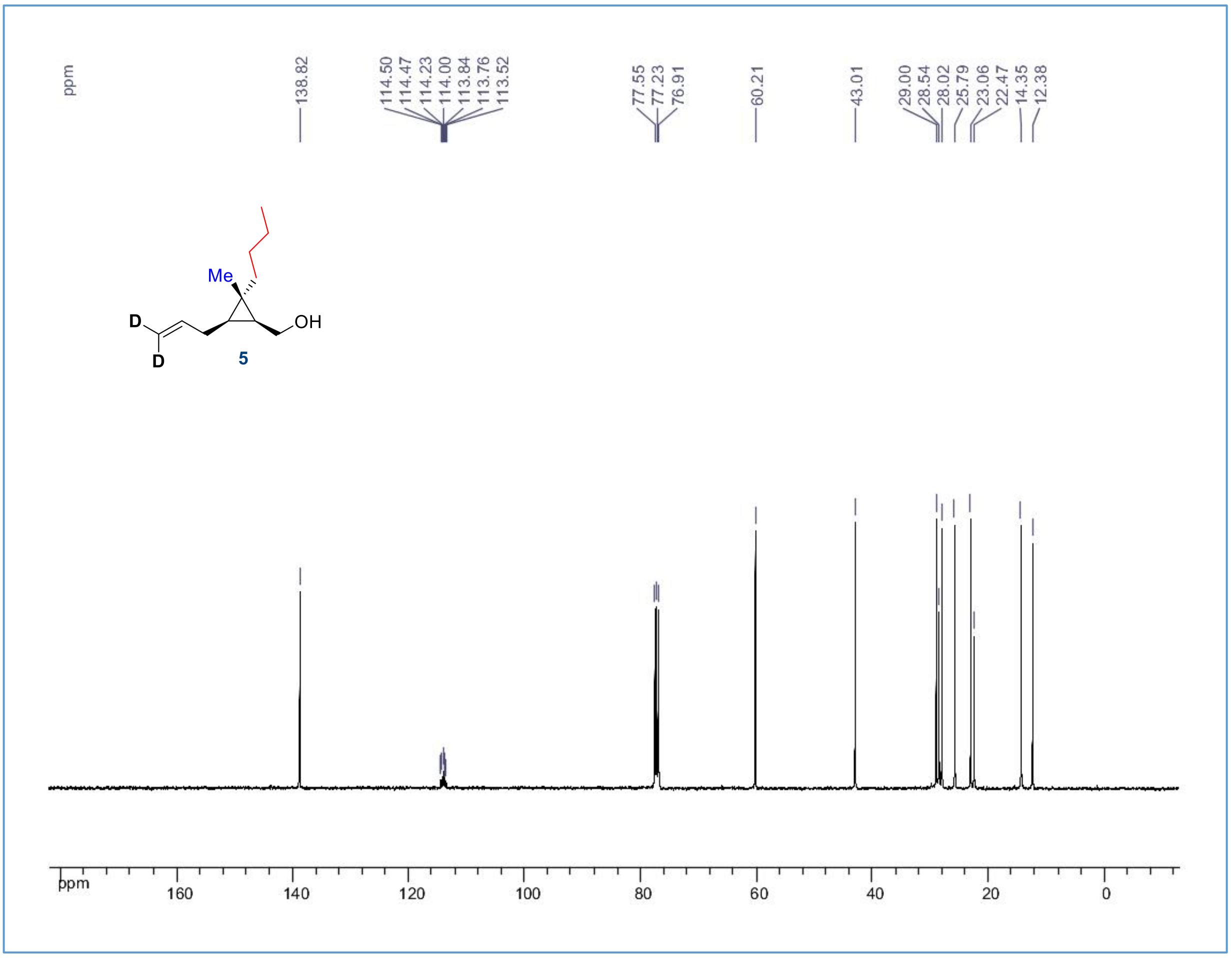

Supplementary Figure 35. ^{1}H and ^{13}C NMR spectra of compound **5**

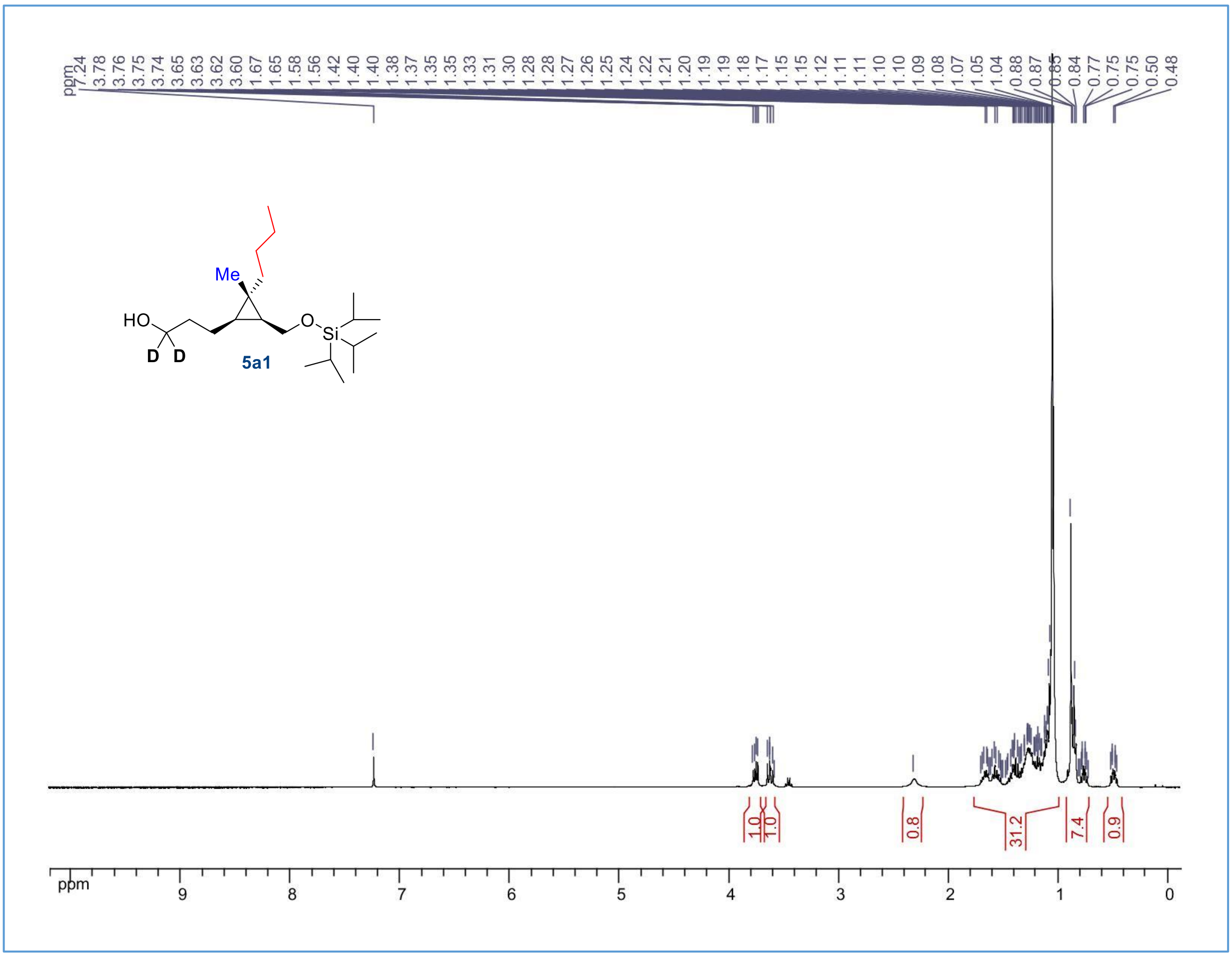

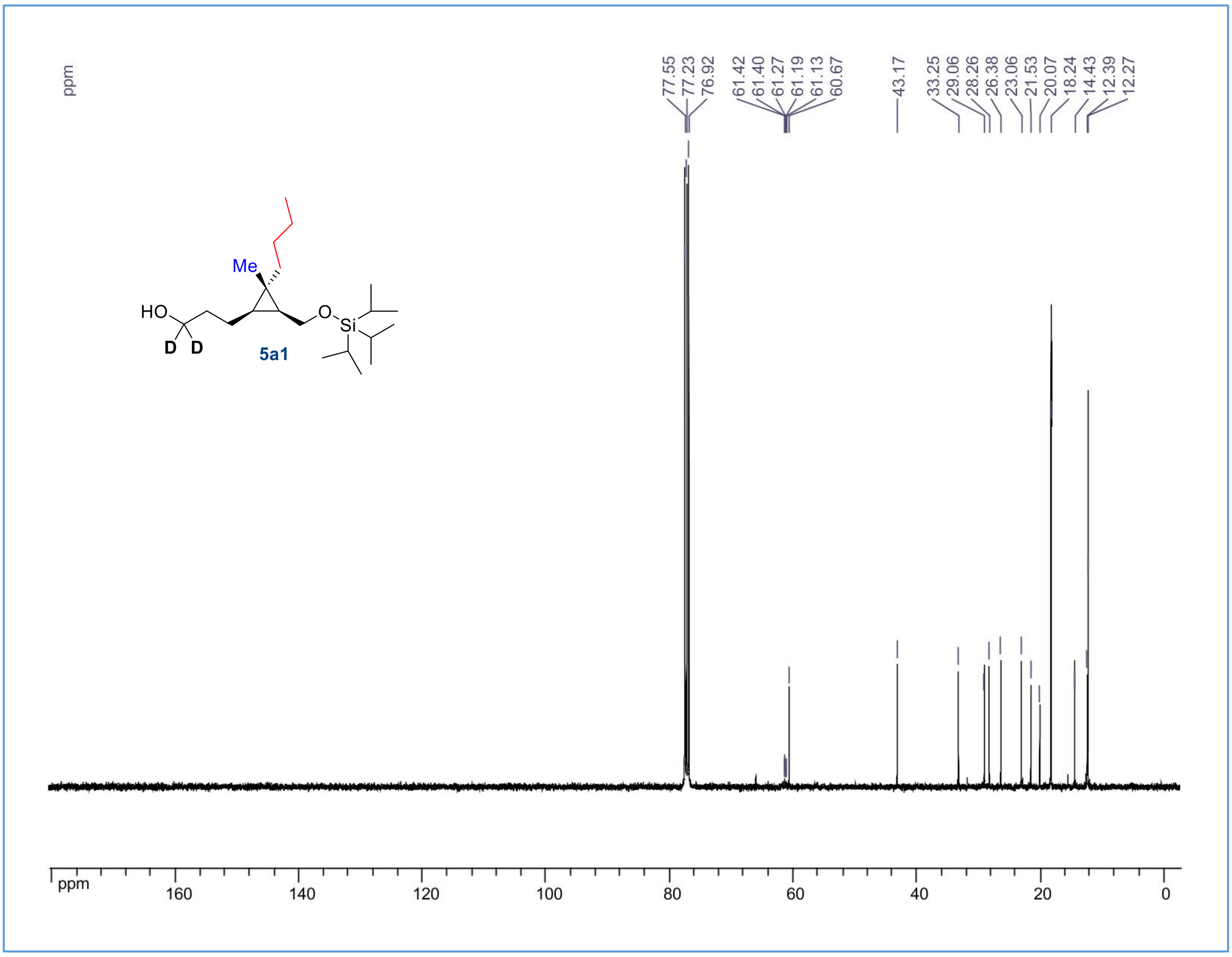

Supplementary Figure 36. ^{1}H and ^{13}C NMR spectra of compound **5a1**

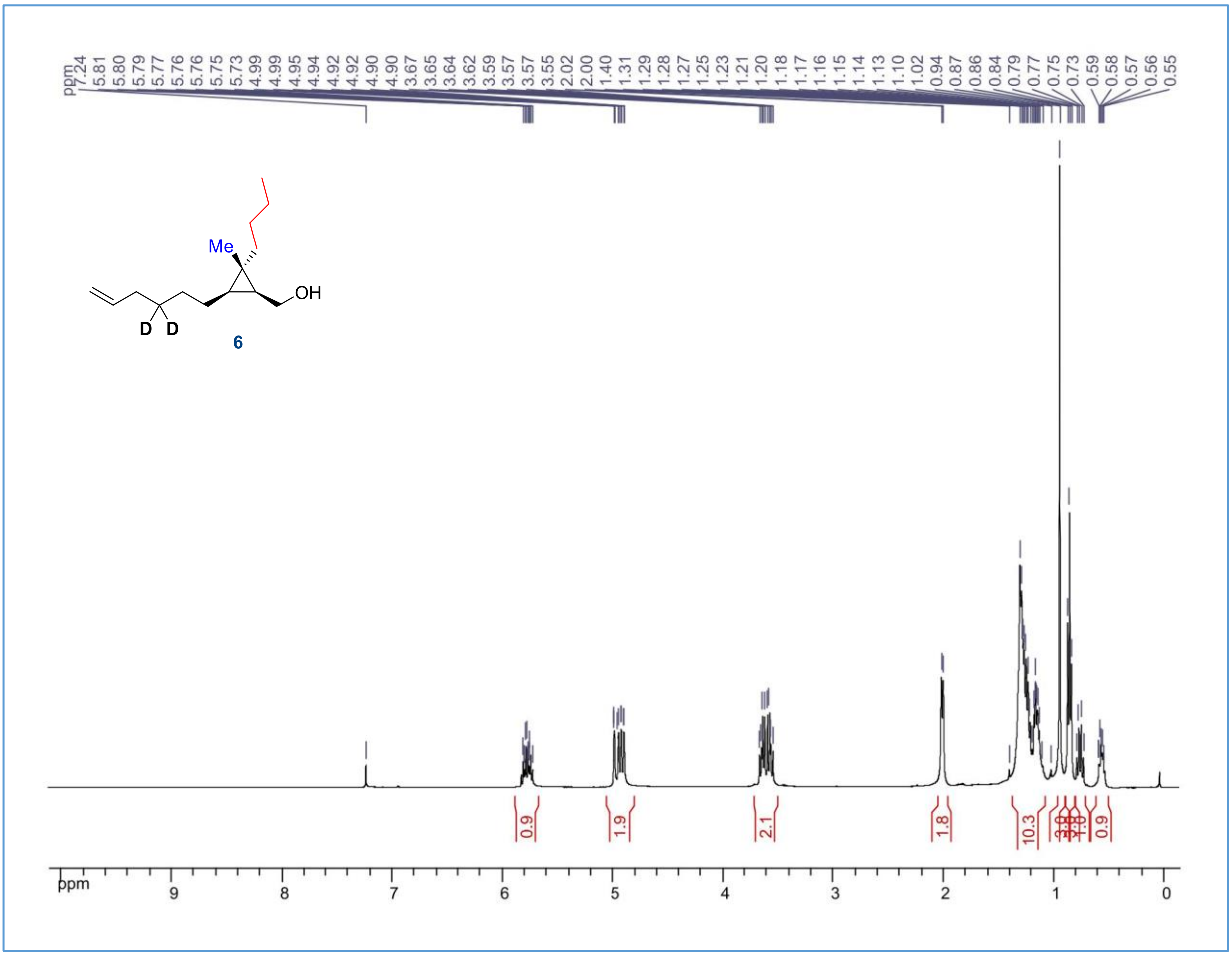

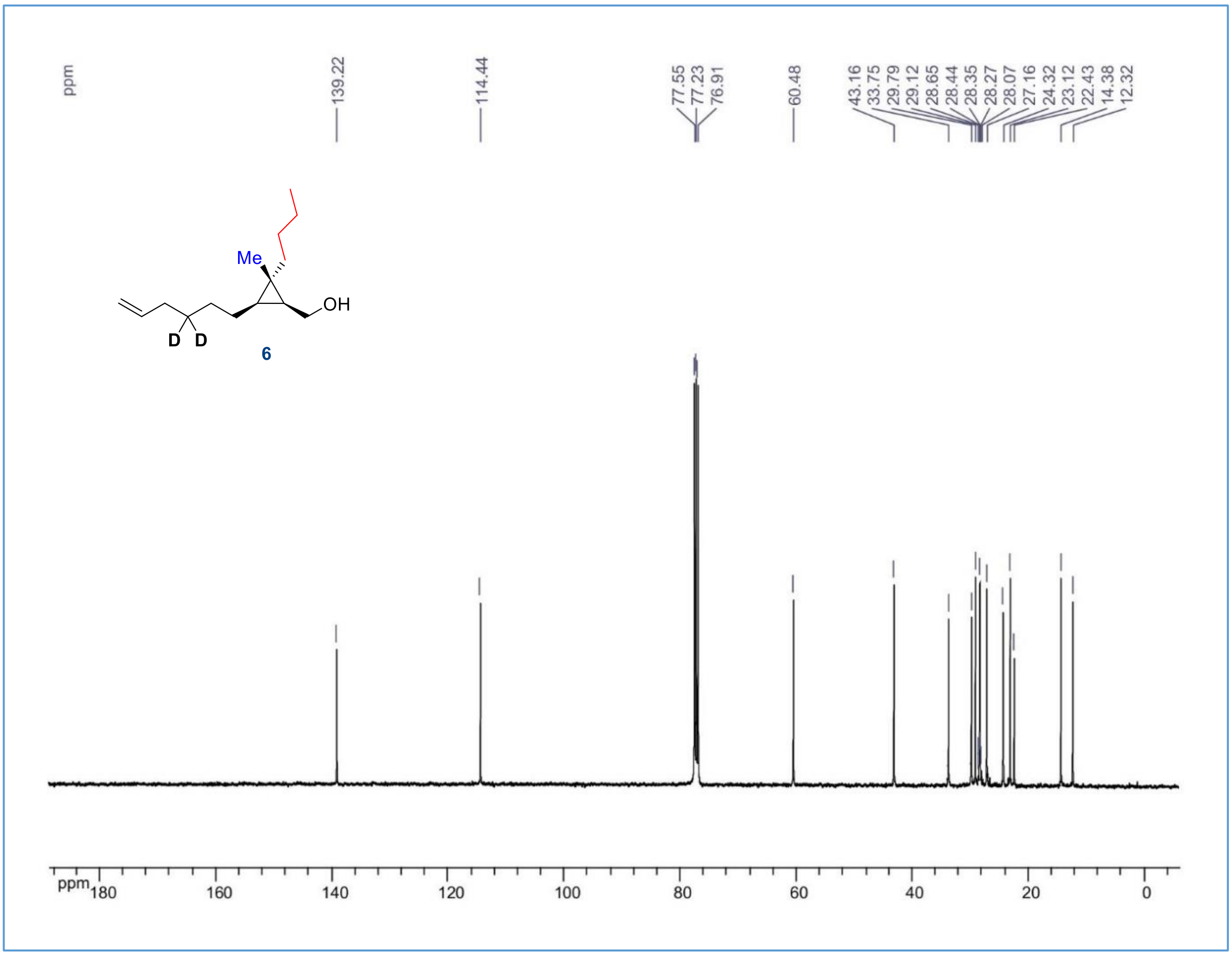

Supplementary Figure 37. ^{1}H and ^{13}C NMR spectra of compound **6**

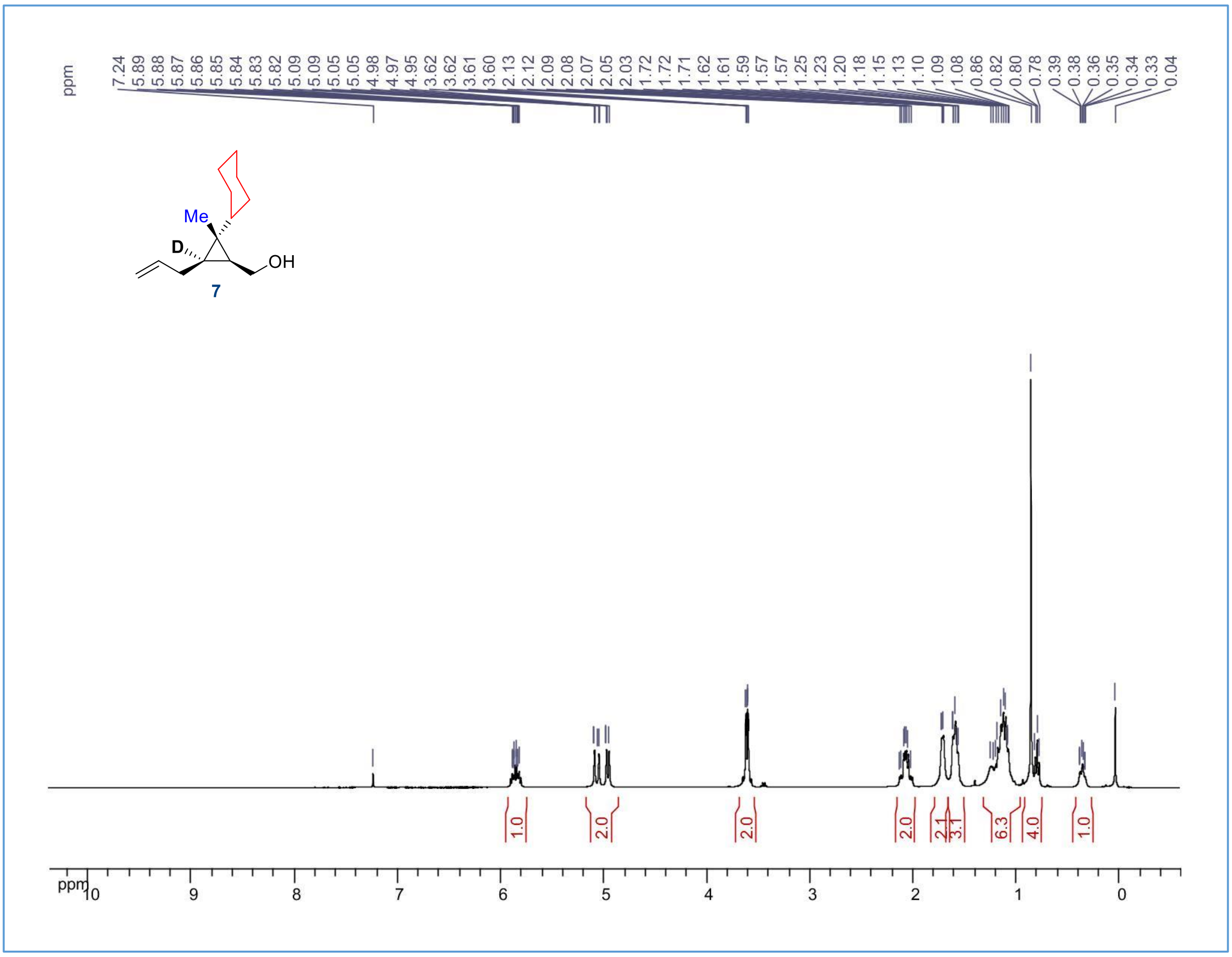

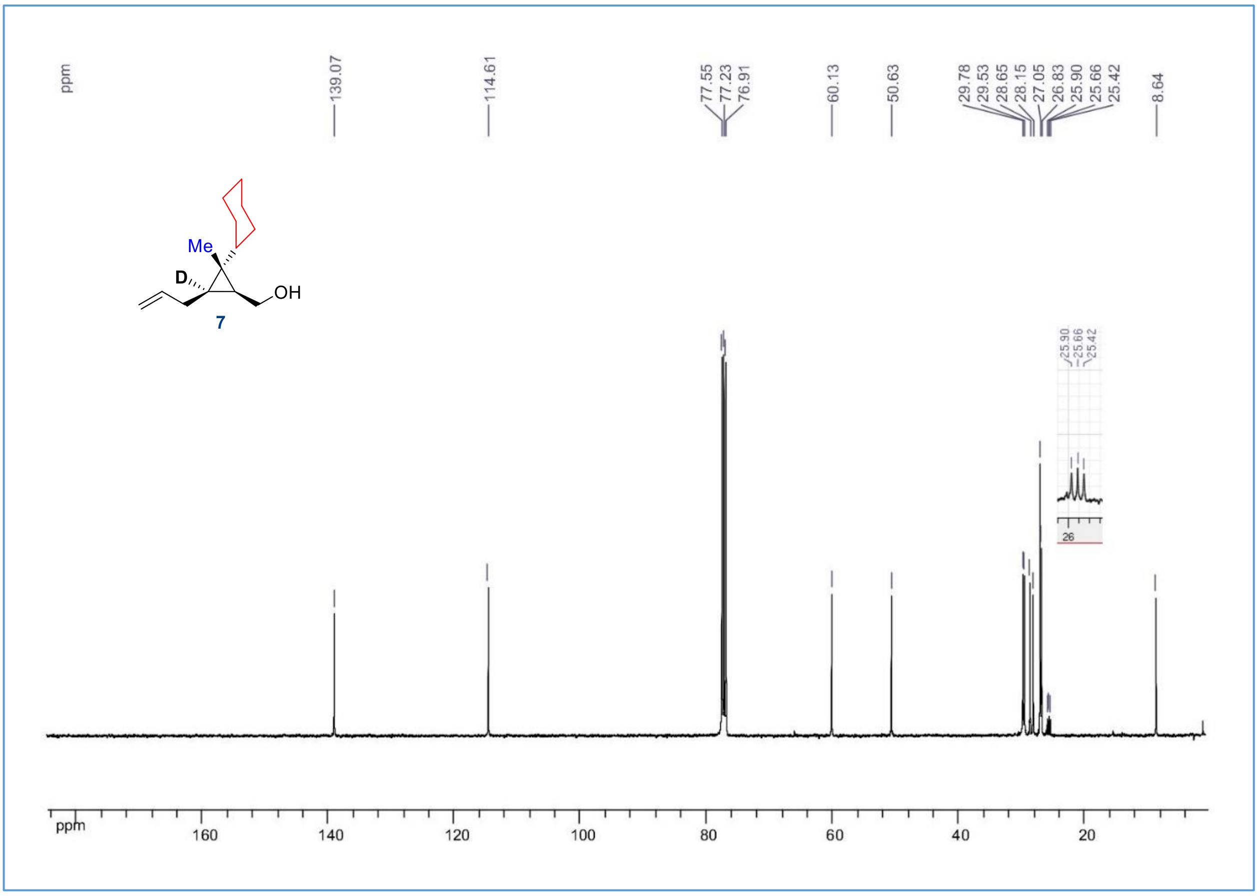

Supplementary Figure 38. ^{1}H and ^{13}C NMR spectra of compound **7**

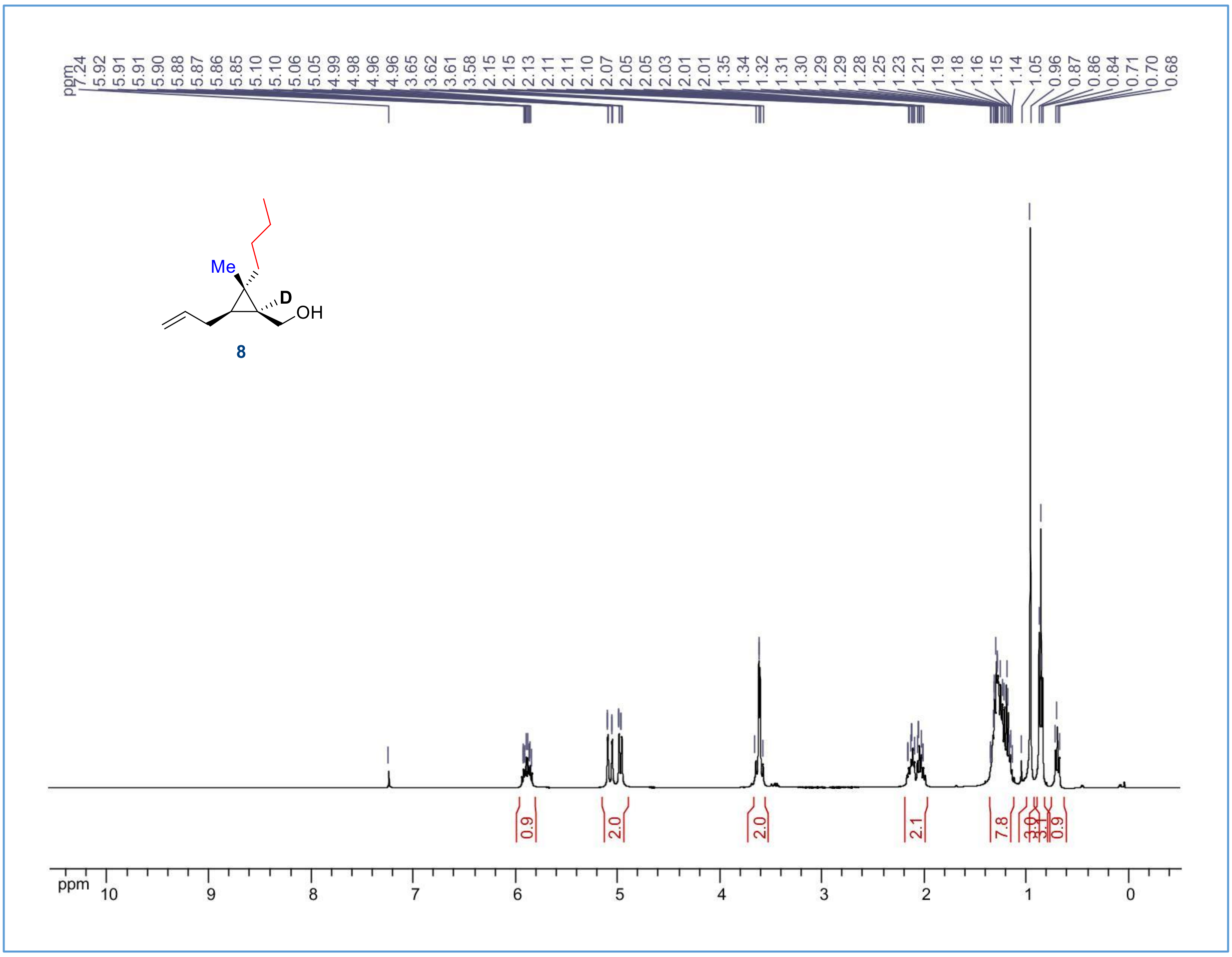

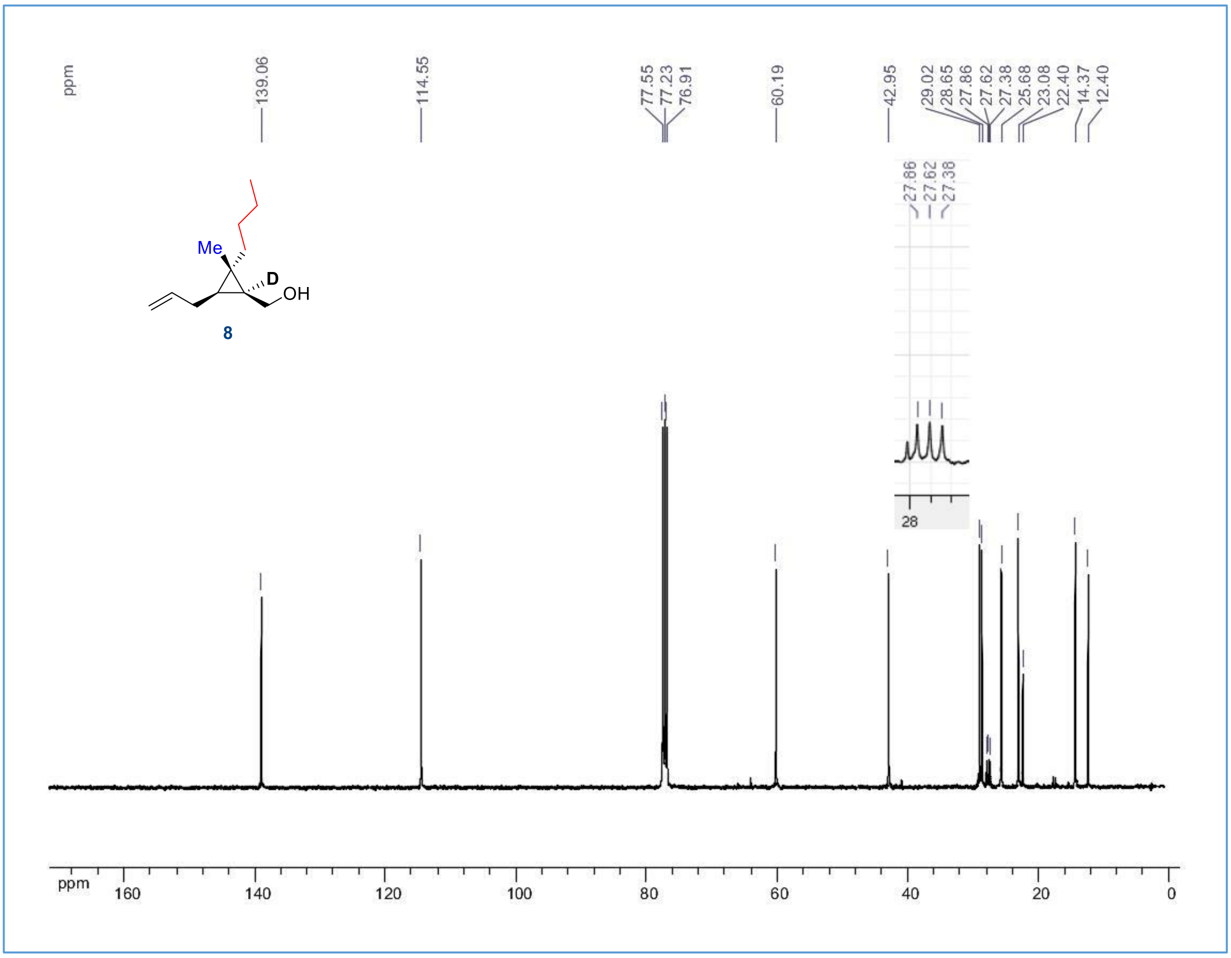

Supplementary Figure 39. ^{1}H and ^{13}C NMR spectra of compound **8**

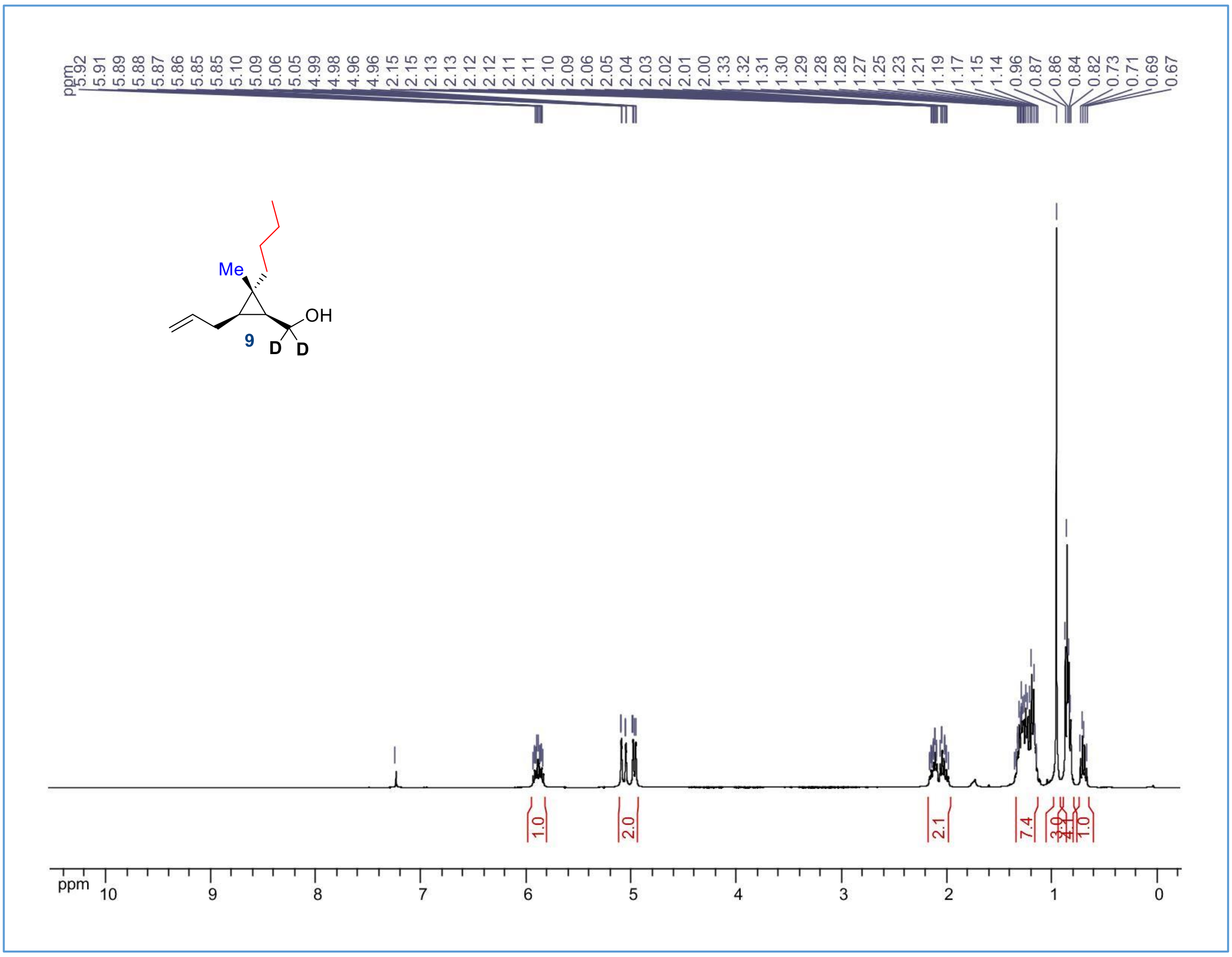

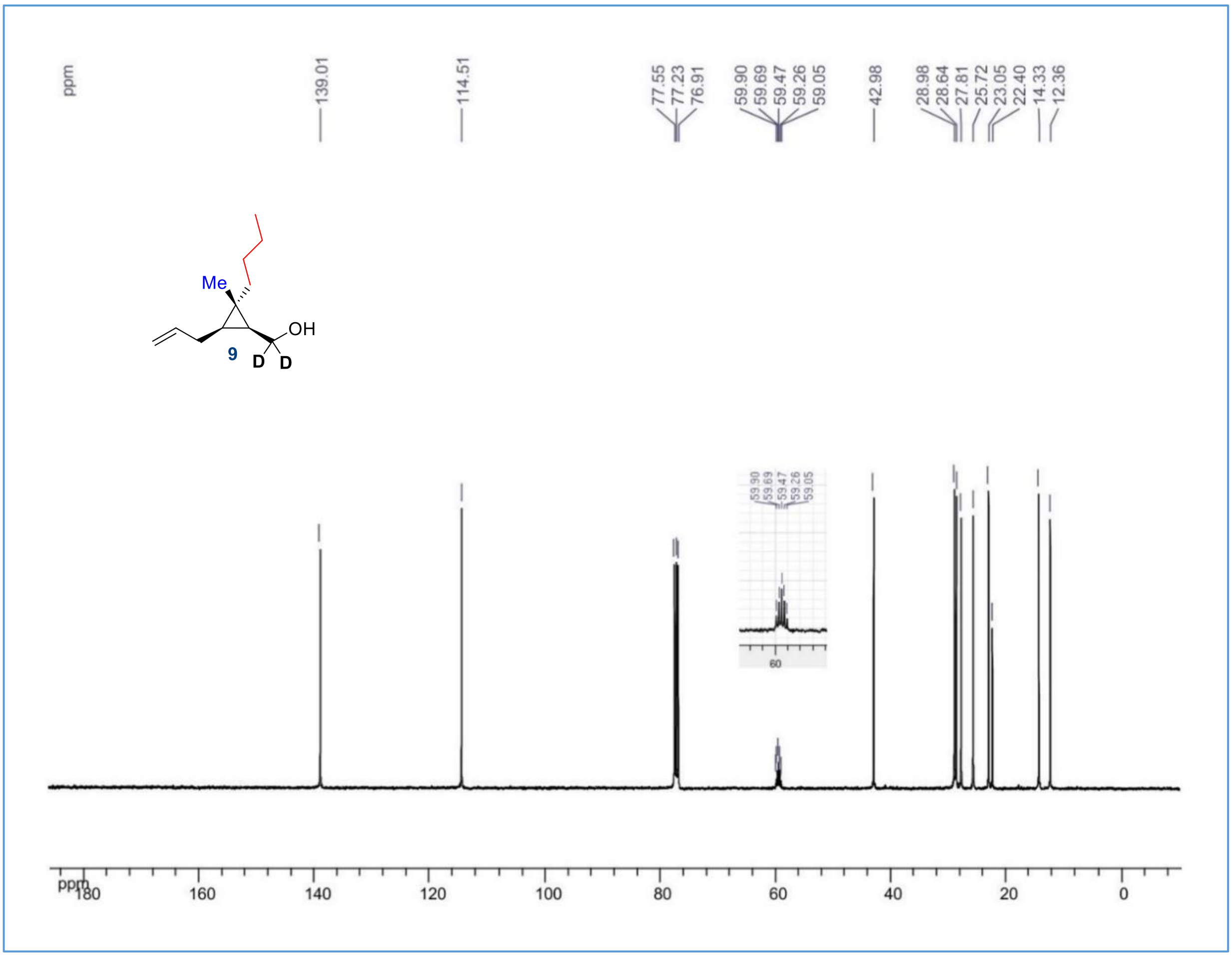

Supplementary Figure 40. ^{1}H and ^{13}C NMR spectra of compound **9**

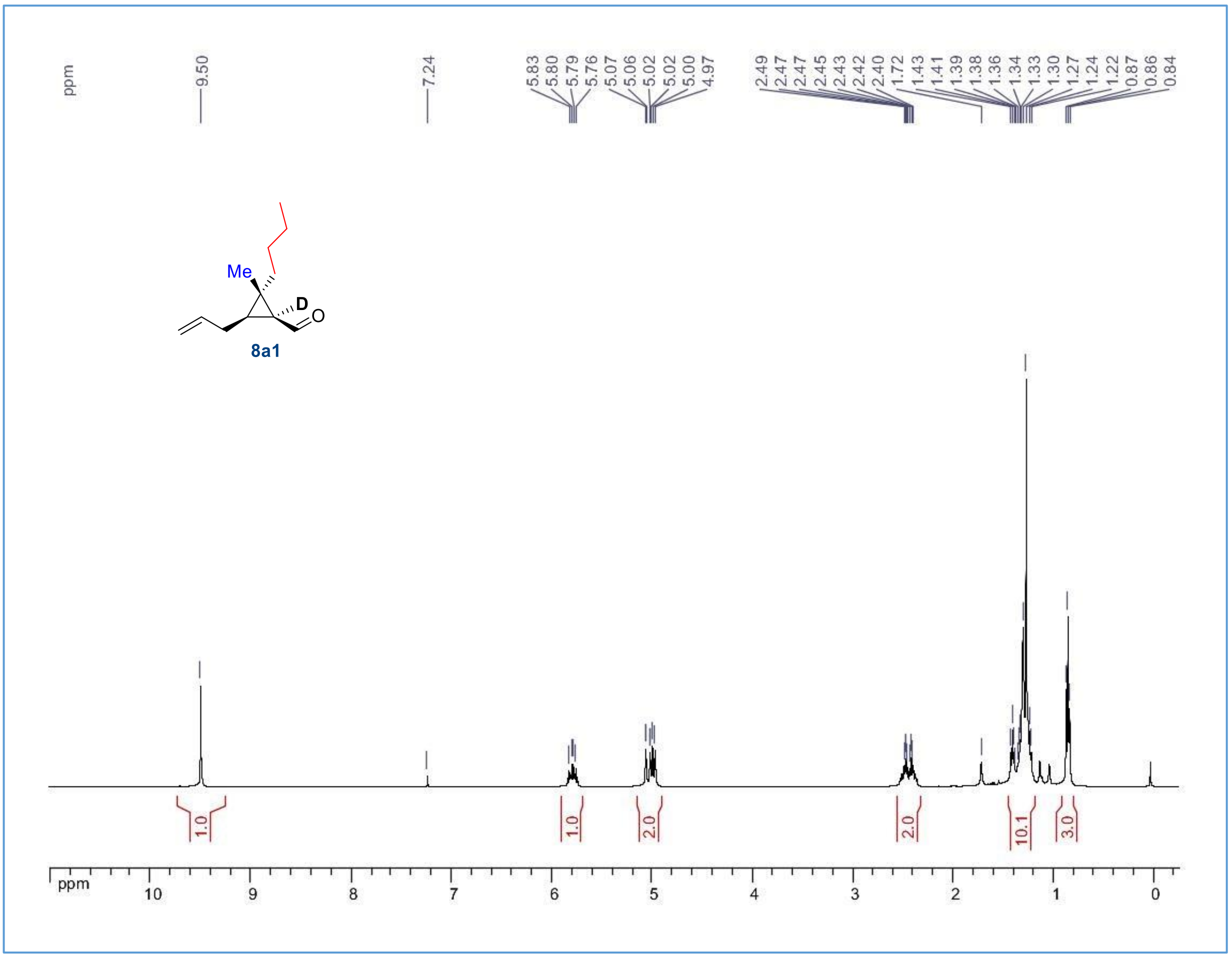

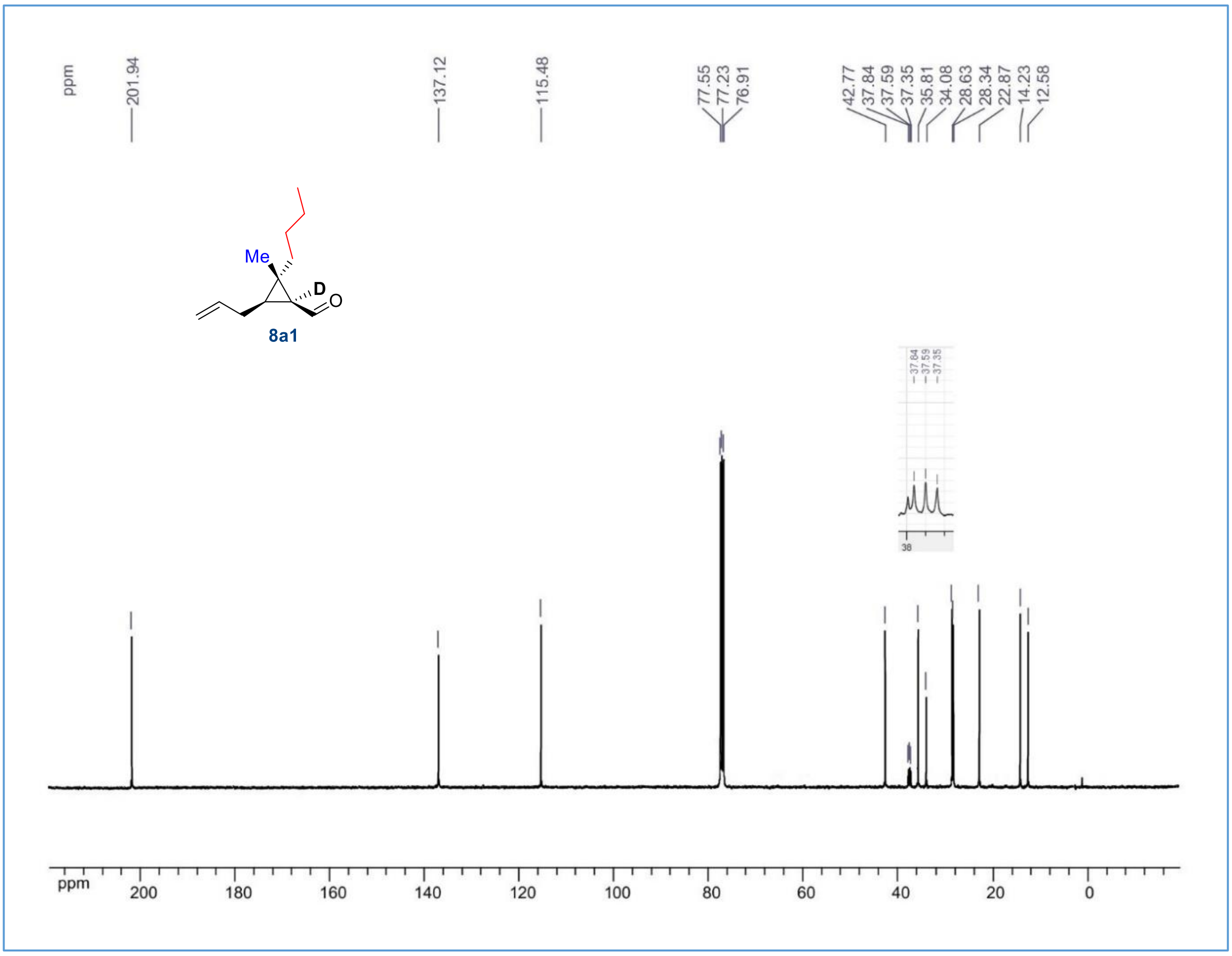

Supplementary Figure 41. ^{1}H and ^{13}C NMR spectra of compound **8a1**

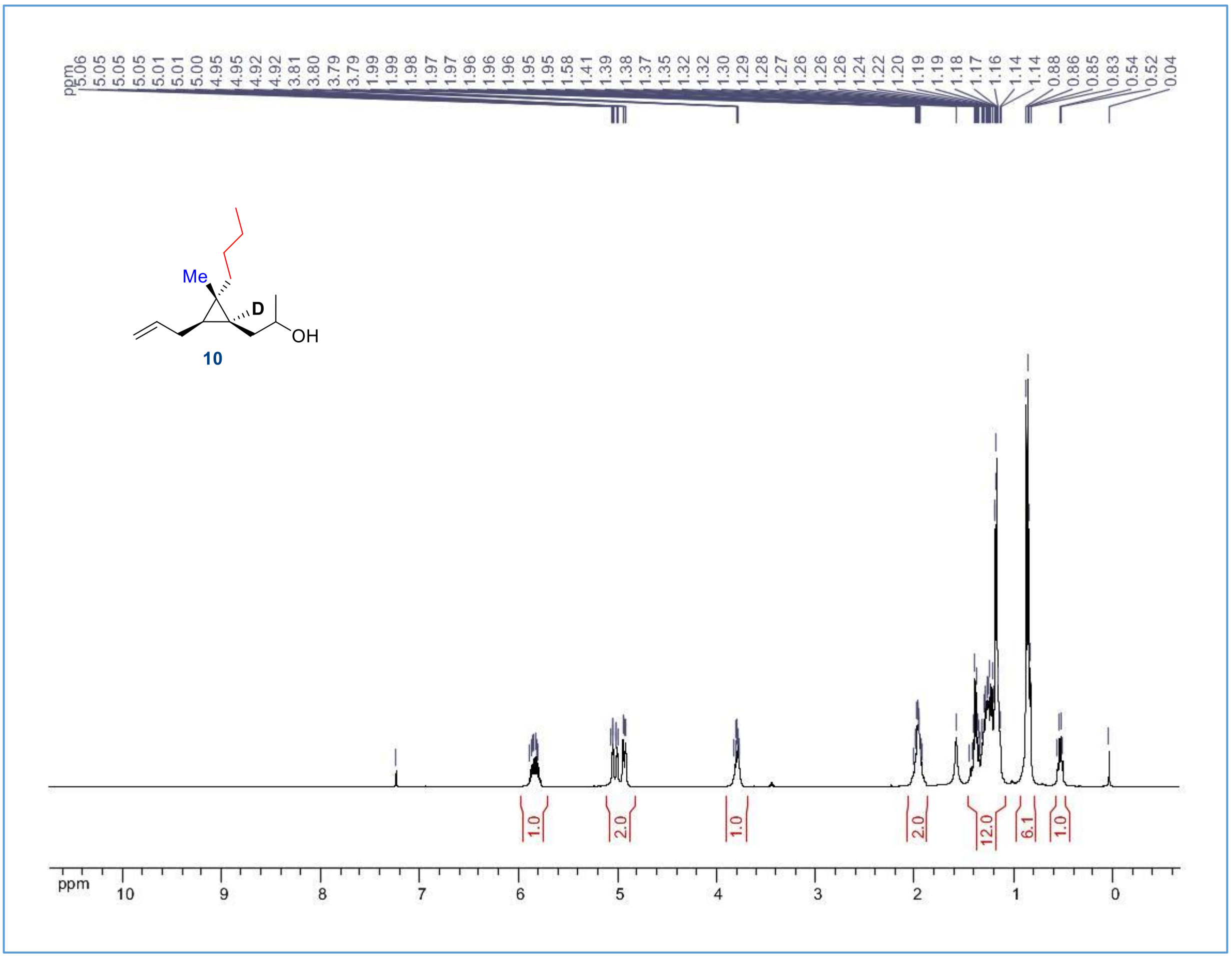

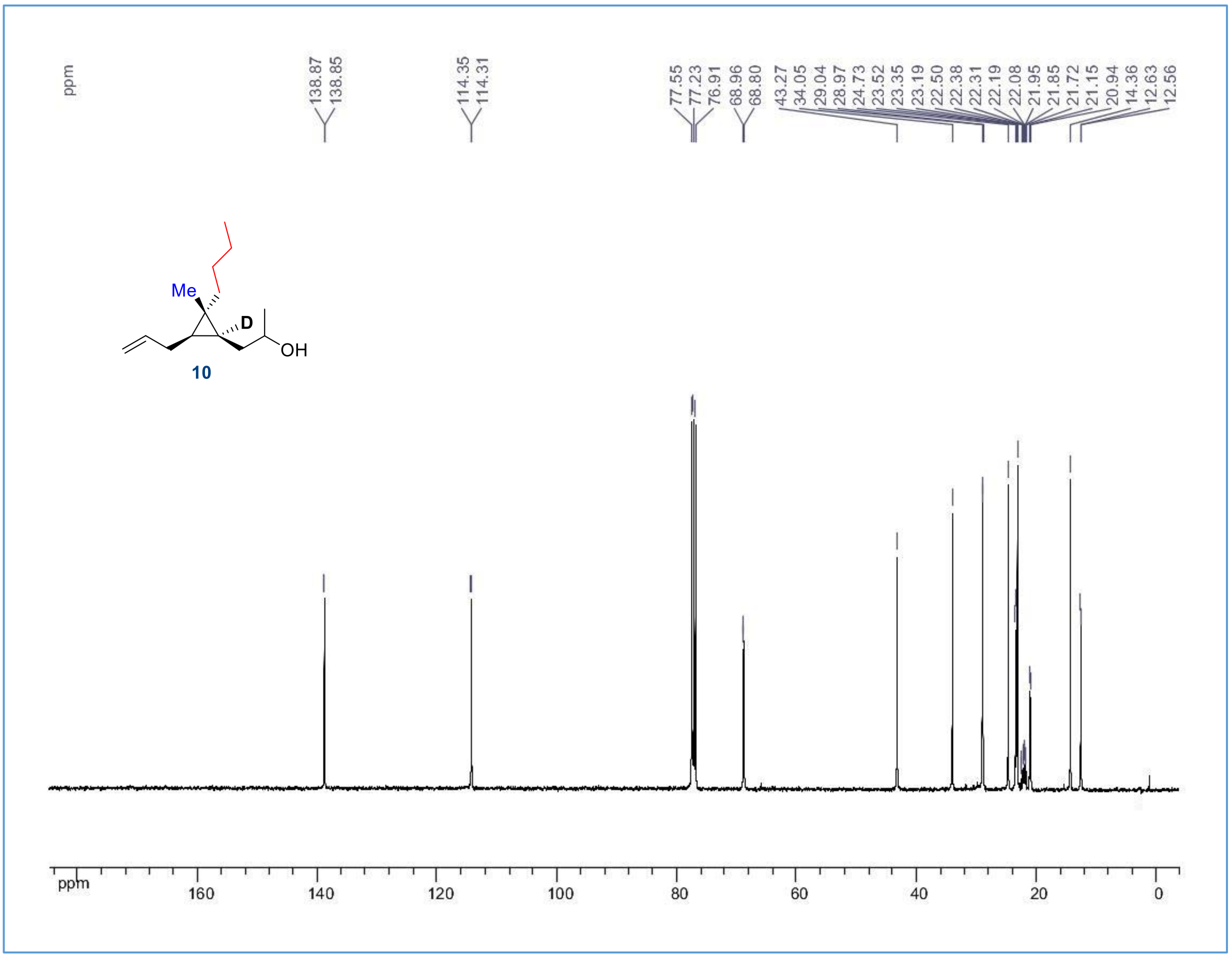

Supplementary Figure 42. ^{1}H and ^{13}C NMR spectra of compound **10**

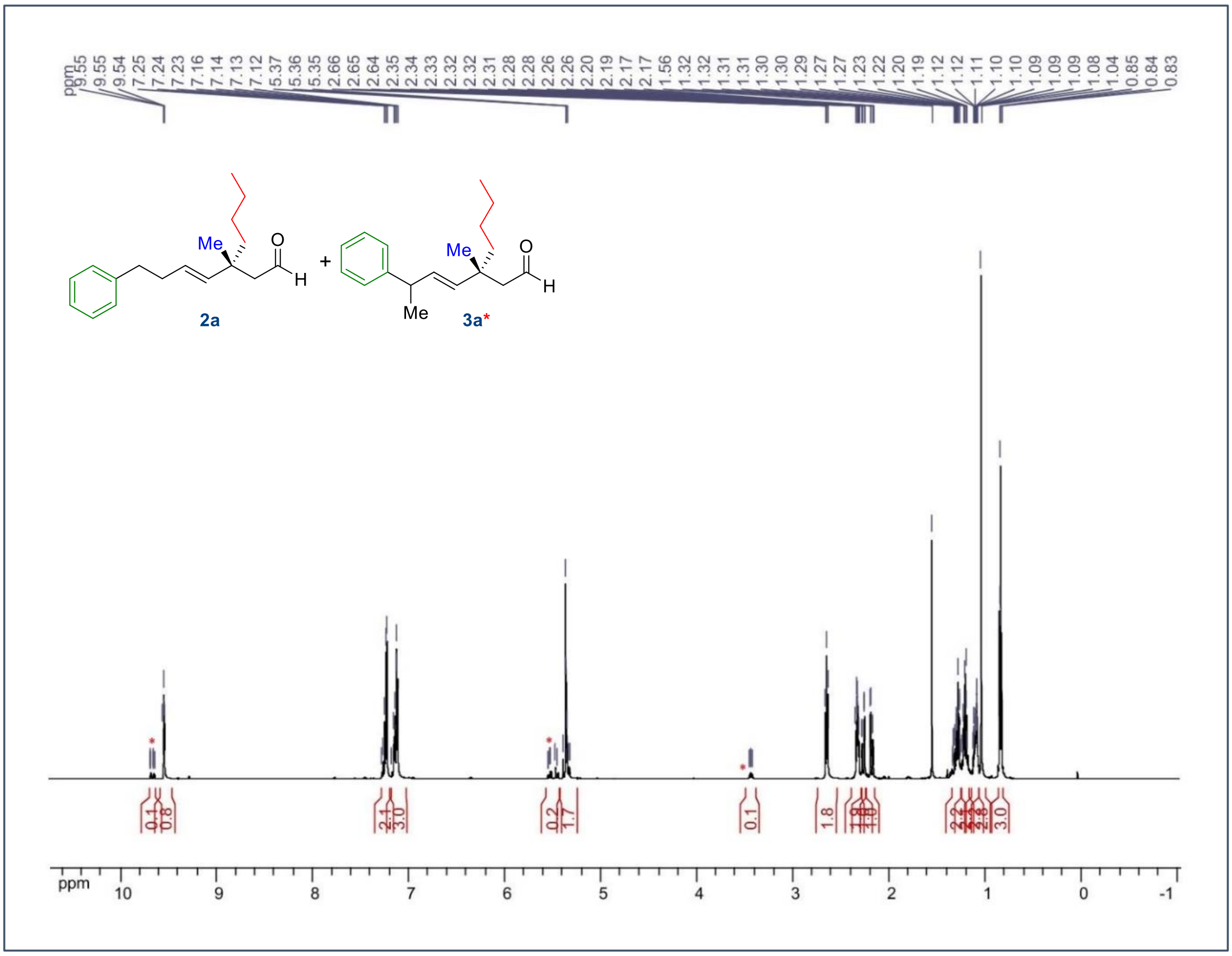

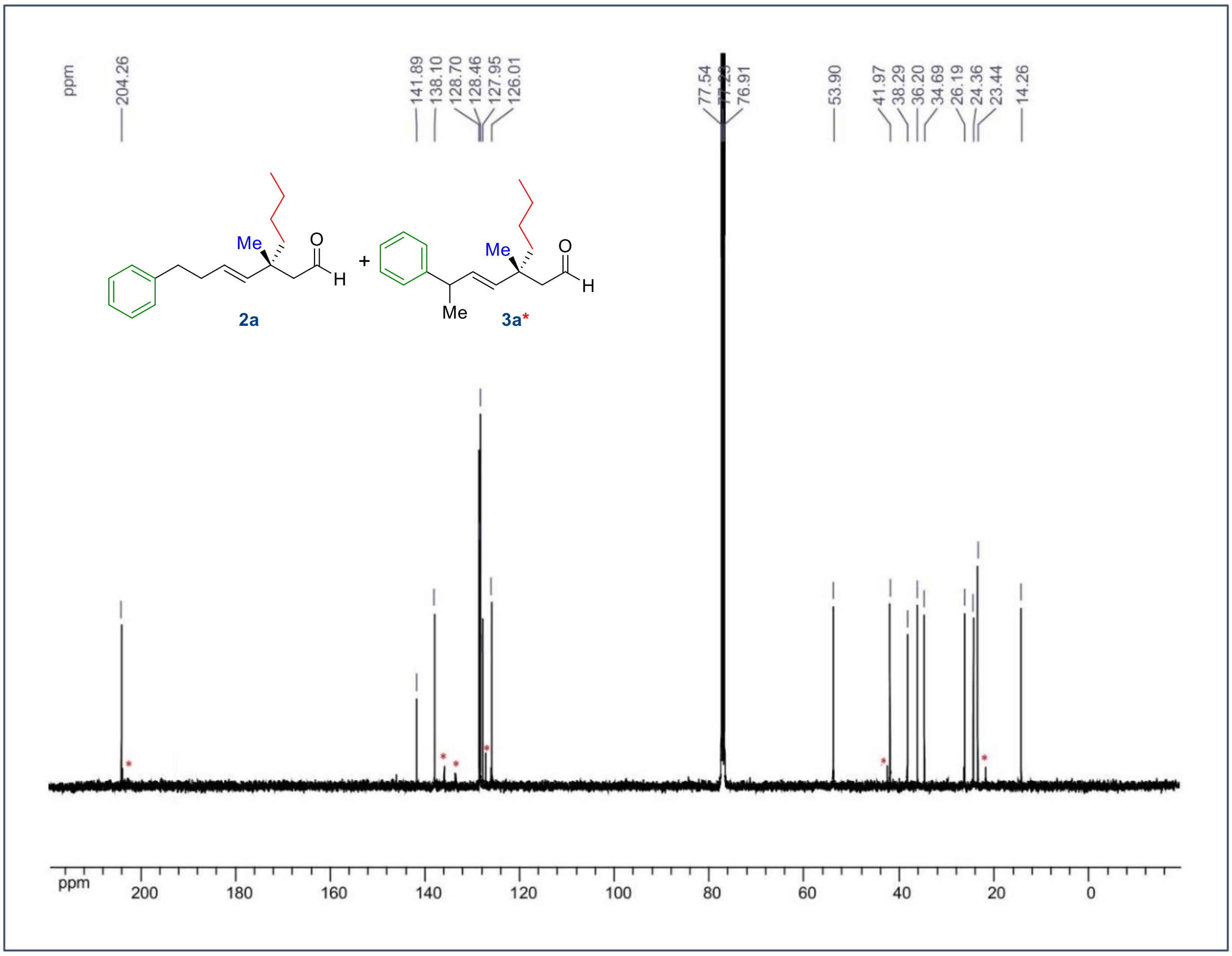

Supplementary Figure 43. ^{1}H and ^{13}C NMR spectra of compound **2a**

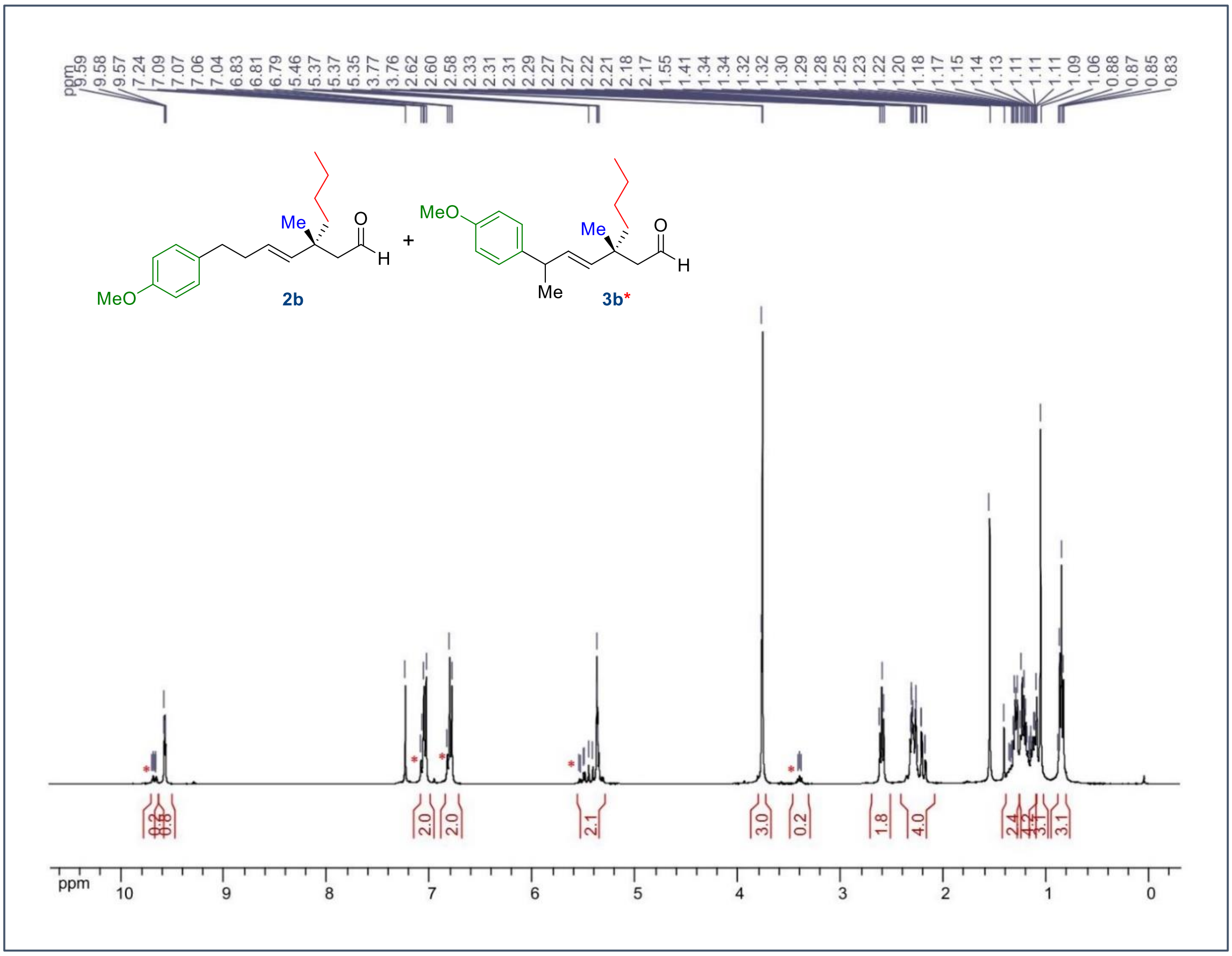

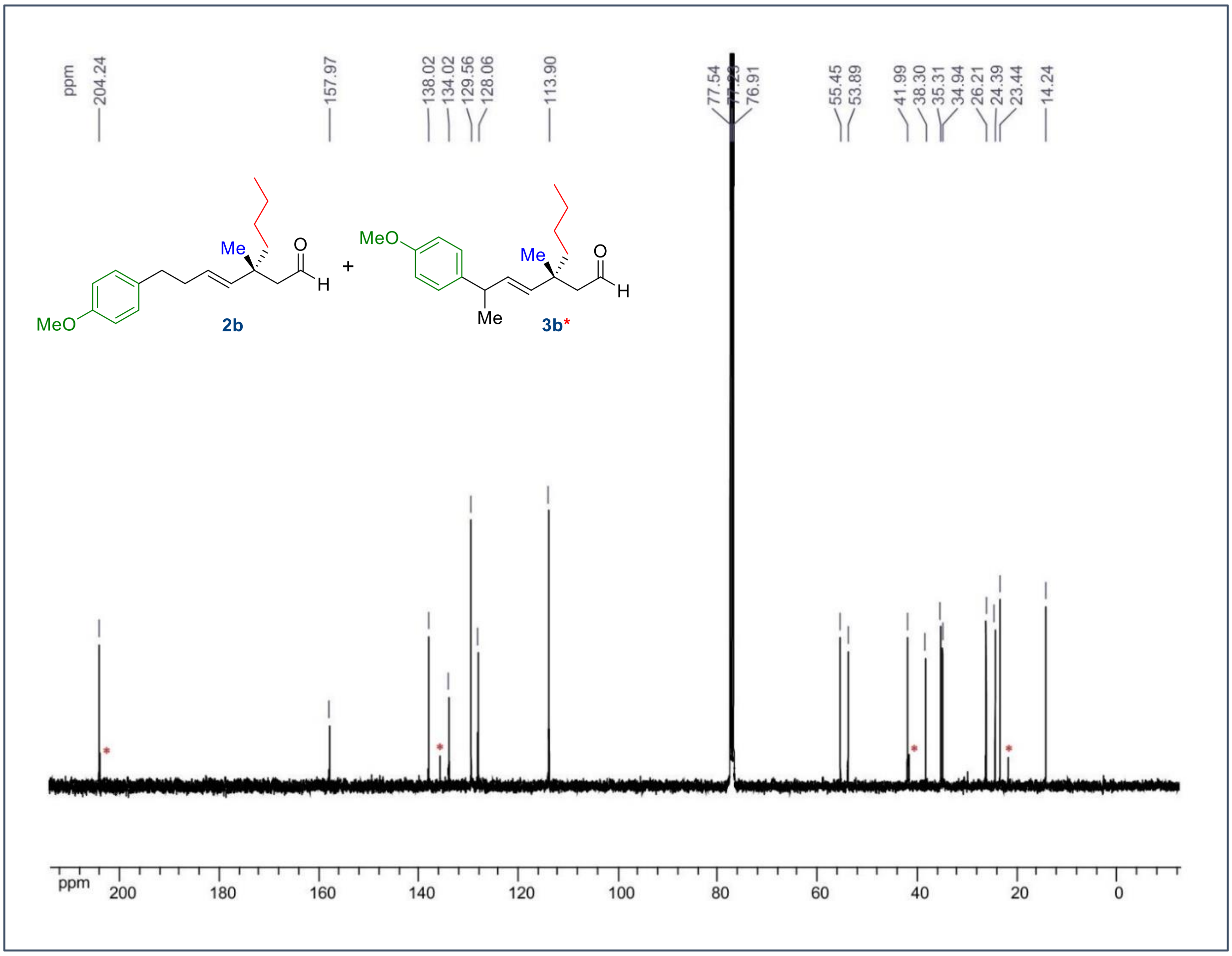

Supplementary Figure 44. ^{1}H and ^{13}C NMR spectra of compound **2b**

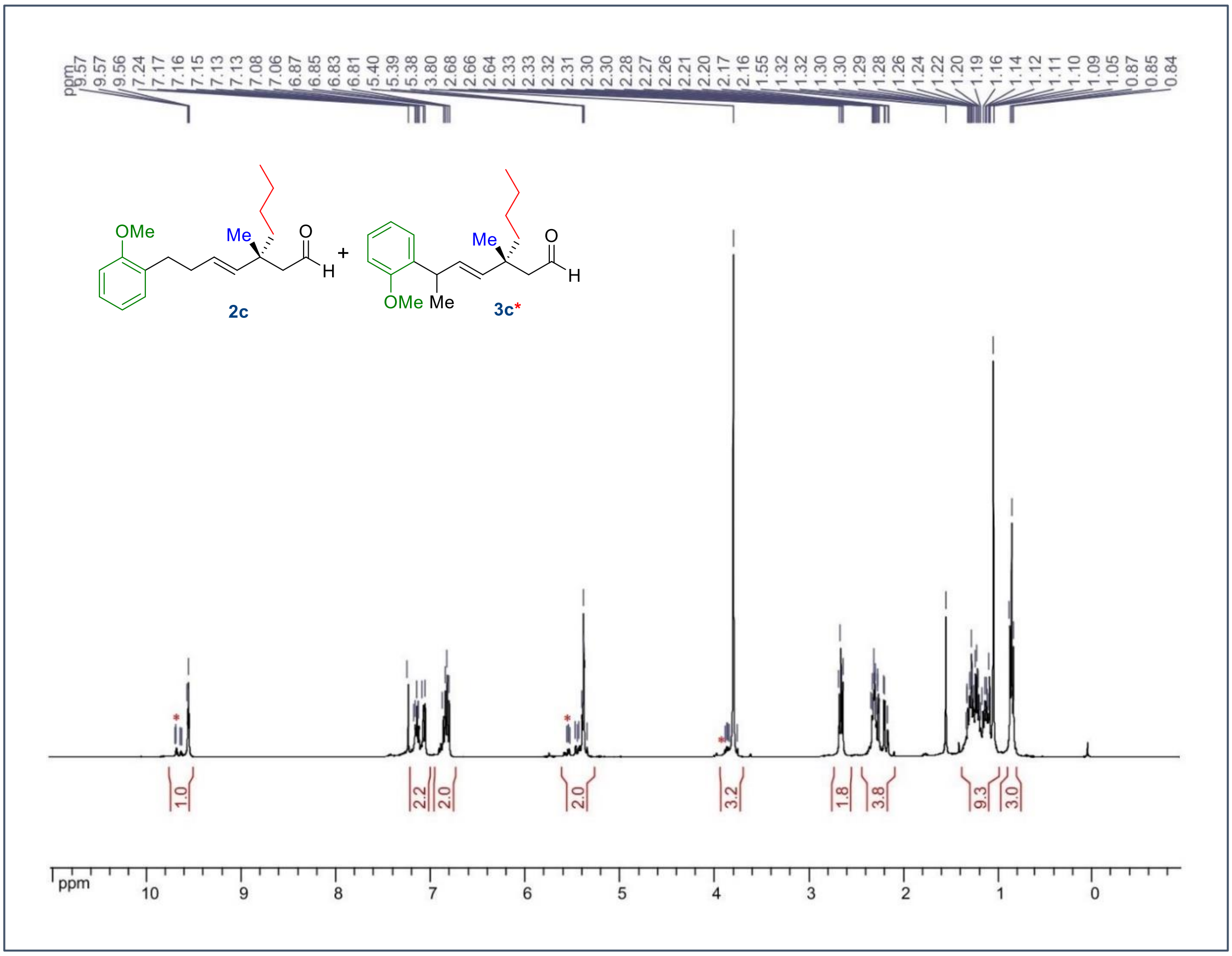

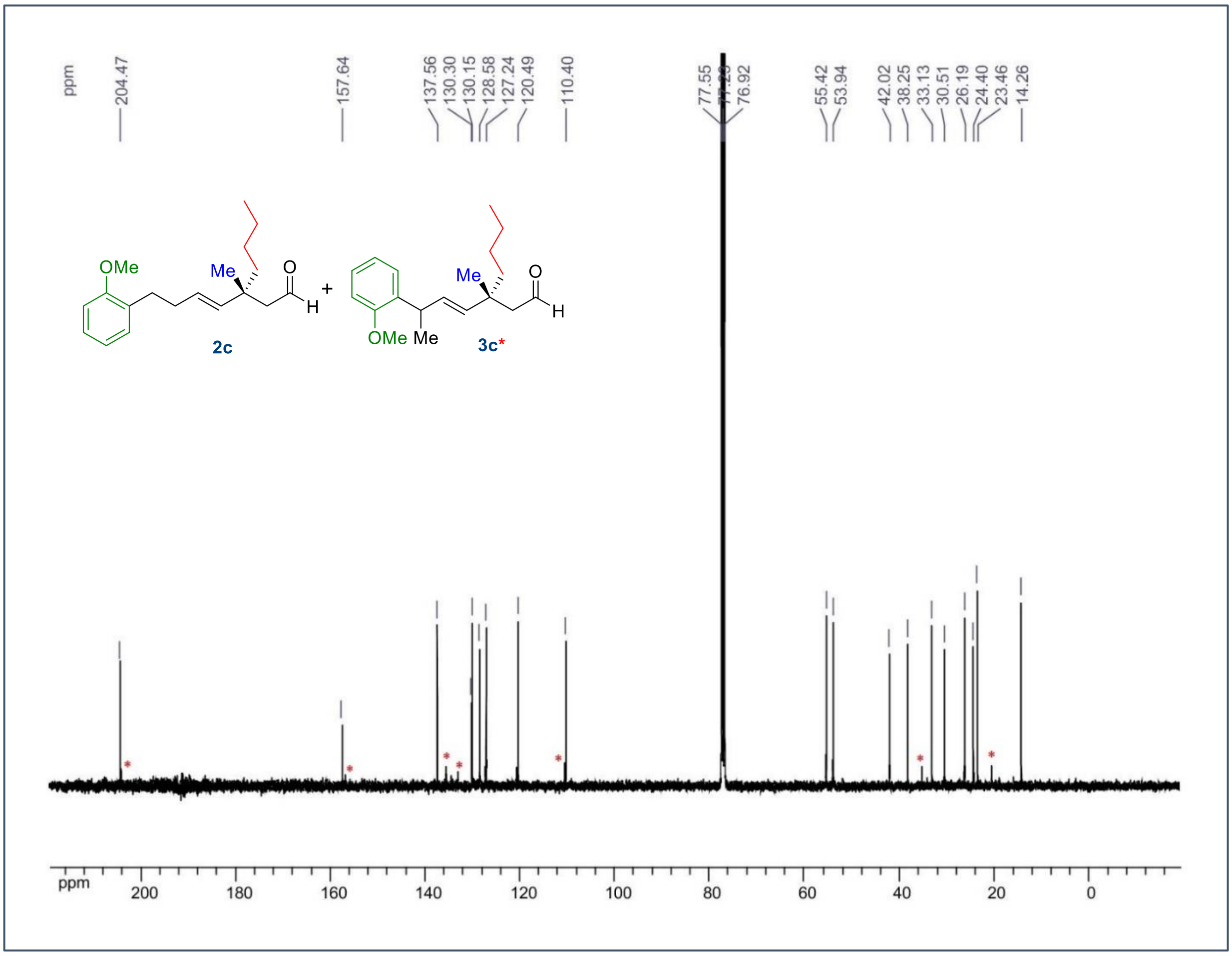

Supplementary Figure 45. ^{1}H and ^{13}C NMR spectra of compound **2c**

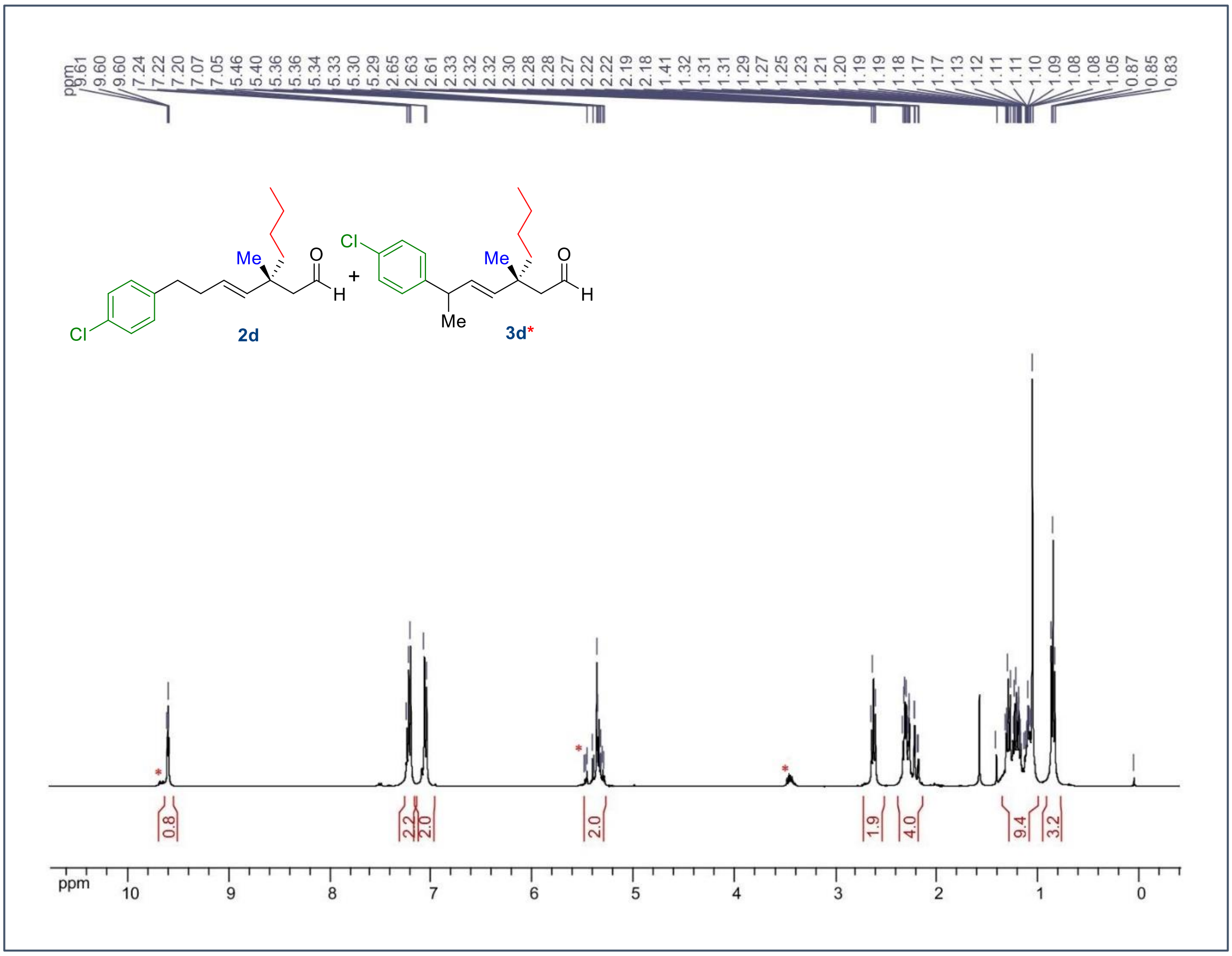

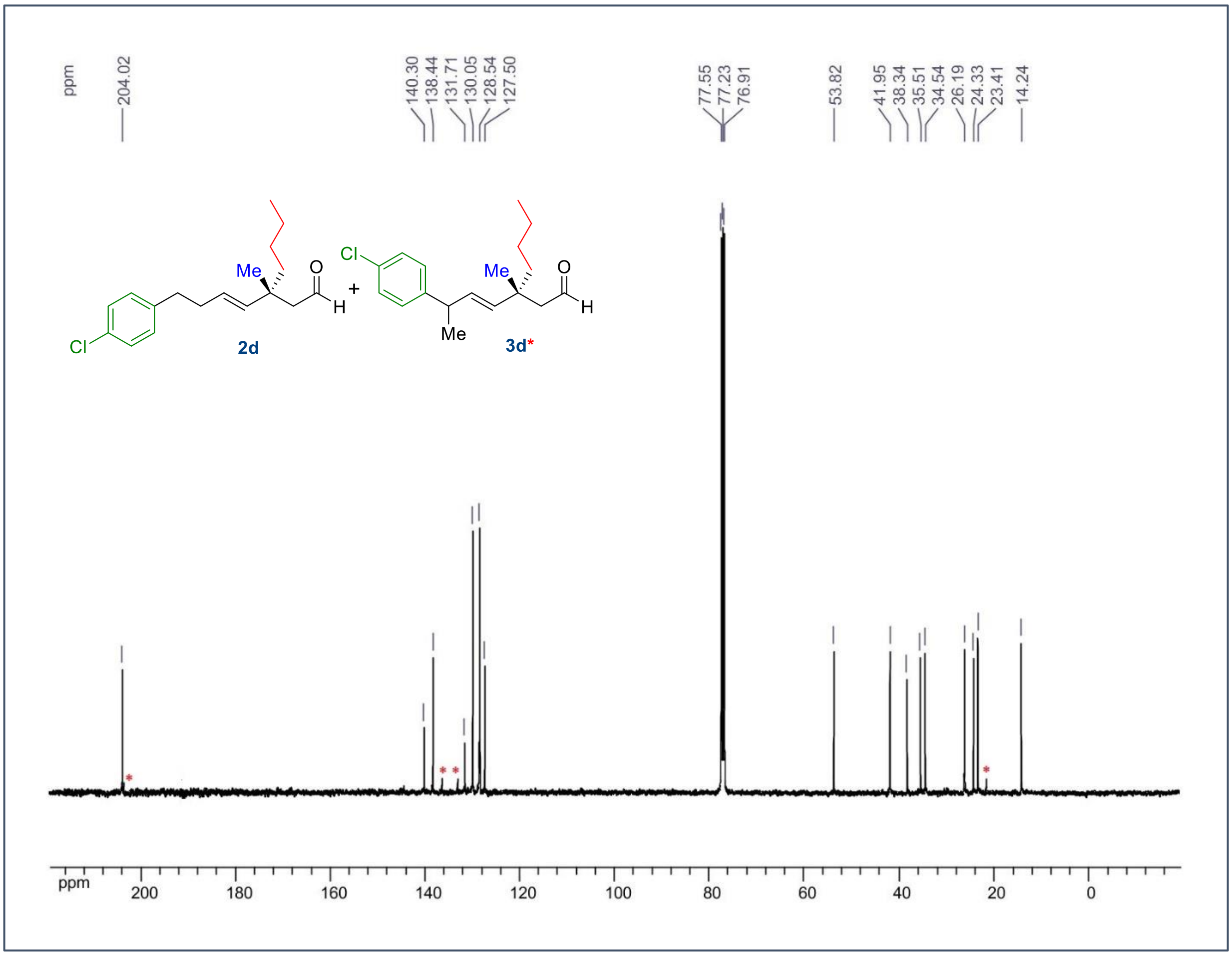

Supplementary Figure 46. ^{1}H and ^{13}C NMR spectra of compound **2d**

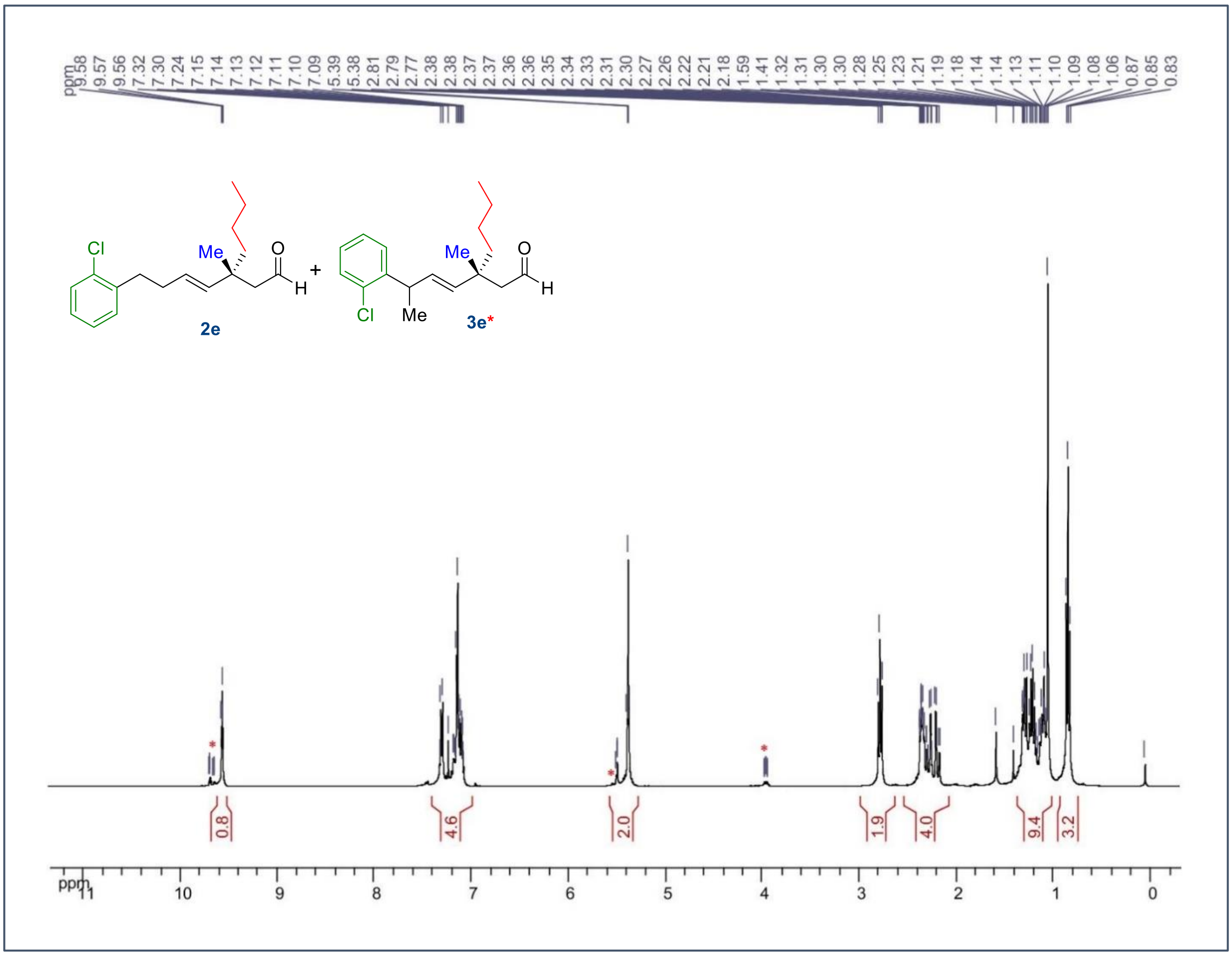

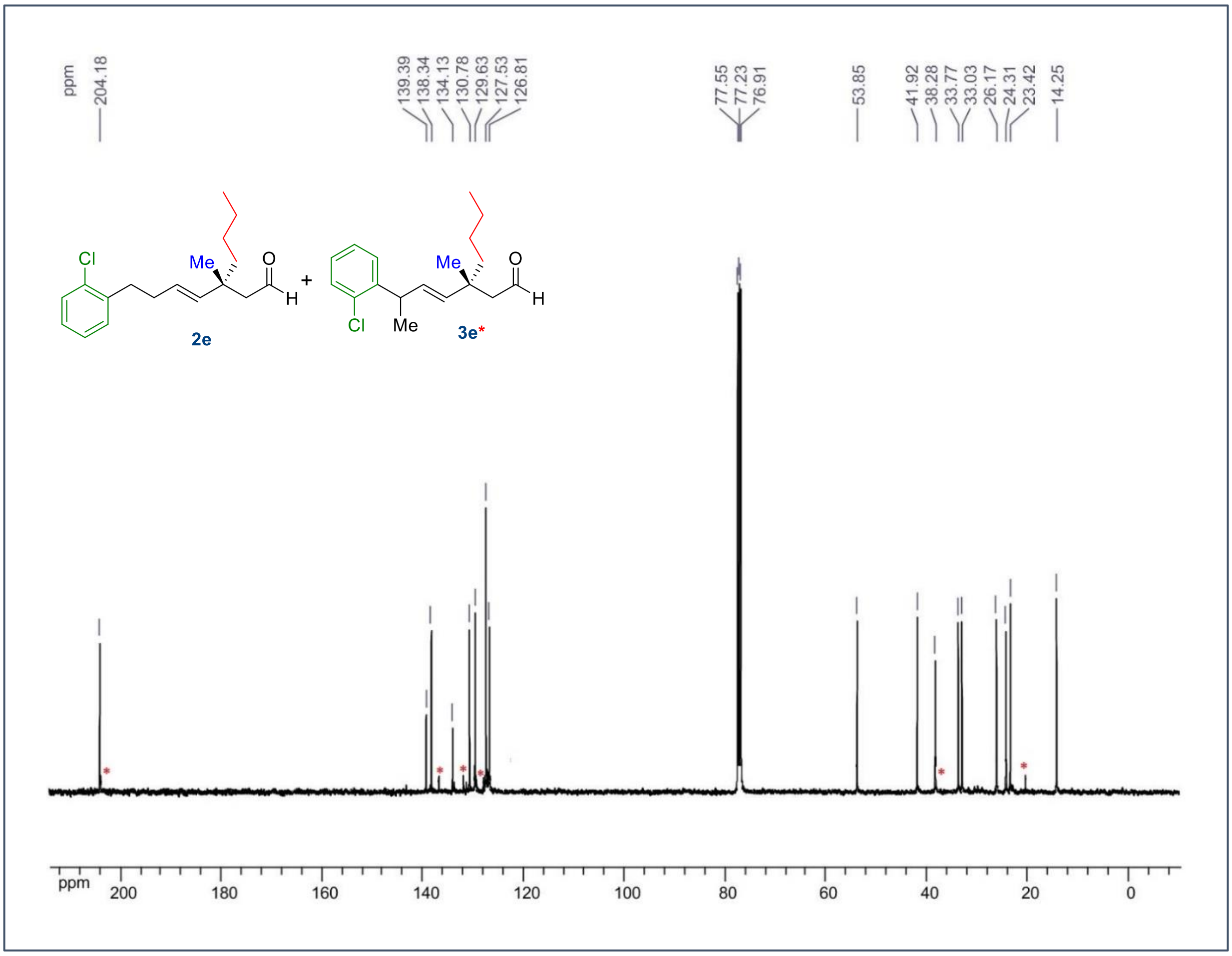

Supplementary Figure 47. ^{1}H and ^{13}C NMR spectra of compound **2e**

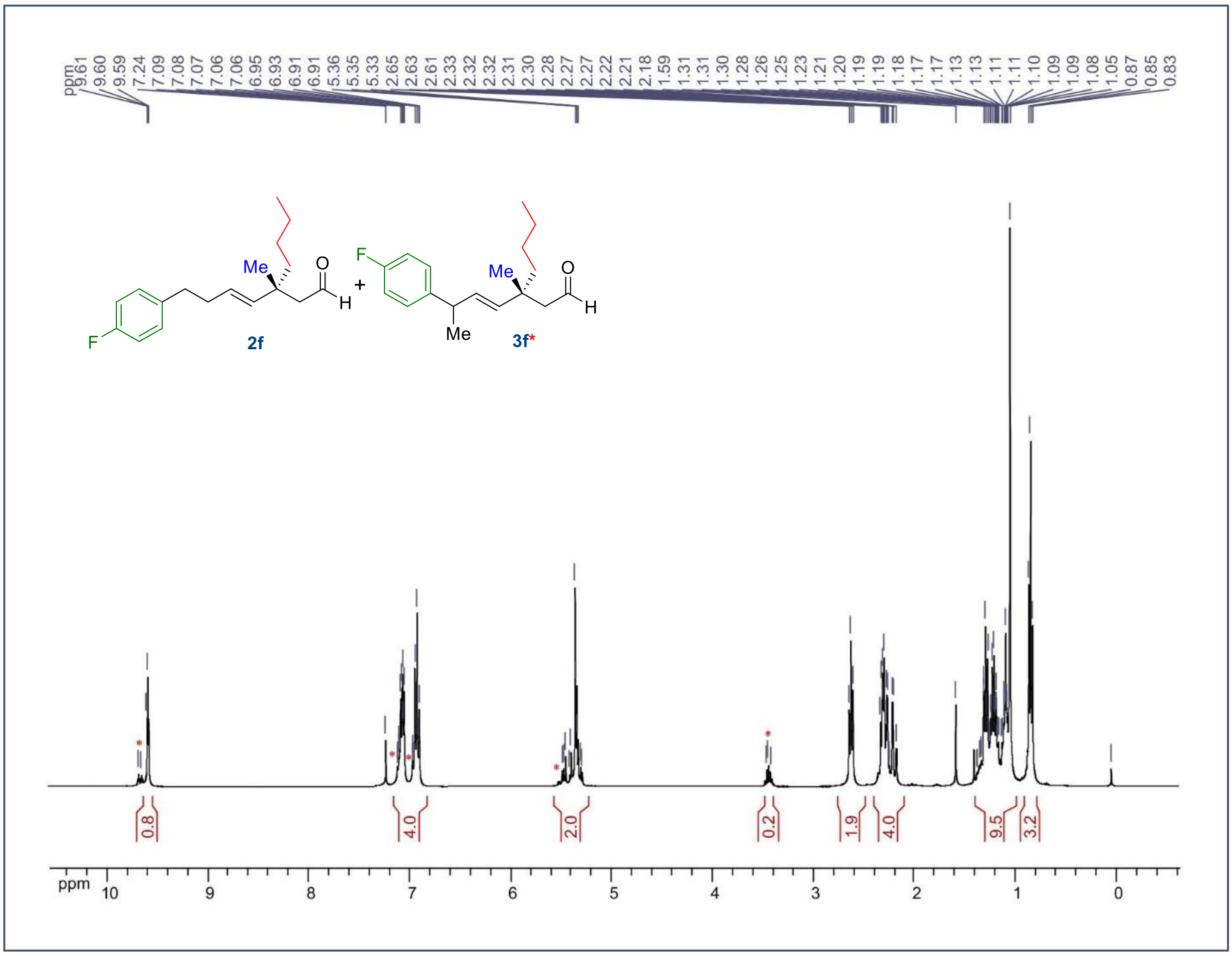

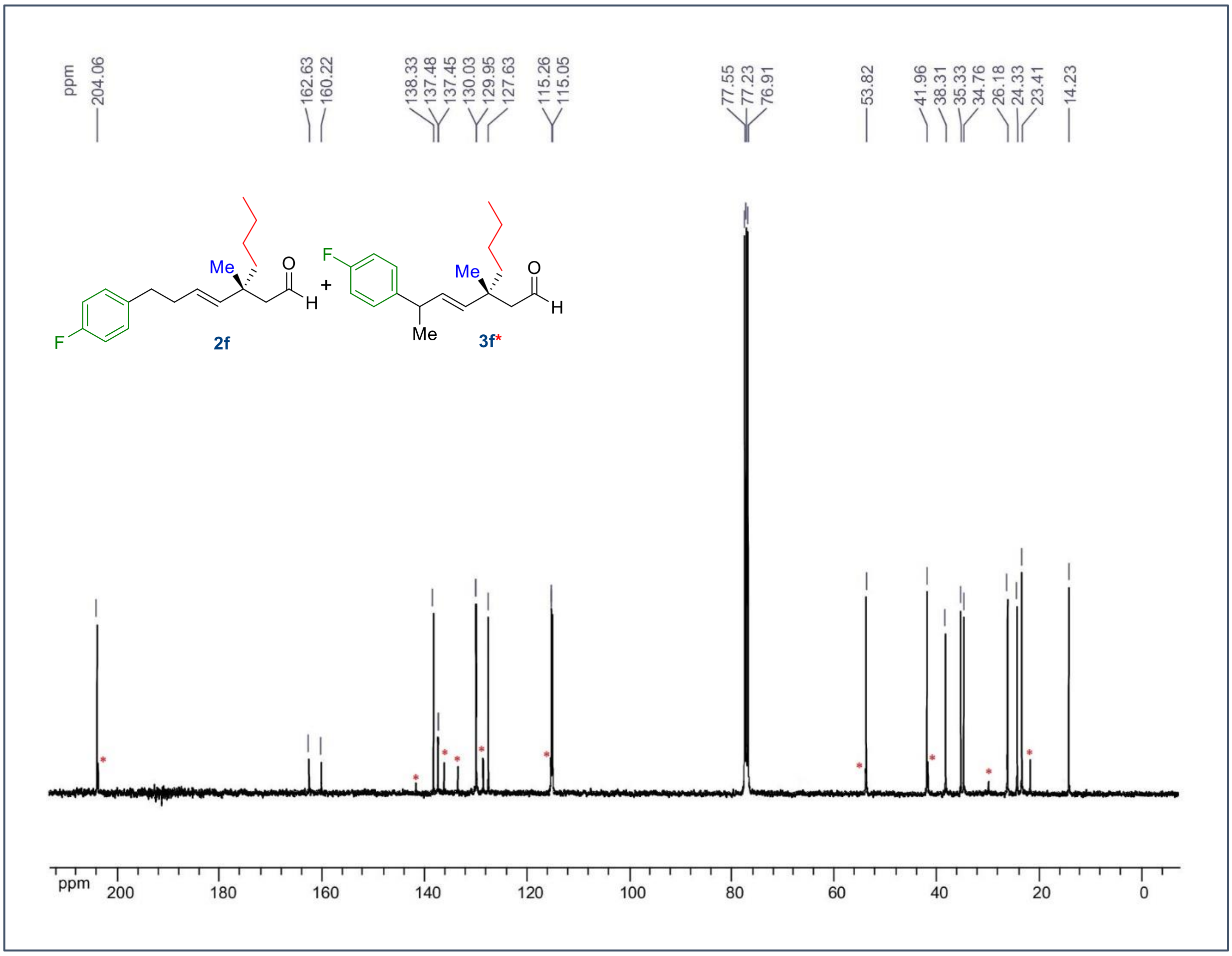

Supplementary Figure 48. ^{1}H and ^{13}C NMR spectra of compound **2f**

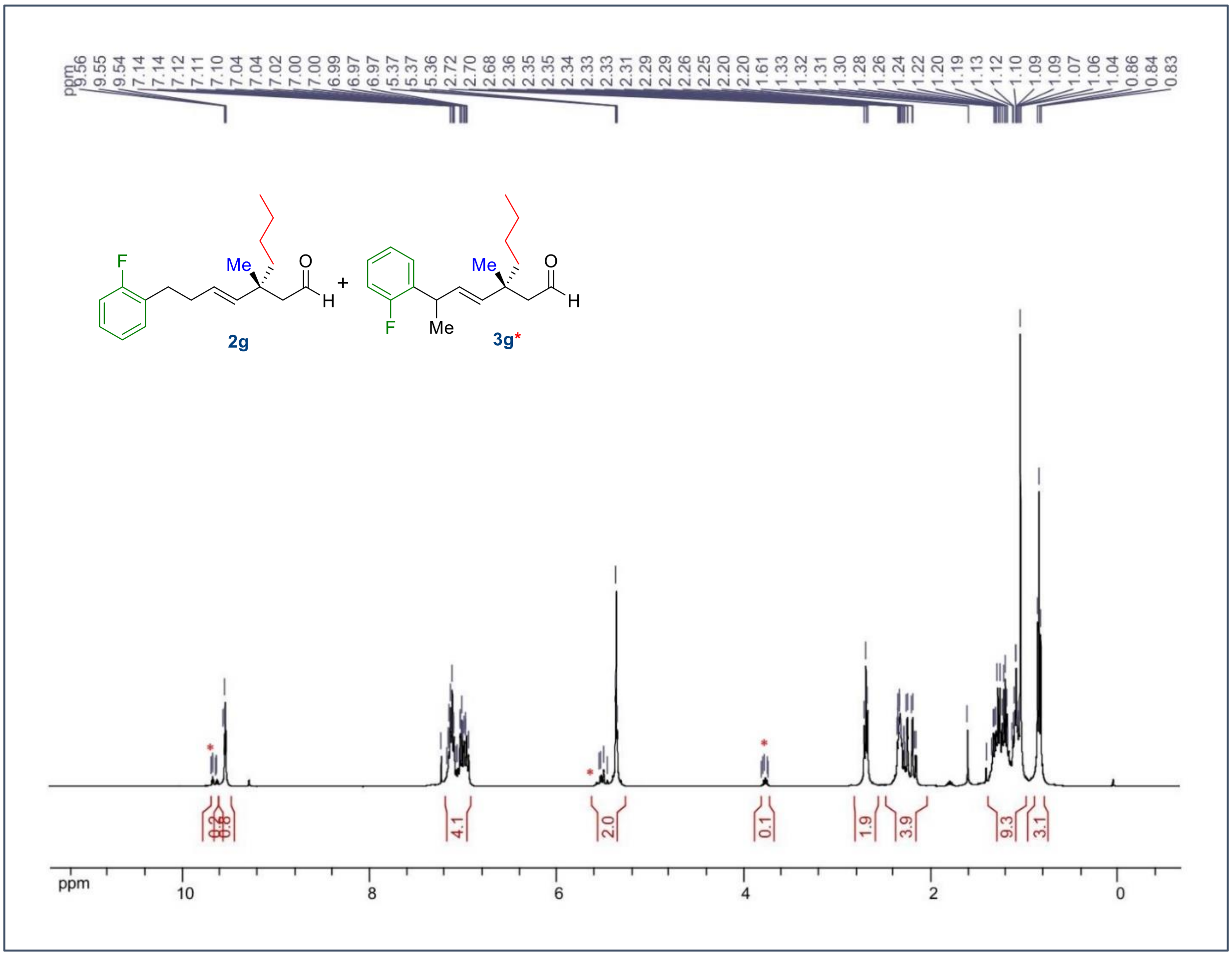

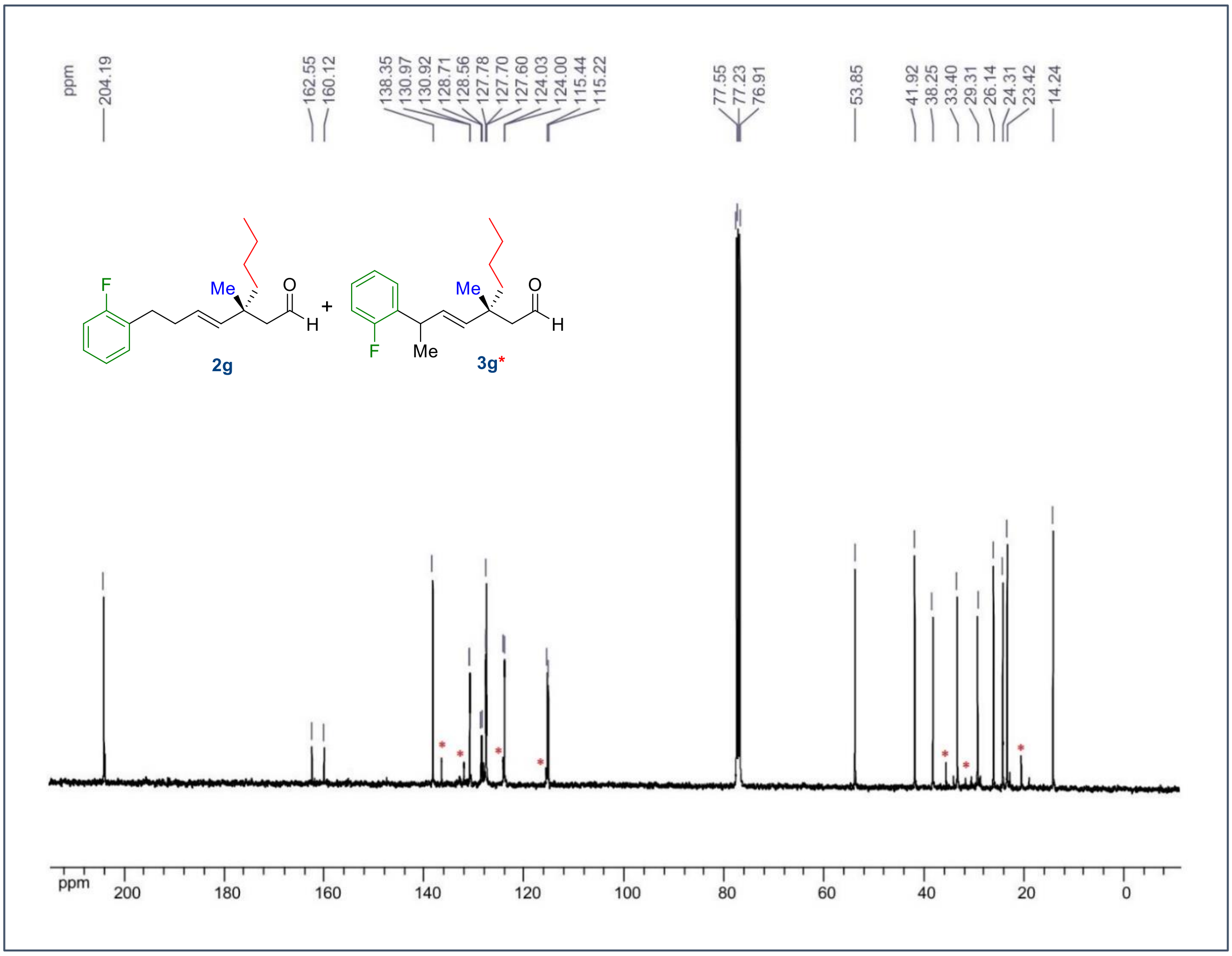

Supplementary Figure 49. ^{1}H and ^{13}C NMR spectra of compound **2g**

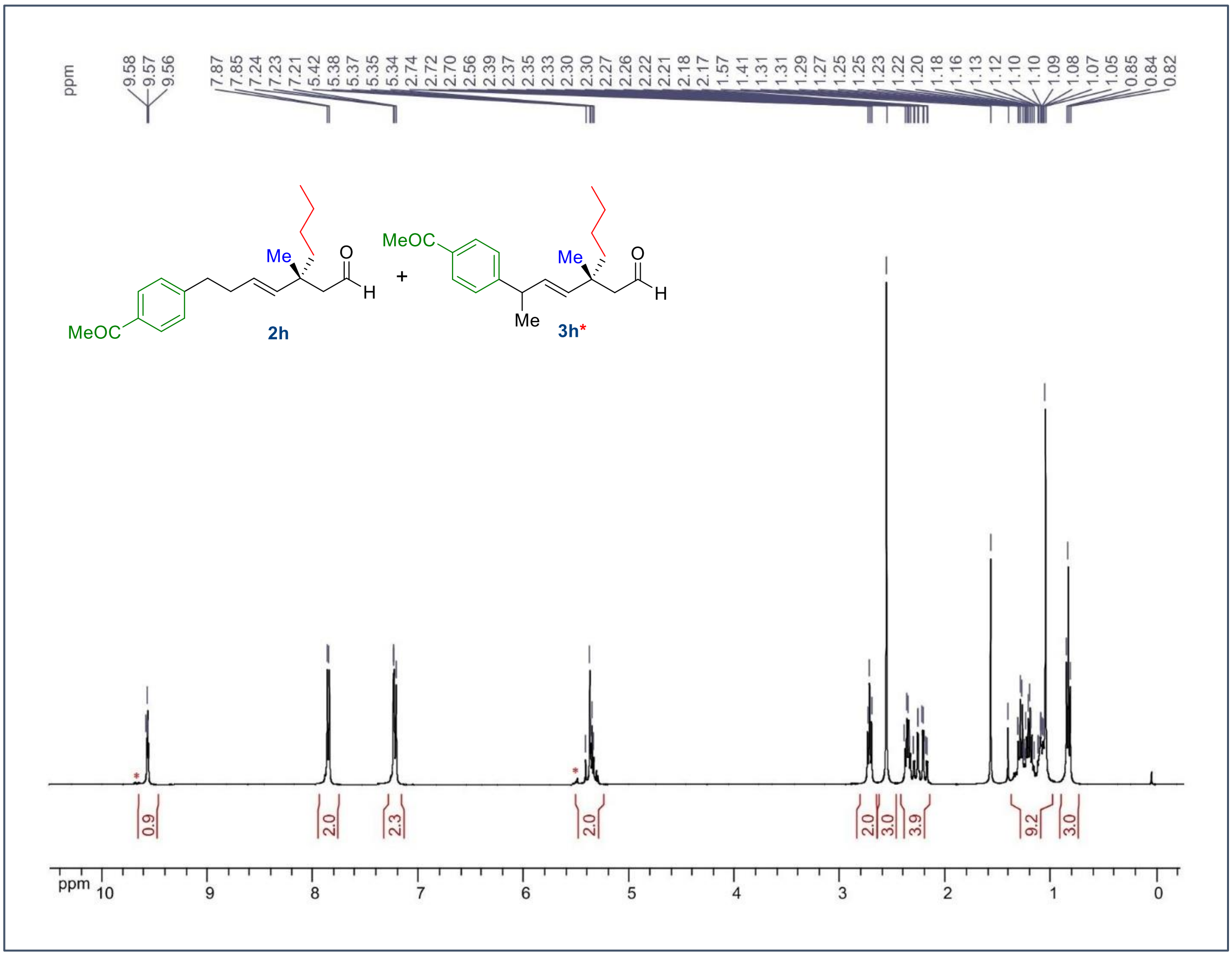

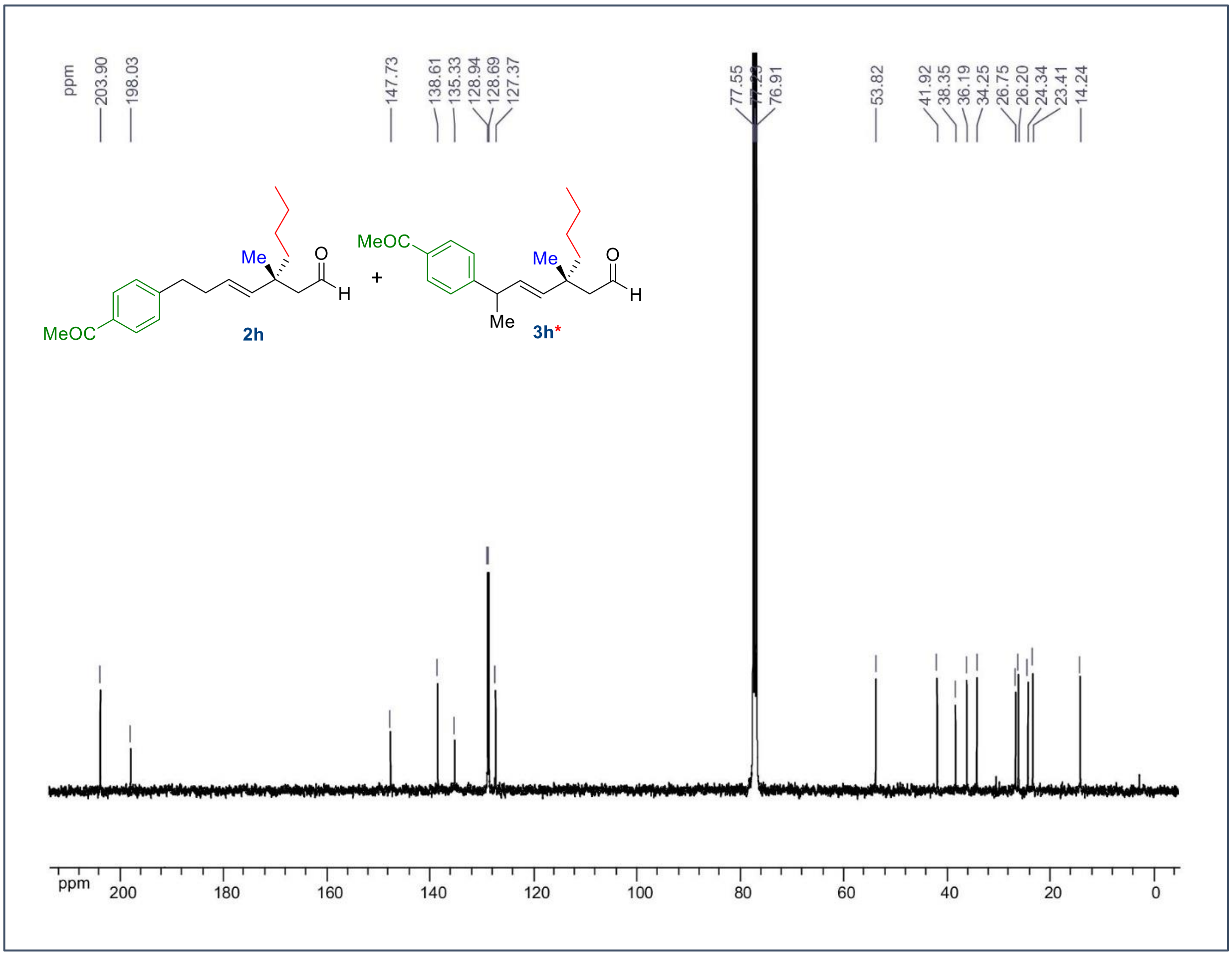

Supplementary Figure 50. ^{1}H and ^{13}C NMR spectra of compound **2h**

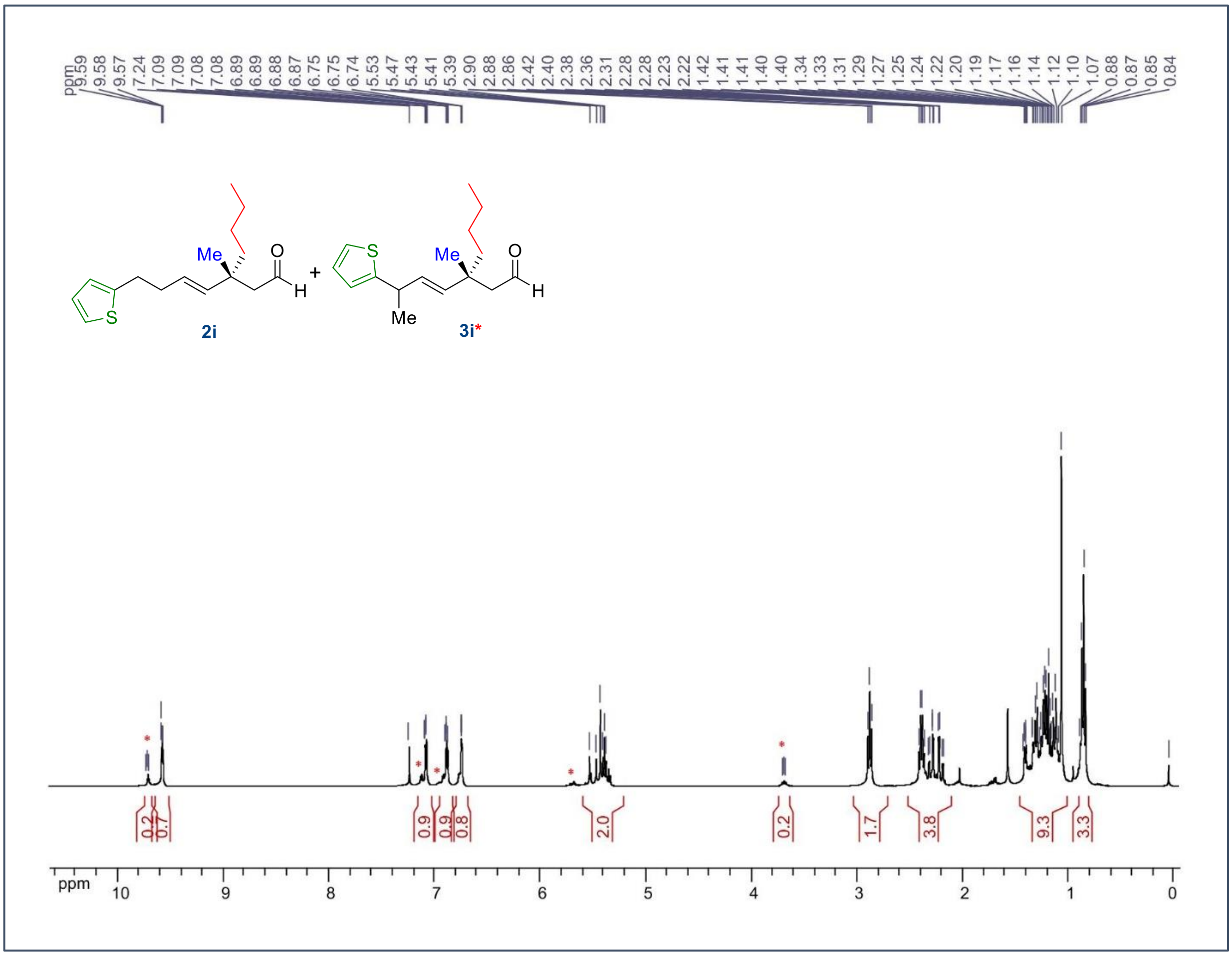

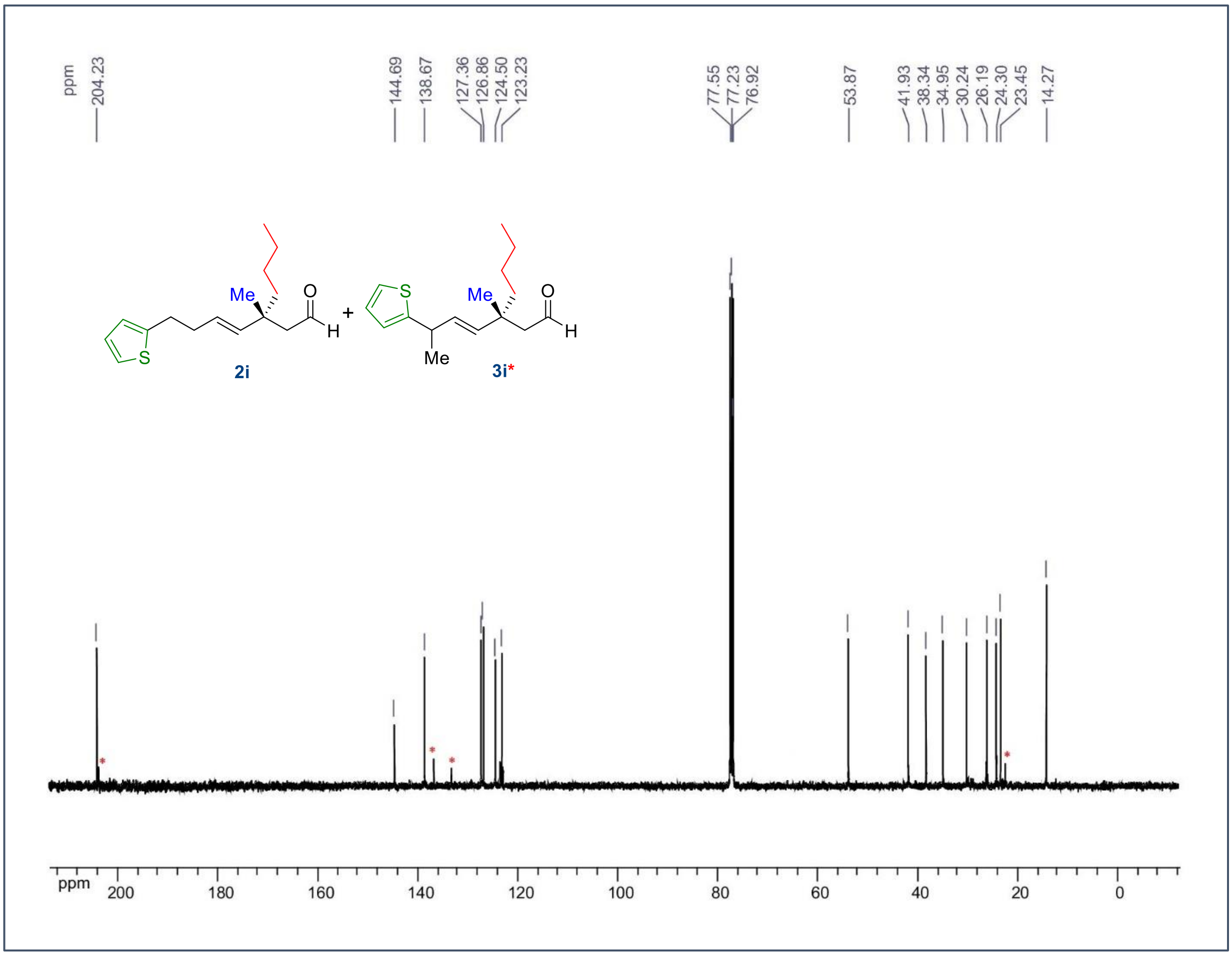

Supplementary Figure 51. ^{1}H and ^{13}C NMR spectra of compound **2i**

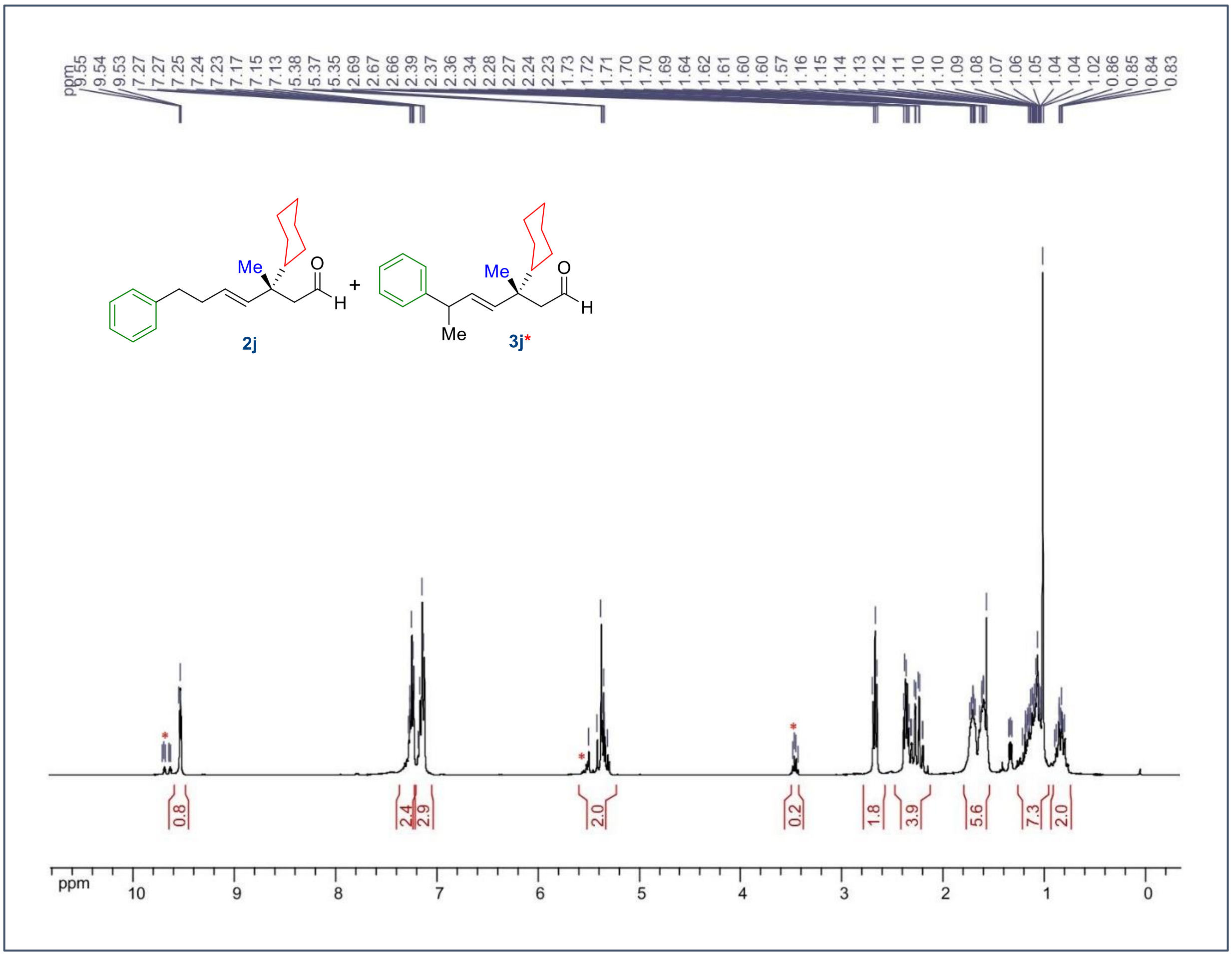

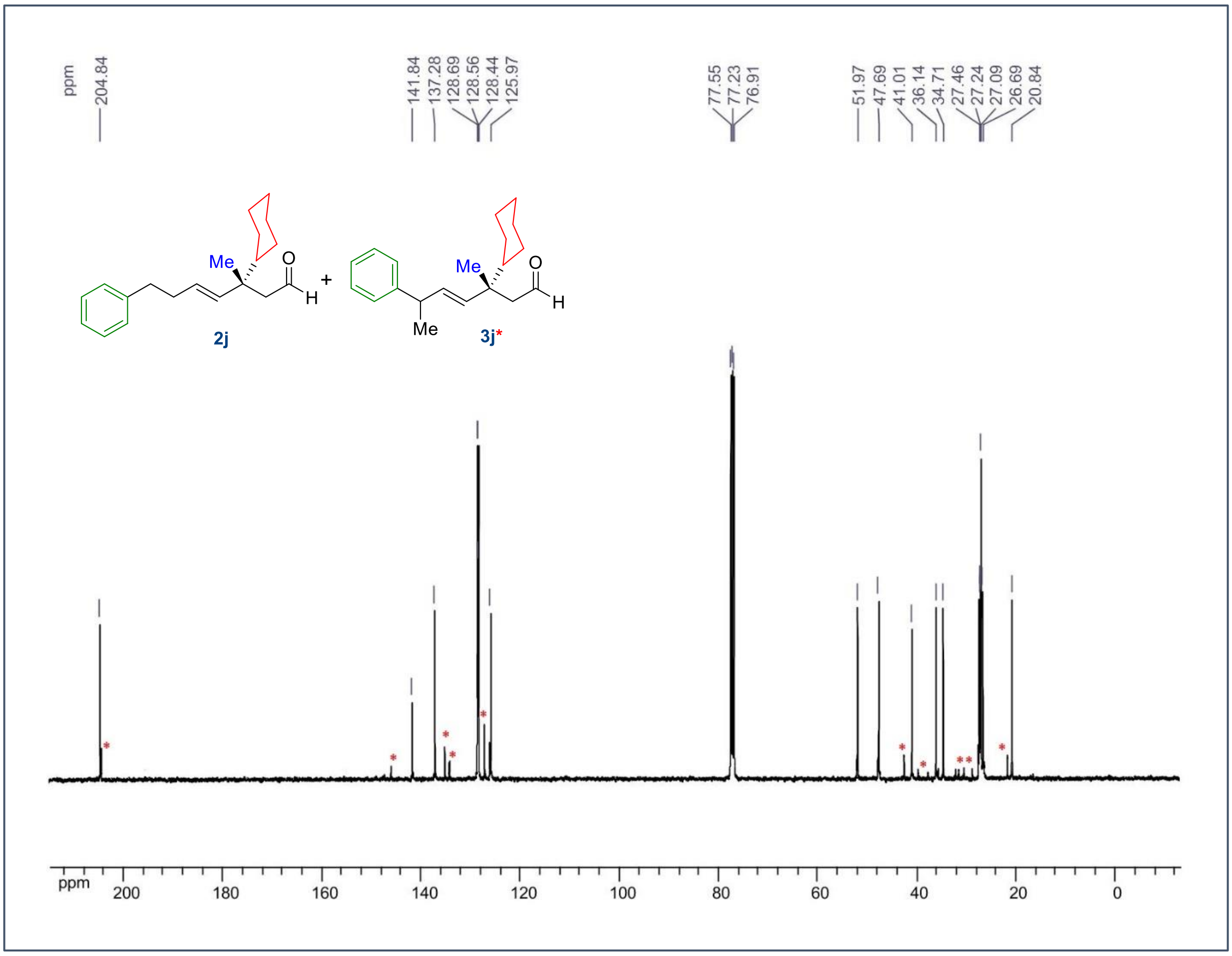

Supplementary Figure 52. ^{1}H and ^{13}C NMR spectra of compound **2j**

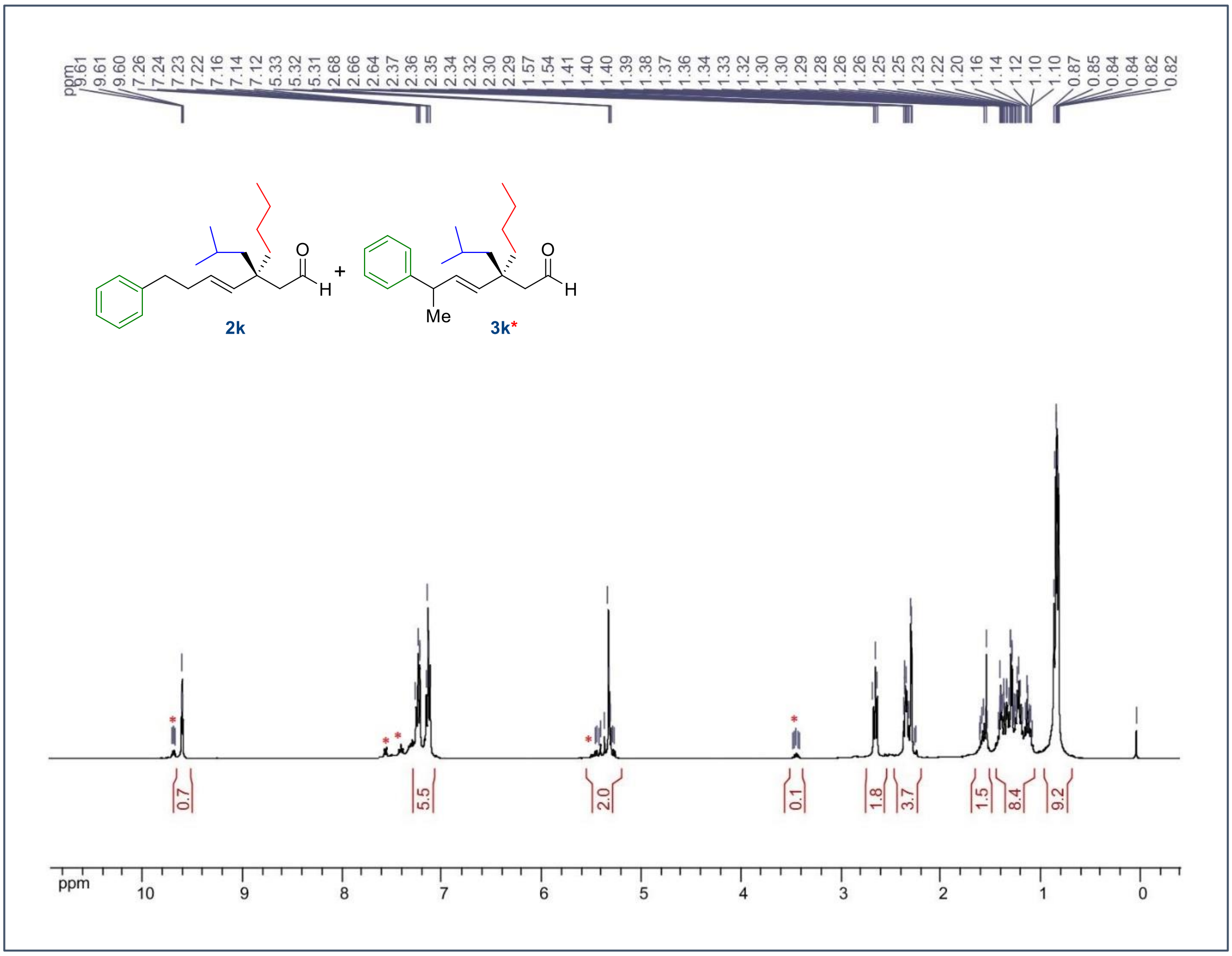

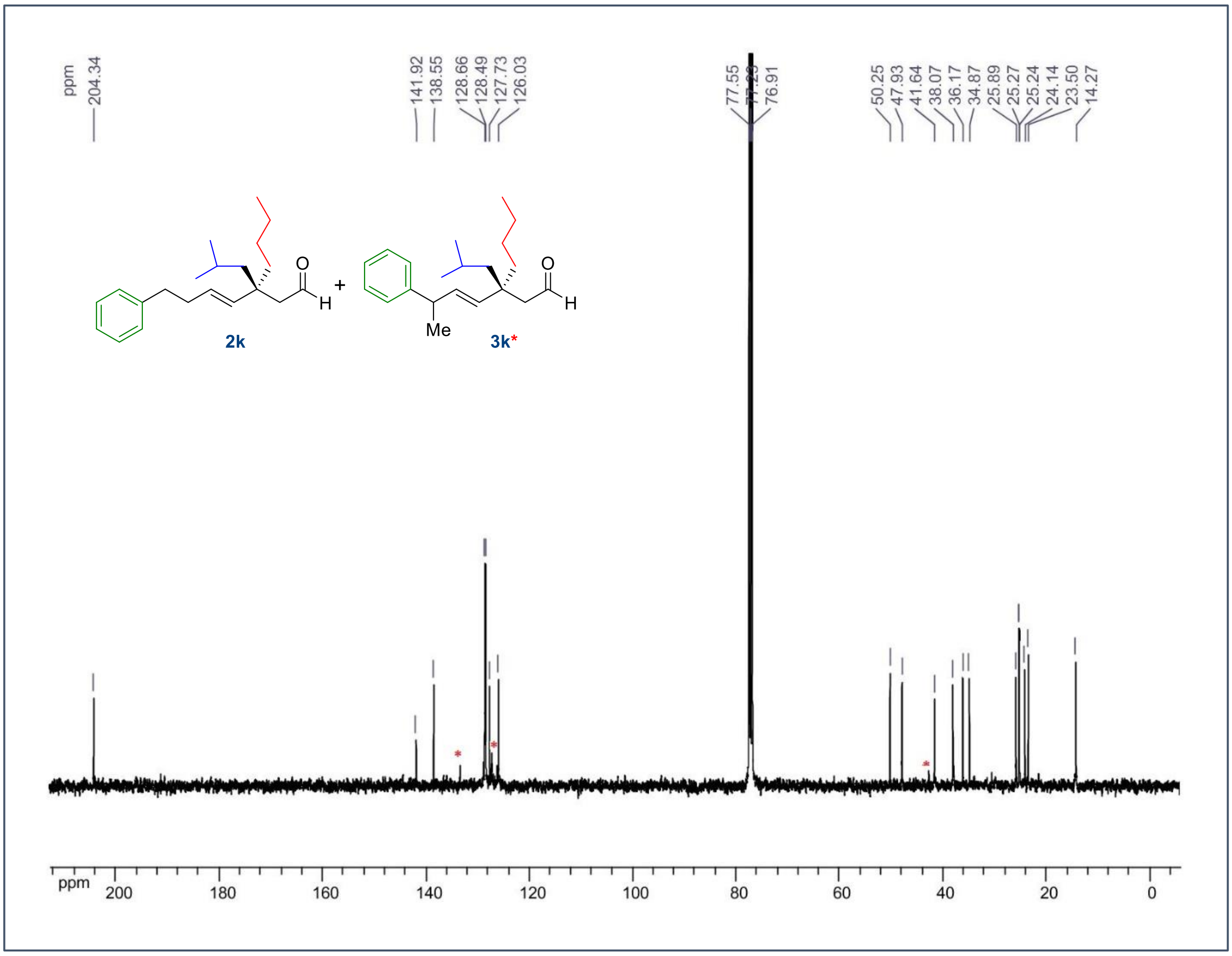

Supplementary Figure 53. ^{1}H and ^{13}C NMR spectra of compound **2k**

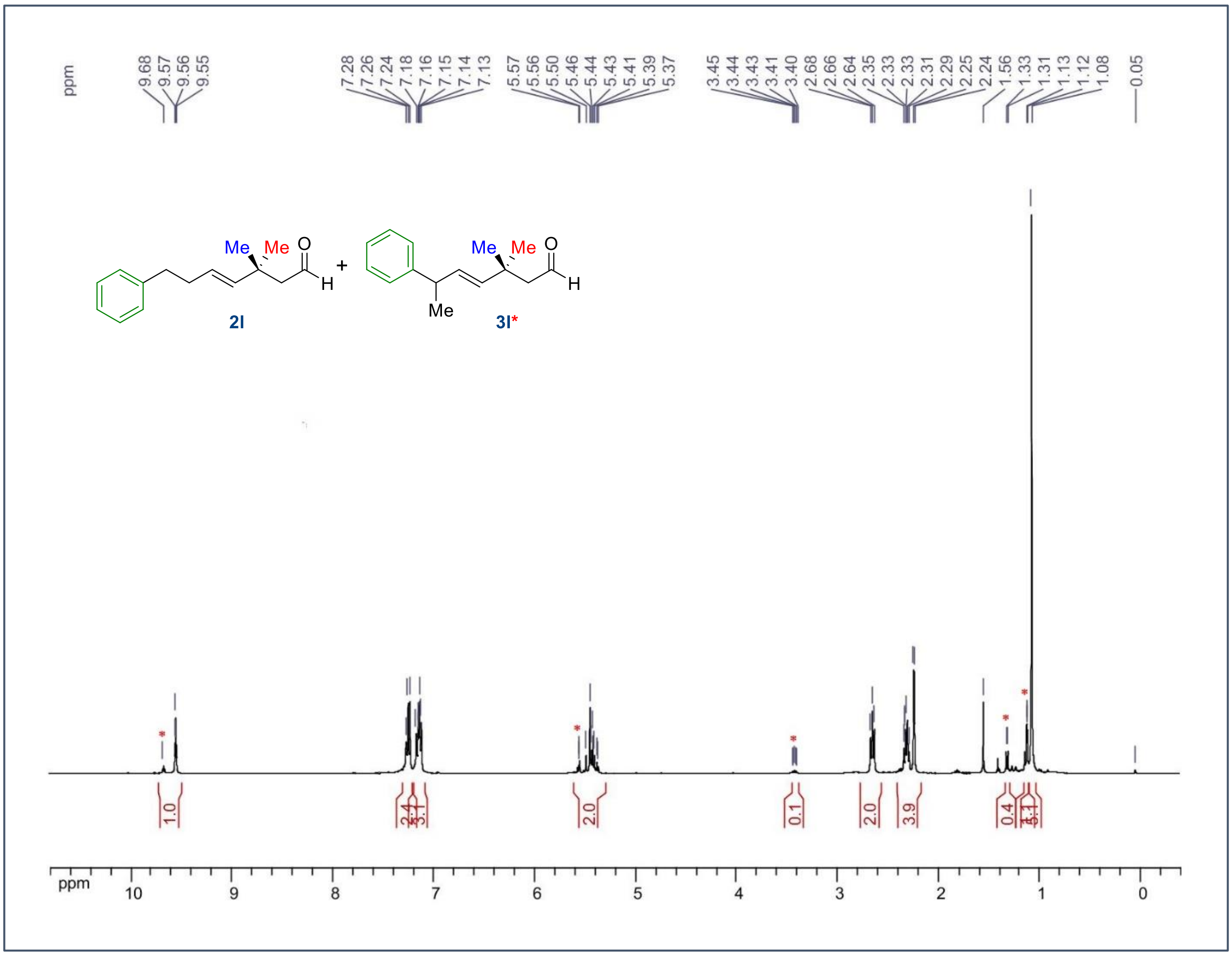

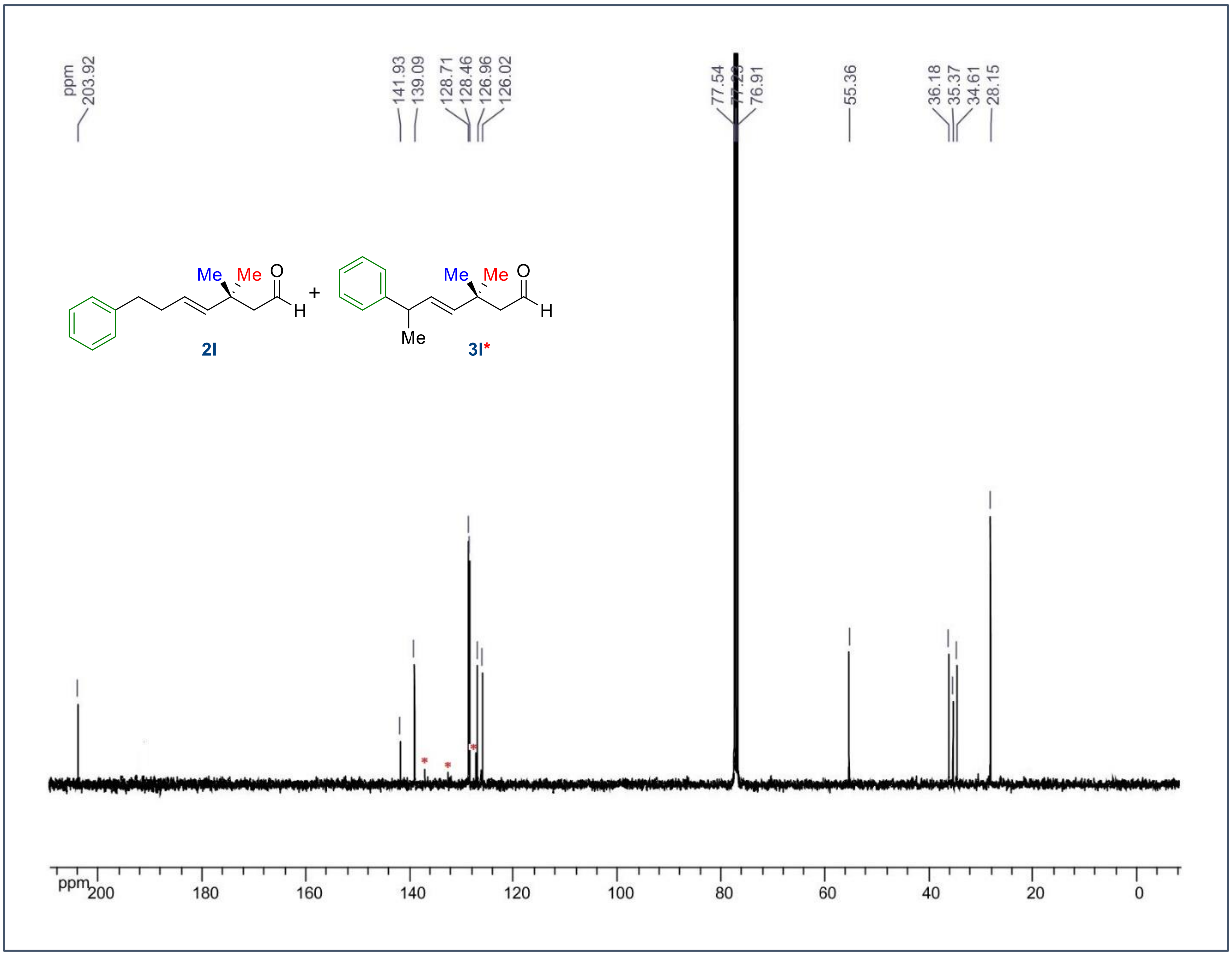

Supplementary Figure 54. ^{1}H and ^{13}C NMR spectra of compound **2l**

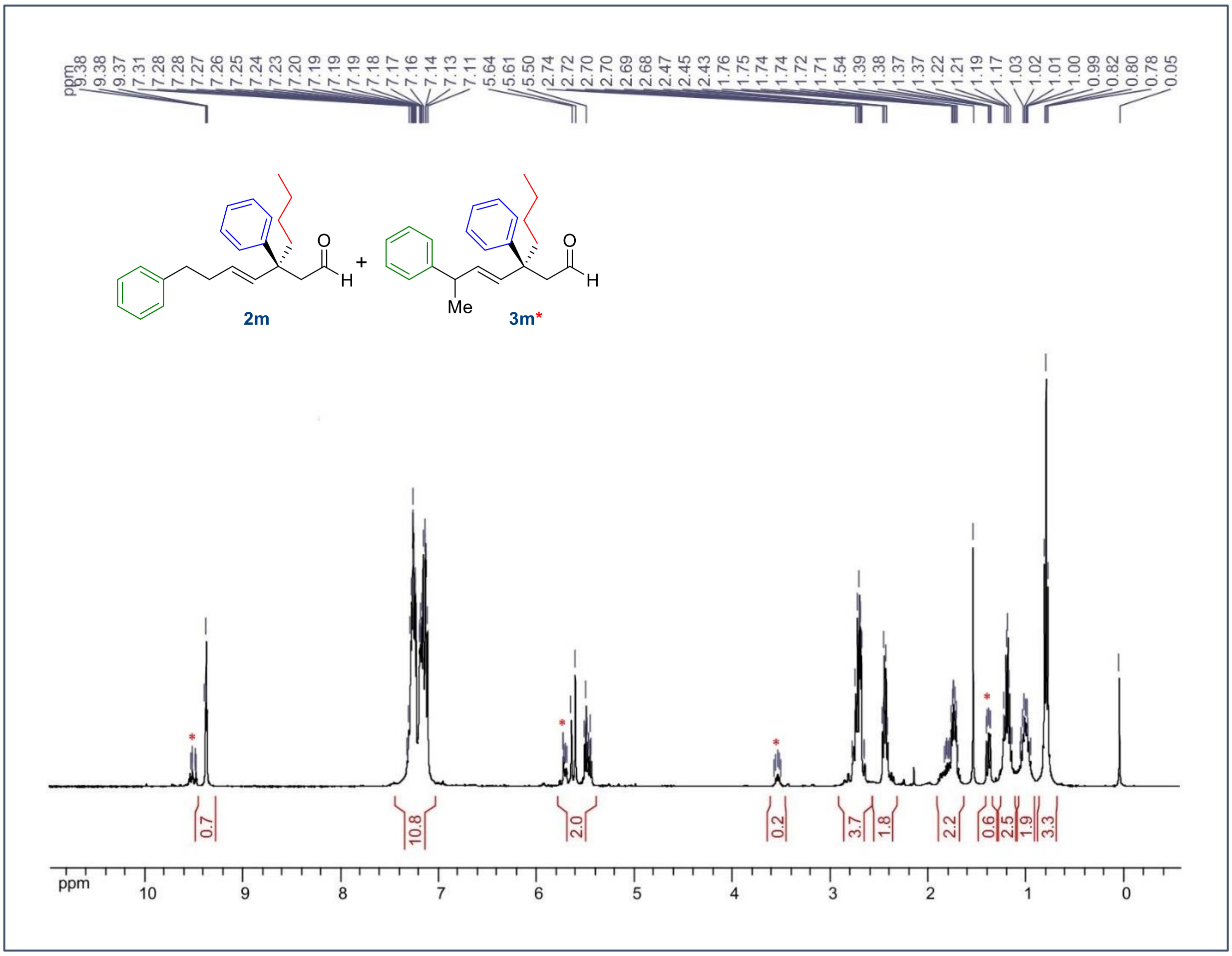

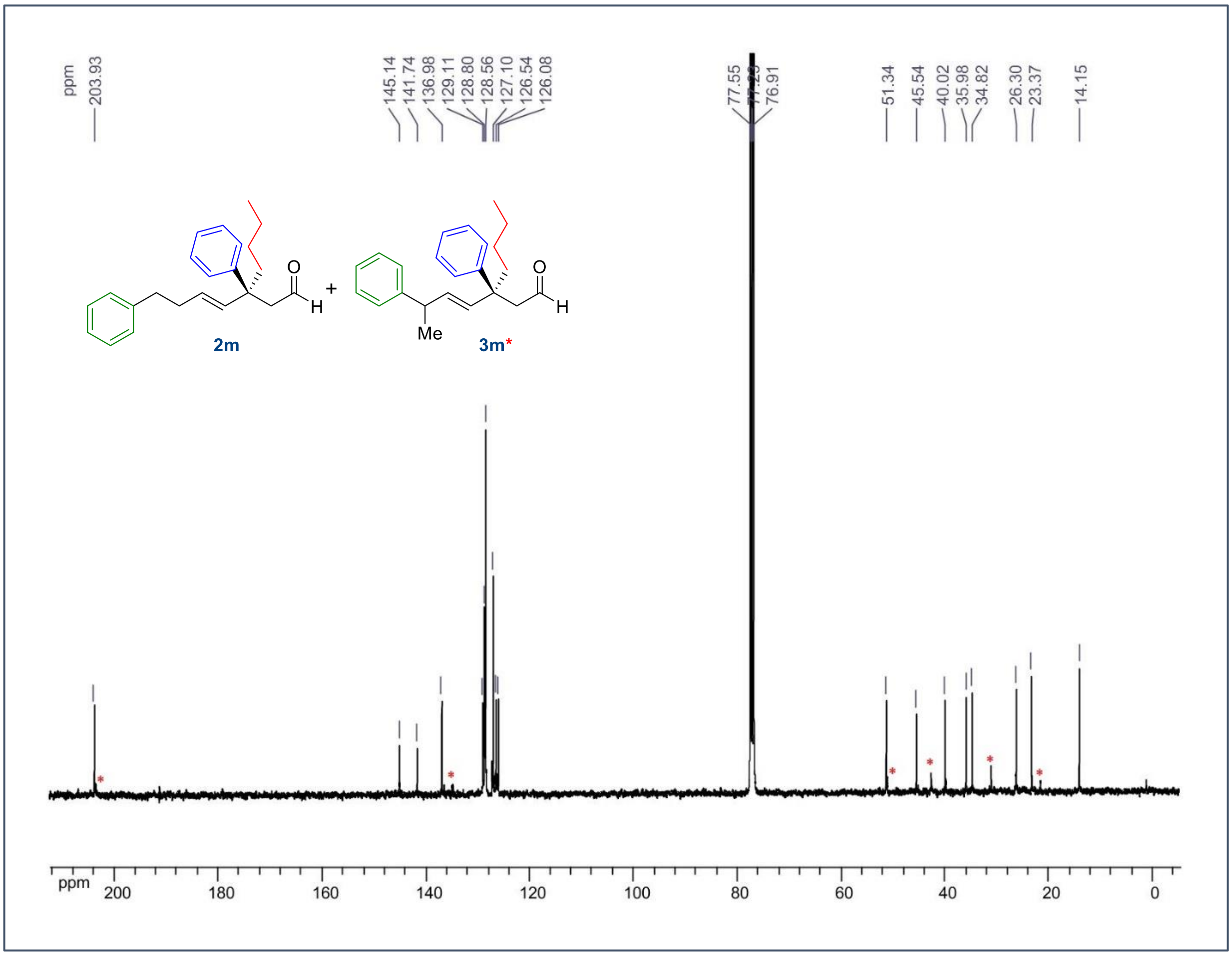

Supplementary Figure 55. ^{1}H and ^{13}C NMR spectra of compound **2m**

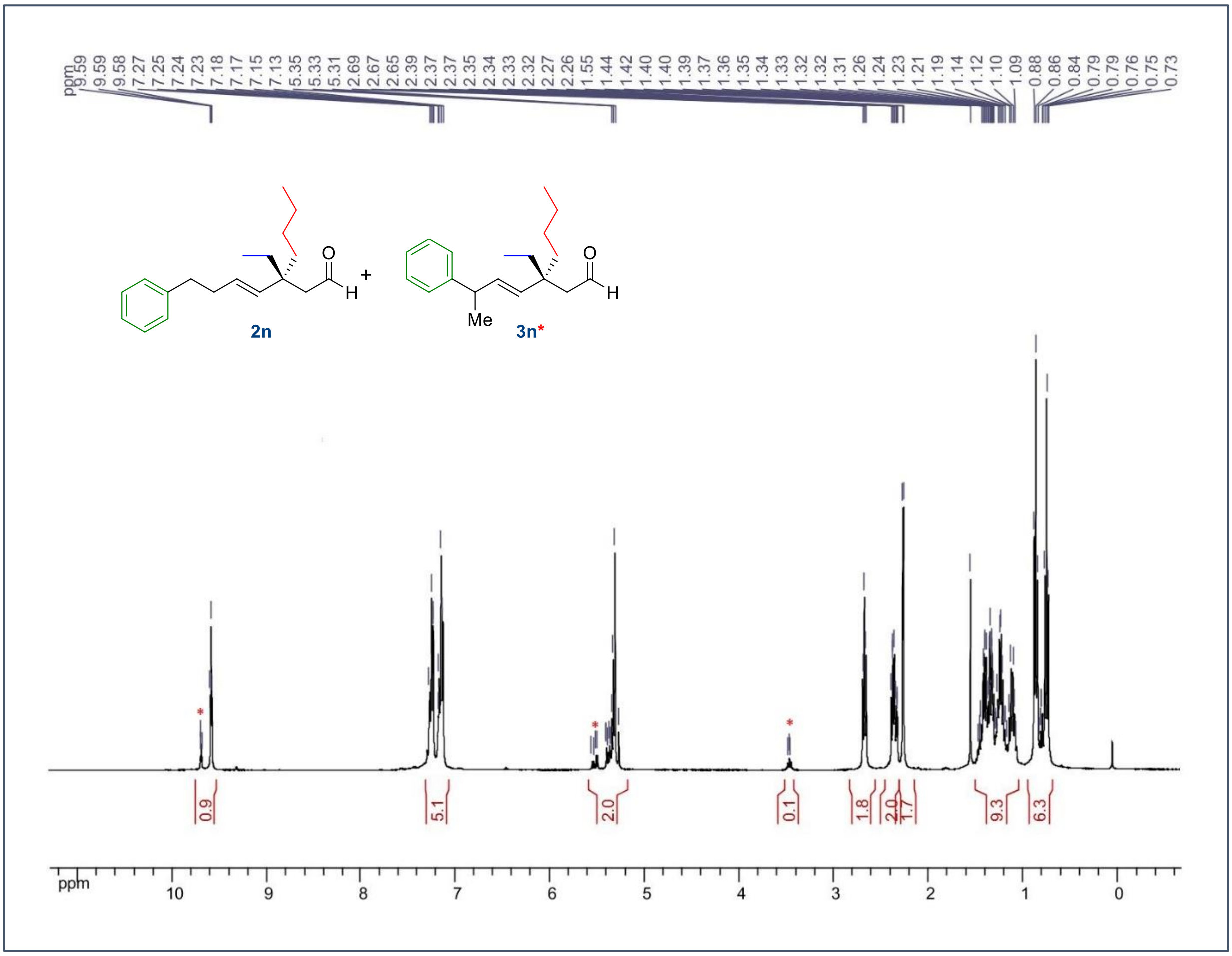

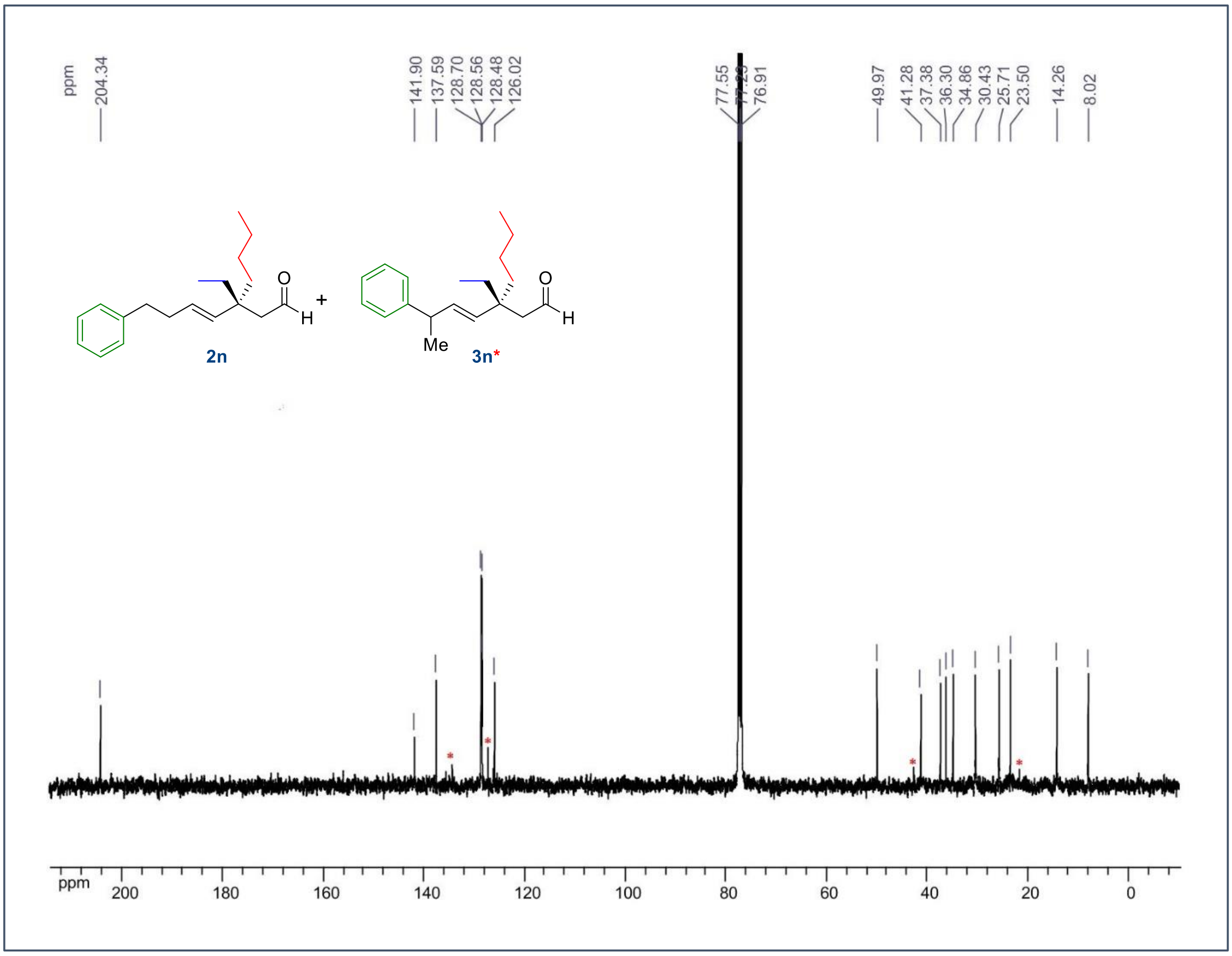

Supplementary Figure 56. ^{1}H and ^{13}C NMR spectra of compound **2n**

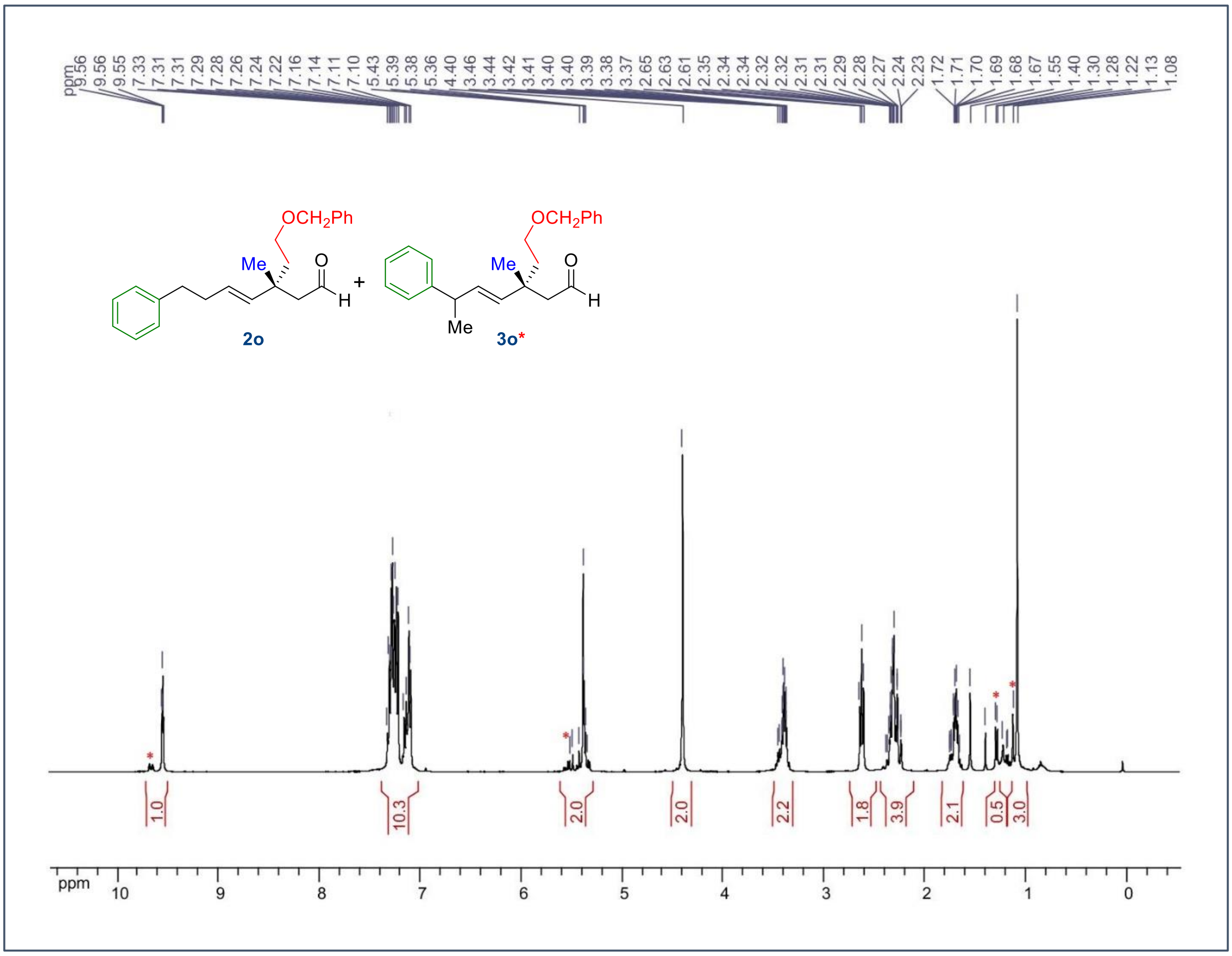

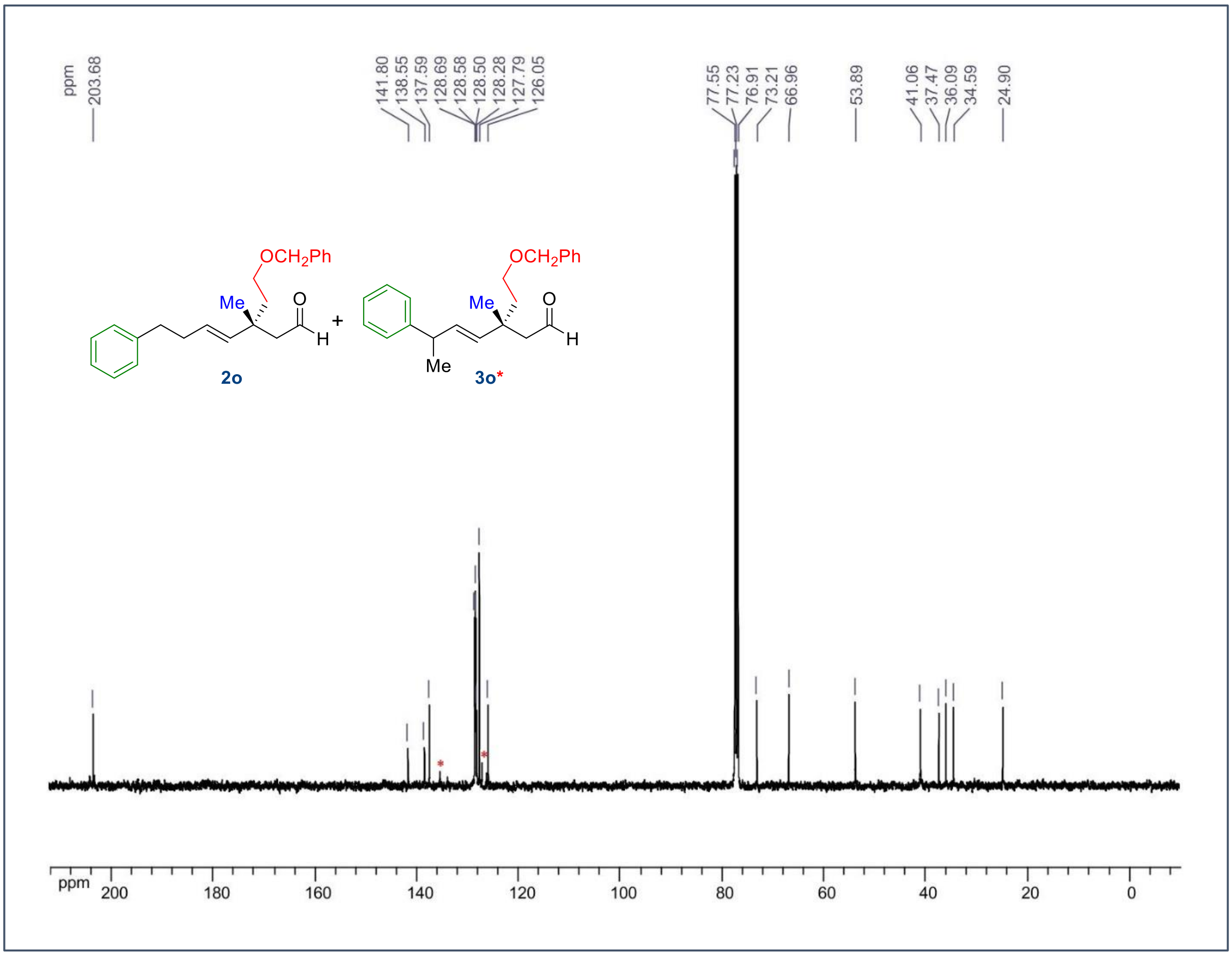

Supplementary Figure 57. ^{1}H and ^{13}C NMR spectra of compound **2o**

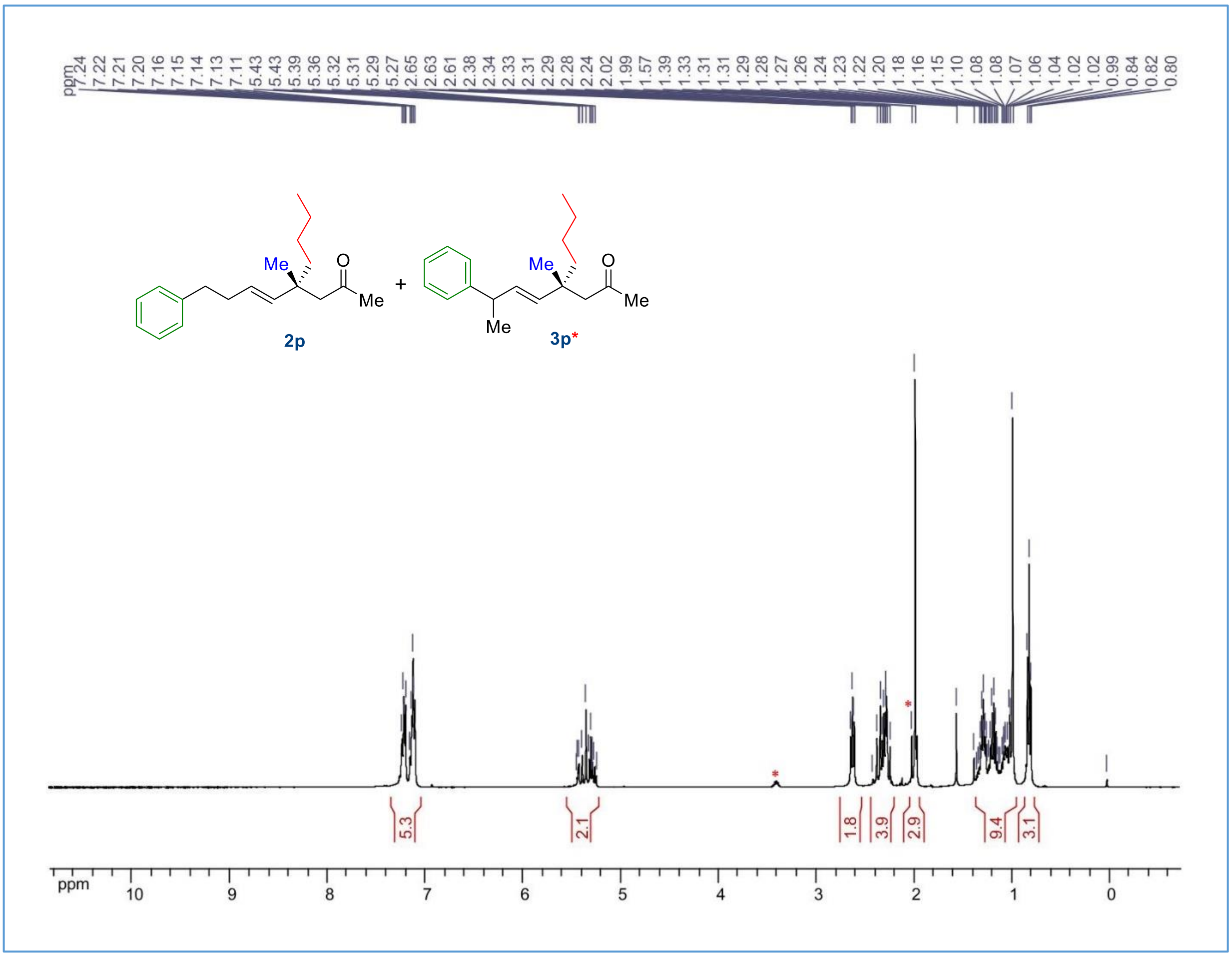

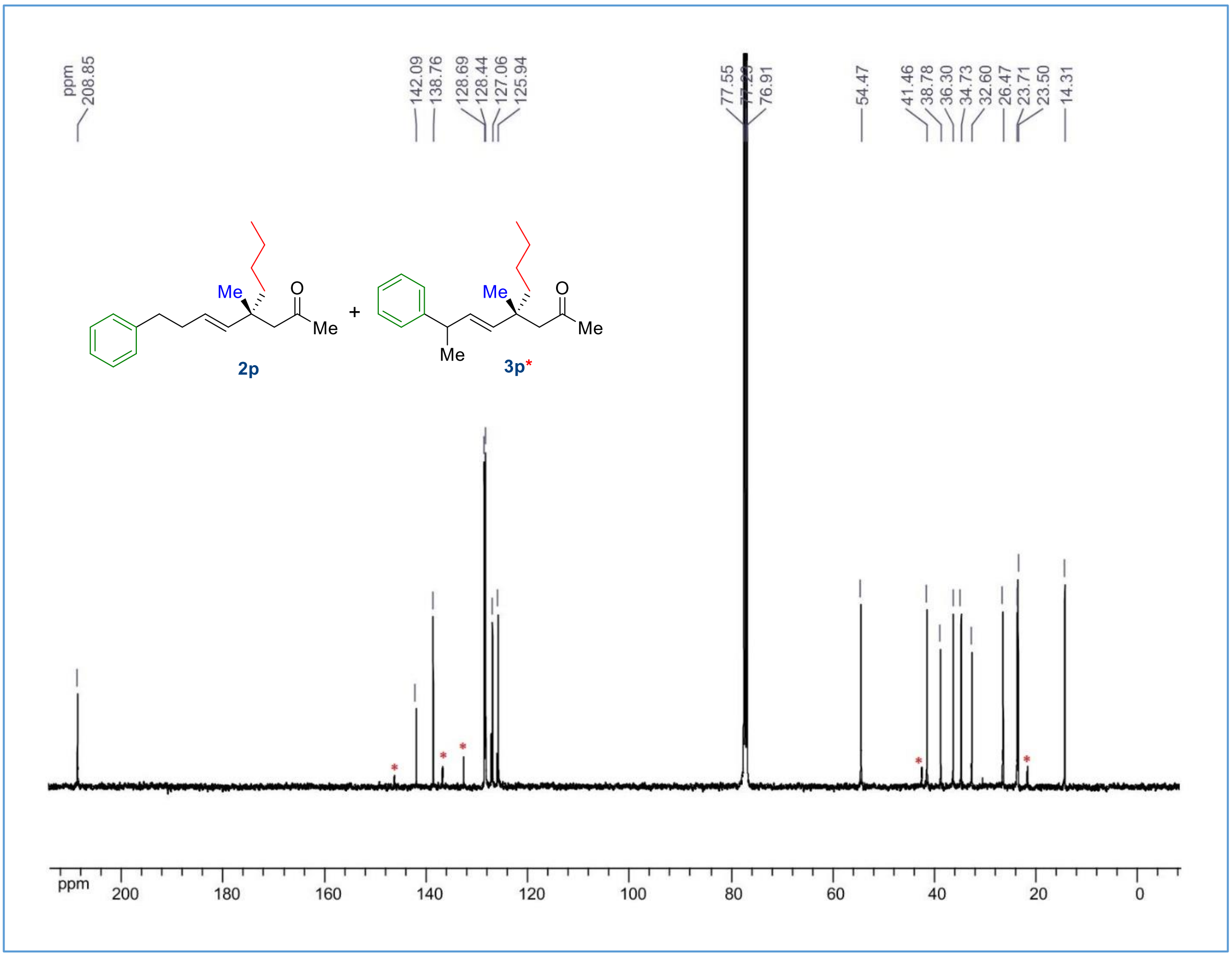

Supplementary Figure 58. ^{1}H and ^{13}C NMR spectra of compound **2p**

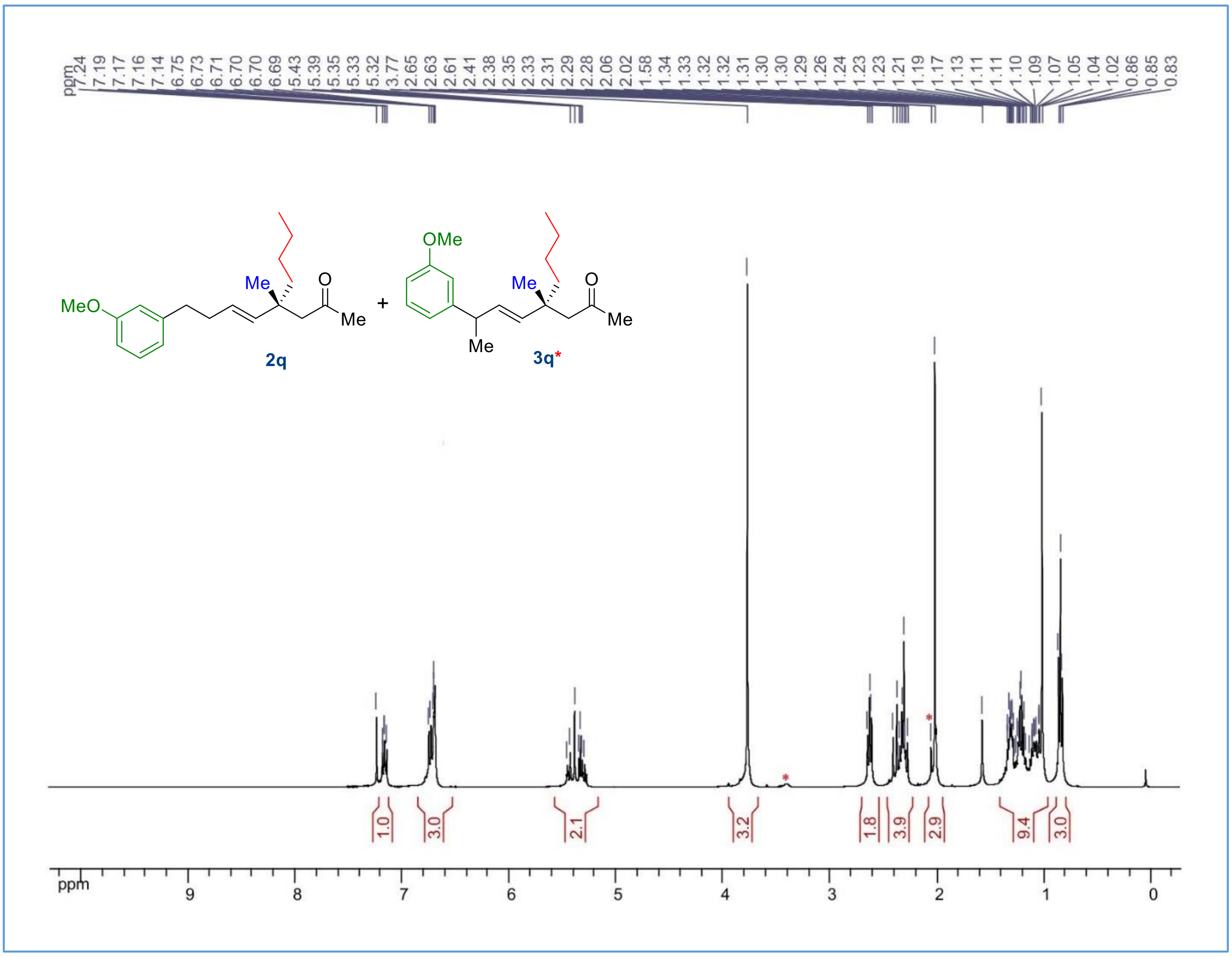

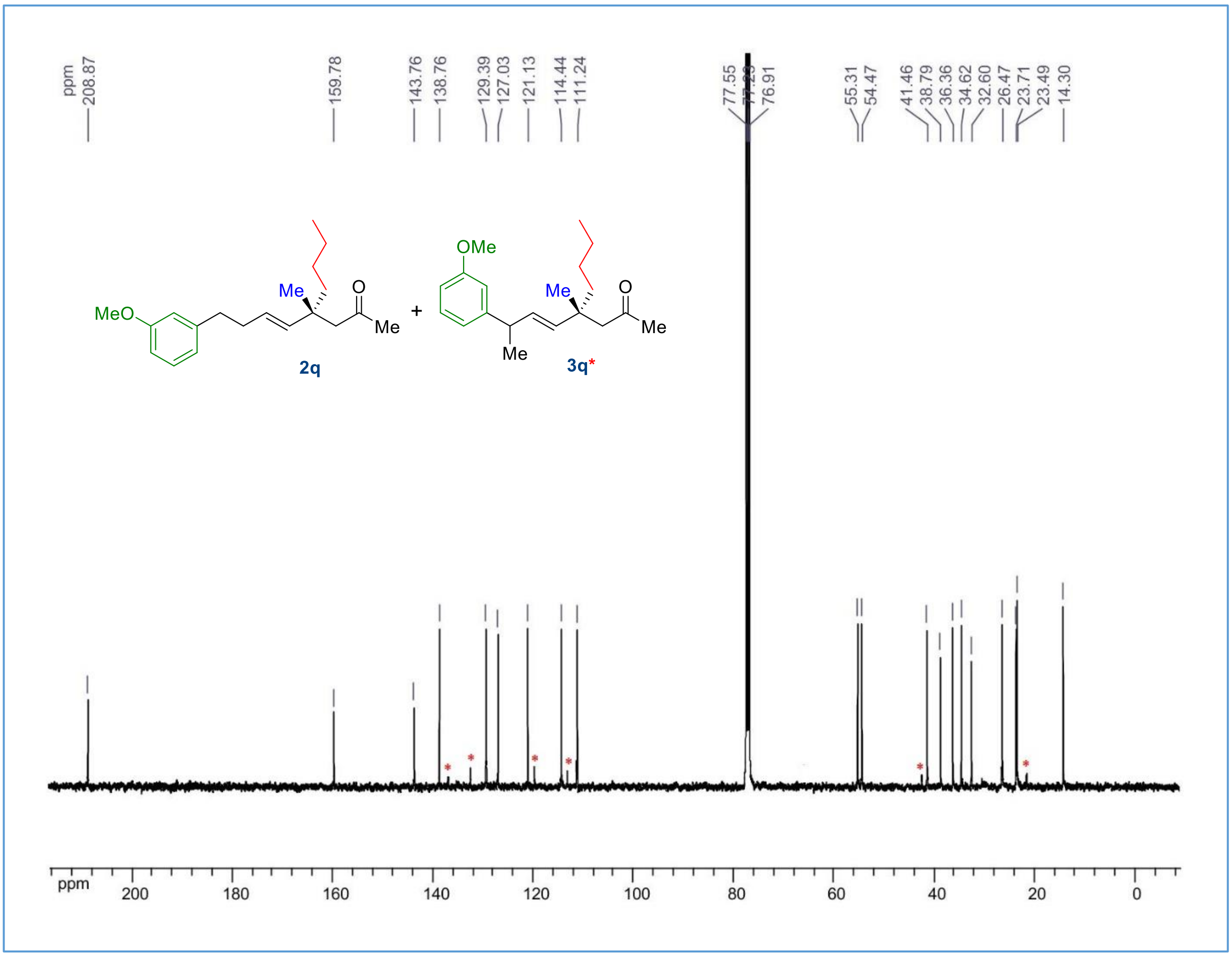

Supplementary Figure 59. ^{1}H and ^{13}C NMR spectra of compound **2q**

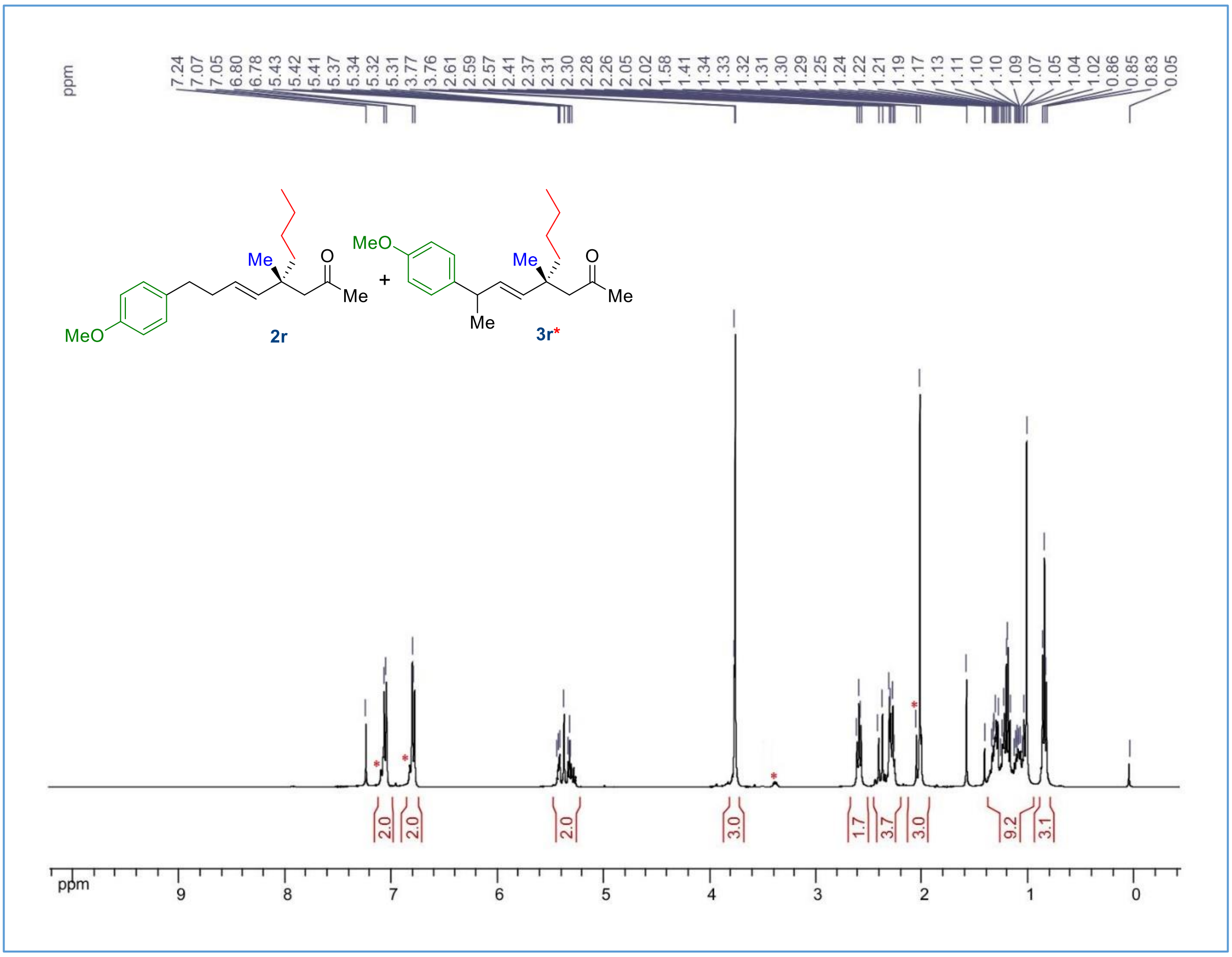

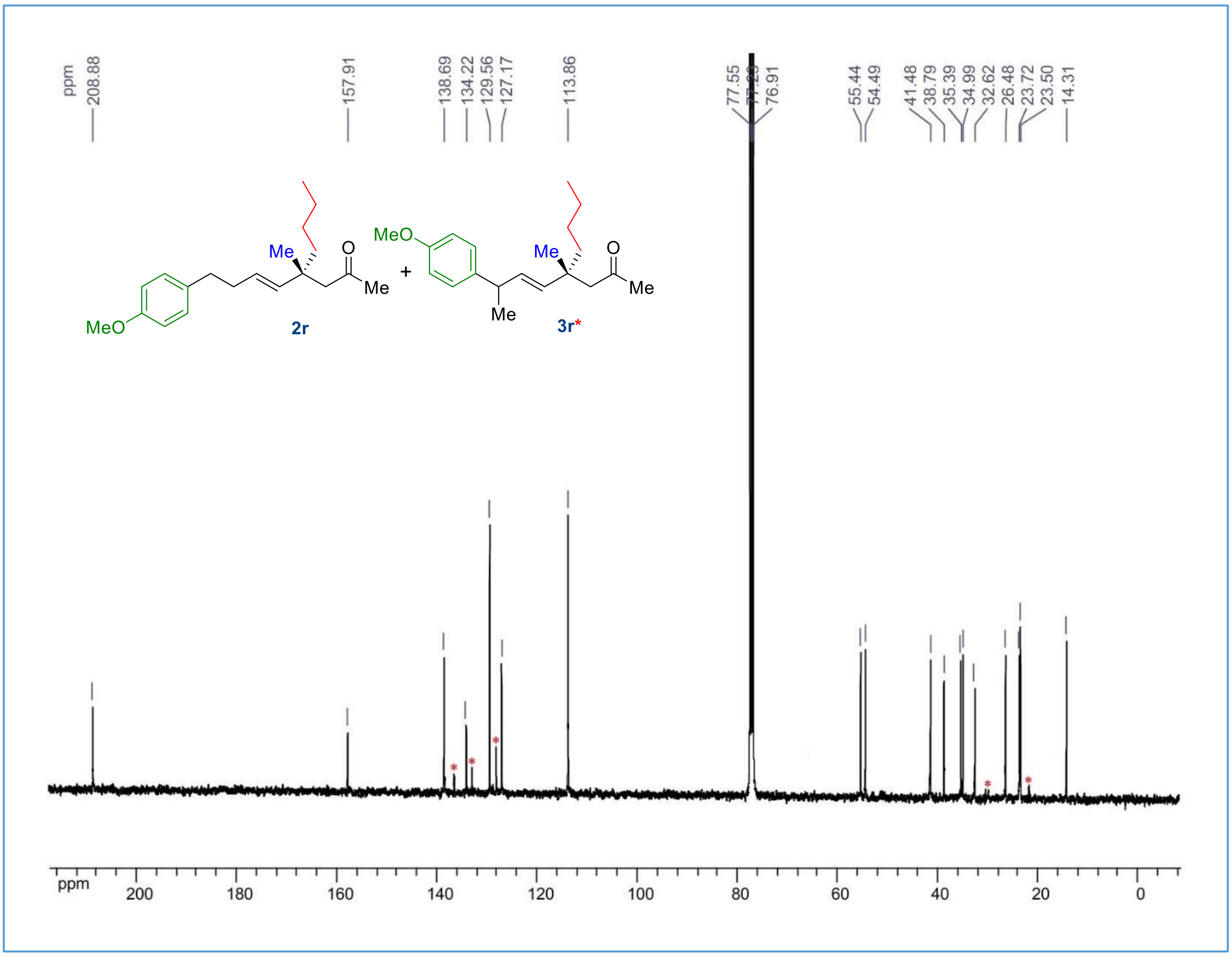

Supplementary Figure 60. ^{1}H and ^{13}C NMR spectra of compound **2r**

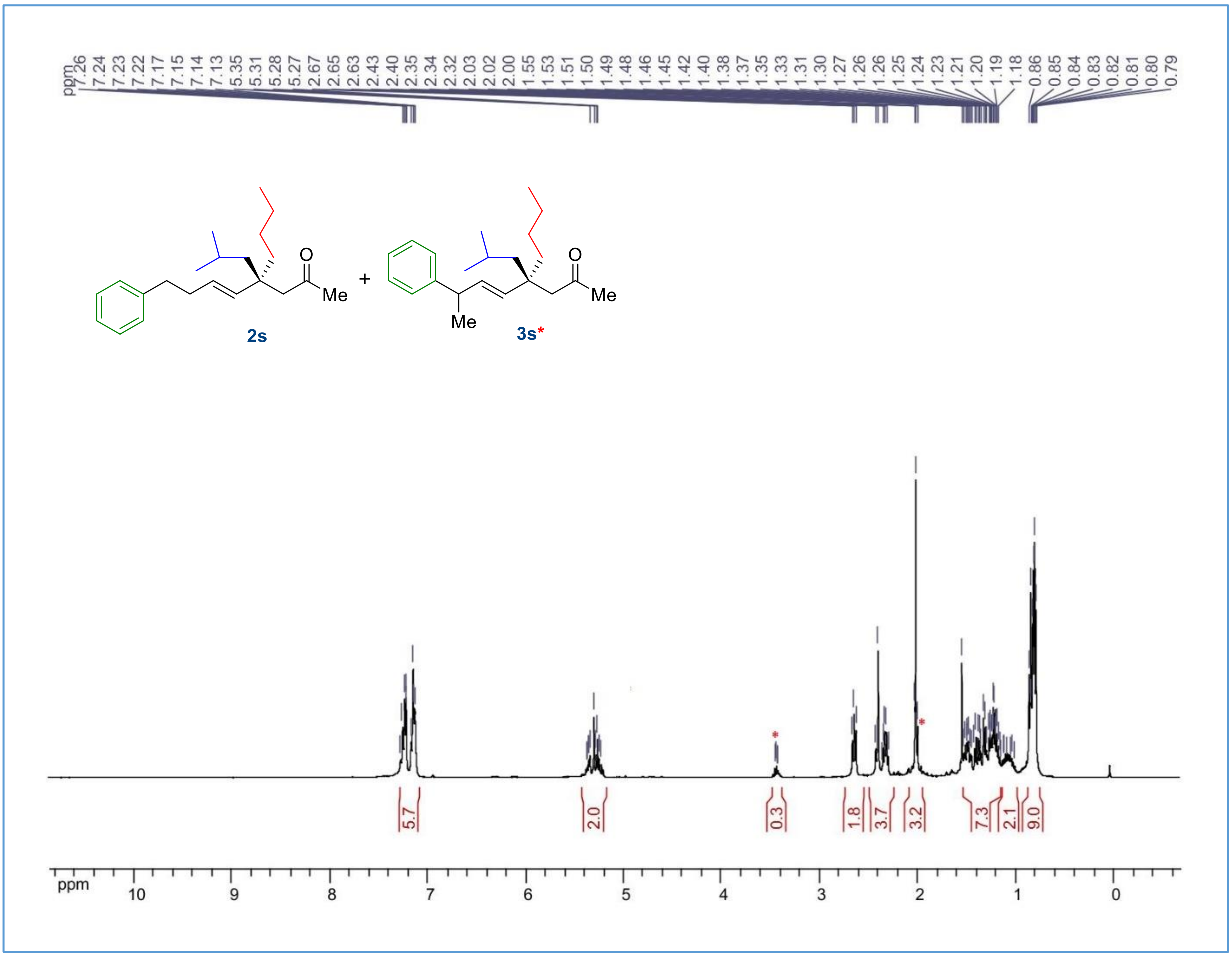

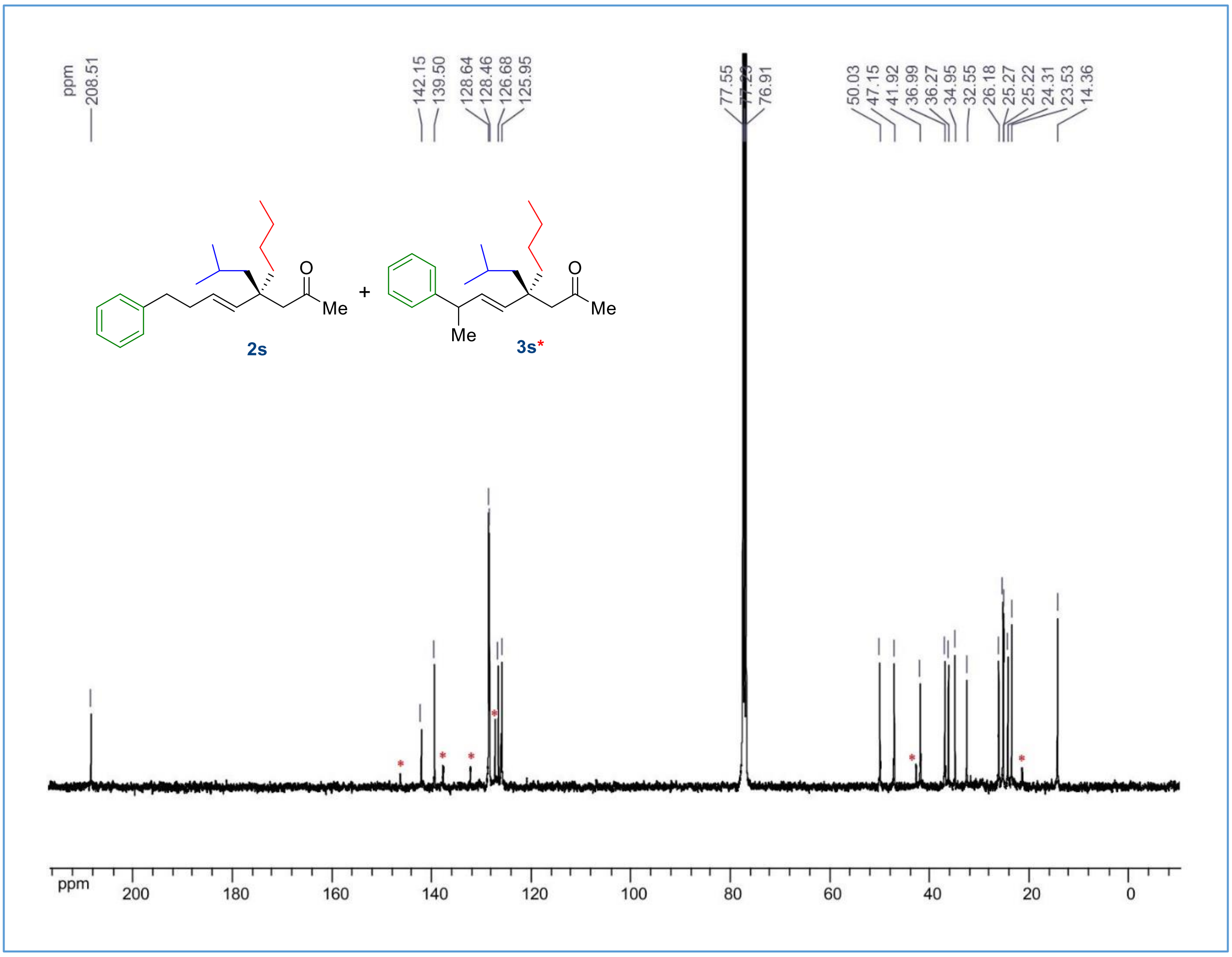

Supplementary Figure 61. ^{1}H and ^{13}C NMR spectra of compound **2s**

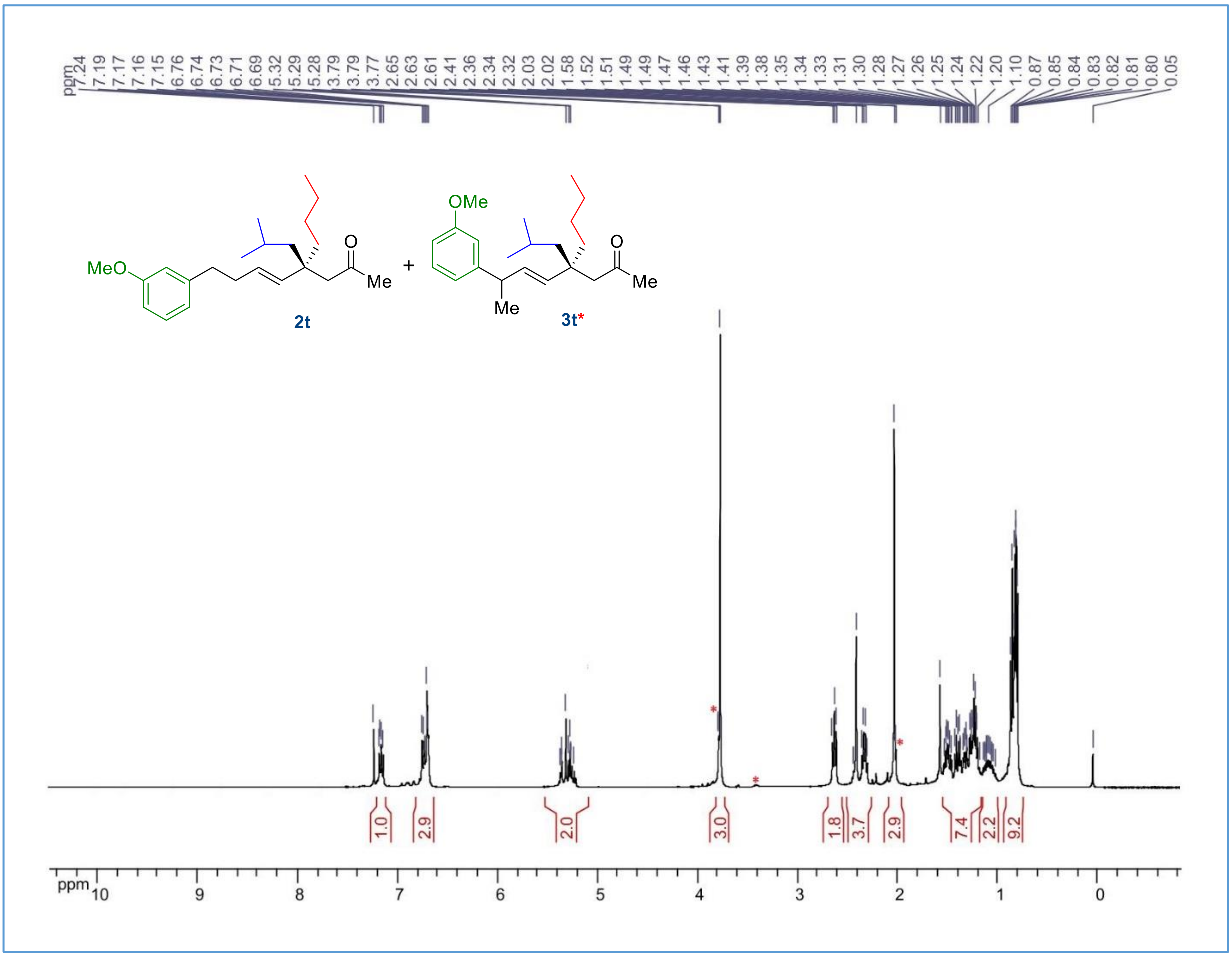

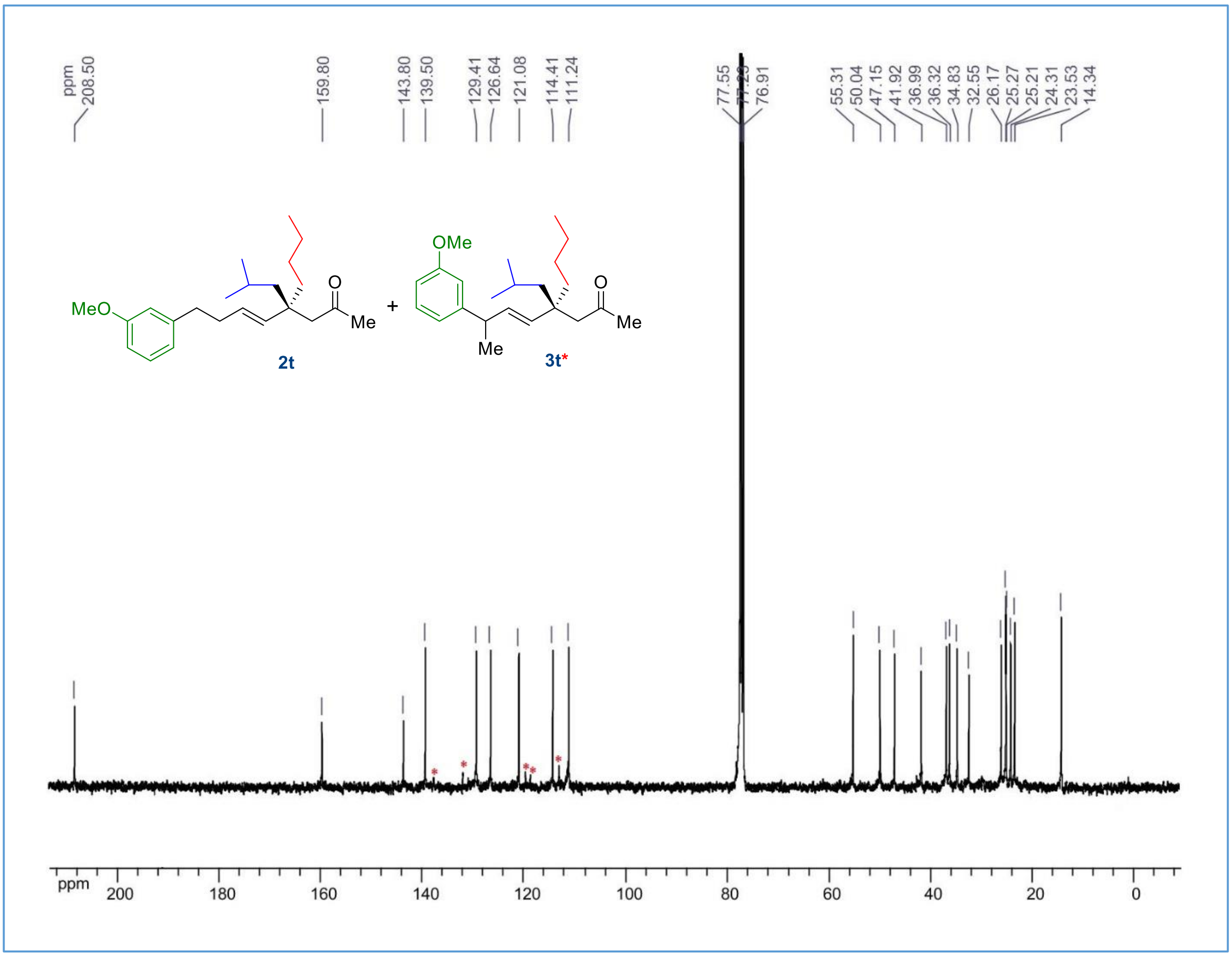

Supplementary Figure 62. ^{1}H and ^{13}C NMR spectra of compound **2t**

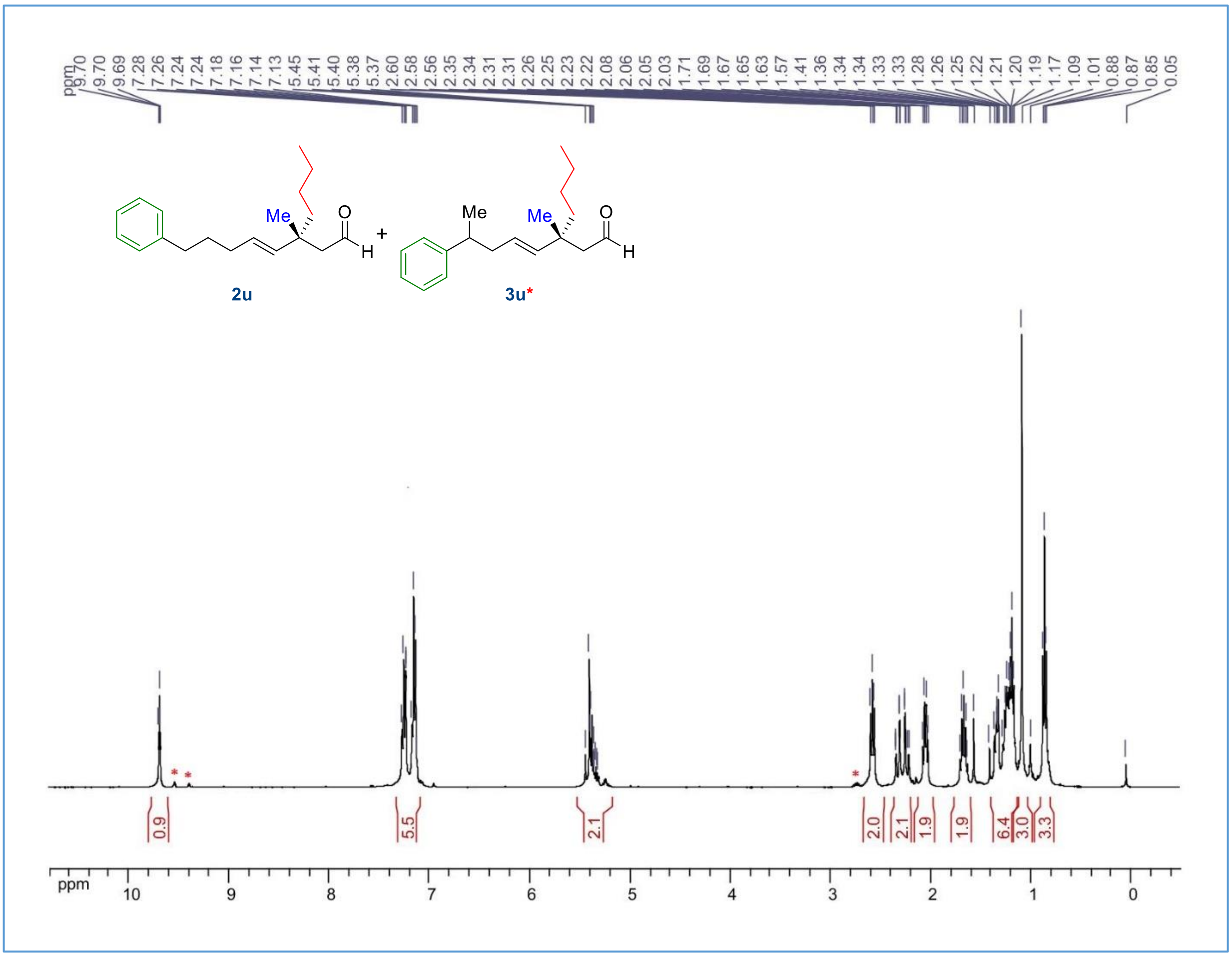

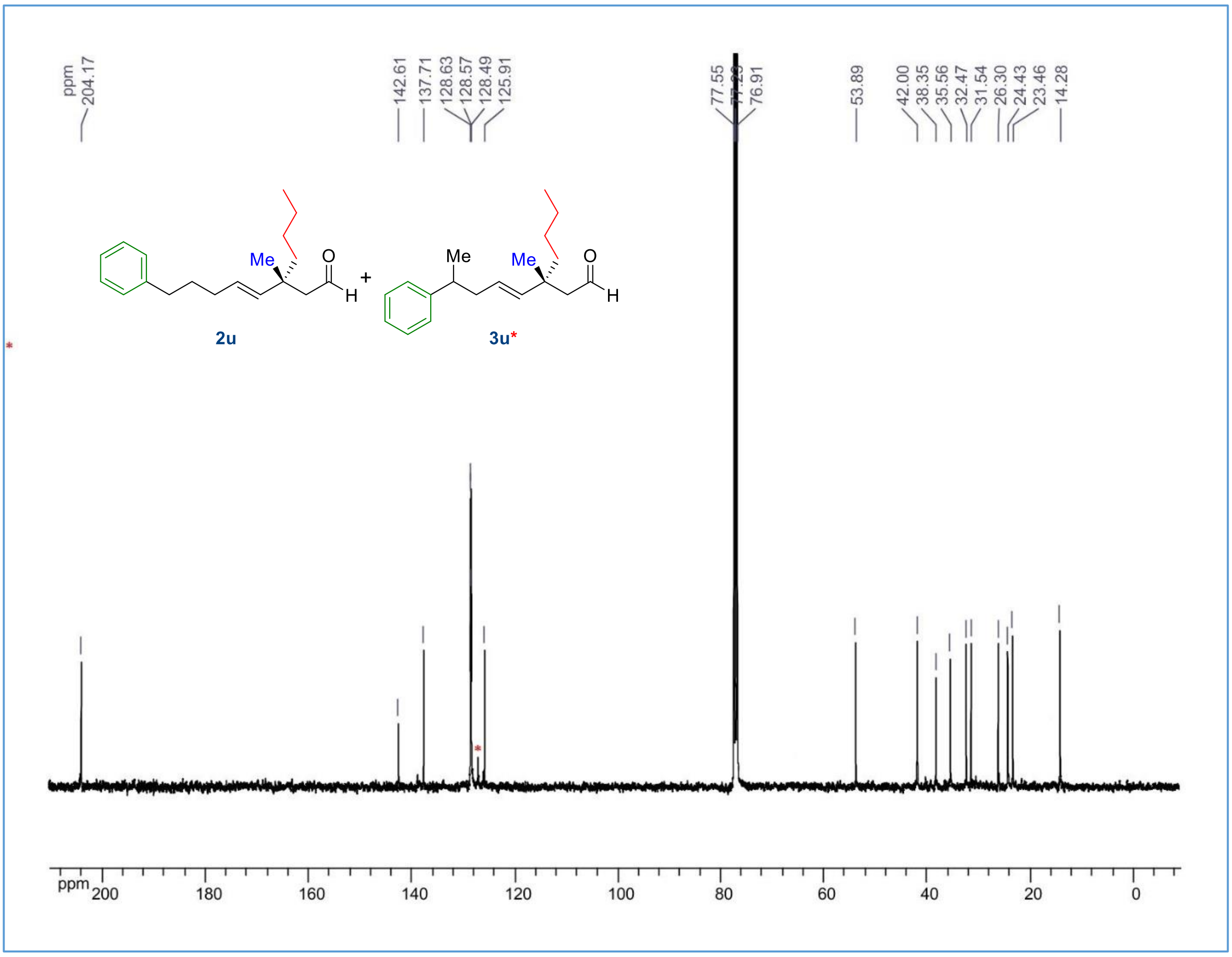

Supplementary Figure 63. ^{1}H and ^{13}C NMR spectra of compound **2u**

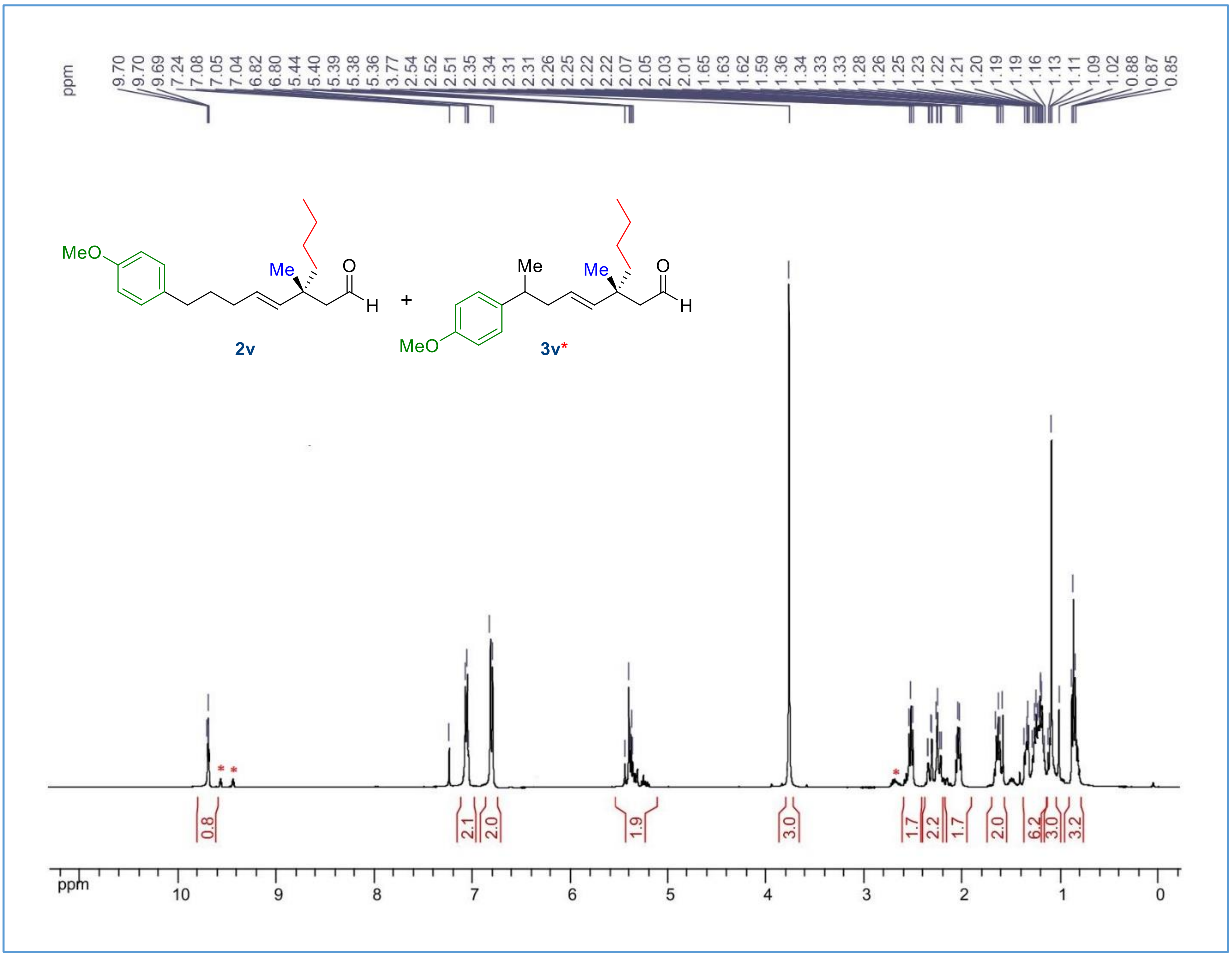

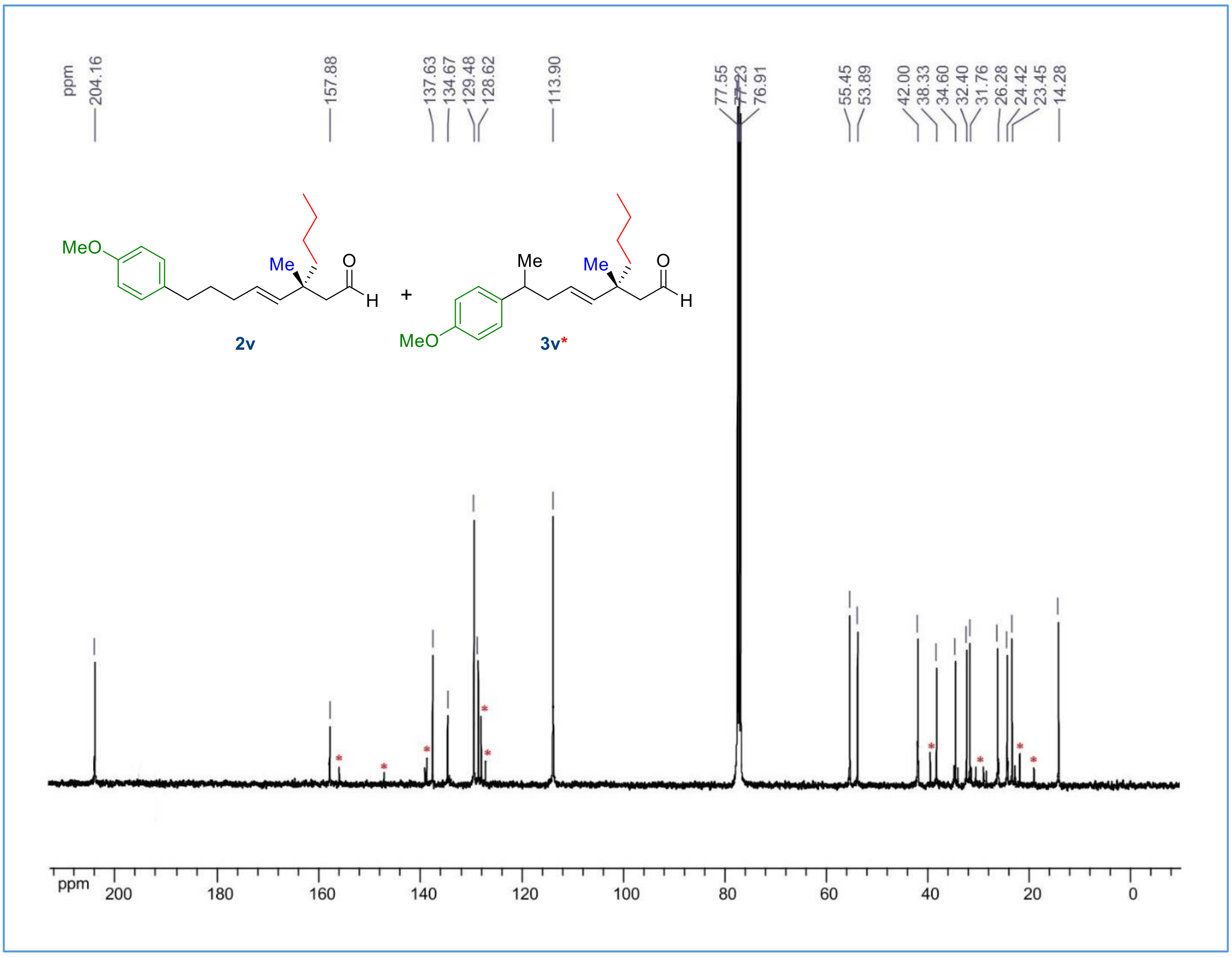

Supplementary Figure 64. ^{1}H and ^{13}C NMR spectra of compound **2v**

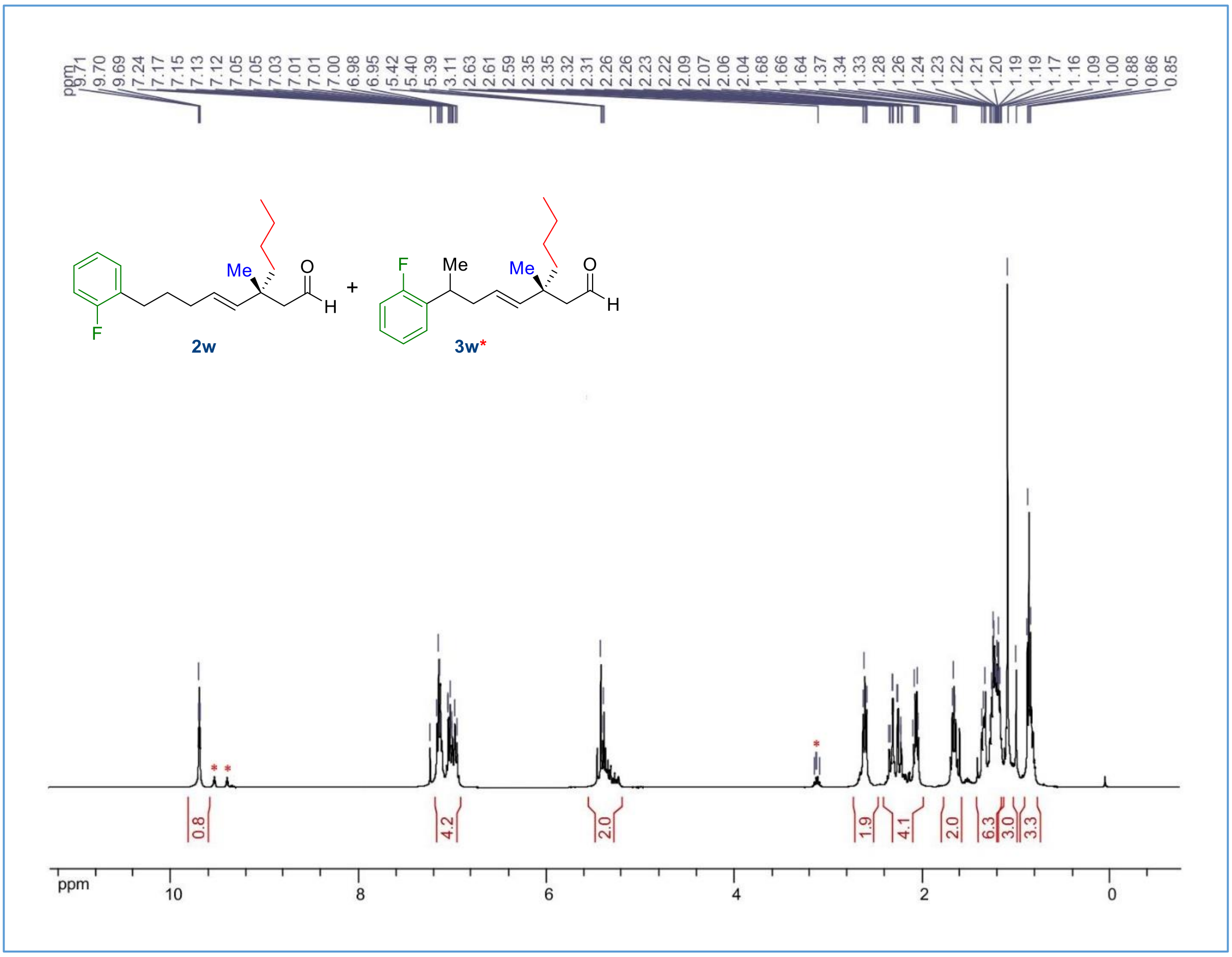

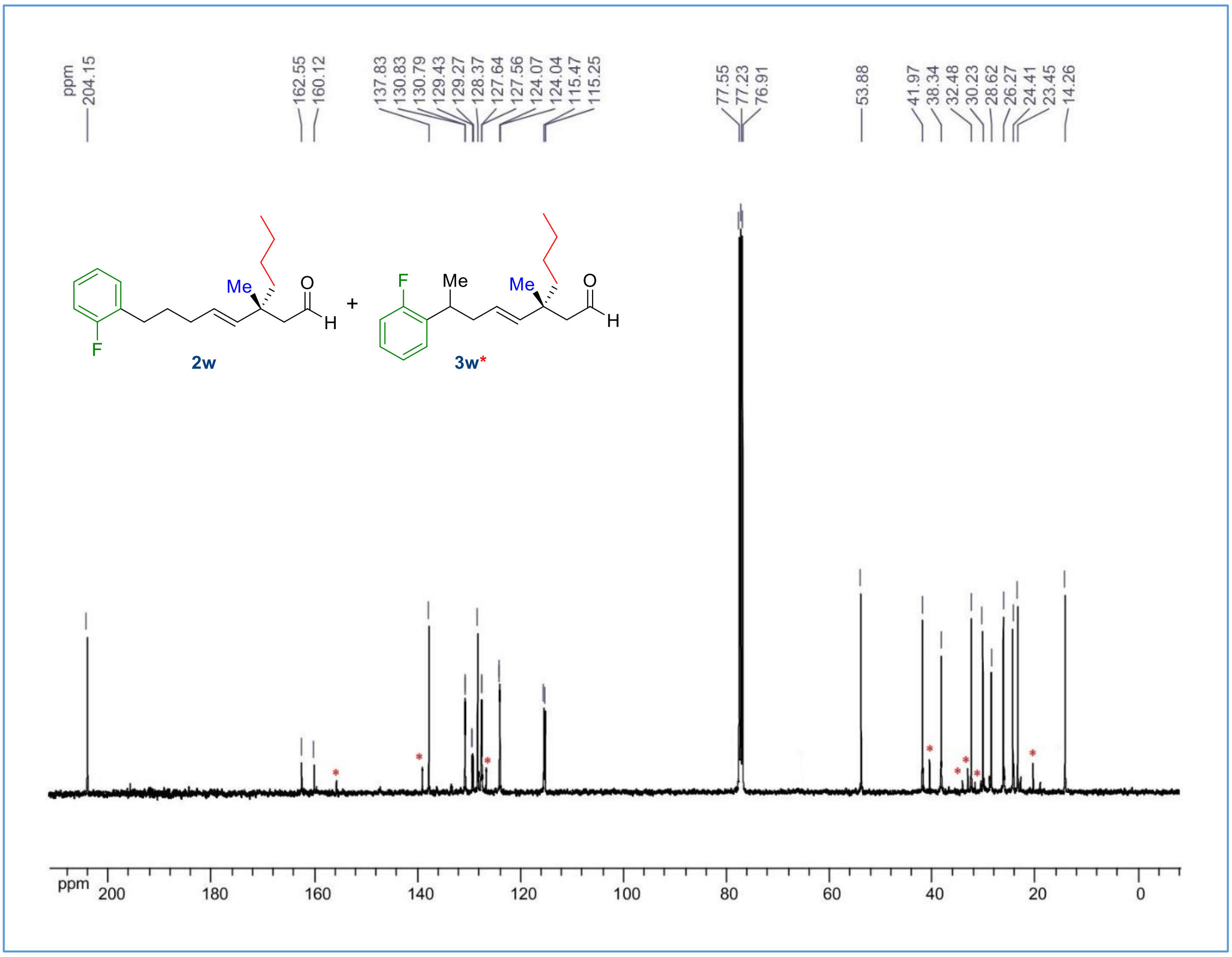

Supplementary Figure 65. ^{1}H and ^{13}C NMR spectra of compound **2w**

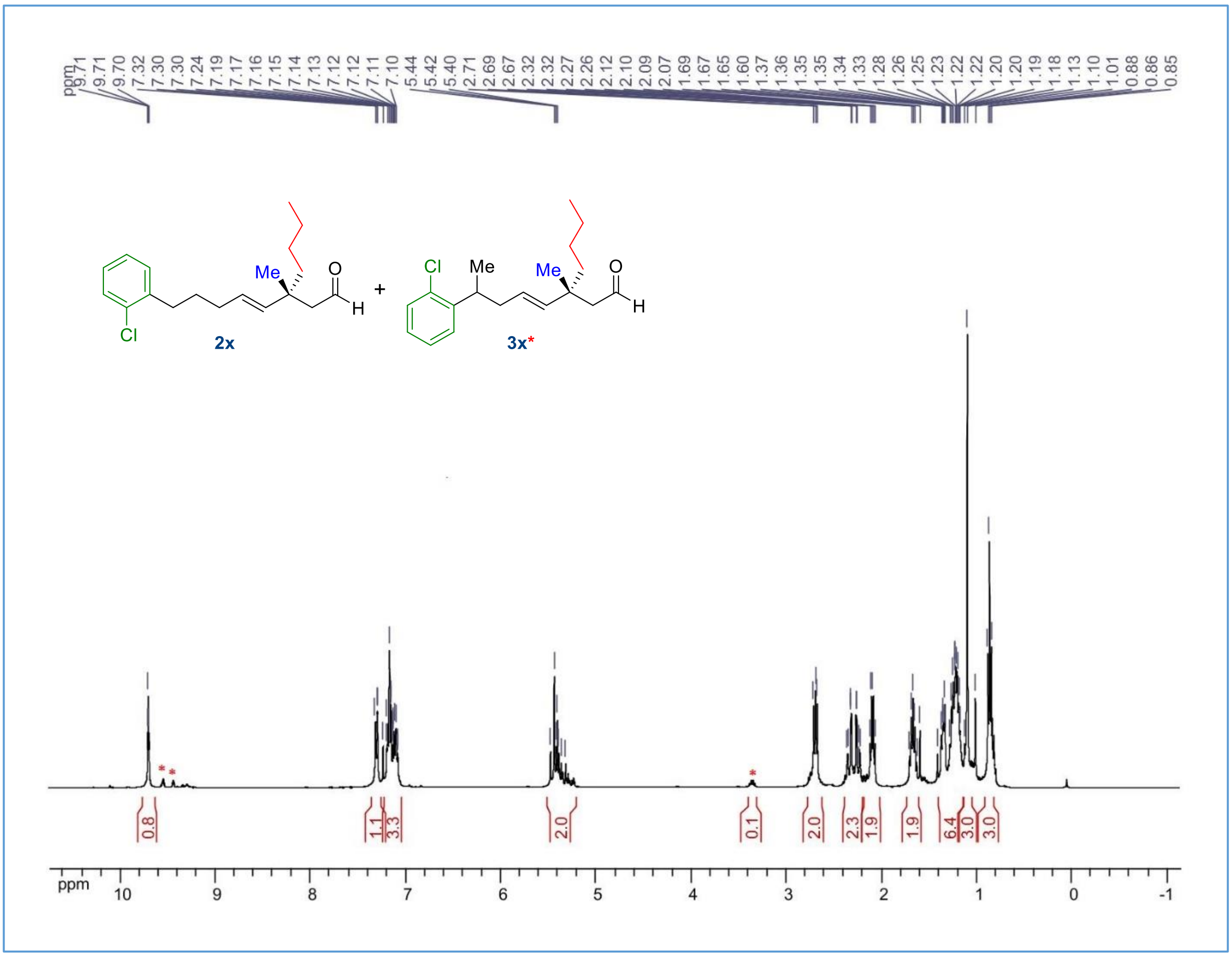

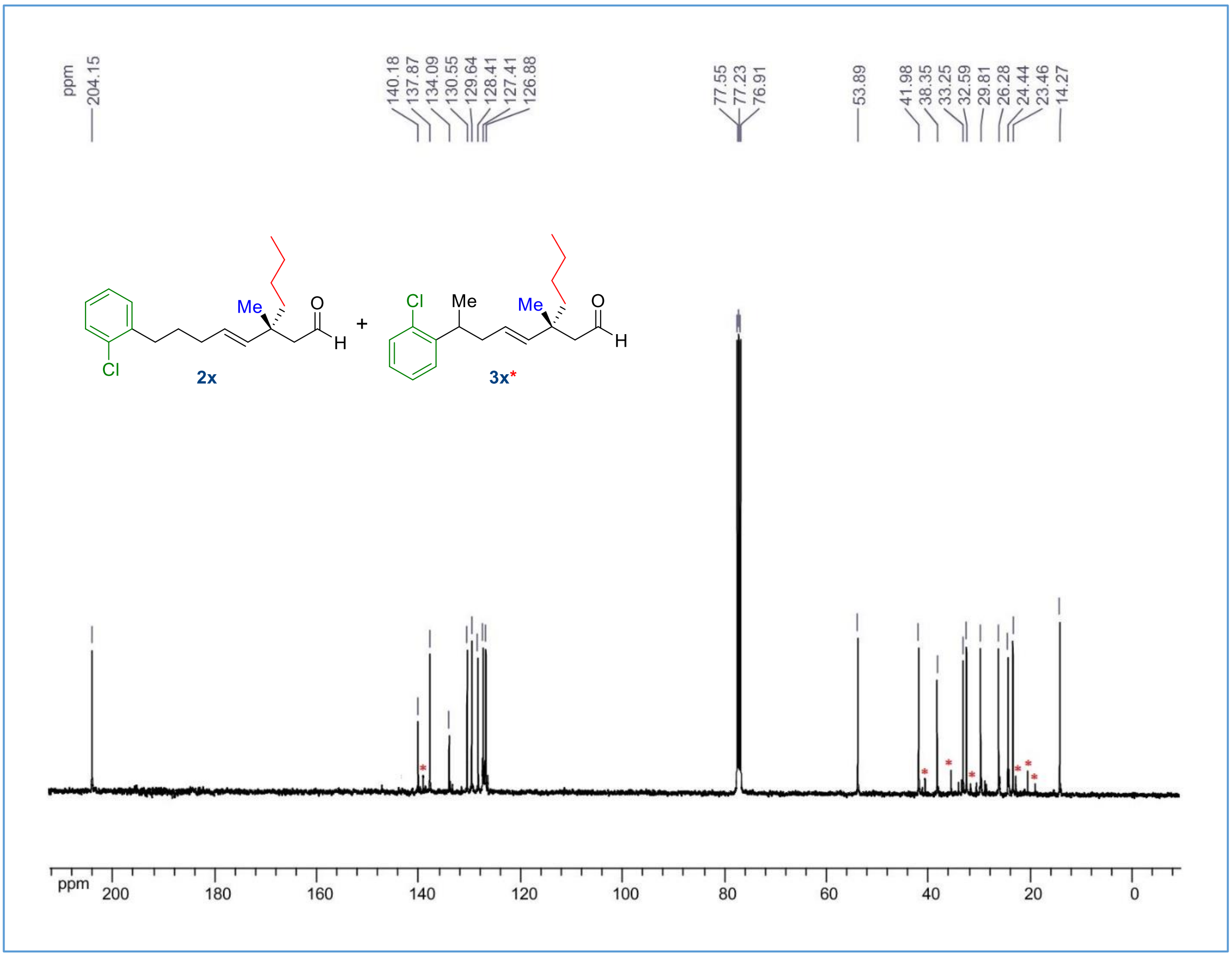

Supplementary Figure 66. ^{1}H and ^{13}C NMR spectra of compound **2x**

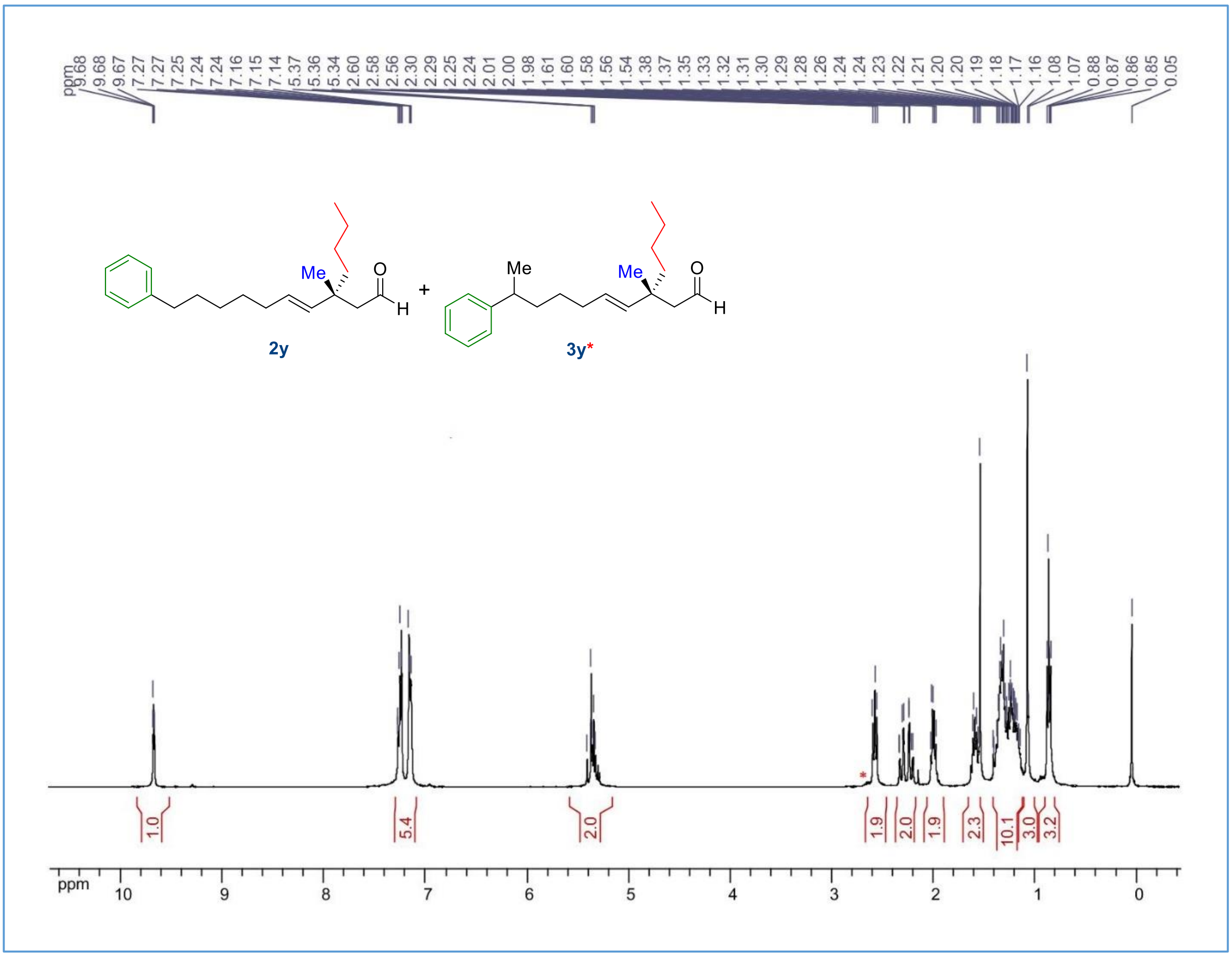

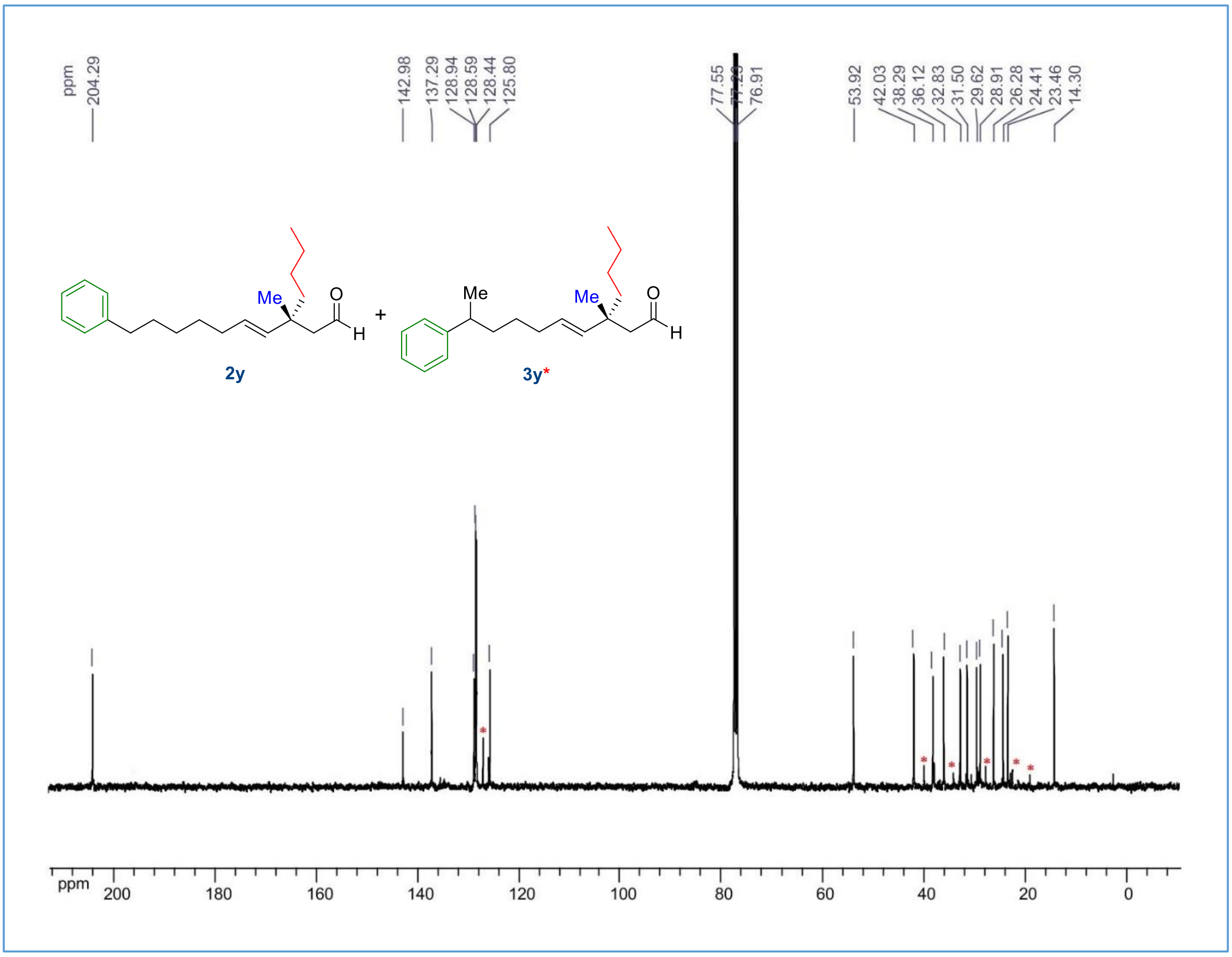

Supplementary Figure 67. ^{1}H and ^{13}C NMR spectra of compound **2y**

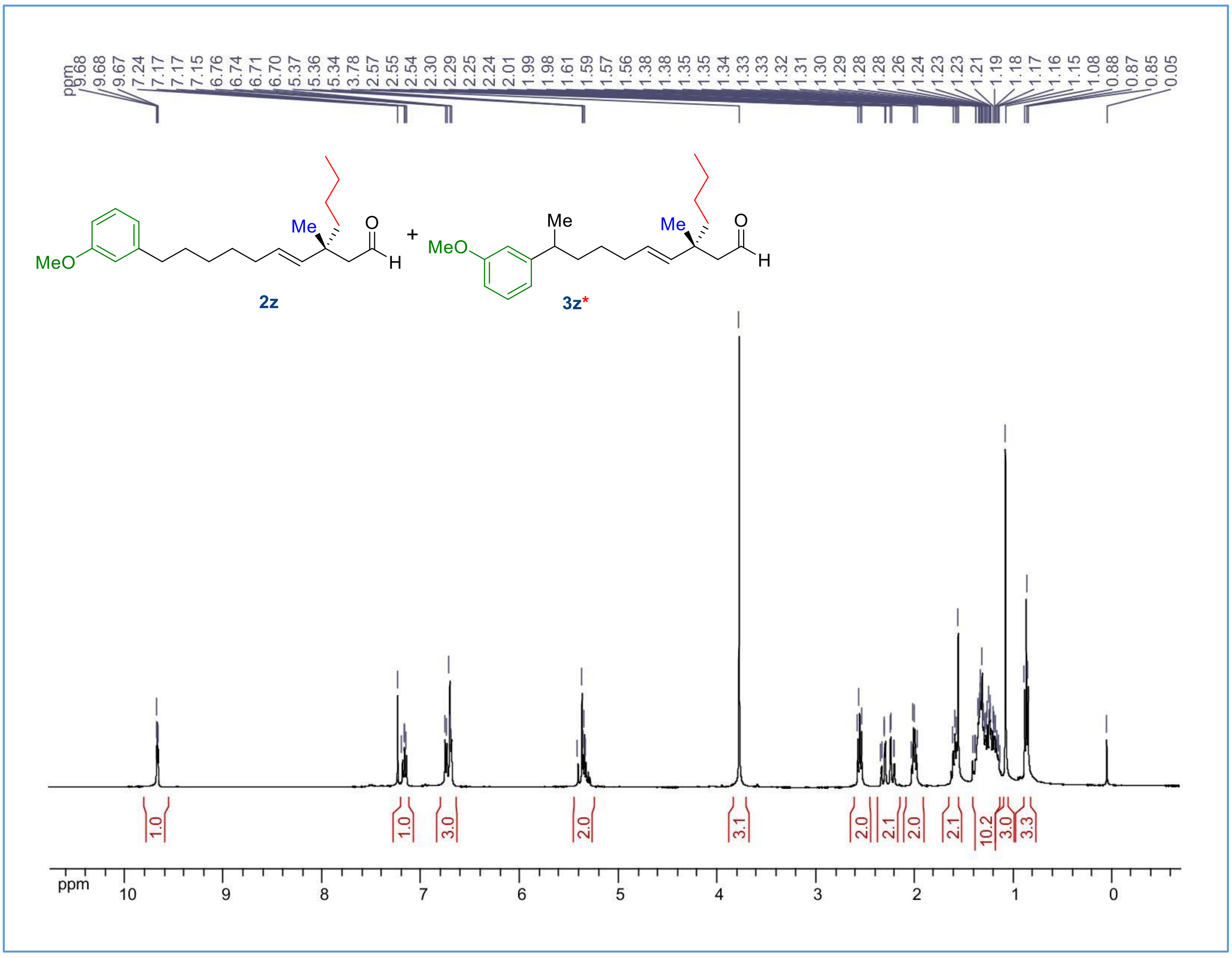

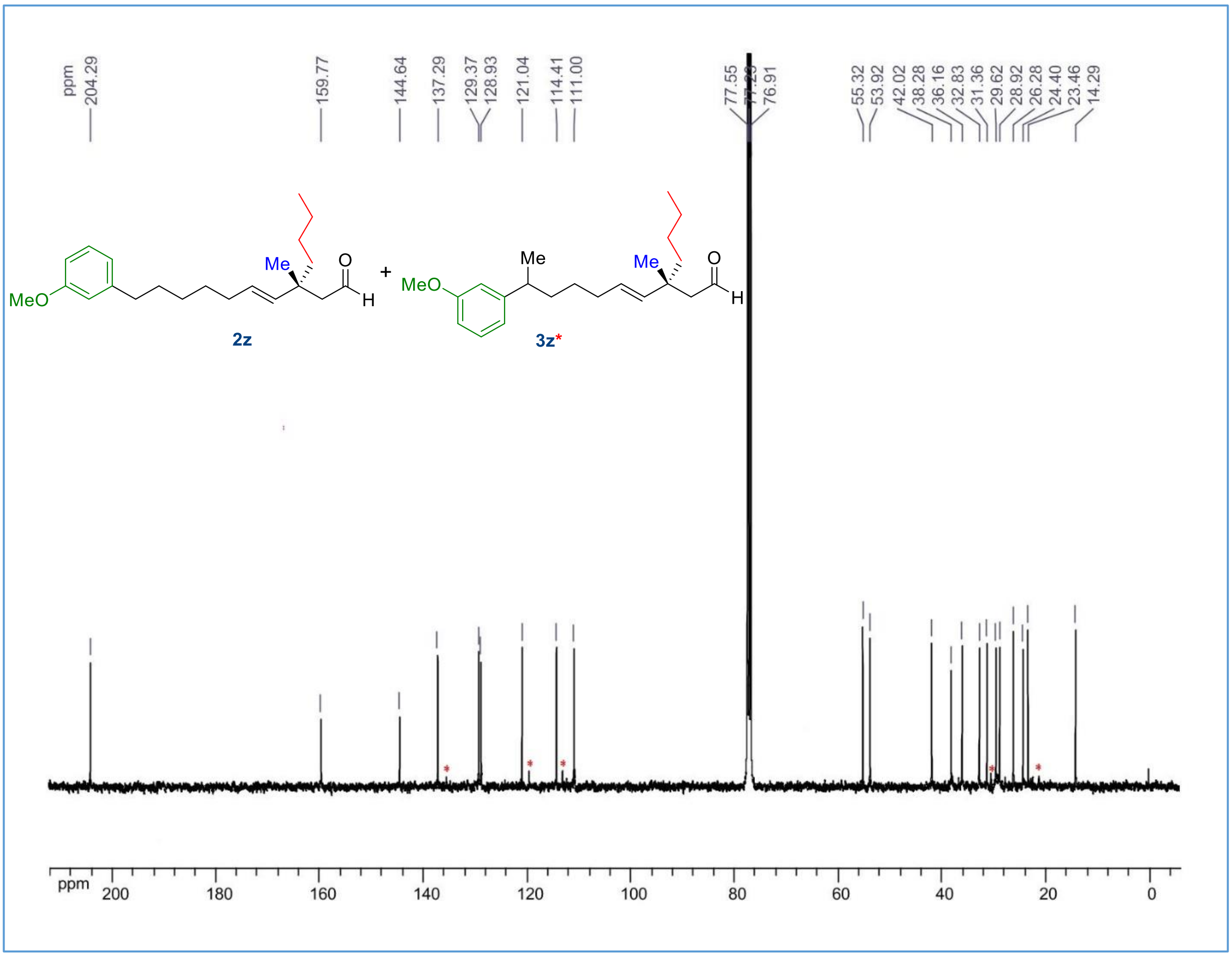

Supplementary Figure 68. ^{1}H and ^{13}C NMR spectra of compound **2z**

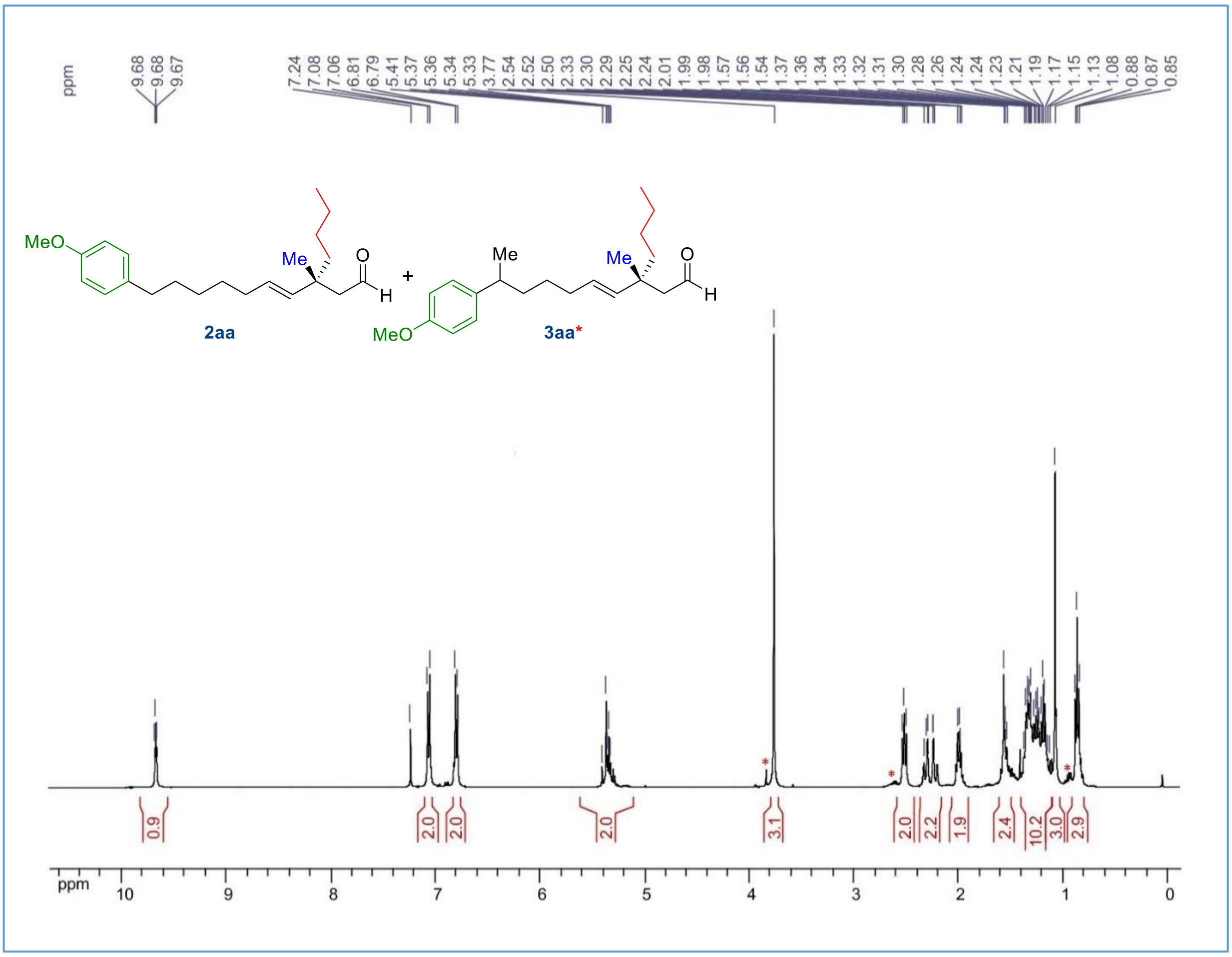

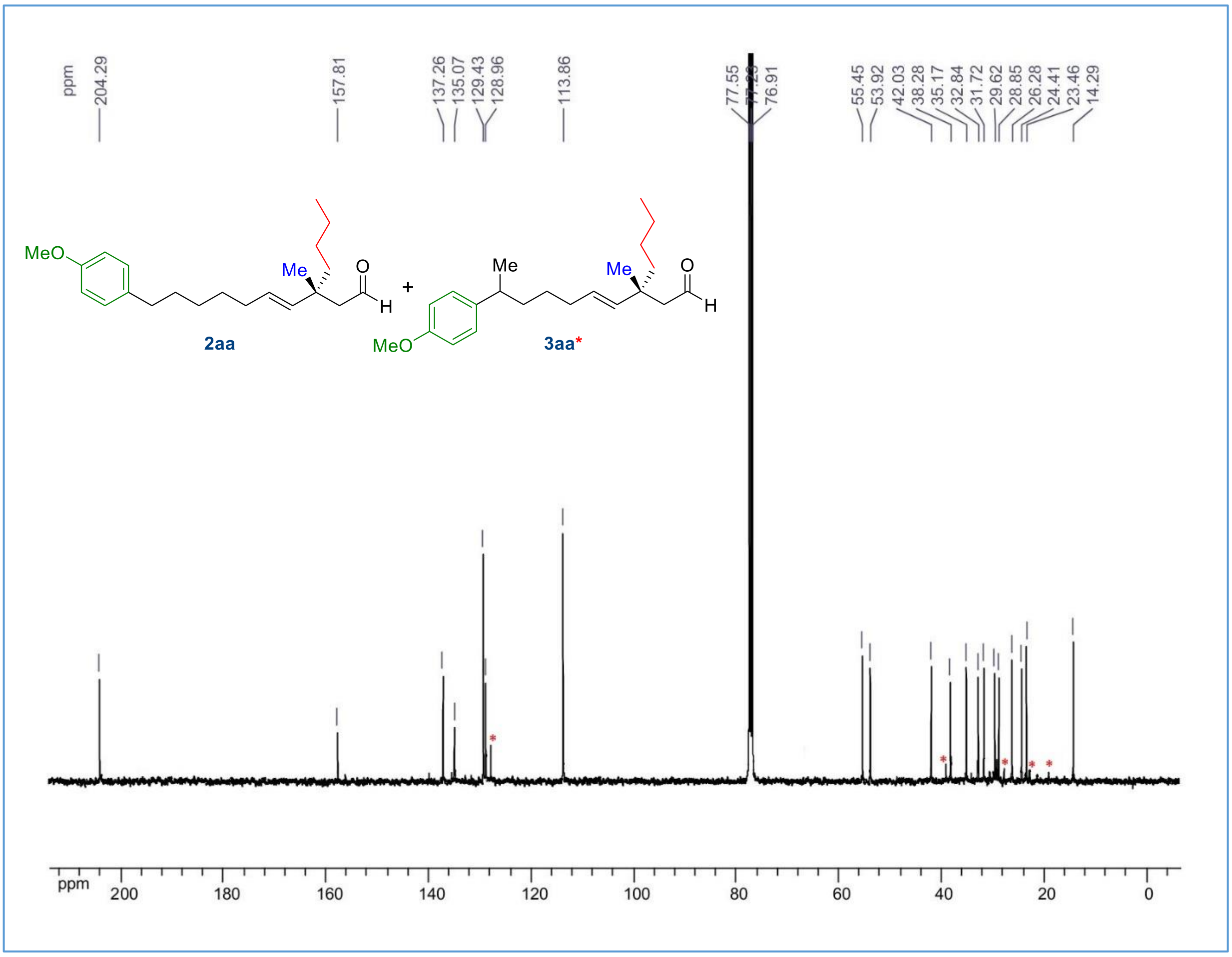

Supplementary Figure 69. ^{1}H and ^{13}C NMR spectra of compound **2aa**

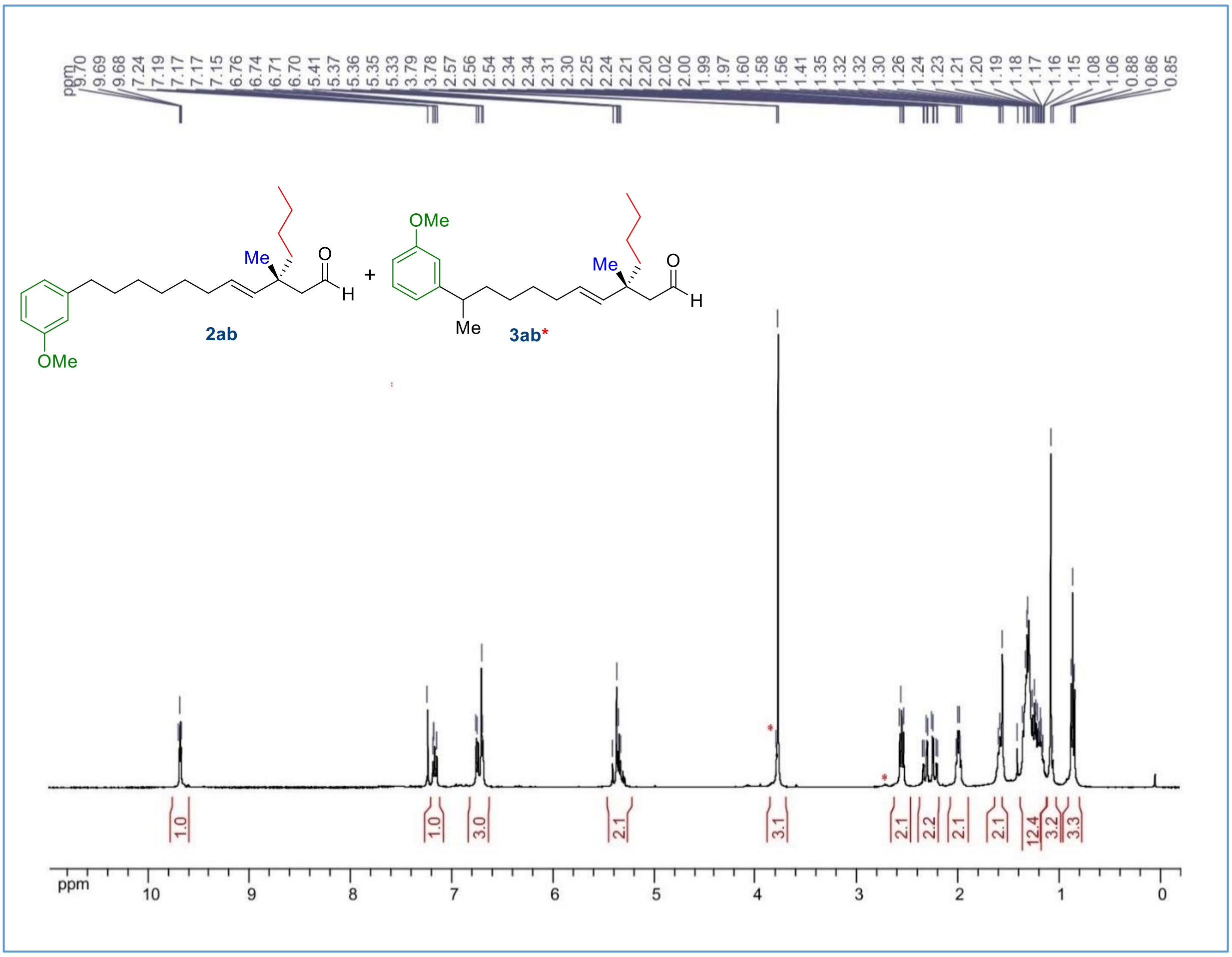

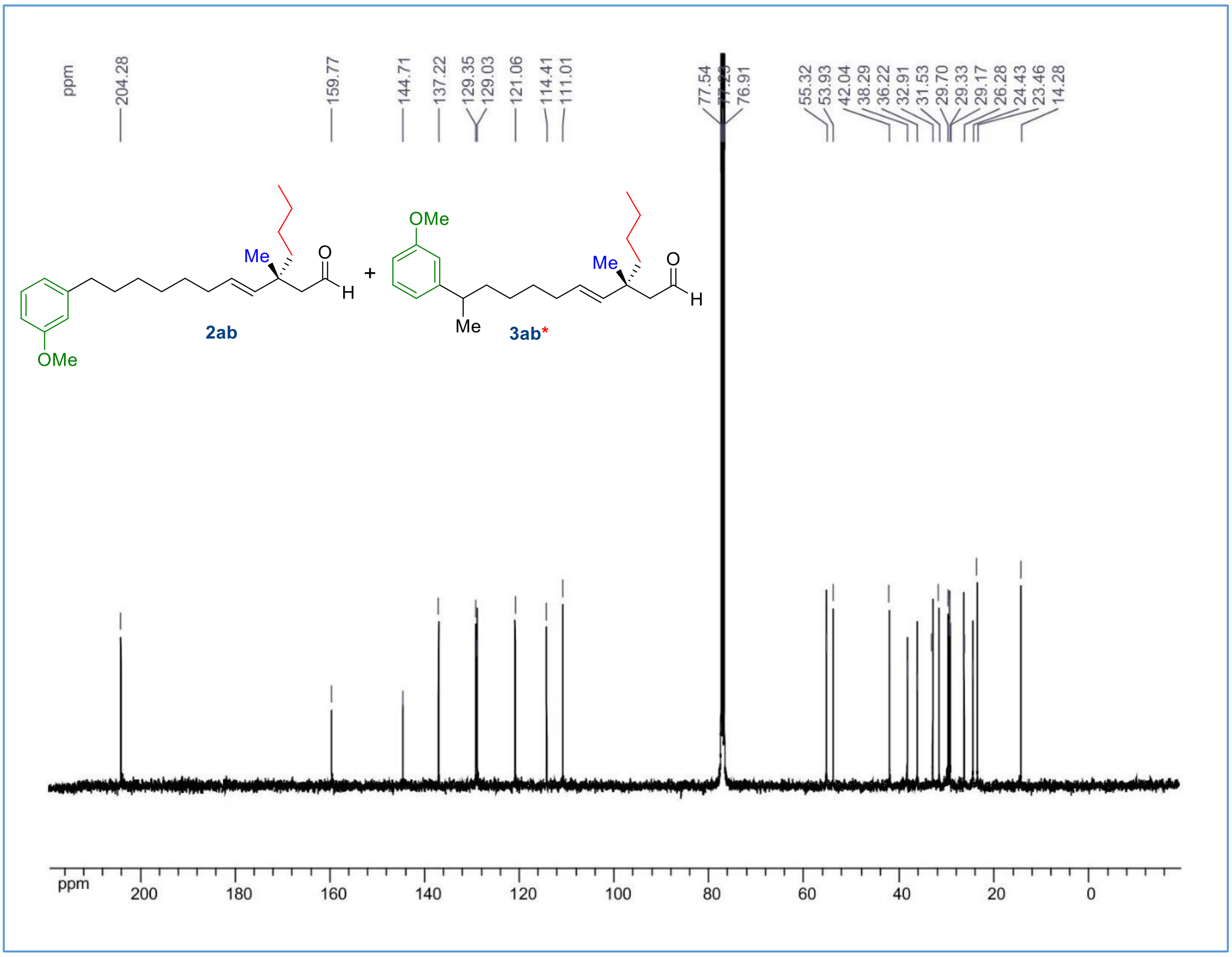

Supplementary Figure 70. ^{1}H and ^{13}C NMR spectra of compound **2ab**

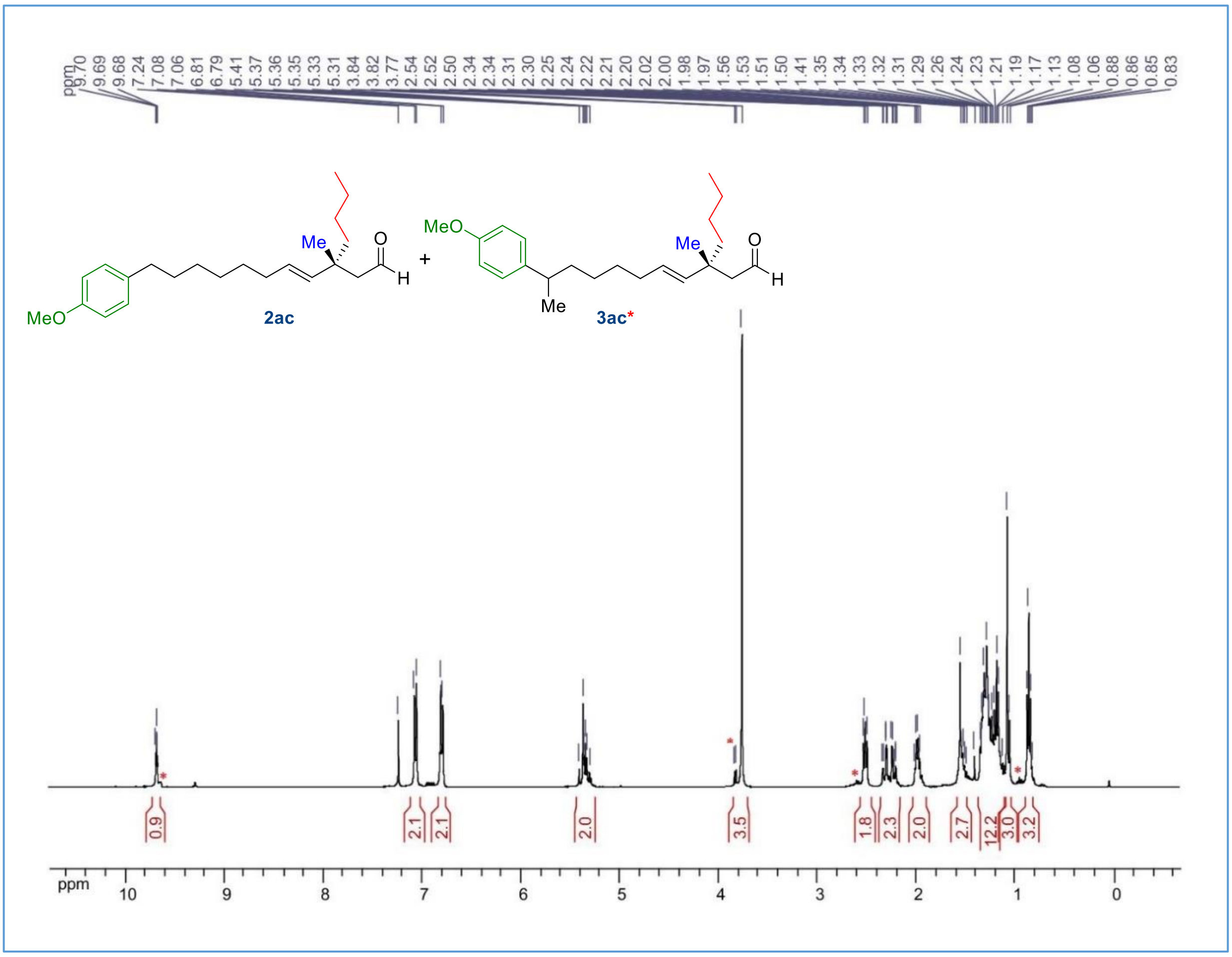

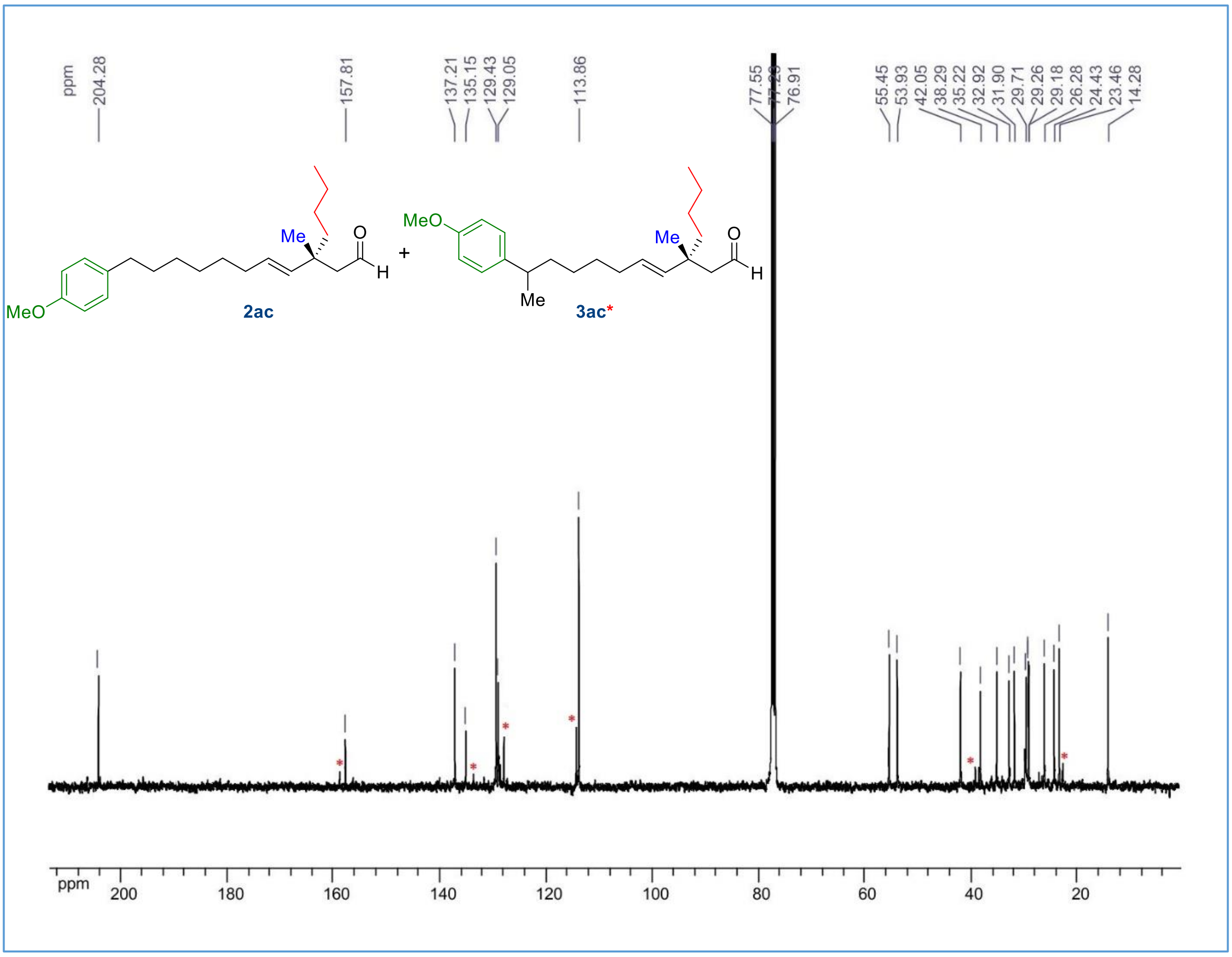

Supplementary Figure 71. ^{1}H and ^{13}C NMR spectra of compound **2ac**

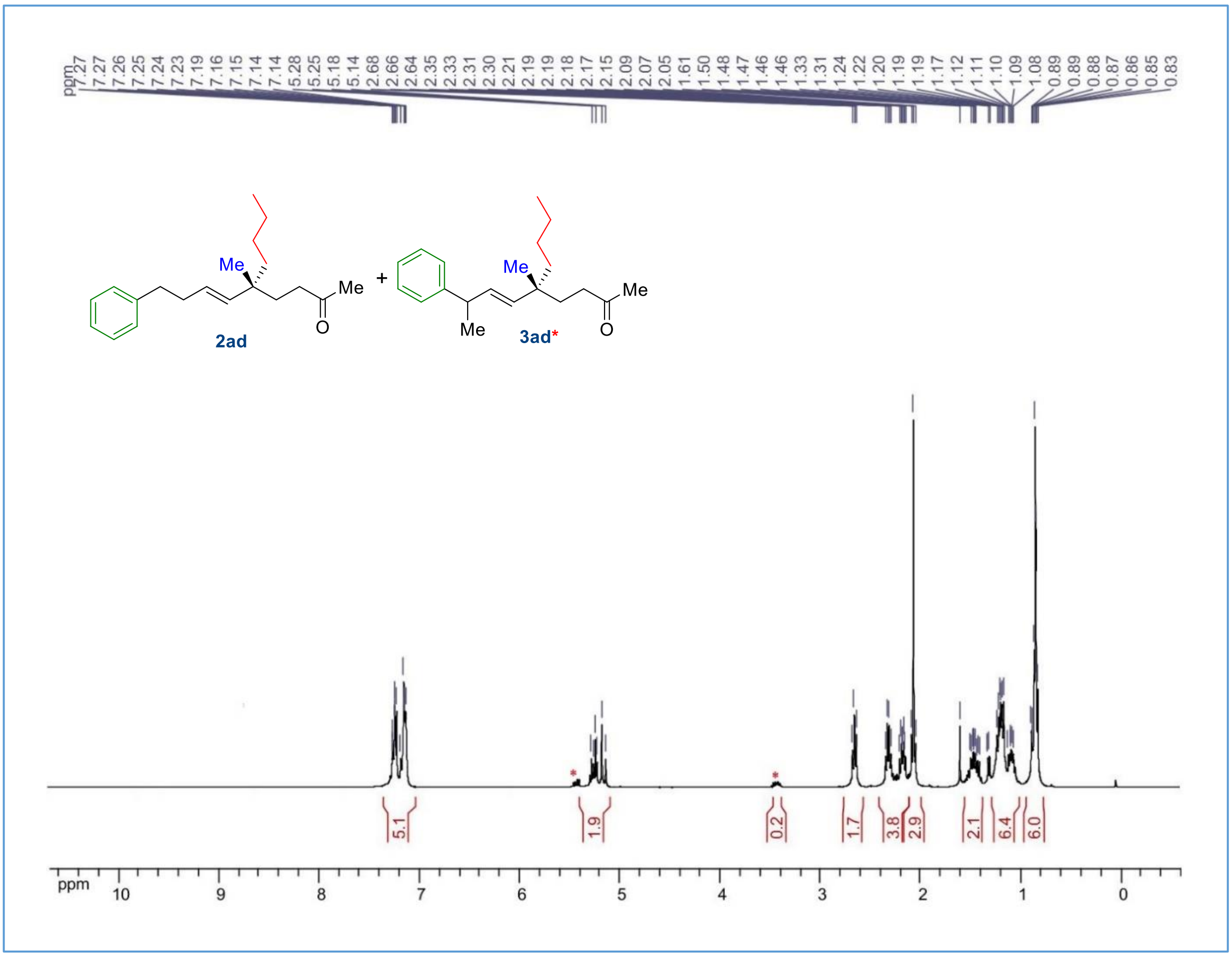

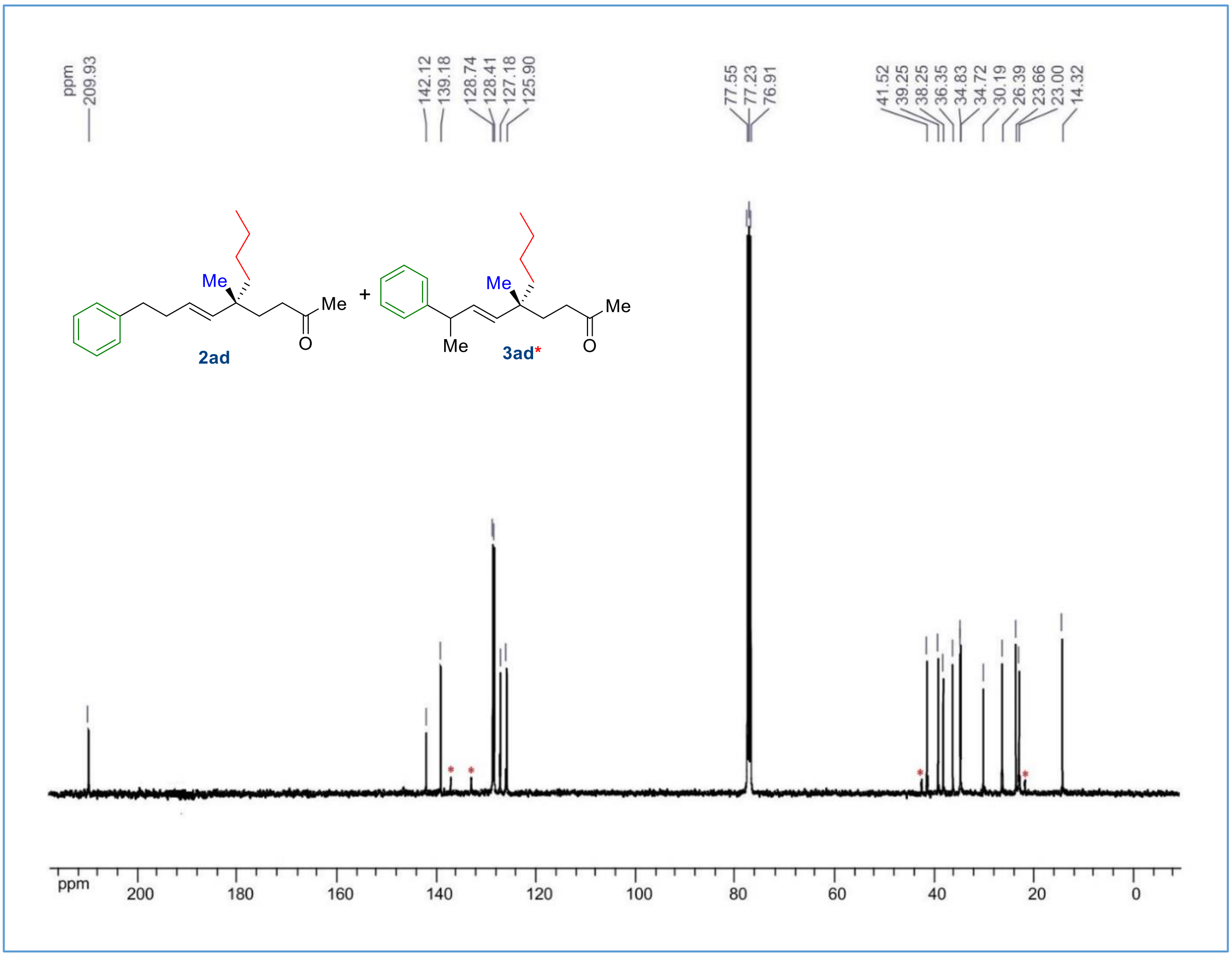

Supplementary Figure 72. ^{1}H and ^{13}C NMR spectra of compound **2ad**

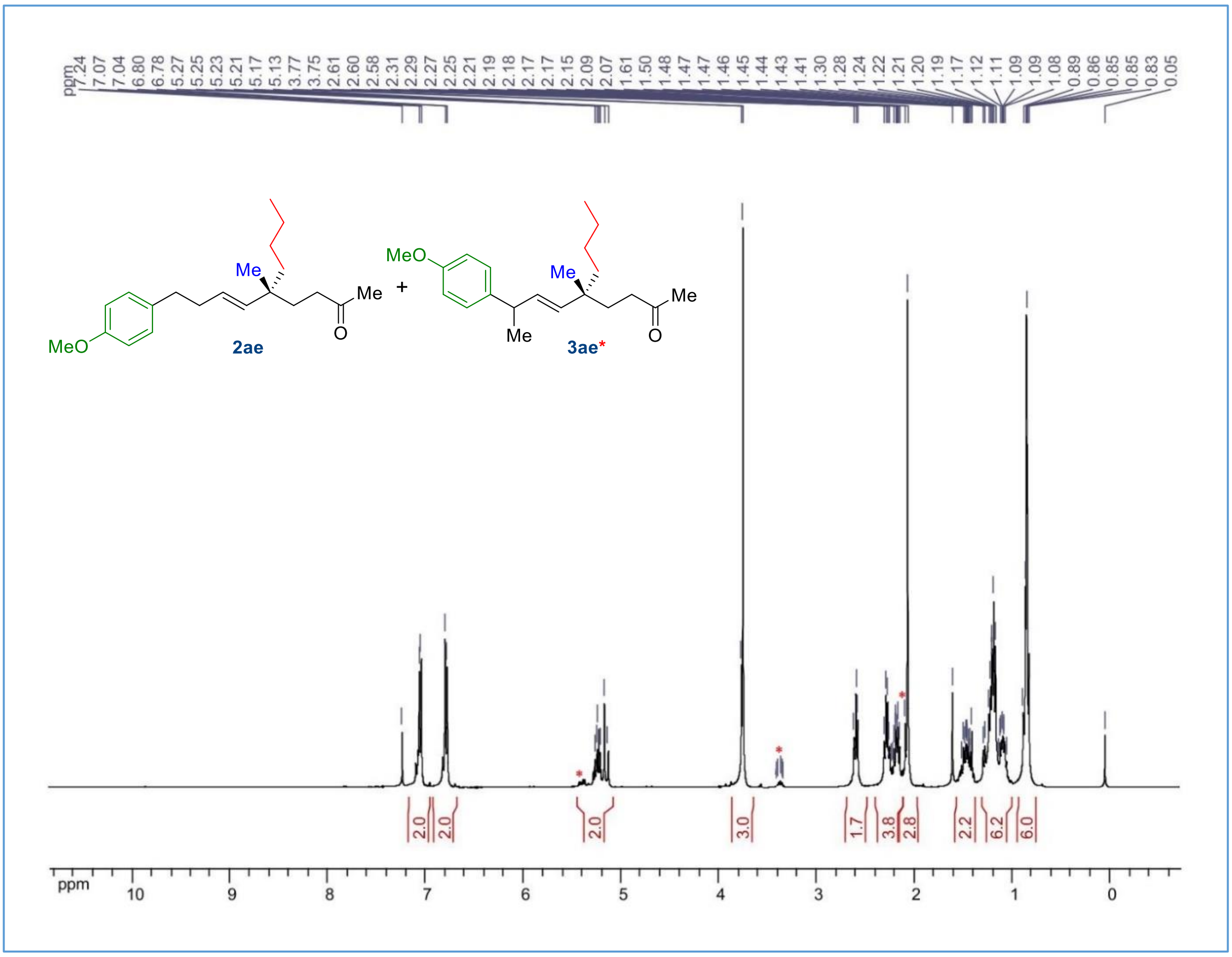

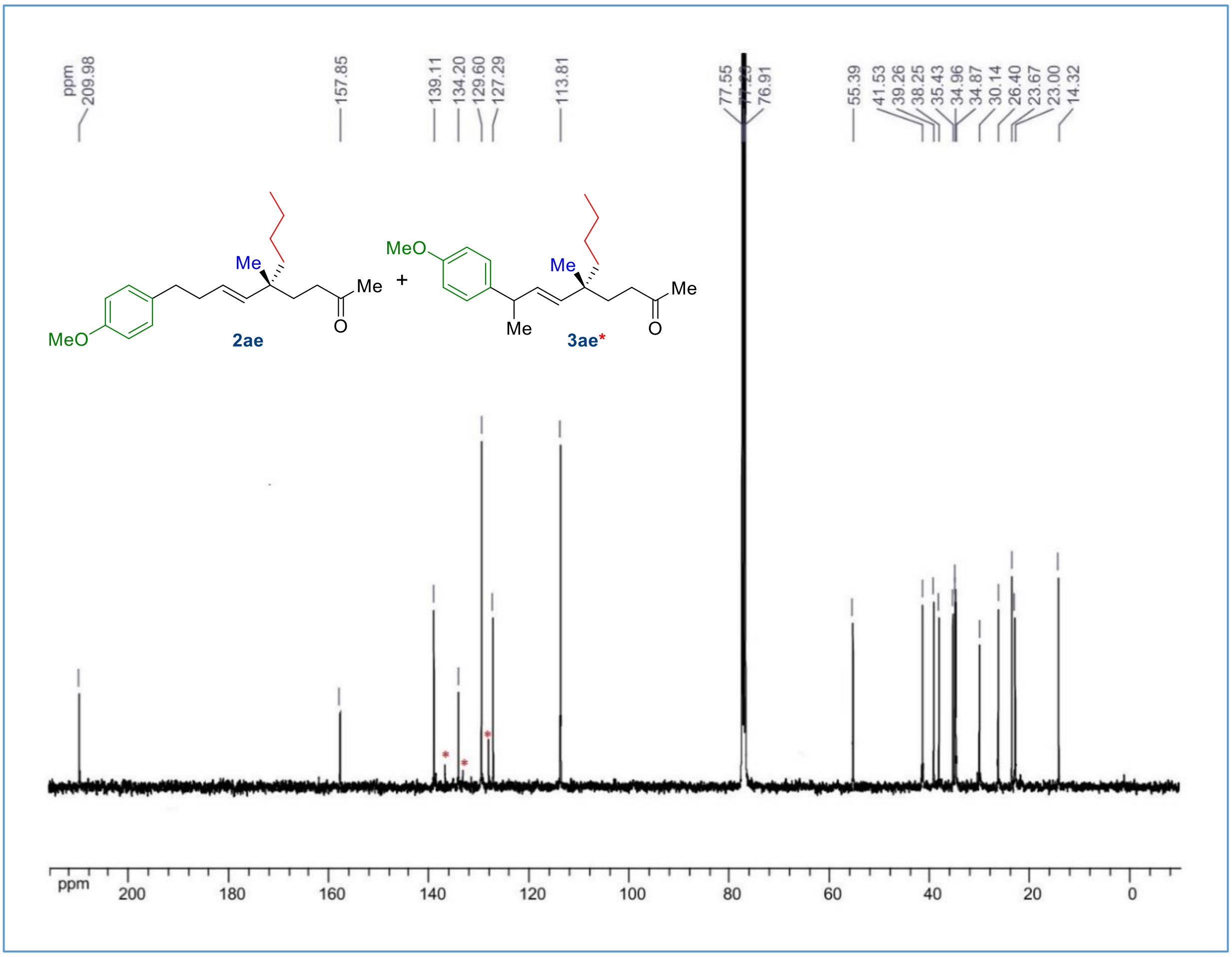

Supplementary Figure 73. ^{1}H and ^{13}C NMR spectra of compound **2ae**

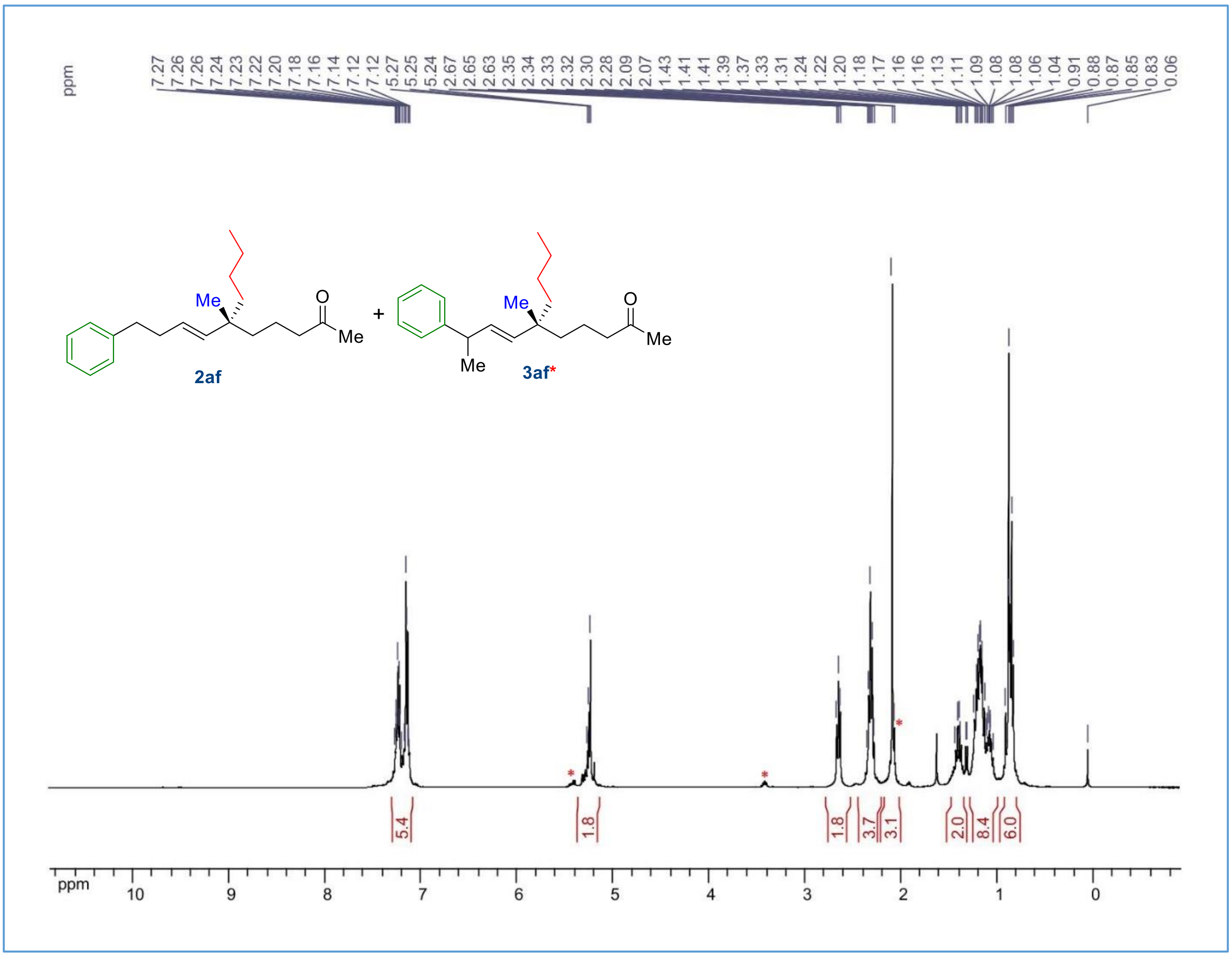

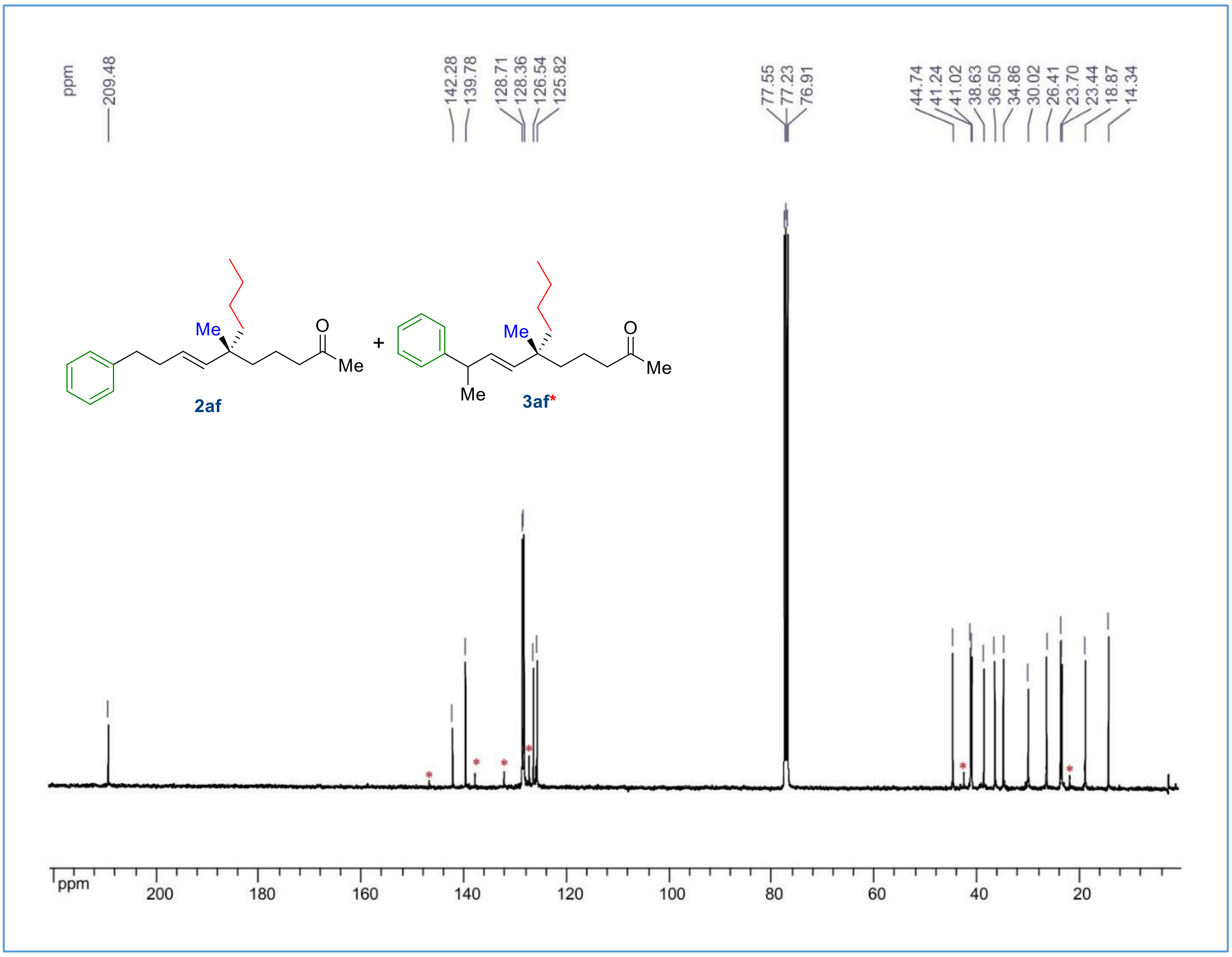

Supplementary Figure 74. ^{1}H and ^{13}C NMR spectra of compound **2af**

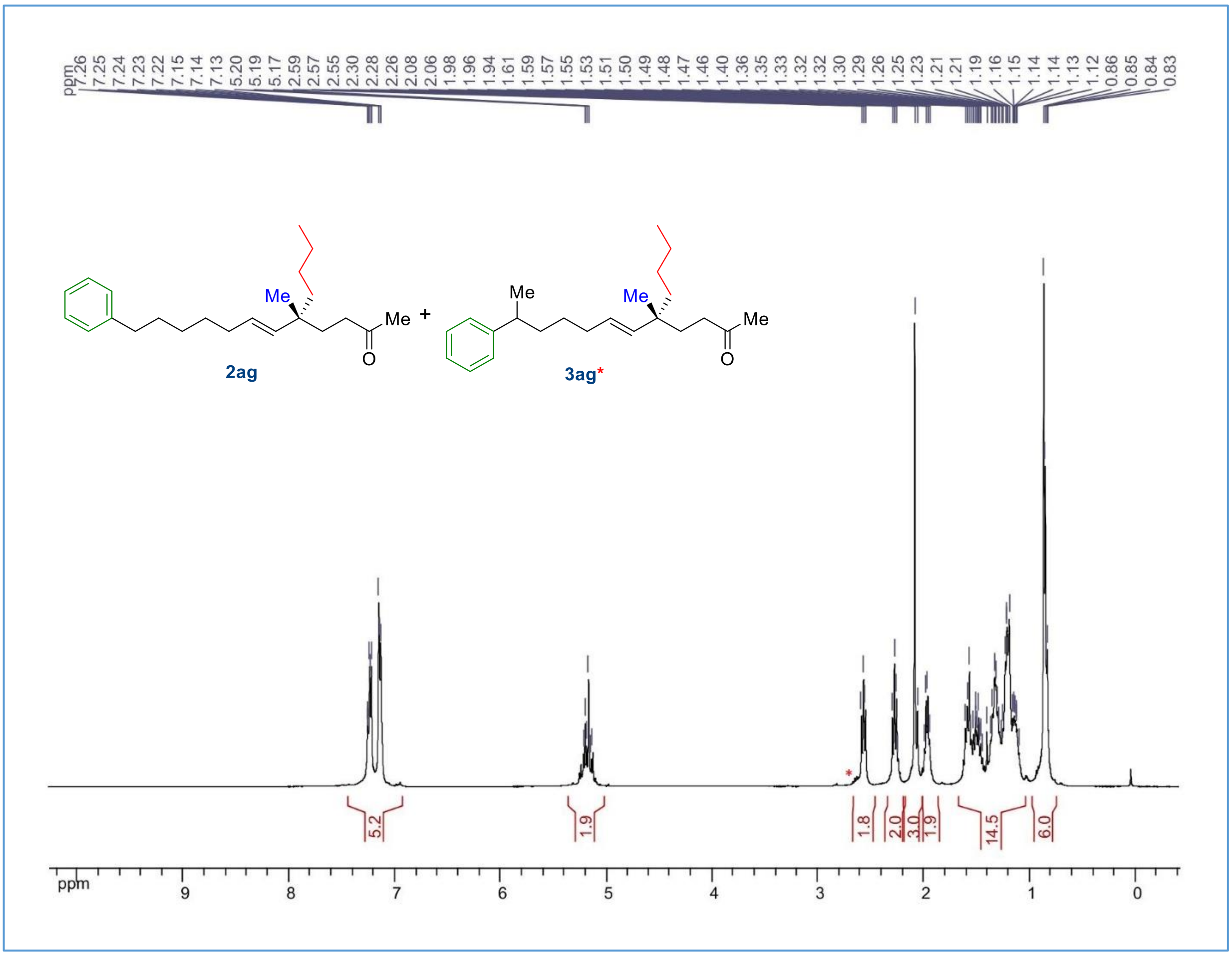

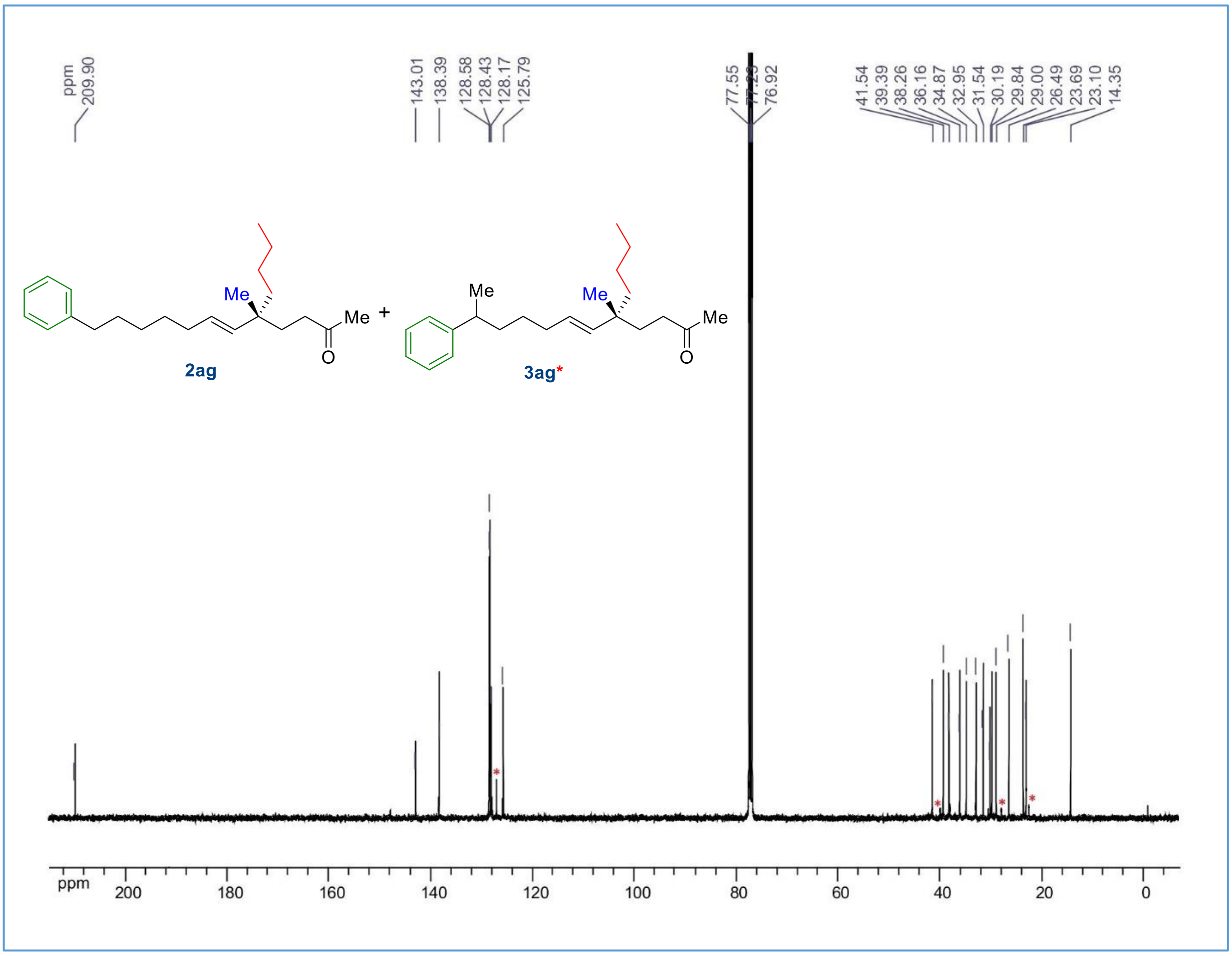

Supplementary Figure 75. ^{1}H and ^{13}C NMR spectra of compound **2ag**

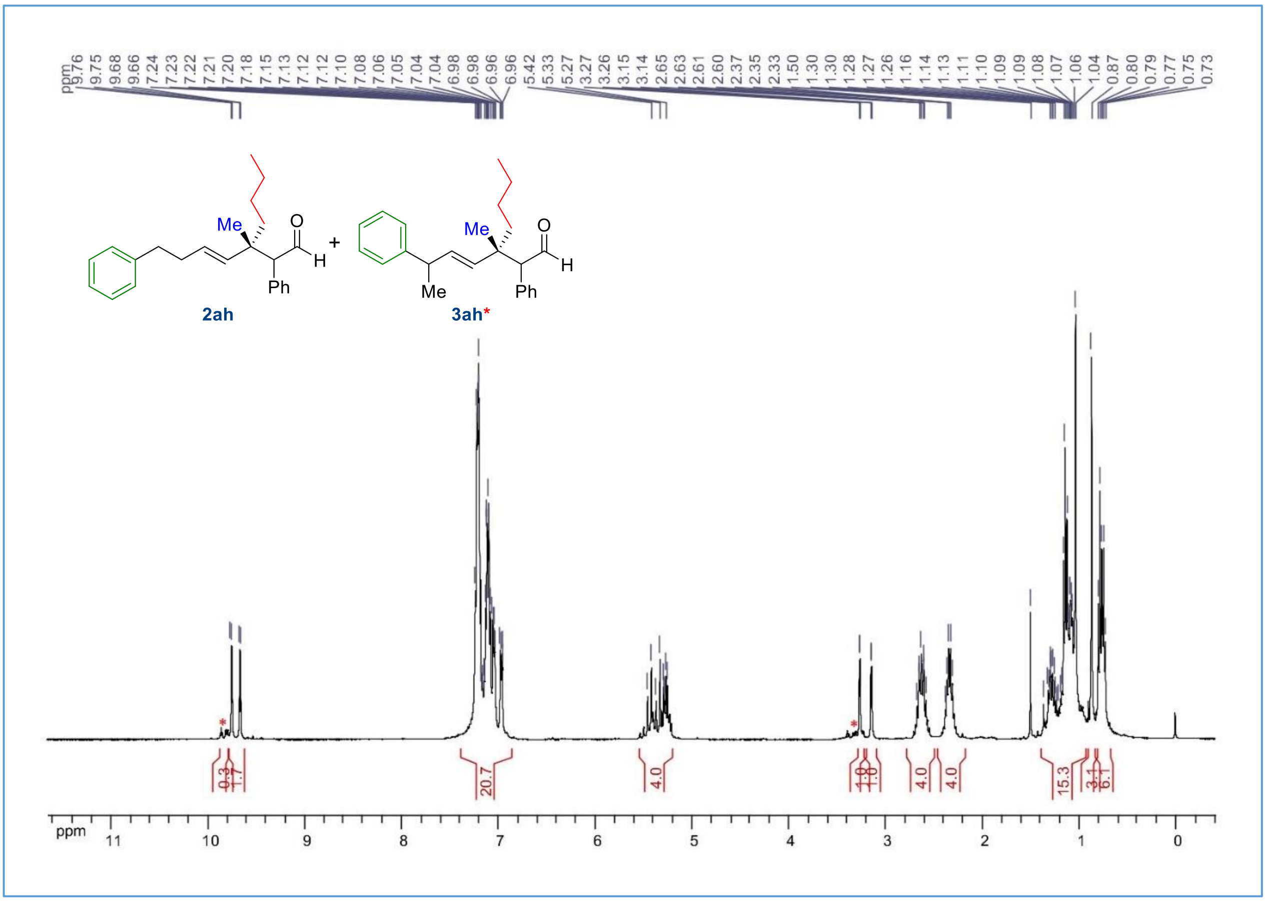

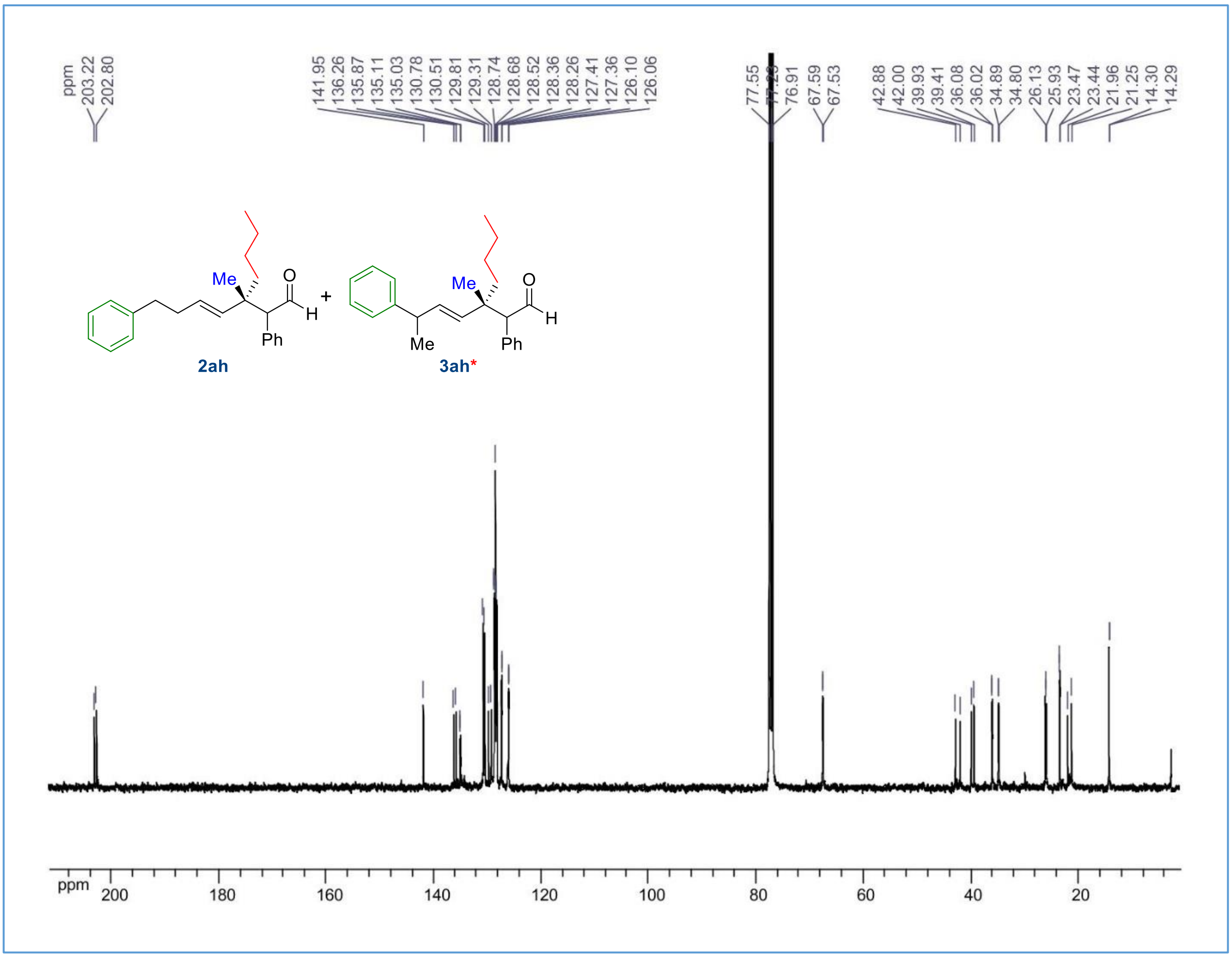

Supplementary Figure 76. ^{1}H and ^{13}C NMR spectra of compound **2ah**

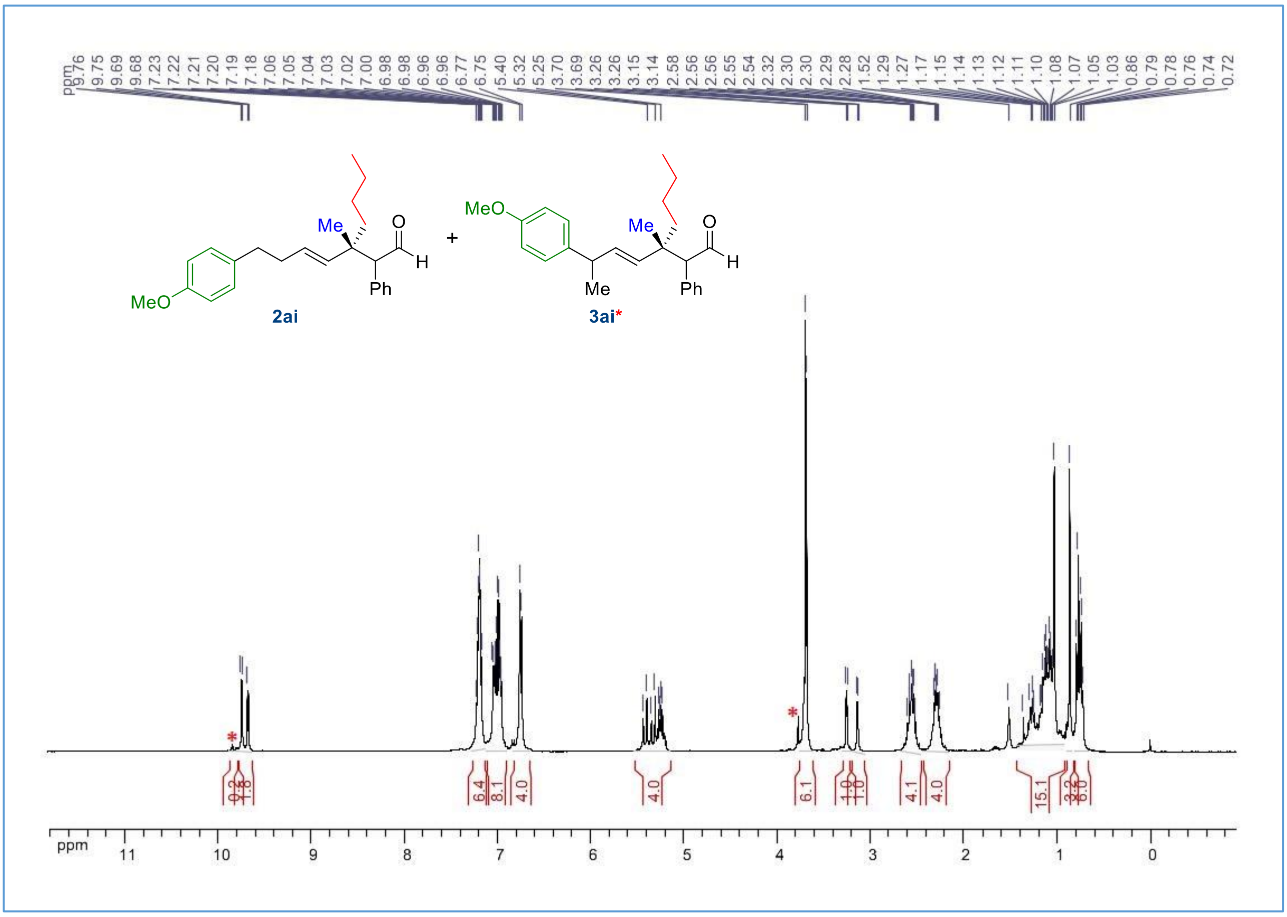

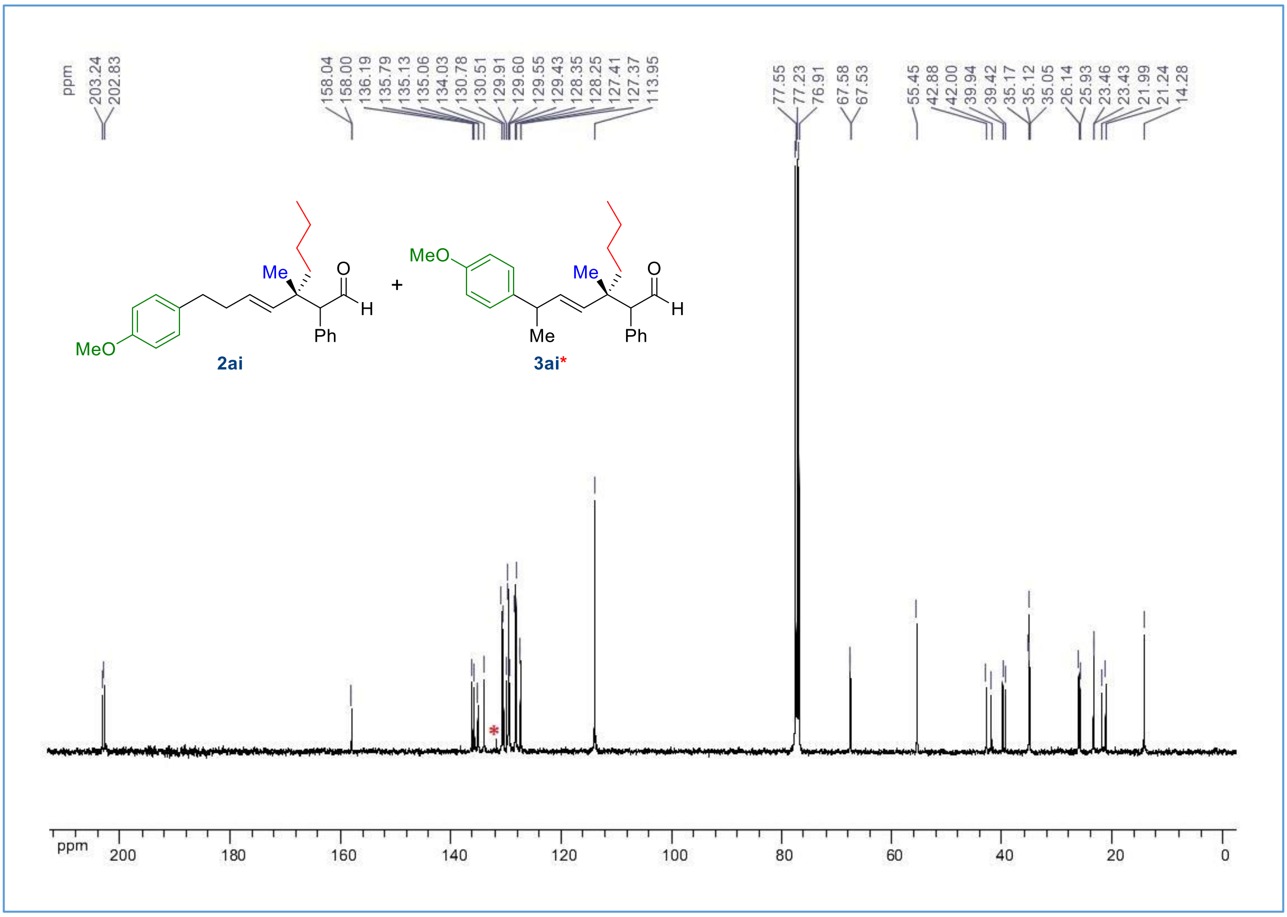

Supplementary Figure 77. ^{1}H and ^{13}C NMR spectra of compound **2ai**

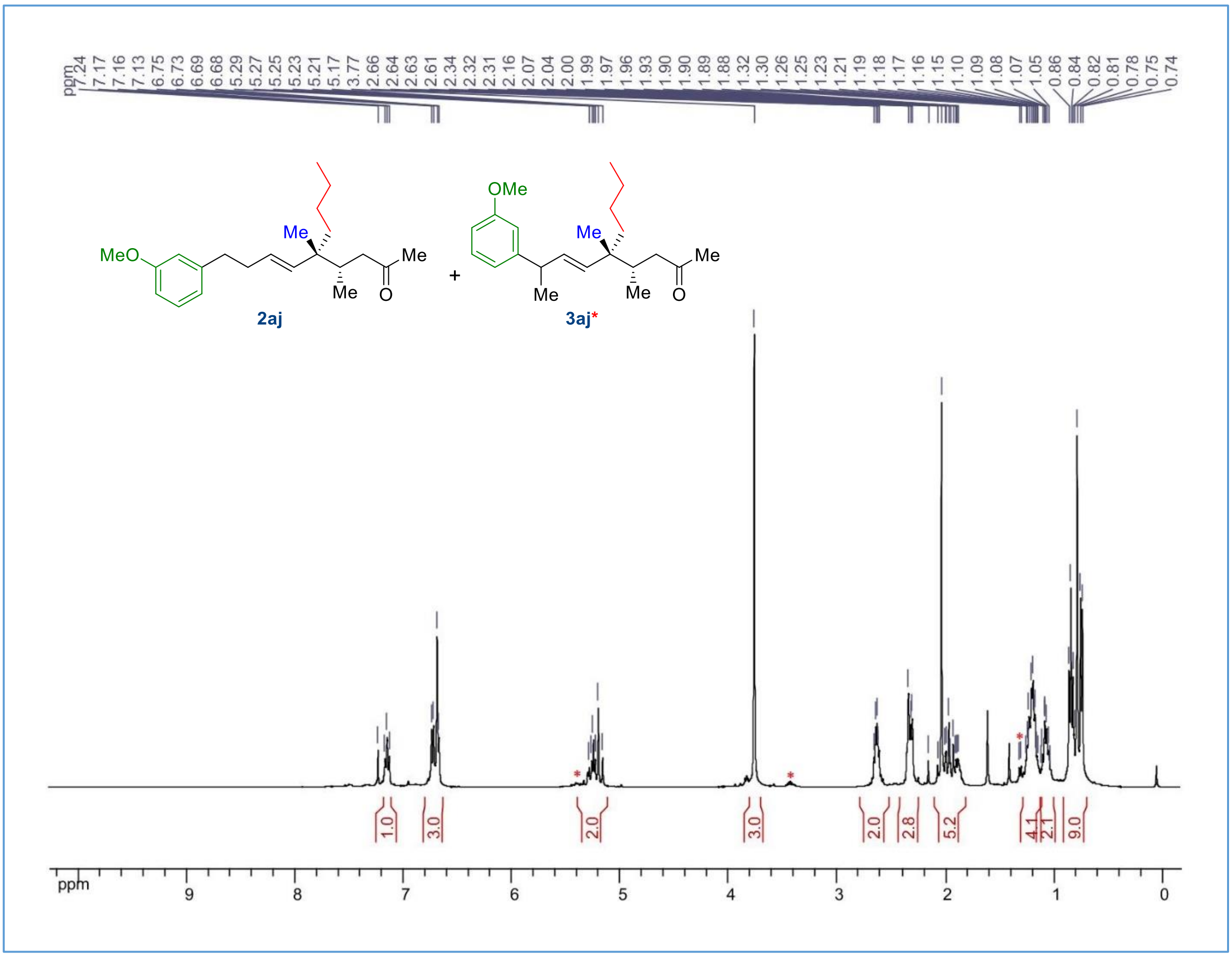

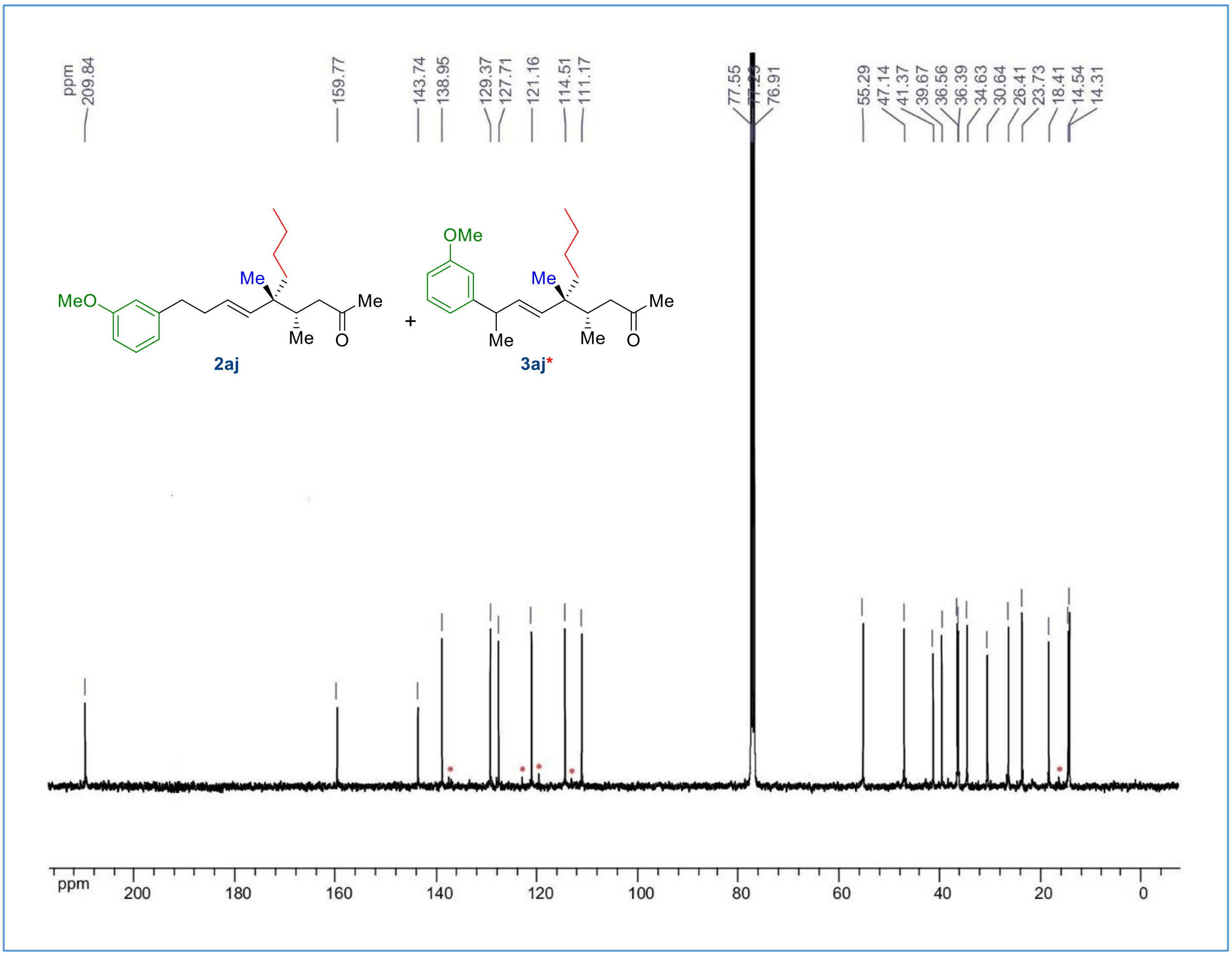

Supplementary Figure 78. ^{1}H and ^{13}C NMR spectra of compound **2aj**

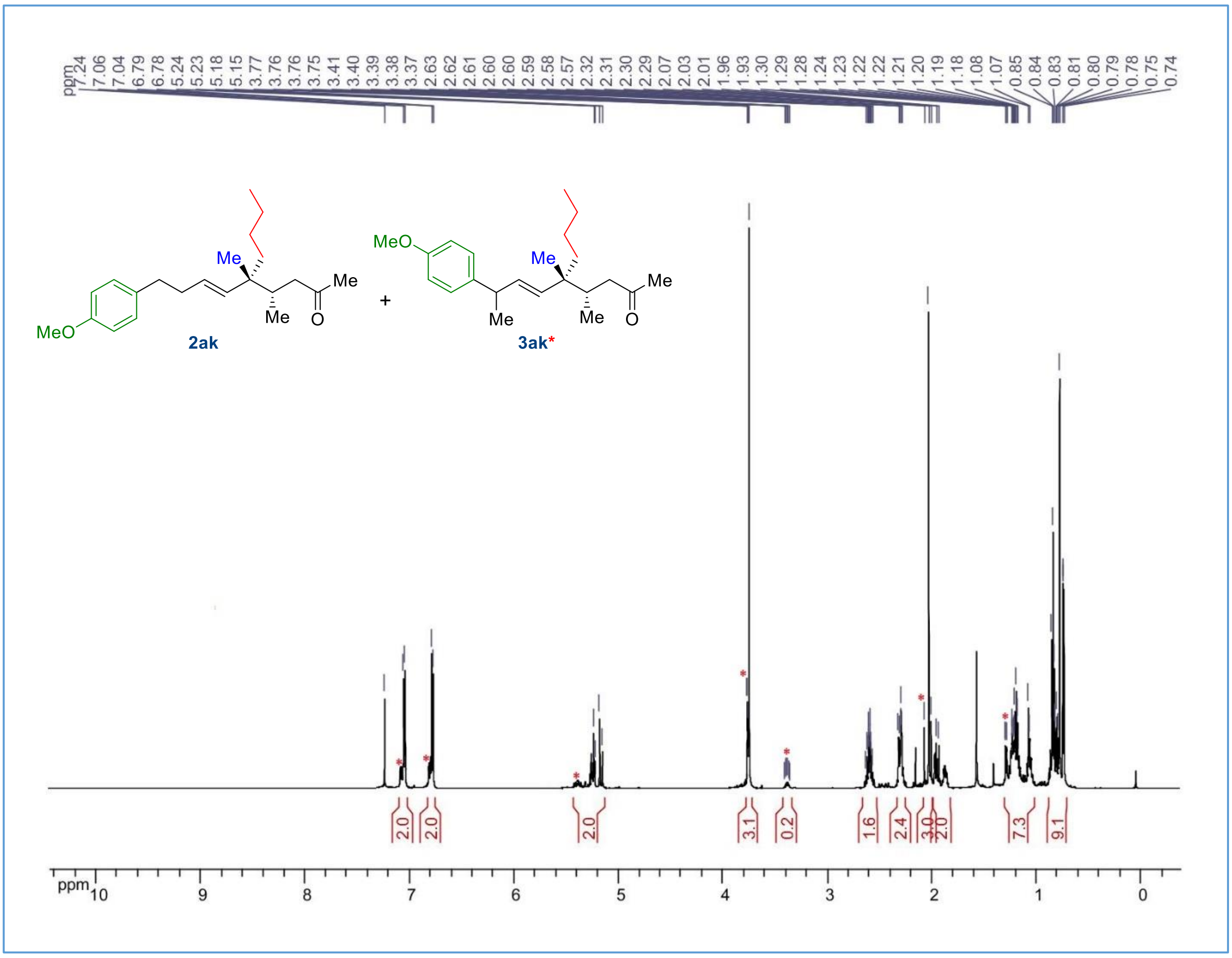

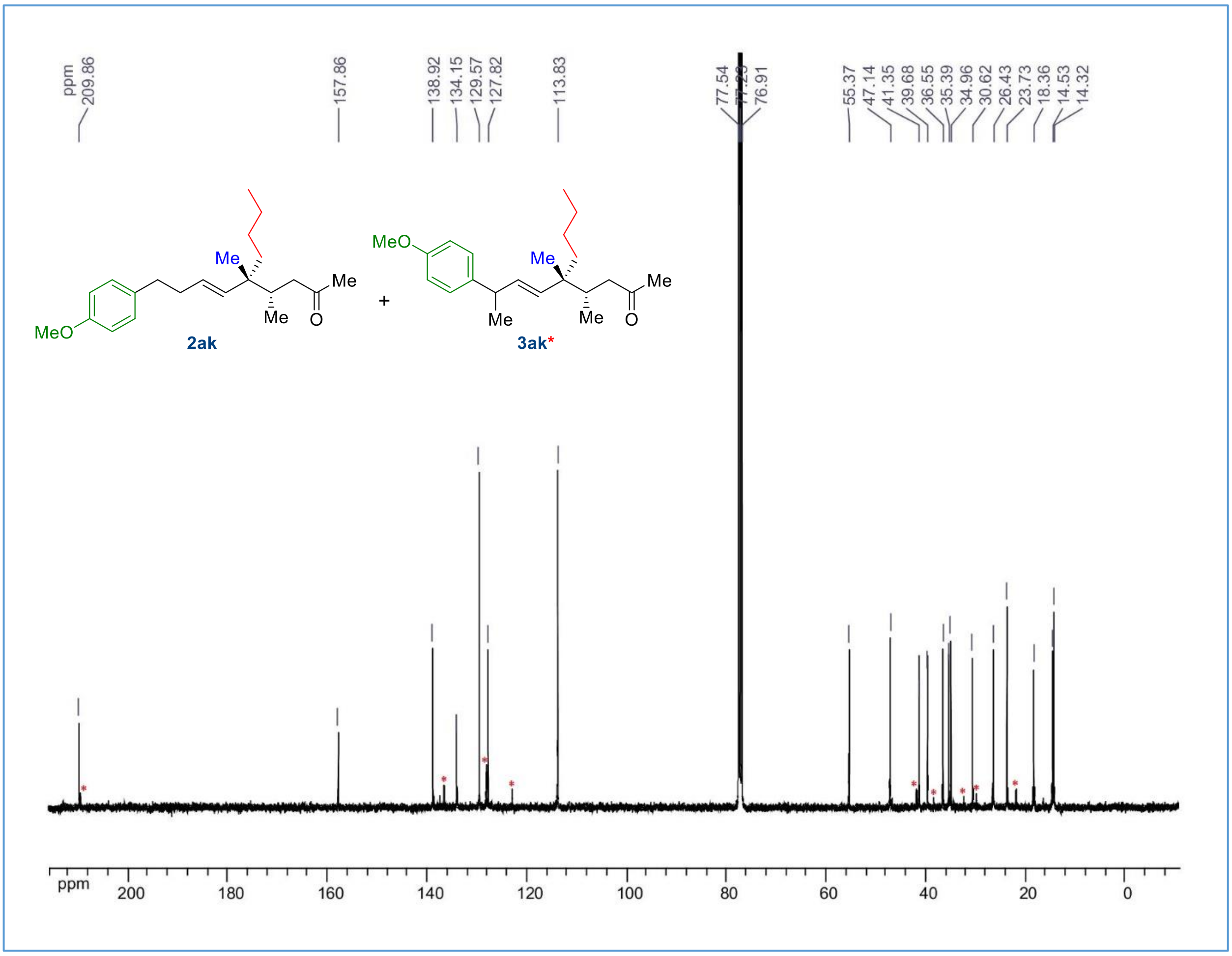

Supplementary Figure 79. ^{1}H and ^{13}C NMR spectra of compound **2ak**

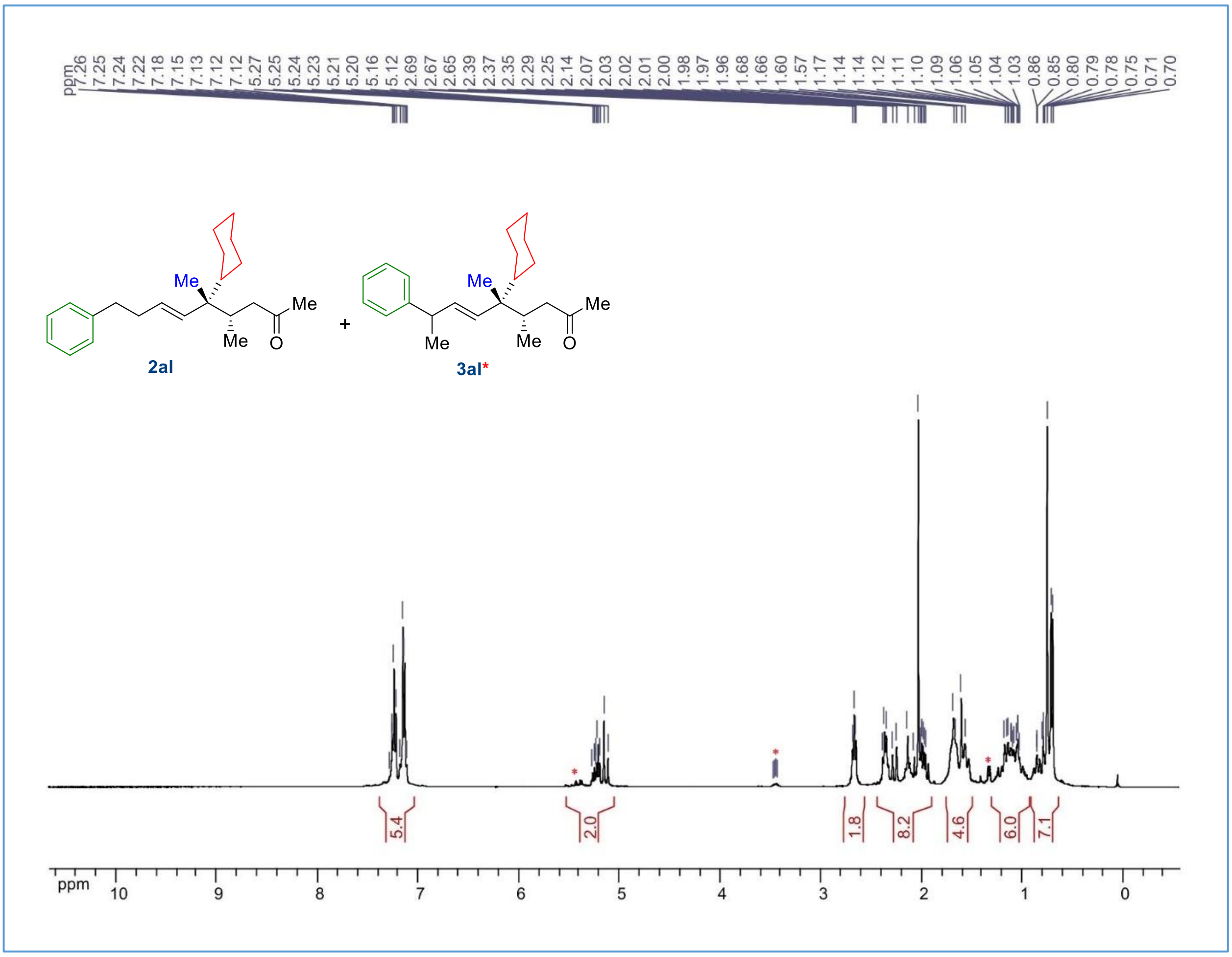

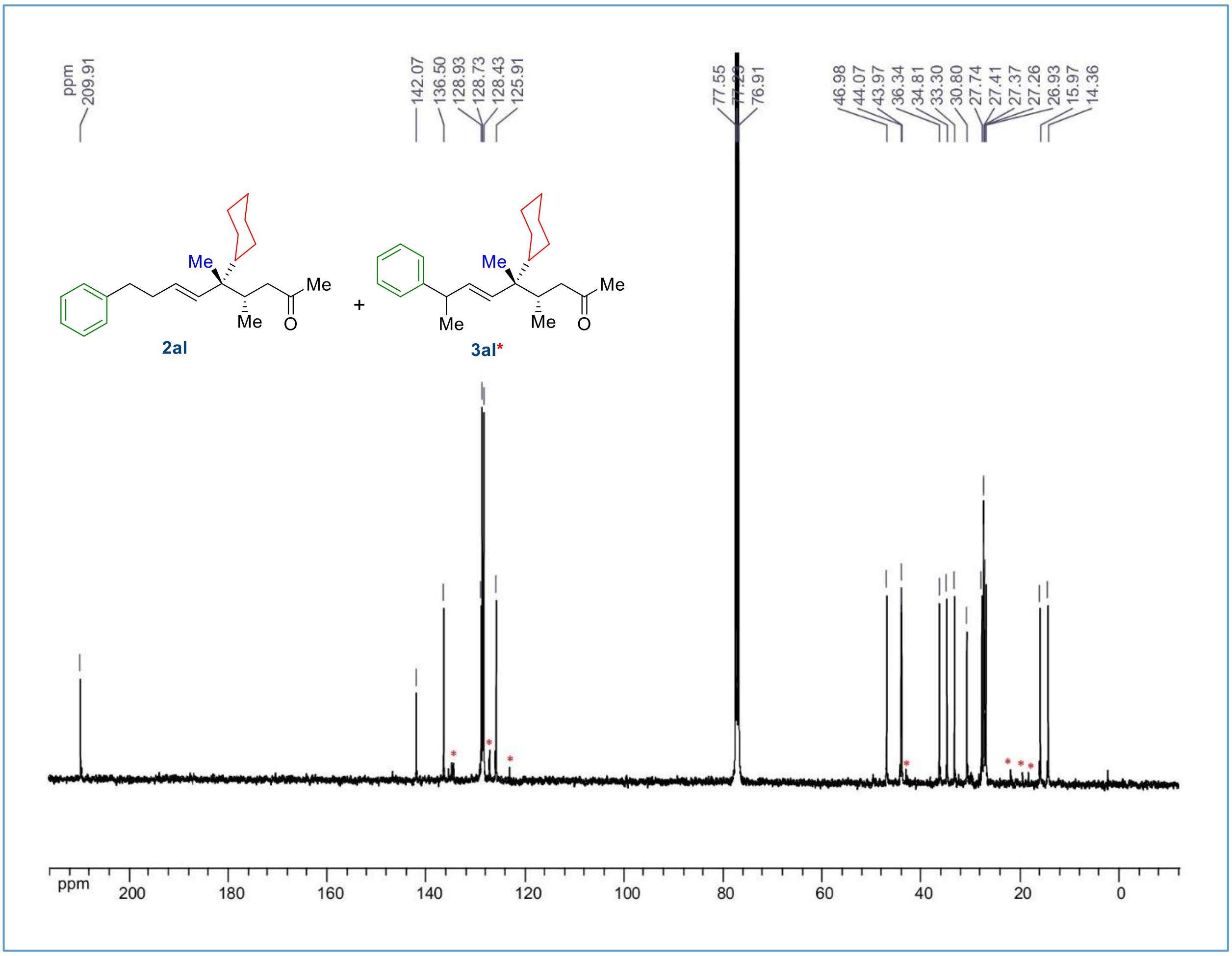

Supplementary Figure 80. ^{1}H and ^{13}C NMR spectra of compound **2al**

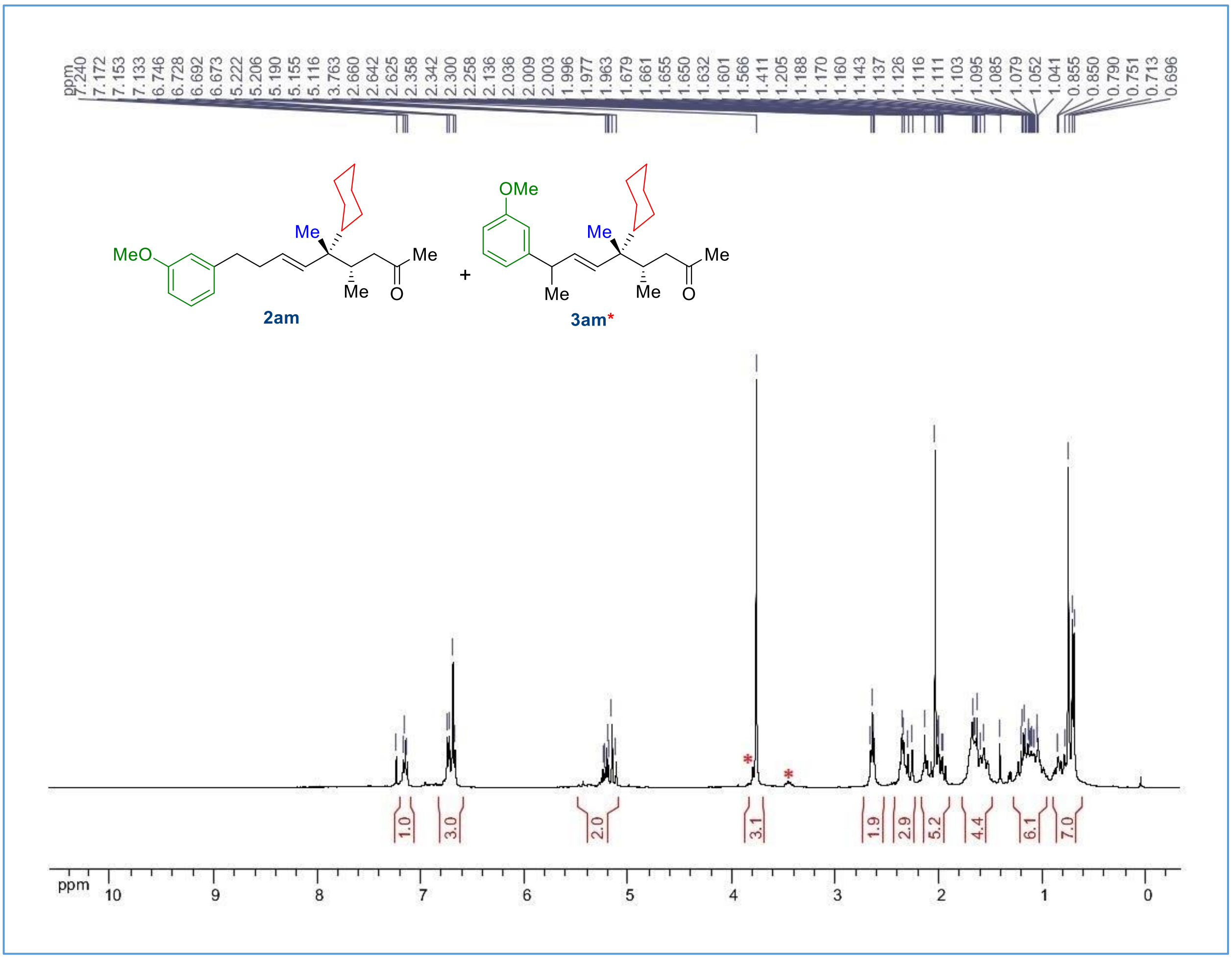

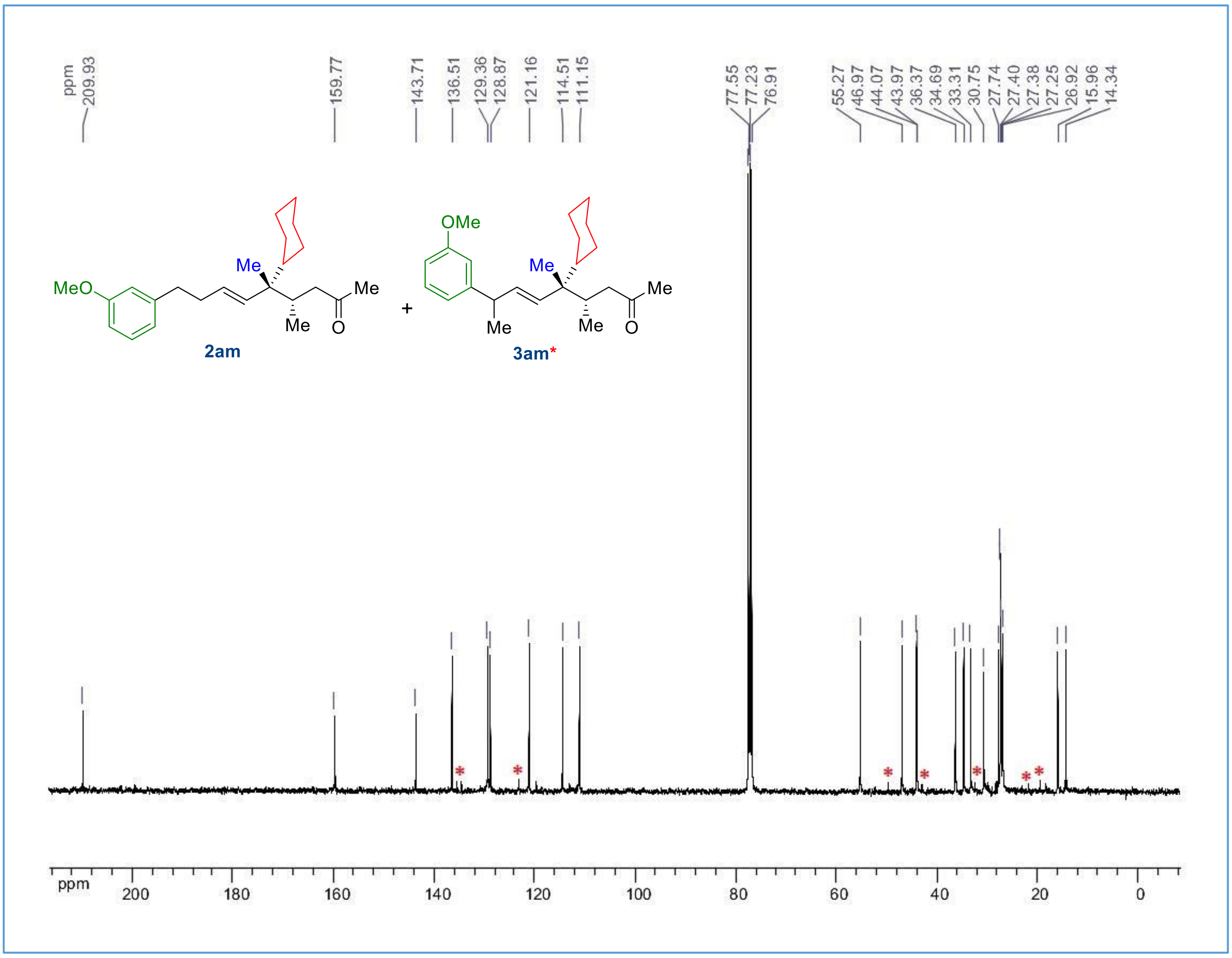

Supplementary Figure 81. ^{1}H and ^{13}C NMR spectra of compound **2am**

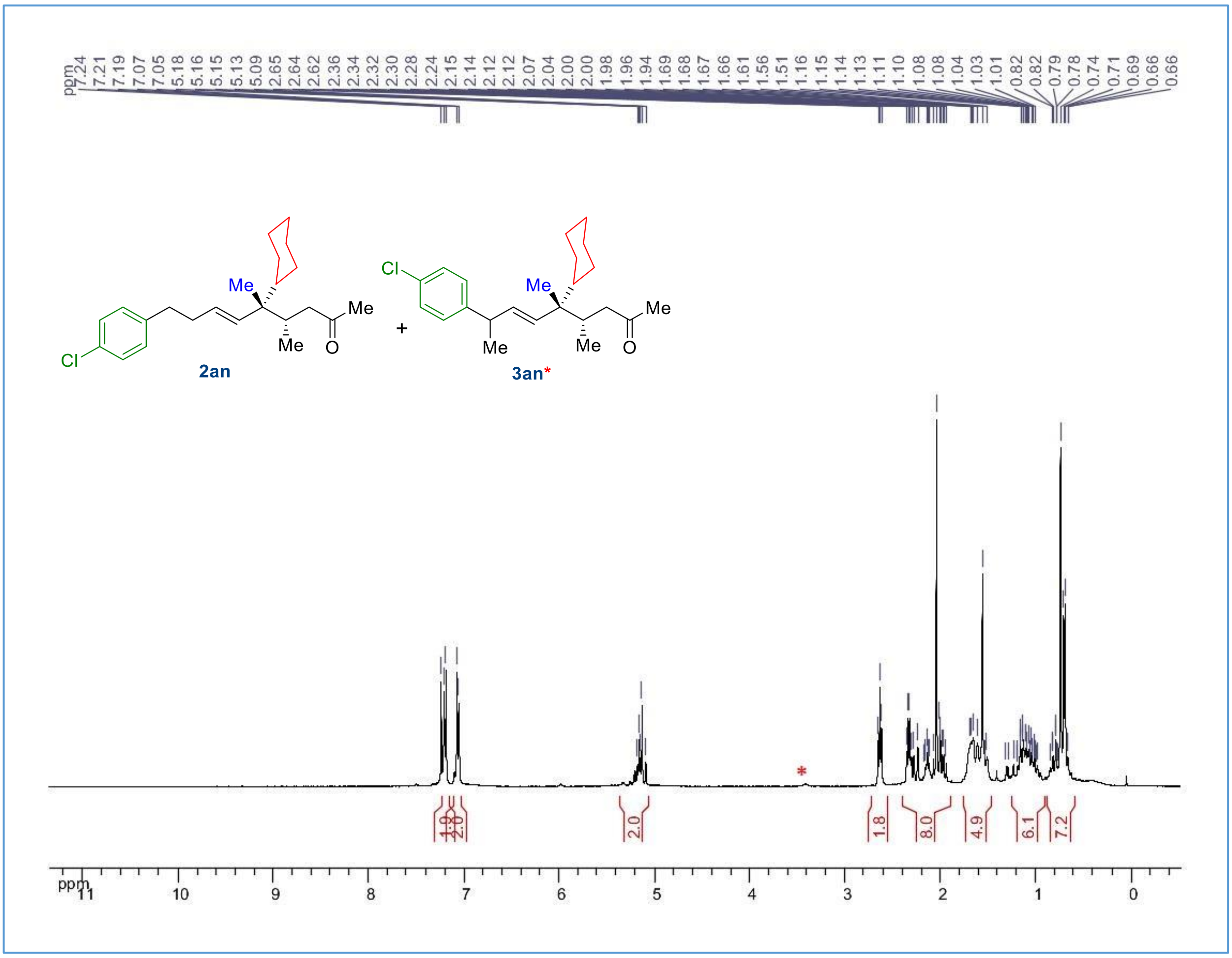

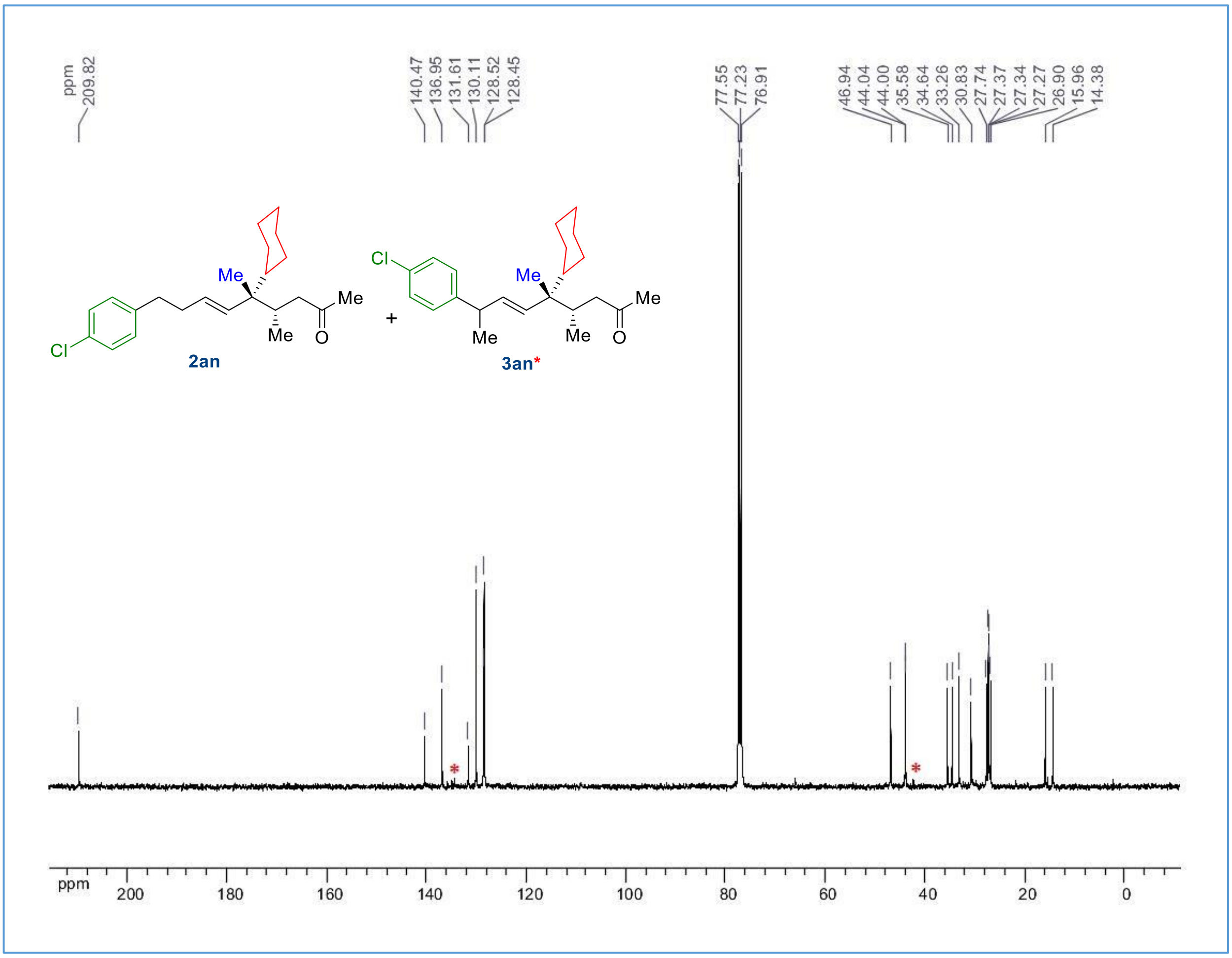

Supplementary Figure 82. ^{1}H and ^{13}C NMR spectra of compound **2an**

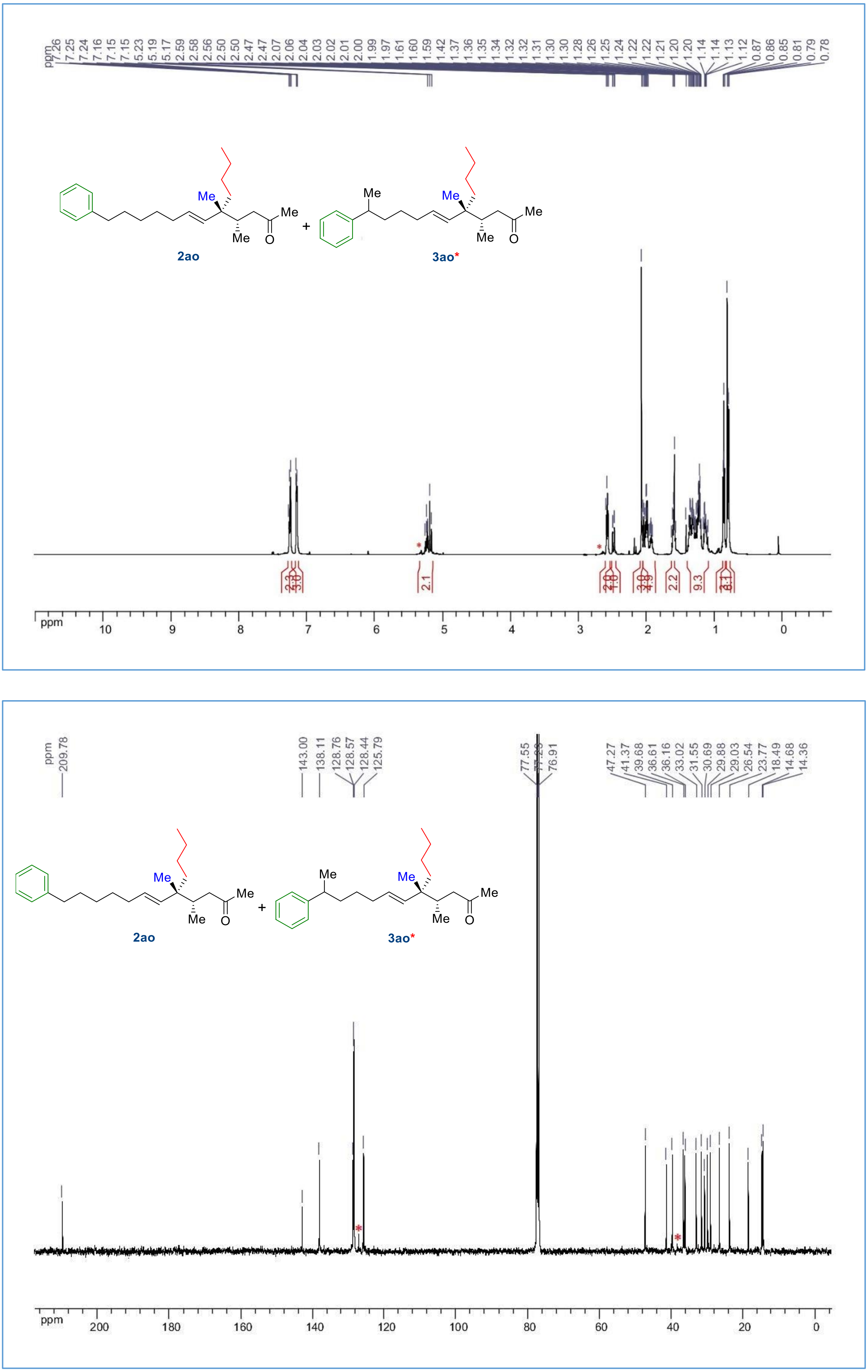

Supplementary Figure 83. ^{1}H and ^{13}C NMR spectra of compound **2ao**

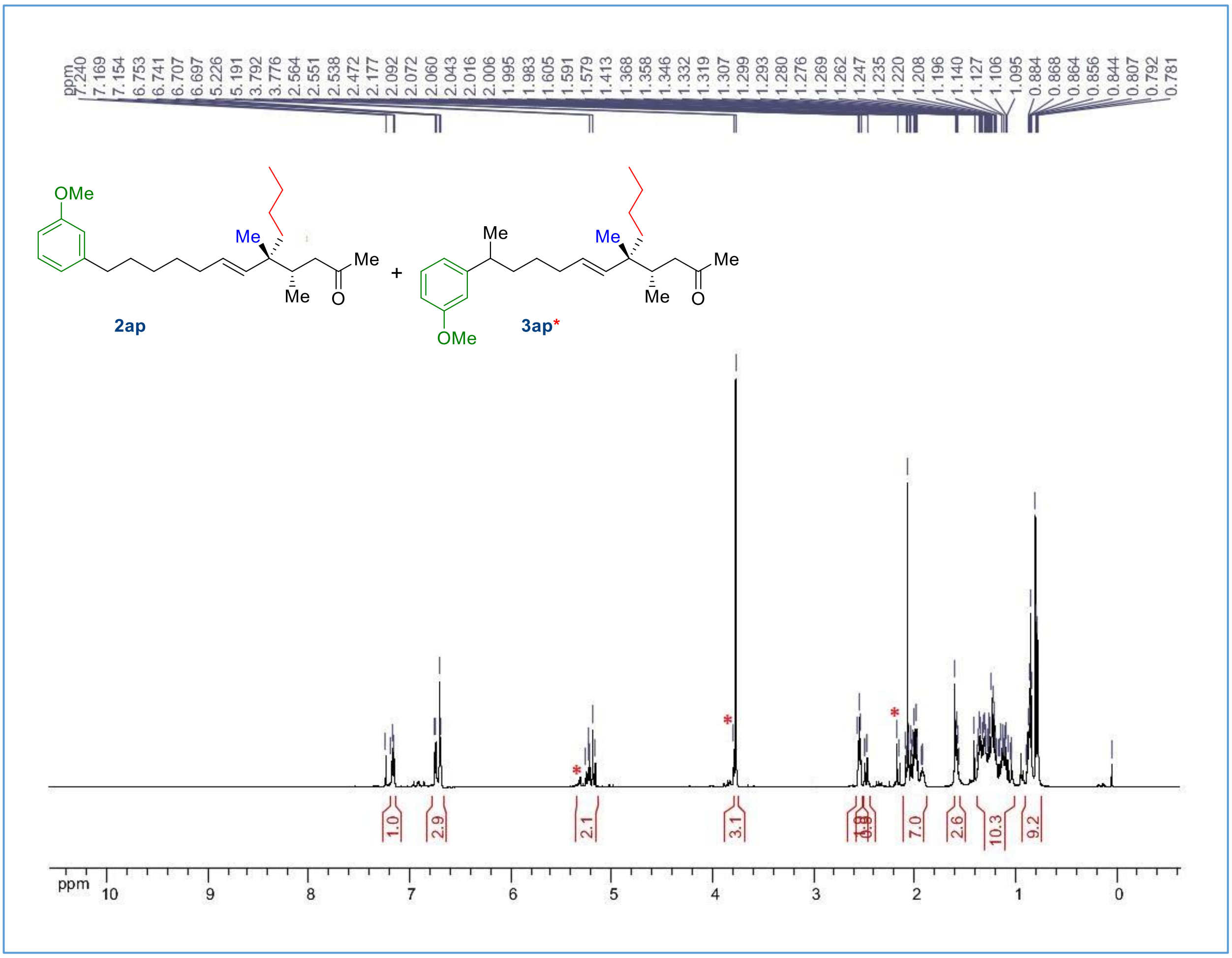

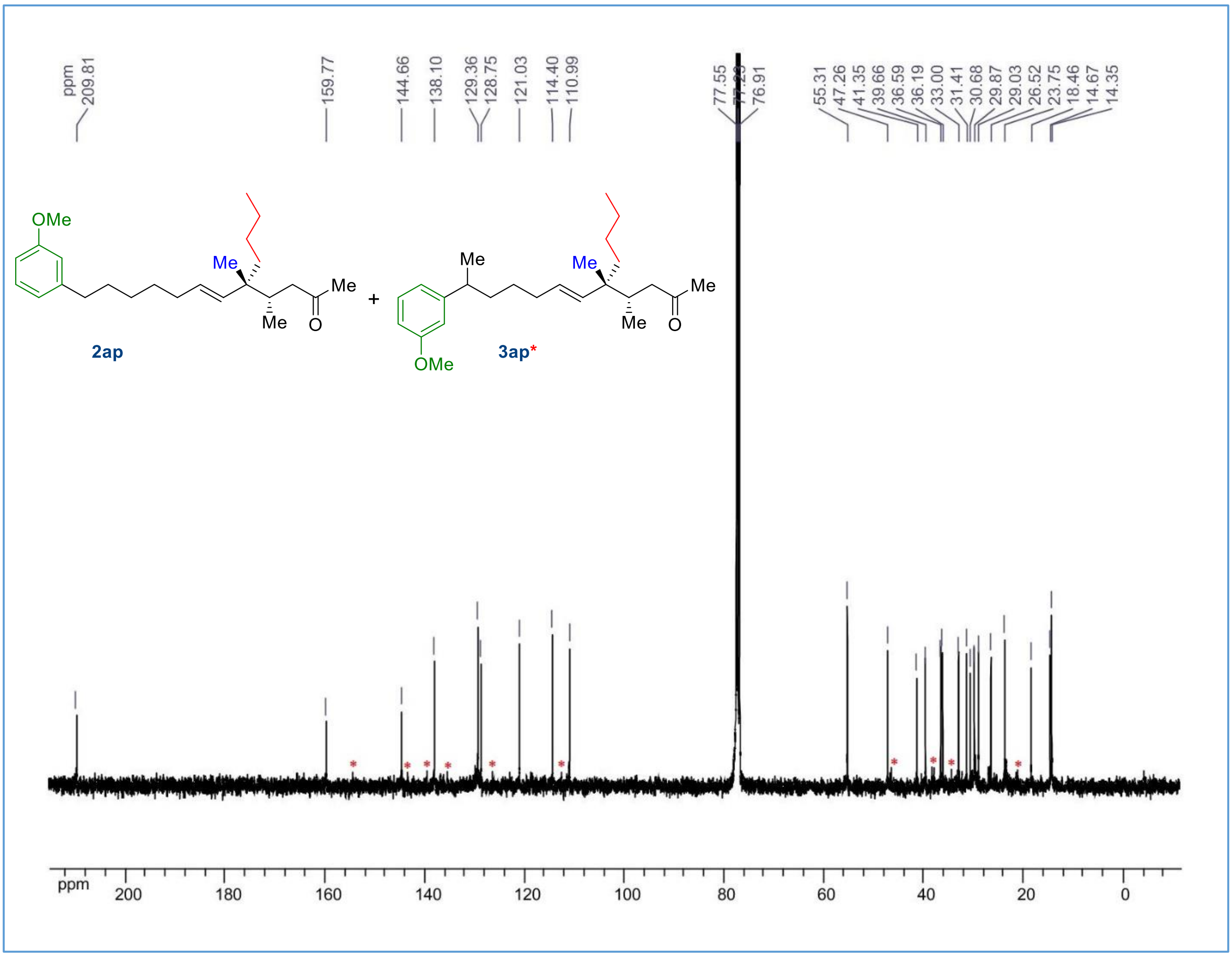

Supplementary Figure 84. ^{1}H and ^{13}C NMR spectra of compound **2ap**

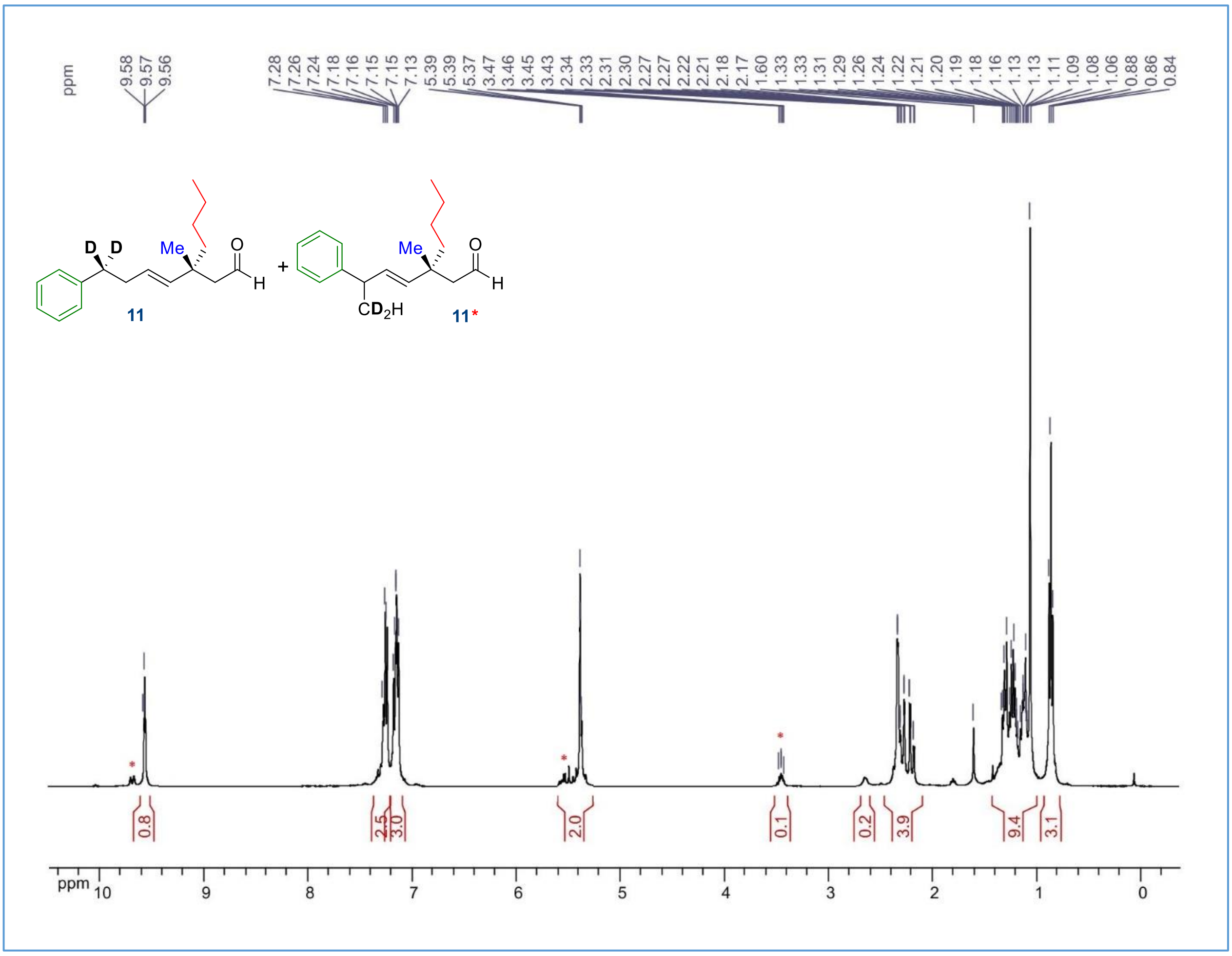

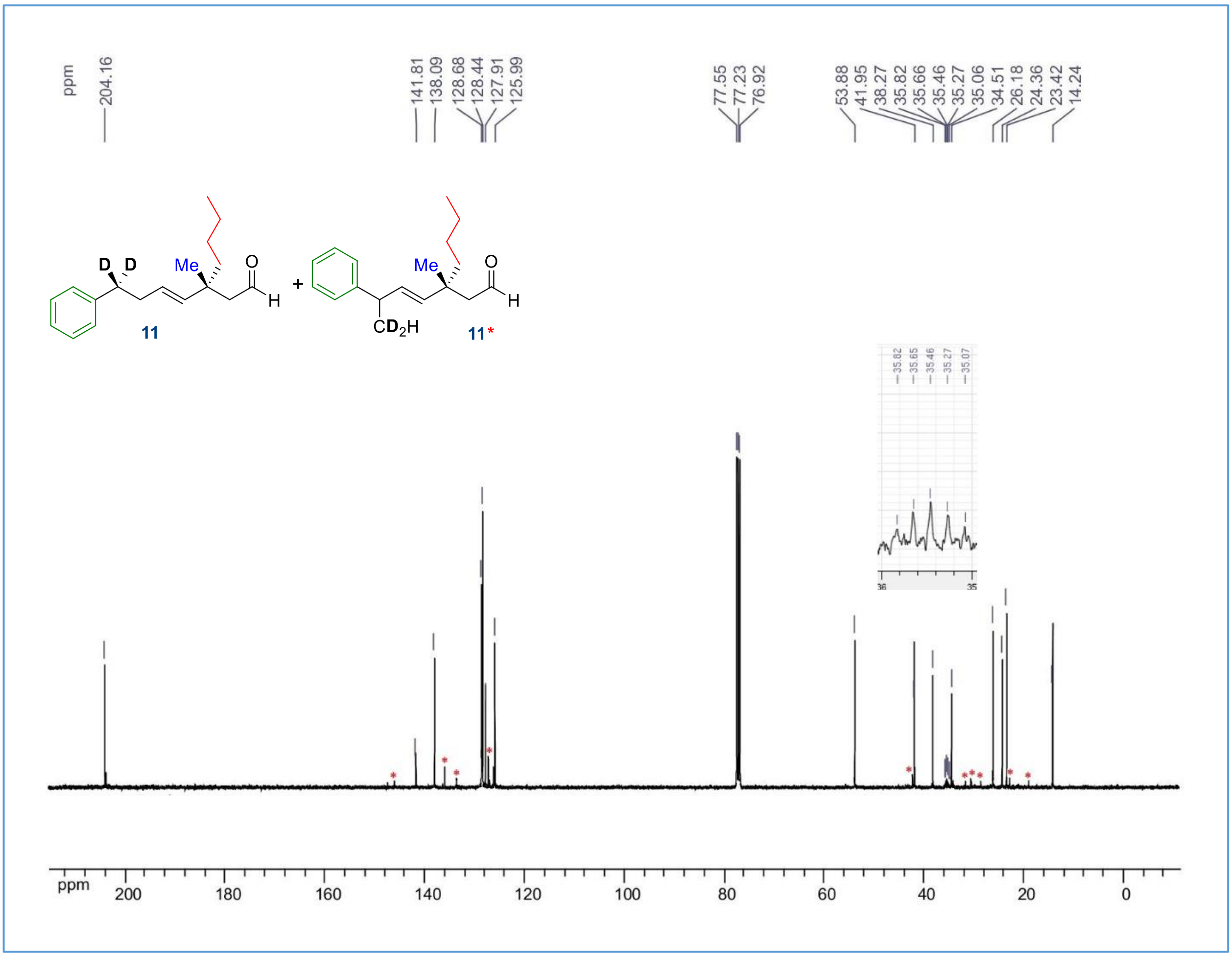

Supplementary Figure 85. ^{1}H and ^{13}C NMR spectra of compound **11**

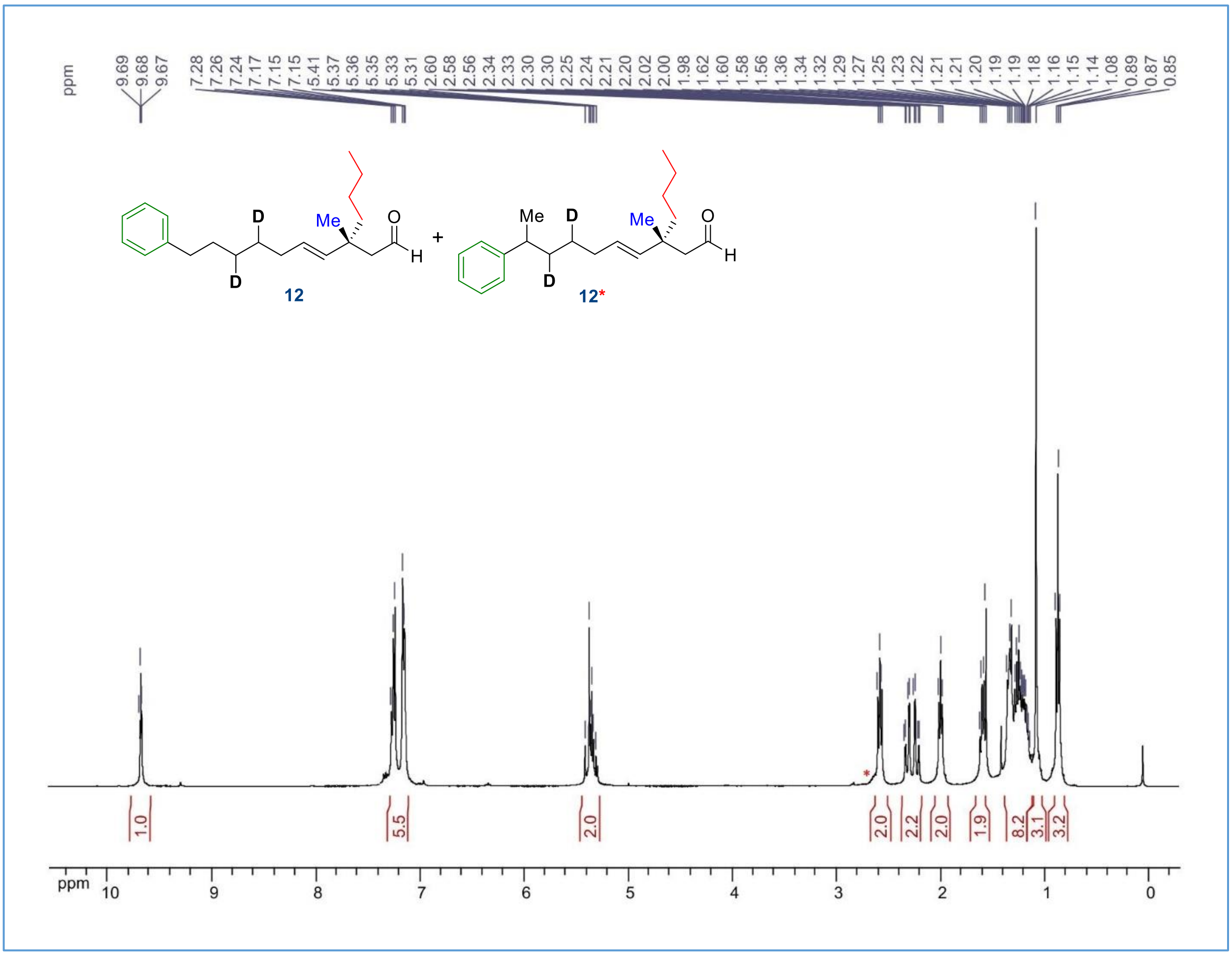

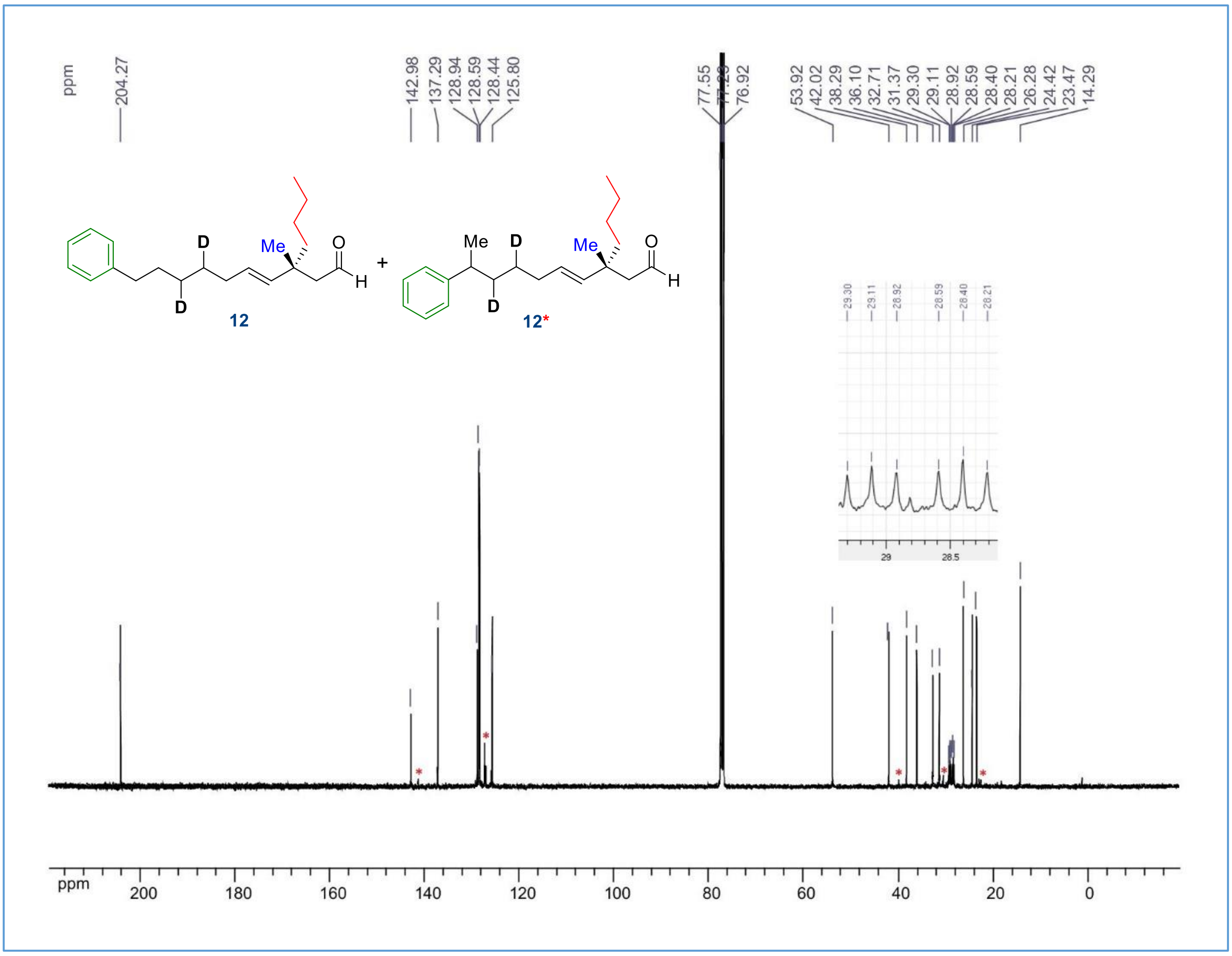

Supplementary Figure 86. ^{1}H and ^{13}C NMR spectra of compound **12**

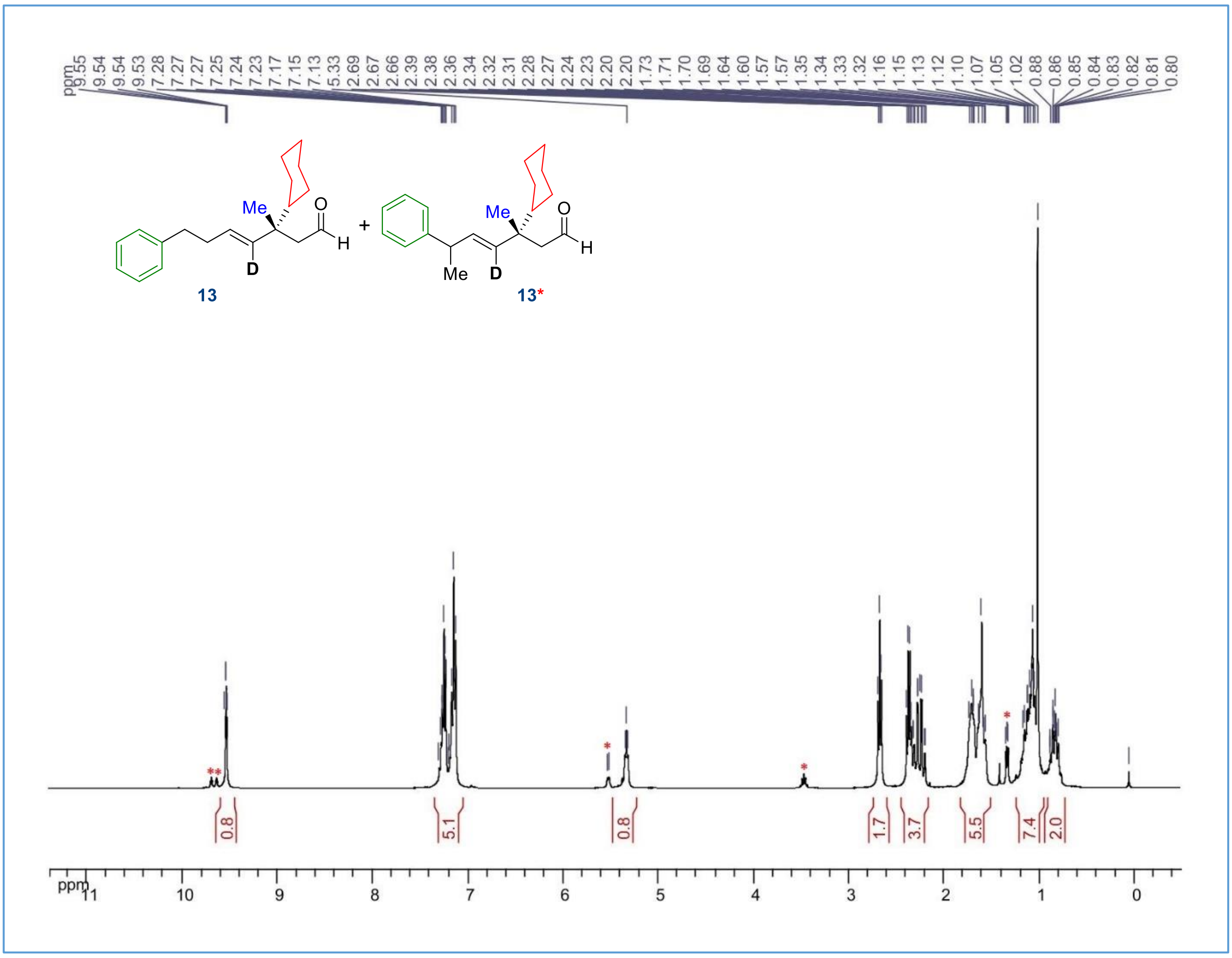

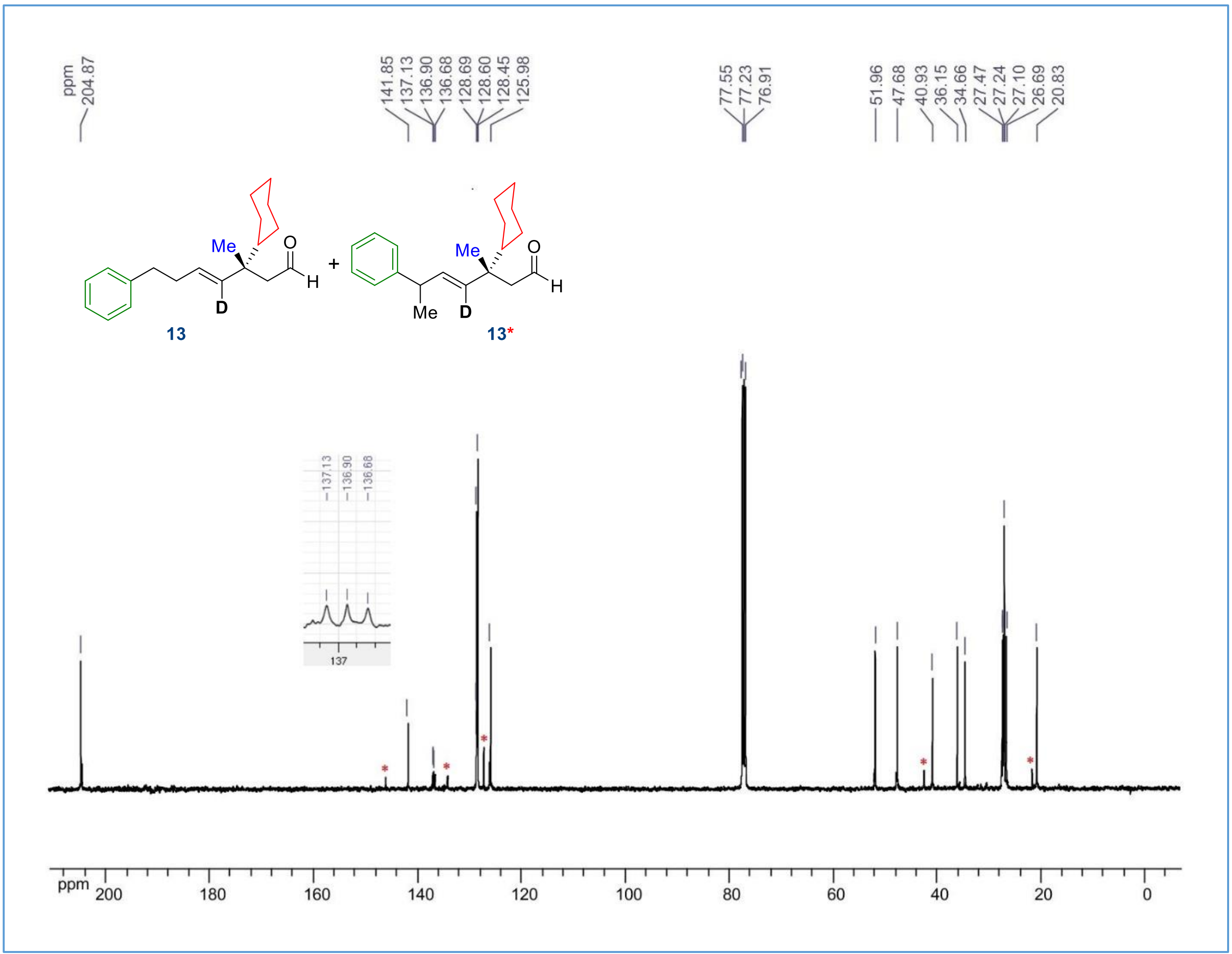

Supplementary Figure 87. ^{1}H and ^{13}C NMR spectra of compound **13**

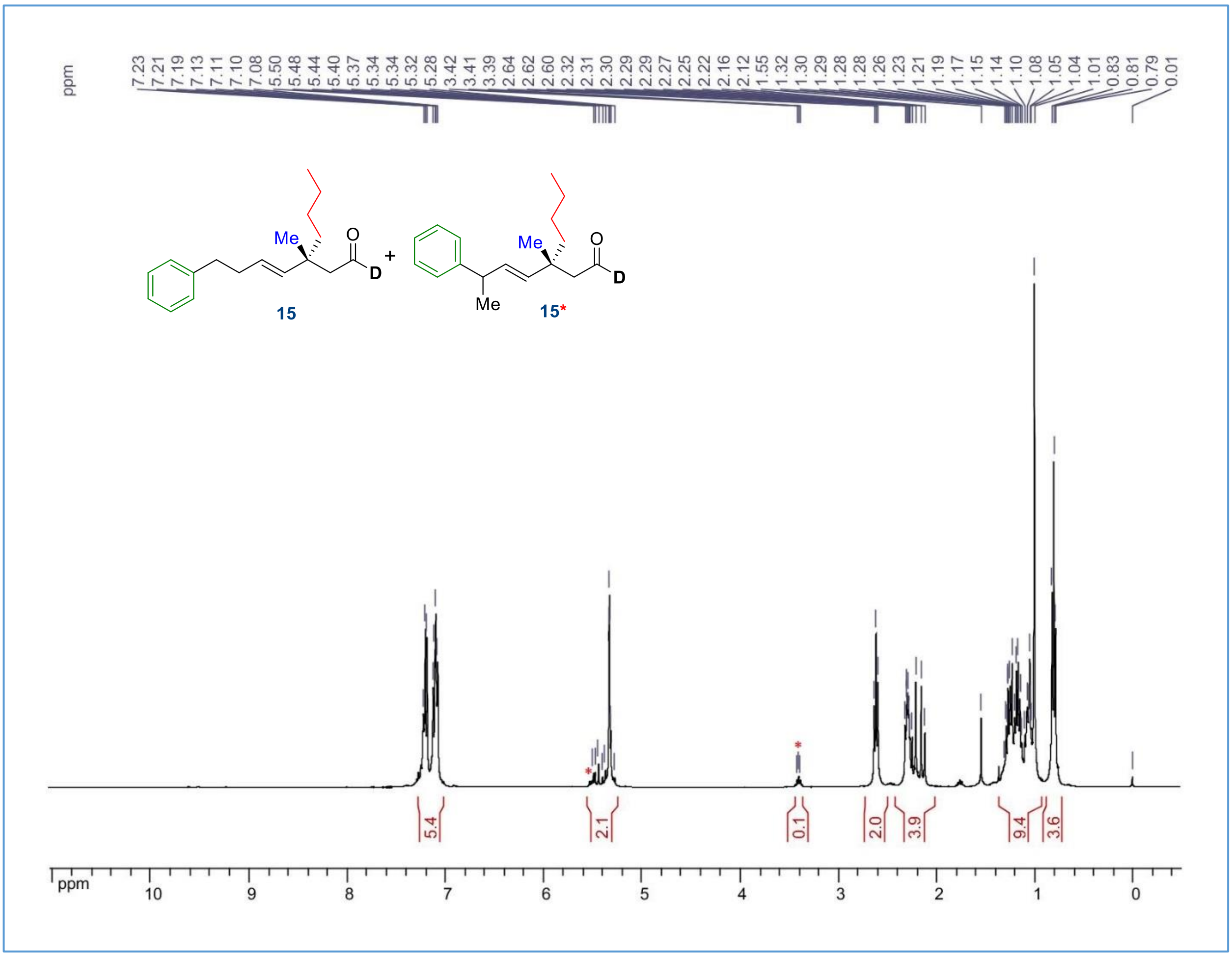

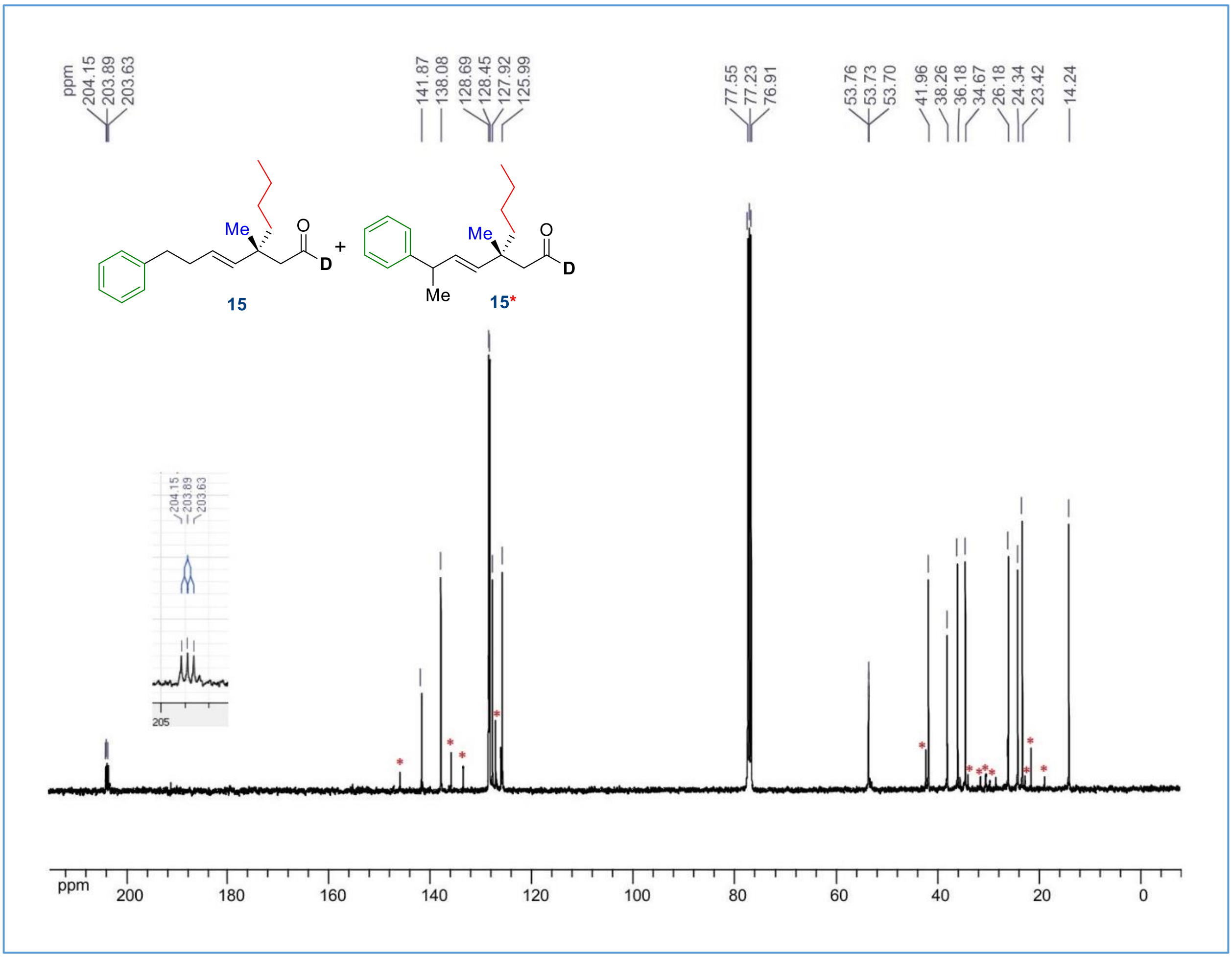

Supplementary Figure 88. ^{1}H and ^{13}C NMR spectra of compound **15**

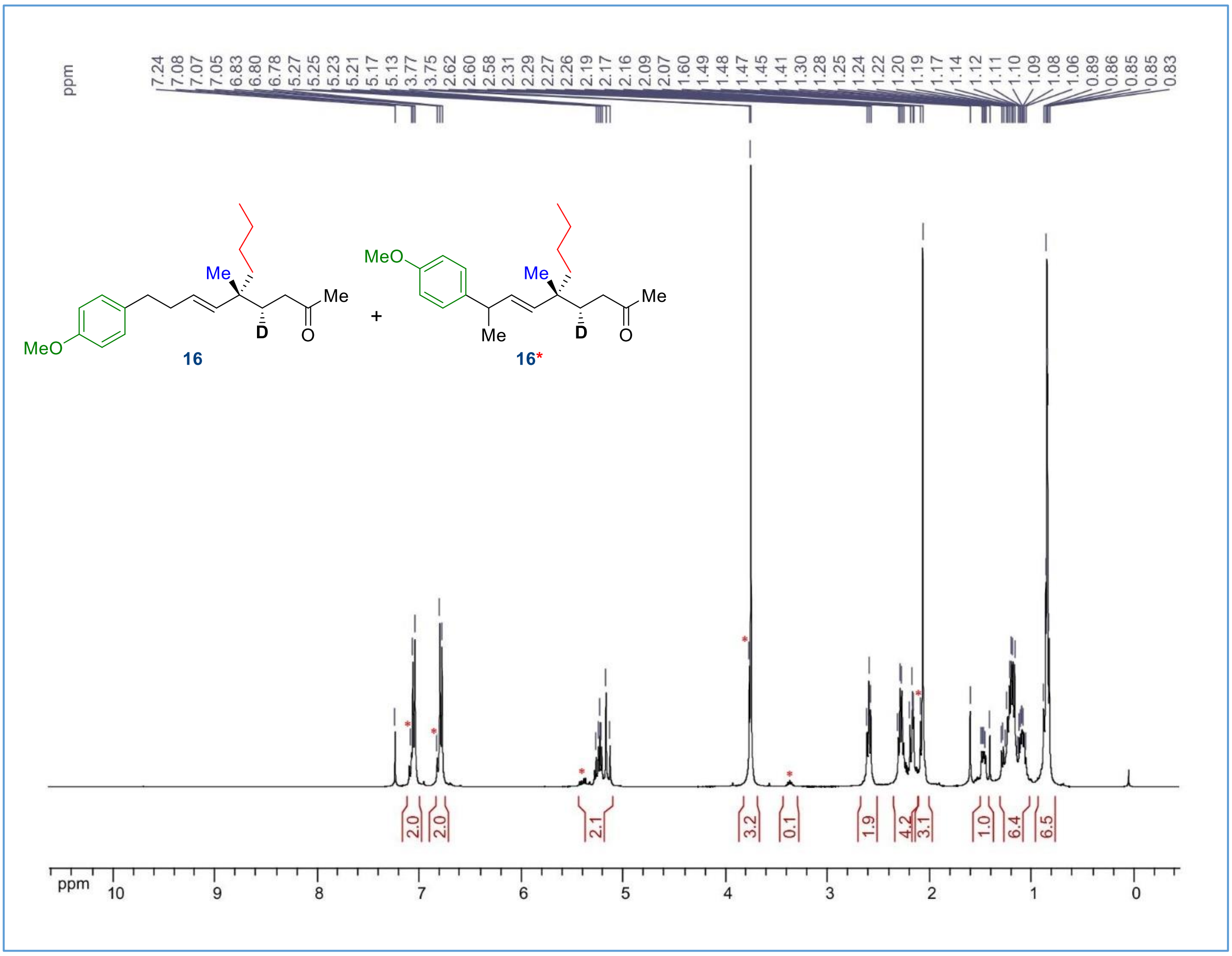

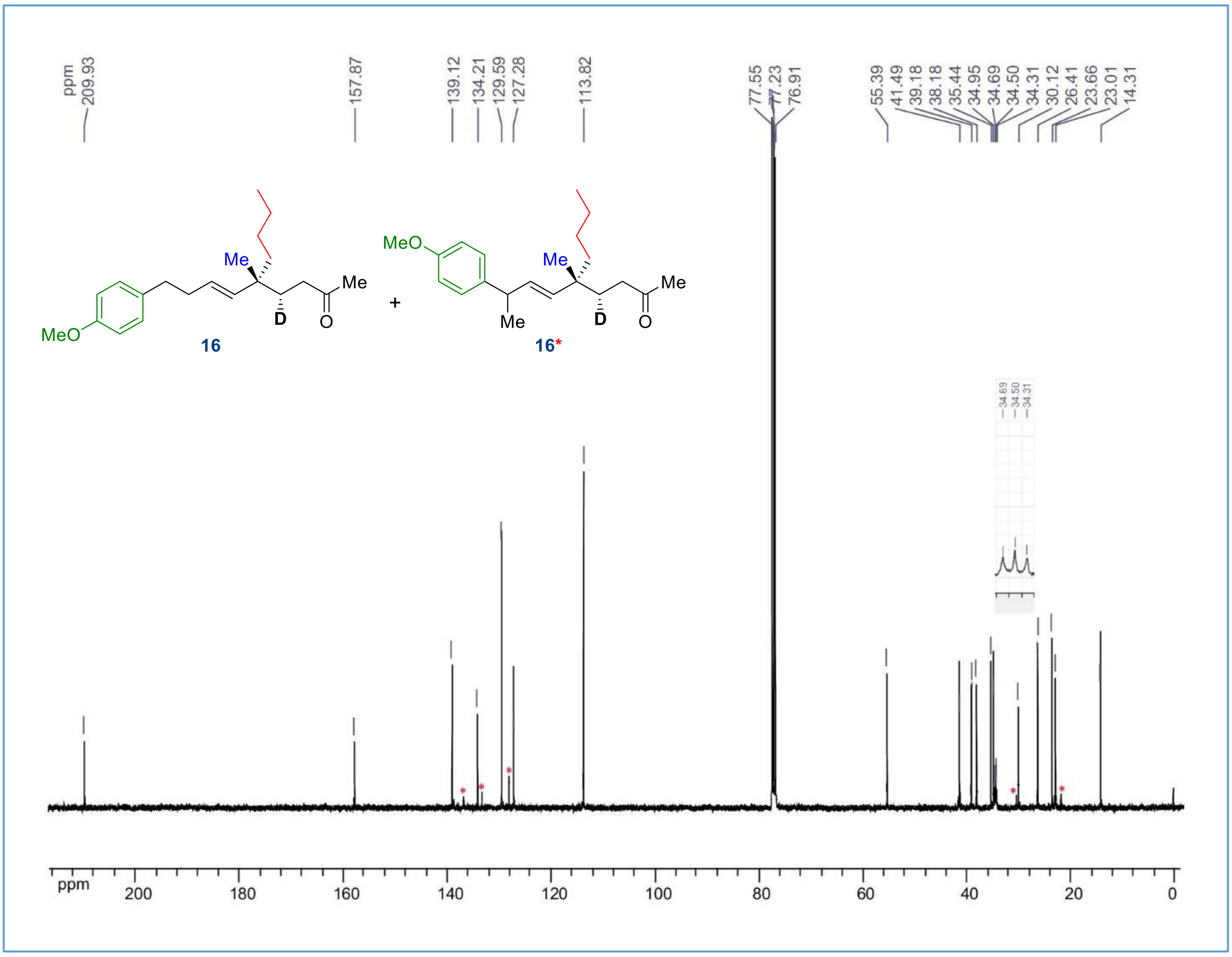

Supplementary Figure 89. ^{1}H and ^{13}C NMR spectra of compound **16**

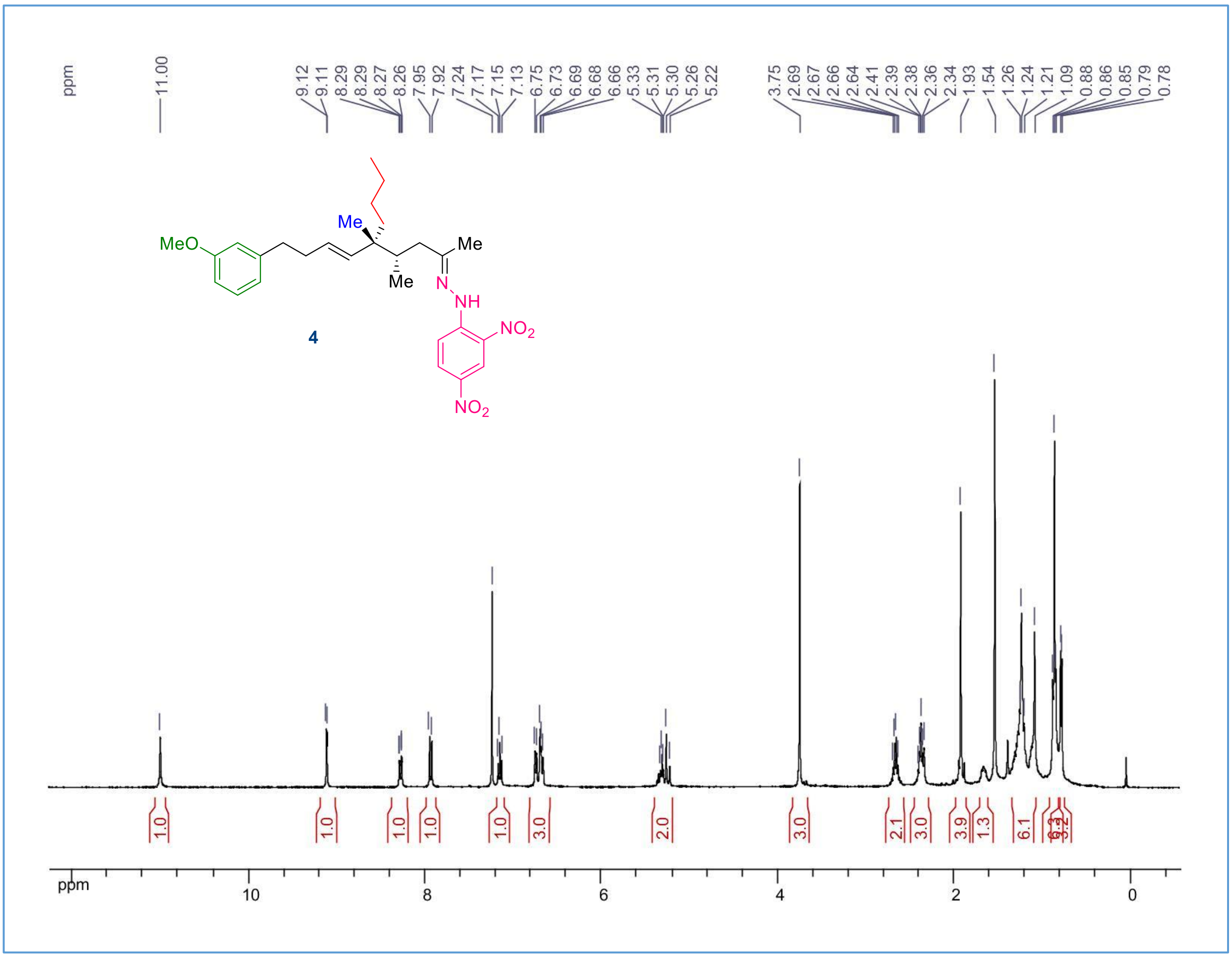

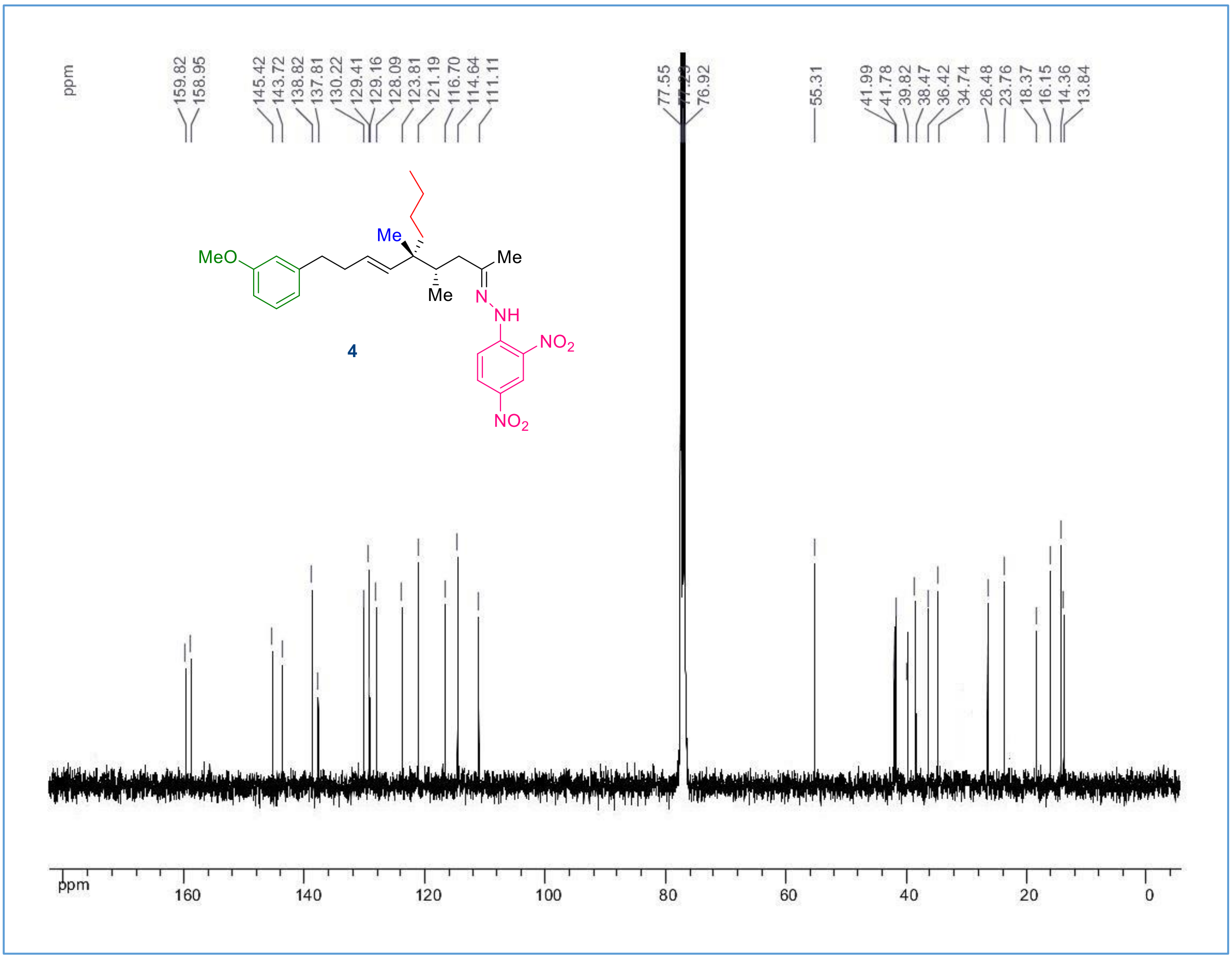

Supplementary Figure 90. ^{1}H and ^{13}C NMR spectra of compound **4**

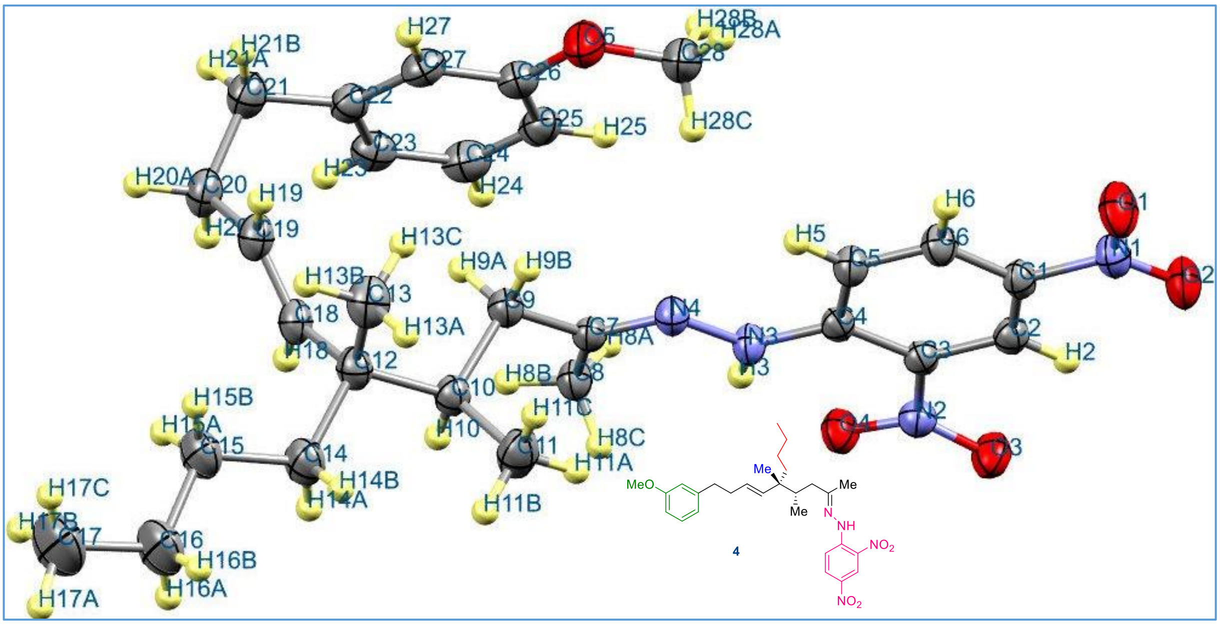

Supplementary Figure 91. XRD crystal structure of compound **4** drawn at 30% probability level

Supplementary Methods

General information

Unless stated otherwise, reactions were conducted in flame-dried glassware under a positive pressure of argon. Ether and THF were dried from Pure-Solv® Purification System (Innovative Technology©). All other commercially obtained reagents were used as received. Dichloromethane was distilled from CaH_2. Copper iodide, rhodium acetate dimer, methyllithium (1.6 M in diethyl ether), butyllithium (1.6 M in hexane), were purchased from Aldrich. Methylmagnesium bromide (3.0 M in diethyl ether) was purchased from Acros. Thin-layer chromatography (TLC) was conducted with Merck silica gel 60 F_{254} pre-coated plates (0.25 mm) and visualized by exposure to UV light (254 nm) or stained with anisaldehyde, phosphomolybdic acid, or potassium permanganate. Column chromatography was performed using Fluka silica gel 60Å (40-63mm, 230-400 mesh). ^{1}H-NMR and ^{13}C-NMR spectra were recorded on a Bruker© spectrometers DPX200, AV300 or AVIII400, using $CDCl_3$ (unless otherwise specified) as solvent. Chemical shifts are reported in parts per million (ppm) with respect to the residual solvent signal $CDCl_3$ (^{1}H NMR: δ = 7.24 ppm; ^{13}C NMR: δ = 77.23 ppm). Peak multiplicities are reported as follows: s = singlet, bs = broad singlet, d = doublet, t = triplet, dd = doublet of doublets, td = triplet of doublets, m = multiplet, app = apparent. The GC chromatograms were recorded using Varian© 3800 apparatus with Varian© CP-Sil 8CB® column. High-resolution mass spectra (HRMS) were obtained by the mass spectrometry facility at the Technion-Israel Institute of Technology. Reactions were monitored by gas chromatography spectrometry (GC) using an Agilent Technologies 7820A GC with an Agilent Technologies 19091J-413 (30 m × 0.3 mm) column or (GC-MS) Thermo Scientific TM Ion Trap GC/MS: ITQTM 900 with a Varian Factor Four Capillary column (VF-5 ms, 30m × 0.25mm). Crystal XRD data were collected on a diffractometer *Nonius Kappa CCD* at Schulich Faculty of Chemistry at Technion-Israel Institute of Technology.

Synthesis of starting materials ***1a-1s***

(i) *Synthesis of the cyclopropyl esters* ***S1a-S1m***: The cyclopropene esters were prepared according to known procedure[1] using commercially available alkynes, rhodium acetate dimer and alkyl diazoacetate in DCM. The cyclopropyl esters **S1a–S1m** were prepared according to previously described sequence of carbometalation/allylation[2] of cylopropene esters as shown in following scheme:

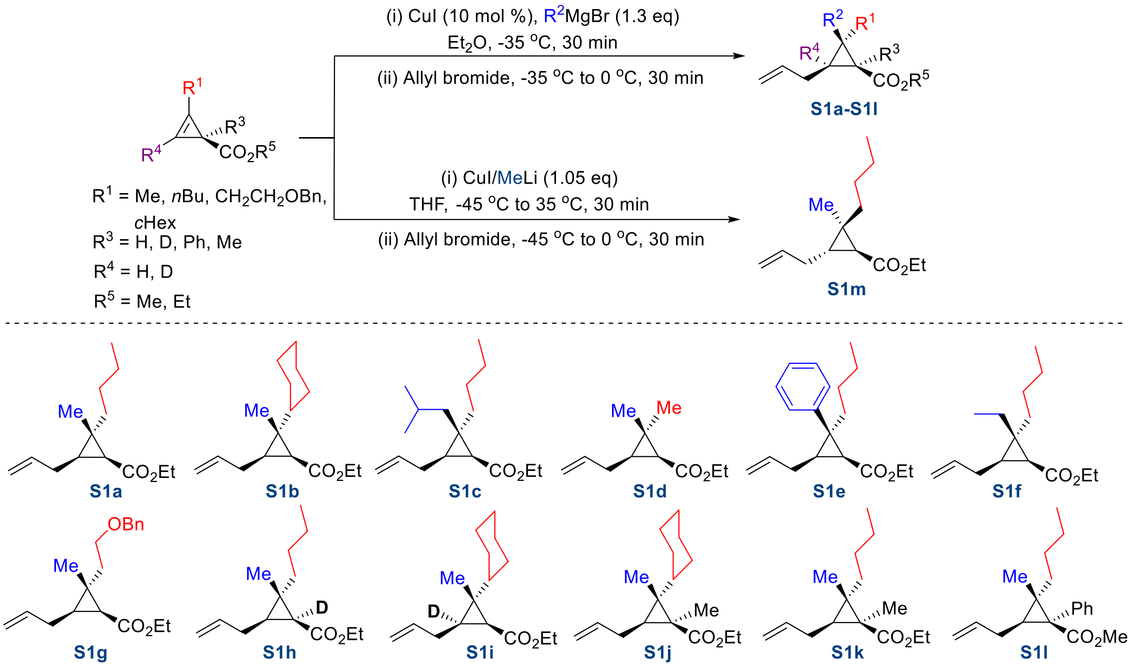

*General procedure for reduction of cyclopropyl esters (Procedure **A**)*

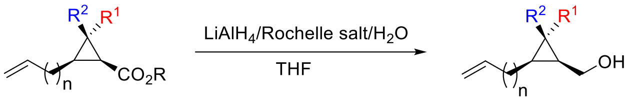

To a suspension of lithium aluminium hydride ($LiAlH_4$) (42 mg, 1.1 mmol) in THF (5 mL) in a flame-dried round-botton flask, the ester (1 mmol) dissolved in THF (5 mL) was added dropwise at 0°C over 15 min time-period. When the addition was over, the reaction mixture was stirred for overnight at room temperature. After completion (as monitored by TLC), ethyl acetate (2 mL) was added at 0 °C and the reaction mixture was stirred for 30 min and then a saturated aqueous solution of Rochelle salt (5 mL) was added dropwise and the reaction mixture was again stirred for 30 min. The reaction mixture was then extracted with Et_2O (3 x 10 mL), dried over $MgSO_4$, filtered and concentrated under vacuum to give the crude alcohol product which was further purified by column chromatography using 10-20% diethyl ether in hexane as eluent.

(ii) *Synthesis of the compounds **1a-1g***: The alcohols **1a-1g** were synthesized through the reduction of the cyclopropyl esters **S1a-S1g** using the general *Procedure **A*** (see scheme below):

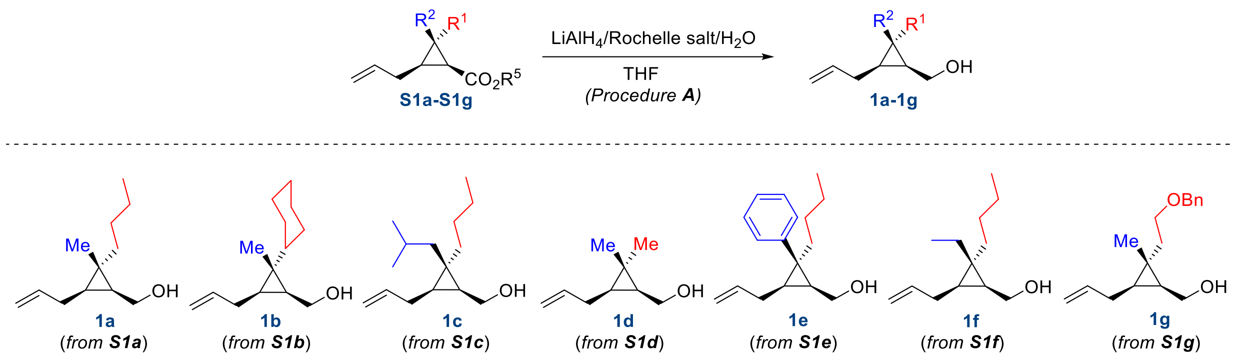

((1*S,2*S**,3*R**)-3-allyl-2-butyl-2-methylcyclopropyl)methanol**: ***1a***

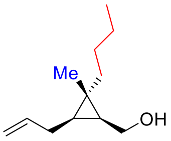

1a

The compound **1a** was obtained as a colorless oil. Yield: 79%. *dr* > 98:02:0:0. R_f = 0.2 (hexane/EtOAc, 90:10, v/v). ^{1}H NMR (400 MHz, $CDCl_3$) δ 0.71 (q, *J* = 8.3 Hz, 1H), 0.79-0.90 (m, 4H), 0.96 (s, 3H), 1.09-1.40 (m, 7H), 1.96-2.19 (m, 2H), 3.53-3.68 (m, 2H), 4.91-5.15 (m, 2H), 5.79-5.98 (m, 1H). ^{13}C NMR (100 MHz, $CDCl_3$) δ 12.42, 14.38, 22.51, 23.08, 25.81, 28.06, 28.66, 29.02, 43.02, 60.29, 114.58, 139.07. HRMS (ESI): Mass calcd for $C_{12}H_{22}O$ $[M]^+$: 182.1671; found: 182.1714.

((1*S,2*S**,3*R**)-3-allyl-2-cyclohexyl-2-methylcyclopropyl)methanol**: ***1b***

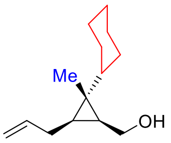

1b

The compound **1b** was obtained as a colorless oil. Yield: 73%. *dr* > 98:02:0:0. R_f = 0.2 (hexane/EtOAc, 90:10, v/v). ^{1}H NMR (400 MHz, $CDCl_3$) δ 0.30-0.42 (m, 1H), 0.65-0.73 (m, 1H), 0.78-0.89 (m, 5H), 0.99-1.33 (m, 6H), 1.59-1.64 (m, 2H), 1.68-1.33 (m, 2H), 1.98-2.18 (m, 2H), 3.54-3.68 (m, 2H), 4.90-5.14 (m, 2H), 5.78-5.95 (m, 1H). ^{13}C NMR (100 MHz, $CDCl_3$) δ 8.67, 26.11, 26.84, 27.07, 28.29, 28.78, 29.53, 29.80, 50.72, 60.21, 114.65, 139.12. HRMS (ESI): Mass calcd for $C_{14}H_{23}$ $[M-OH]^+$: 191.1800; found: 191.1745.

((1*S,2*R**,3*R**)-3-allyl-2-butyl-2-isobutylcyclopropyl)methanol**: ***1c***

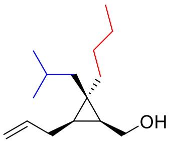

1c

The compound **1c** was obtained as a colorless oil. Yield: 69%. *dr* > 98:02:0:0. R_f = 0.3 (hexane/EtOAc, 90:10, v/v). ^{1}H NMR (400 MHz, $CDCl_3$) δ 0.64-0.75 (m, 1H), 0.77-0.92 (m, 9H), 0.98-1.44 (m, 10H), 1.66-1.78 (m, 1H), 1.89-2.01 (m, 1H), 2.19-2.32 (m, 1H), 3.43-3.54 (m, 1H), 3.65-3.75 (m, 1H), 4.92-5.17 (m 2H), 5.85-6.02 (m, 1H). ^{13}C NMR (100 MHz, $CDCl_3$) δ 14.33, 22.97, 23.21, 23.24, 24.95, 25.28, 25.85, 28.57, 28.63, 28.91, 34.41, 39.41, 60.21, 114.65, 139.57. HRMS (ESI): Mass calcd for $C_{15}H_{27}$ $[M-OH]^+$: 207.2113; found: 207.2106.

((1*S,3*R**)-3-allyl-2,2-dimethylcyclopropyl)methanol**: ***1d***

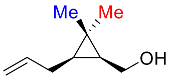

1d

The compound **1d** was obtained as a colorless oil. Yield: 88%. *dr* > 98:02. R_f = 0.2 (hexane/EtOAc, 90:10, v/v). ^{1}H NMR (300 MHz, $CDCl_3$) δ 0.65-0.93 (m, 2H), 0.99 (s, 3H), 1.07 (s, 3H), 1.33 (bs, 1H), 1.93-2.21 (m, 2H), 3.50-3.73 (m, 2H), 4.87-5.14 (m, 2H), 5.77-6.01 (m, 1H). ^{13}C NMR (100 MHz, $CDCl_3$) δ 14.98, 18.32, 26.14, 28.59, 29.29, 60.29, 114.49, 139.04. HRMS (ESI): Mass calcd for C_9H_{15} $[M-OH]^+$: 123.1174; found: 123.1169.

((1*S,2*R**,3*R**)-3-allyl-2-butyl-2-phenylcyclopropyl)methanol**: ***1e***

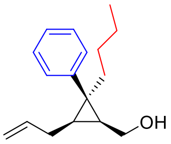

1e

The compound **1e** was obtained as a colorless oil. Yield: 86%. *dr* = 90:10:0:0. R_f = 0.3 (hexane/EtOAc, 90:10, v/v). ^{1}H NMR (400 MHz, $CDCl_3$) δ 0.78 (t, J = 7.0 Hz, 3H), 1.10-1.23 (m, 5H), 1.24-1.51 (m, 4H), 1.60-1.73 (m, 1H), 2.29-2.45 (m, 1H), 3.18-3.33 (m, 1H), 3.68-3.84 (m, 1H), 4.89-5.20 (m, 2H), 5.87-6.08 (m, 1H), 7.03-7.37 (m, 5H). ^{13}C NMR (100 MHz, $CDCl_3$) δ 14.29, 22.91, 25.99, 29.09, 30.74, 34.64, 44.69, 61.45, 114.97, 126.44, 128.33, 131.21, 139.56 (2xC). HRMS (ESI): Mass calcd for $C_{17}H_{23}$ $[M-OH]^+$: 227.1800; found: 227.1684.

((1*S,2*S**,3*R**)-3-allyl-2-butyl-2-ethylcyclopropyl)methanol**: ***1f***

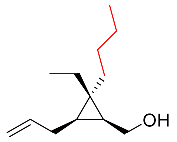

1f

The compound **1f** was obtained as a colorless oil. Yield: 80%. *dr* = 94:06:0:0. R_f = 0.2 (hexane/EtOAc, 90:10, v/v). ^1H NMR (400 MHz, $CDCl_3$) δ 0.58-0.95 (m, 8H), 1.07-1.45 (m, 8H), 1.75 (bs, 1H), 1.91-2.27 (m, 2H), 3.41-3.72 (m, 2H), 4.83-5.13 (m, 2H), 5.67-5.99 (m, 1H). ^{13}C NMR (100 MHz, $CDCl_3$) δ 10.99, 14.23, 18.60, 23.05, 26.09, 27.25, 28.33, 28.44, 38.35, 59.59, 114.43, 139.20. HRMS (ESI): Mass calcd for $C_{13}H_{23}$ $[M-OH]^+$: 179.1800; found: 179.1729.

((1*S,2*S**,3*R**)-3-allyl-2-(2-(benzyloxy)ethyl)-2-methylcyclopropyl)methanol**: ***1g***

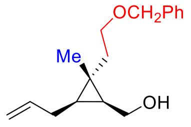

1g

The compound **1g** was obtained as a colorless oil. Yield: 93%. *dr* > 98:02:0:0. R_f = 0.3 (hexane/EtOAc, 90:10, v/v). ^1H NMR (400 MHz, $CDCl_3$) δ 0.62-0.73 (m, 1H), 0.81-0.90 (m, 1H), 0.94 (s, 3H), 1.38-1.63 (m, 2H), 1.91-2.05 (m, 2H), 2.16 (bs, 1H), 3.34-3.62 (m, 4H), 4.40 (s, 2H), 4.80-5.07 (m, 2H), 5.67-5.90 (m, 1H), 7.08-7.36 (m, 5H). ^{13}C NMR (100 MHz, $CDCl_3$) δ 12.24, 19.65, 25.22, 27.65, 28.33, 42.20, 59.46, 68.57, 73.02, 114.38, 127.52, 127.62, 128.32, 138.33, 138.45. HRMS (ESI): Mass calcd for $C_{17}H_{24}O_2$ $[M]^+$: 260.1819; found: 260.1828.

*General procedure for oxidation of cyclopropyl alcohols (Procedure **B**)*

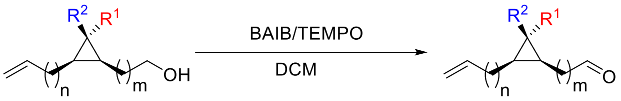

To a solution of cyclopropyl alcohol (2 mmol) and bis(acetoxy)iodobenzene (BAIB) (773 mg, 2.4 mmol) in DCM (6 mL) in a round-bottom flask, (2,2,6,6-tetramethyl-1-piperidin-1-yl)oxy (TEMPO) (32 mg, 0.2 mmol) was added at rt and the resulting reaction mixture was stirred for 2 h at that temperature. After completion (as monitored by TLC), the reaction mixture was diluted with DCM (20 mL) and washed with a saturated aqueous solution of $Na_2S_2O_3$ (20 mL). The aqueous layer was extracted with DCM (2 x 20 mL) and the combined organic layers were dried over $MgSO_4$, filtered and evaporated under vacuum to give the crude aldehyde product which was further purified by column chromatography using 5-10% Et_2O in hexane as eluent.

*General procedure for Grignard reaction on cyclopropyl aldehydes (Procedure **C**)*

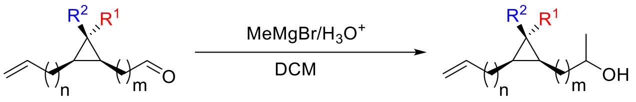

To a solution of cyclopropyl aldehyde (1 mmol) in dry Et_2O (10 mL), MeMgBr 3M (0.75 mL, 2.25 mmol) was added dropwise at 0 °C and then reaction mixture was stirred overnight at room temperature. After completion (as monitored by TLC), the reaction mixture was quenched with saturated aqueous solution of NH_4Cl (10 mL) and extracted with Et_2O (3 x 10 mL). The combined organic layers were dried over $MgSO_4$, filtered and evaporated under vacuum to give the crude alcohol product which was further purified by flash chromatography using 20-30% Et_2O in hexane as eluent.

(iii) *Synthesis of the compound **1h***: The alcohol **1h** was synthesized from the alcohol **1a** as shown in the following scheme:

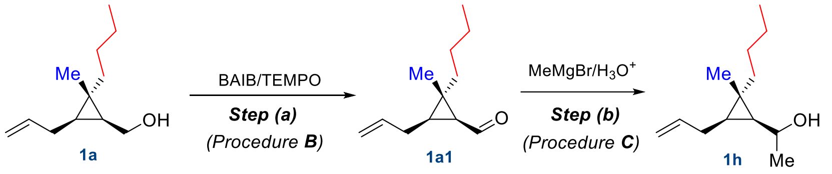

Step (a): The aldehyde **1a1** was synthesized through oxidation of the alcohol **1a** using the general *Procedure **B***. The compound **1a1** was obtained as a colorless oil in 82% yield. *dr* > 98:02:0:0. R_f = 0.6 (hexane/EtOAc, 90:10, v/v). ^{1}H NMR (400 MHz, $CDCl_3$) δ 0.86 (t, *J* = 6.9 Hz, 3H), 1.74-1.48 (m, 10H), 1.56-1.65 (m, 1H), 2.32-2.57 (m, 2H), 4.92-5.10 (m, 2H), 5.71-5.88 (m, 1H), 9.49 (d, *J* = 5.8 hz, 1H). ^{13}C NMR (100 MHz, $CDCl_3$) δ 12.63, 14.27, 22.88, 28.38, 28.64, 34.18, 35.89, 37.98, 42.83, 115.51, 137.10, 202.02.

Step (b): The alcohol **1h** was synthesized from the aldehyde **1a1** by using the general *Procedure **C***.

1-((1*S,2*S**,3*R**)-3-allyl-2-butyl-2-methylcyclopropyl)ethan-1-ol**: ***1h***

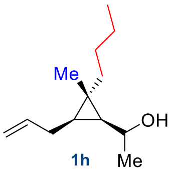

The compound **1h** was obtained as a colorless oil and as a unique diastereomer except secondary alcohol with *dr*: 6:4. Yield: 92%. R_f = 0.3 (hexane/EtOAc, 90:10, v/v). ^{1}H NMR (400 MHz, $CDCl_3$) (*mixture of diastereomers*) δ 0.53-0.74 (m, 2H), 0.81-1.16 (m, 7H), 1.17-1.38 (m, 8H), 1.45 & 1.60 (2 x s, 1H), 1.90-2.30 (m, 2H), 3.47-3.63 (m, 1H), 4.91-5.19 (m, 2H), 5.73-6.06 (2 x m, 1H). ^{13}C NMR

(100 MHz, $CDCl_3$) (*mixture of diastereomers*) δ 12.55 & 12.71, 14.36, 22.25 & 22.61, 23.14, 23.18 & 24.05, 25.63 & 26.13, 28.79 & 28.84, 28.97 & 29.18, 34.06 & 34.39, 42.99 & 43.26, 65.78 & 66.32, 114.65 & 114.70, 138.49 & 139.65. HRMS (ESI): Mass calcd for $C_{13}H_{23}$ $[M-OH]^+$: 179.1800; found: 179.1801.

(iv) *Synthesis of the compound **1i***: The compound **1i** was synthesized from the alcohol **1c** as shown in the following scheme:

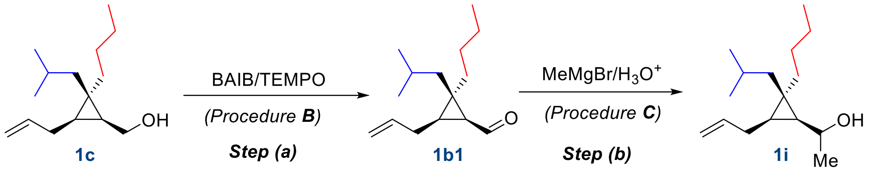

Step (a): The aldehyde **1b1** was synthesized by the oxidation of the alcohol **1c** using the general *Procedure* ***B*** for the oxidation of cyclopropyl alcohols as described above. The compound **1b1** was obtained as a colorless oil in 95% yield. *dr* > 98:02:0:0. R_f = 0.5 (hexane/EtOAc, 90:10, v/v). ^{1}H NMR (400 MHz, $CDCl_3$) δ 0.79-0.95 (m, 9H), 1.00-1.11 (m, 1H), 1.15-1.87 (m, 10H), 2.33-2.60 (m, 2H), 4.87-5.20 (m, 2H), 5.68-5.89 (m, 1H), 9.46 (d, J = 6.1 Hz, 1H). ^{13}C NMR (100 MHz, $CDCl_3$) δ 14.27, 22.34, 23.02, 23.42, 25.36, 28.42, 28.67, 33.41, 35.35, 36.52, 38.33, 39.21, 115.71, 137.12, 202.01.

Step (b): The alcohol **1i** was synthesized from the aldehyde **1b1** by using the general *Procedure* ***C***.

1-((1*S,2*R**,3*R**)-3-allyl-2-butyl-2-isobutylcyclopropyl)ethan-1-ol**: ***1i***

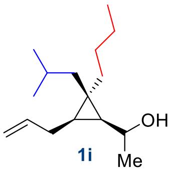

The compound **1i** was obtained as a colorless oil and as a unique diastereomer except secondary alcohol with *dr*: 5:4. Yield: 88%. R_f = 0.3 (hexane/EtOAc, 90:10, v/v). ^{1}H NMR (400 MHz, $CDCl_3$) (*mixture of diastereomers*) δ 0.38-0.82 (m, 3H), 0.82-0.98 (m, 9H), 1.04-1.44 (m, 8H), 1.47-1.84 (m, 4H), 1.88-2.02 (m, 1H), 2.14-5.51 (2 x m, 1H), 3.48-3.66 (m, 1H), 4.90-5.25 (m, 2H), 5.76-6.13 (2 x m, 1H). ^{13}C NMR (100 MHz, $CDCl_3$) (*mixture of diastereomers*) δ 14.33, 22.12, 23.02 & 23.21, 23.29 & 23.32, 24.40 & 24.58, 25.11 & 25.25, 25.50 & 25.65, 26.96 & 27.15, 28.47 & 28.92, 29.60 & 29.85, 34.00, 34.44 & 34.56, 39.44 & 39.61, 65.13 & 65.88, 114.84 & 114.90, 138.61 & 140.33. HRMS (ESI): Mass calcd for $C_{16}H_{29}$ $[M-OH]^+$: 221.2269; found: 221.2276.

*Typical procedure for triisopropylsilylation of cyclopropyl alcohol (Procedure **D**)*

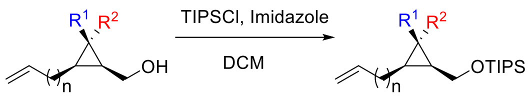

To a solution of cyclopropyl alcohol (3 mmol) and imidazole (510 mg, 7.5 mmol) in DCM (15 mL), TIPSCl (0.8 mL, 3.75 mmol) was added dropwise at 0 °C under an inert atmosphere. After the addition was over, the resulting reaction mixture was allowed to warm-up to room temperature and stirred overnight until completion of the reaction was observed by TLC). Then, the reaction mixture was diluted with an equal volume of DCM and sucesscively washed with water (20 mL) and brine (20 mL). The organic layer was dried over $MgSO_4$, filiteredand concentrated under vacuum. The resulting crude silyl ether product was purified by flash chromatography using pure pentane as eluent.

__Typical procedure for hydroboration/oxidation of cyclopropyl alkenes (Procedure E)__

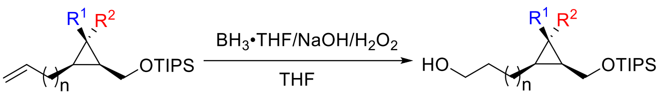

To a solution of cyclopropyl alkene (3 mmol) in THF (15 mL), BH_3•THF 1M solution in THF (3.3 mL,3.3 mmol) was added dropwise at 0 °C under an inert atmosphere and after the addition was over, the resulting solution was stirred at room temperature for 2 h. When the starting alkene was consumed, excess of BH_3•THF was quenched with water (6 mL) and then 1M NaOH solution (3 mL) was added slowly at room temperature followed by the addition of H_2O_2 (1.5 mL, 30% solution in water). The reaction mixture was further stirred overnight. The reaction mixture was lastly extracted with Et_2O (3 x 20 mL) and the combined oganic layers were dried over $MgSO_4$, filtered and concentrated under vacuum to give the crude alcohol product which was further purified by flash column chromatography (using 20-30% Et_2O in hexane as eluent).

__General procedure used for the conversion of silyl ether into alcohol (Procedure F)__

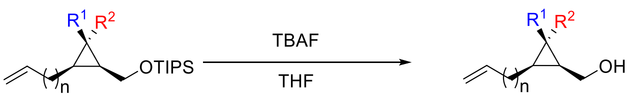

To a solution of silyl ether (1 mmol) in dry THF (10 mL), tetrabutylammonium fluoride 1M solution in THF (TBAF) (1.5 mL, 1.5 mmol,) was added dropwise at 0 °C under argon and the resulting reaction mixture was further stirred overnight. After completion (as indicated by TLC), the resulting mixture was diluted with DCM (10 mL) and the reaction was quenched with water (5 mL). The extracted organic layer was washed with brine (5 mL) and then dried over $MgSO_4$, filtered and concentrated

under vacuum. The crude alcohol product obtained was purified by column chromatography using 30% diethyl ether in hexane as eluent.

(v) *Synthesis of the compound* ***1j***: The alcohol **1j** was synthesized from the alcohol **1a** as shown in the following scheme:

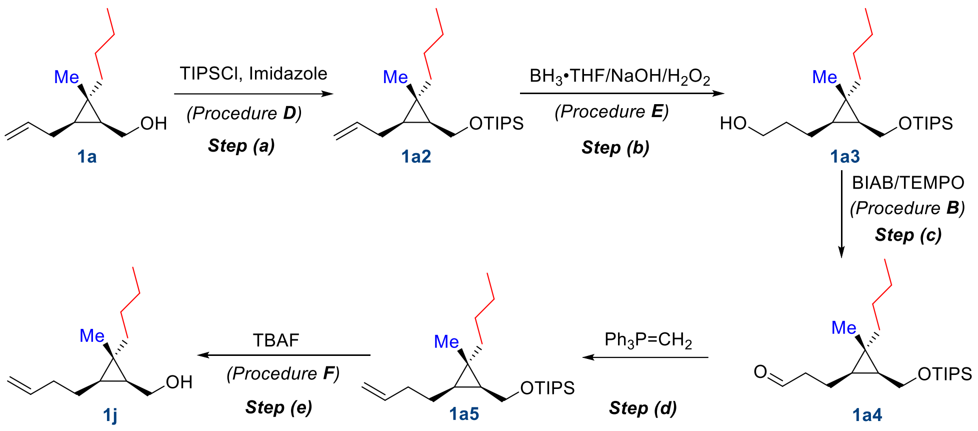

Step (a): The silyl ether **1a2** was synthesized from the alcohol **1a** by using the general *Procedure* ***D*** and then used for the next without any purification.

Step (b): The alcohol **1a3** was synthesized from the compound **1a2** by using the general *Procedure* ***E*** and then used for the next without any purification.

Step (c): The aldehyde **1a4** was synthesized from the alcohol **1a3** by using the general *Procedure* ***B*** and was obtained as a colorless oil in 73% overall yield starting from the alcohol **1a**. R_f = 0.5 (hexane/EtOAc, 90:10, v/v). ^{1}H NMR (400 MHz, $CDCl_3$) δ 0.43-0.61 (m, 1H), 0.74-0.88 (m, 4H), 0.93 (s, 3H), 0.98-1.37 (m, 27H), 1.63 (q, *J* = 7.4 Hz, 2H), 2.37-2.65 (m, 2H), 3.45-3.85 (m, 2H), 9.77 (t, *J* = 1.7 Hz, 1H). ^{13}C NMR (100 MHz, $CDCl_3$) δ 12.23, 12.31, 14.40, 17.56, 18.29, 22.21, 23.04, 29.05, 43.05, 44.79, 60.36, 203.10.

Step (d): To a solution of methyltriphenylphosphonium bromide (1.4 g, 4 mmol) in dry THF (20 mL) in a three-neck round-bottom flask at -30 °C, *n*BuLi 2.35 M in hexanes (1.7 mL,4.0 mmol) and the reaction mixture was allowed to stir while increasing the temperature from -30 °C to 0 °C. After 30 minutes, the reaction mixture was cool down to -30 °C and the aldehyde **1a4** (708 mg, 2 mmol), dissolved in THF (10 mL) was added dropwise. The reaction mixture was then stirred for 1 h from -30 °C to room temperature. After completion (as indicated by TLC), the reaction mixture was quenched with a saturated aqueous solution of NH_4Cl (20 mL) and extracted with Et_2O (3 x 20 mL). The combined organic layers were dried over $MgSO_4$, filtered and evaporated to give the crude alkene product **1a5** which was purified by column chromatography in pure pentane. The compound **1a5** was obtained in 80% yield and used for the next step without any characterisation.

Step (e): The cyclopropyl alcohol **1j** was synthesized from the silyl ether **1a5** by using the general *Procedure **F***.

((1*S,2*S**,3*R**)-3-(but-3-en-1-yl)-2-butyl-2-methylcyclopropyl)methanol**: ***1j***

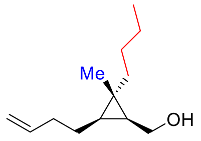

1j

The compound **1j** was obtained as a colorless oil. Overall yield from **1a4**: 73%. R_f = 0.3 (hexane/EtOAc, 90:10, v/v). ^{1}H NMR (400 MHz, $CDCl_3$) δ 0.51-0.66 (m, 1H), 0.69-1.05 (m, 8H), 1.09-1.51 (m, 8H), 1.98-2.14 (m, 2H), 3.49-3.70 (m, 2H), 4.81-5.12 (m, 2H), 5.68-5.94 (m, 1H). ^{13}C NMR (100 MHz, $CDCl_3$) δ 12.29, 14.38, 22.38, 23.10, 24.09, 26.60, 28.33, 29.09, 34.64, 43.08, 60.29, 114.78, 138.96. HRMS (ESI): Mass calcd for $C_{13}H_{25}O$ $[M+H]^+$: 197.1905; found: 197.1944.

*General procedure for mesylation of cyclopropyl alcohols (Procedure **G**)*

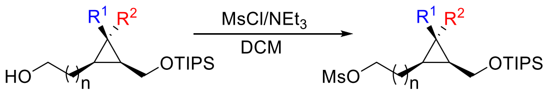

To a solution of alcohol (2 mmol) and 4-dimethylaminopyridine (DMAP) (122 mg, 1.0 mmol) in dry DCM (10 mL) in a flame dried round-bottom flask, triethylamine (0.56 mL, 4 mmol) was added dropwise at 0 °C and the resulting reaction mixture was stirred at room temperature for 20 min. Then, MsCl (0.4 mL, 5 mmol) was added dropwise at 0 °C and the reaction mixture was stirred for 2 h. After completion (as indicated by TLC), the reaction mixture was quenched with an aqueous solution of HCl 1M (10 mL) and extracted with DCM (3 x 20 mL), dried over $MgSO_4$, filtered and evaporated under vacuum to give the crude mesylate product which was further purified by column using 20% Et_2O in hexane as eluent.

*General procedure for alkenylation of cyclopropyl mesylate using Grignard reagent (Procedure **H**)*

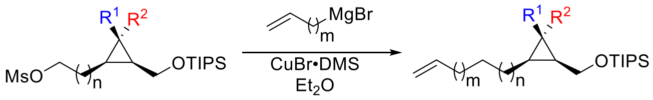

To a solution of mesylate product (1.5 mmol) and CuBr•DMS (62 mg, 0.3 mmol) in dry Et_2O (8 mL) in a flame dried round-bottom flask, alkenylmagnesium bromide (solution in Et_2O) (3 mmol) was added dropwise at 0 °C and the resulting reaction mixture was stirred at room temperature for 2 h. After completion (as monitored by TLC), the reaction mixture was quenched with a saturated aqueous

solution of NH_4Cl (15 mL) and extracted with Et_2O (3 x 15 mL), dried over $MgSO_4$ and concentrated under vacuum to give the crude silyl ether product which was passed through a pad of silica using pentane as eluent before using it for the next step.

(vi) *Synthesis of the compound **1k***: The alcohol **1k** was synthesized from alcohol **1a3** according to the following scheme:

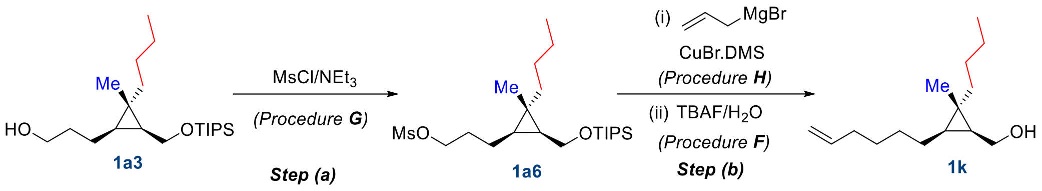

Step (a): The mesylate **1a6** was synthesized from the alcohol **1a3** by using the general *Procedure **F*** and was obtained as a colorless oil in 80% yield. R_f = 0.5 (hexane/EtOAc, 80/20, v/v). ^{1}H NMR (400 MHz, $CDCl_3$) δ 0.46-0.56 (m, 1H), 0.73-0.89 (m, 4H), 0.92 (s, 3H), 0.97-1.09 (m, 18H), 1.10-1.46 (m, 9H), 1.71-1.91 (m, 2H), 2.97 (s, 3H), 3.58-3.71 (m, 2H), 4.17-4.28 (m, 2H). ^{13}C NMR (100 MHz, $CDCl_3$) δ 12.36, 12.50, 14.41, 17.91, 20.70, 22.07, 23.06, 26.00, 28.35, 29.09, 29.92, 37.59, 43.13, 60.44, 70.28.

Step (b): The alcohol **1k** was synthesized from the mesylate **1a6** by using the general *Procedure **H*** followed by *Procedure **F***.

((1*S,2*S**,3*R**)-2-butyl-3-(hex-5-en-1-yl)-2-methylcyclopropyl)methanol**: ***1k***

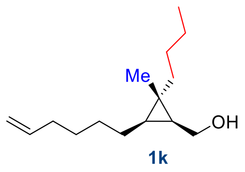

The compound **1k** was obtained as a colorless oil. Overall yield from **1a6**: 54%. *dr* > 98:02:0:0. R_f = 0.3 (hexane/EtOAc, 90:10, v/v). ^{1}H NMR (400 MHz, $CDCl_3$) δ 0.50-0.65 (m, 1H), 0.71-0.91 (m, 5H), 0.95 (s, 3H), 1.00-1.43 (m, 12H), 1.93-2.14 (m, 2H), 3.48-3.74 (m, 2H), 4.79-5.13 (m, 2H), 5.67-5.90 (m, 1H). ^{13}C NMR (100 MHz, $CDCl_3$) δ 12.34, 14.39, 22.44, 23.13, 24.39, 27.16, 28.36, 29.07, 29.14, 30.00, 33.95, 43.17, 60.52, 114.46, 139.23. HRMS (ESI): Mass calcd for $C_{15}H_{29}O$ $[M+H]^+$: 225.2218; found: 225.2210.

(vii) *Synthesis of the compound **1l***: The alcohol **1l** was synthesized from the mesylate **1a6** by using the general *Procedure **H*** followed by *Procedure **F*** according to following scheme:

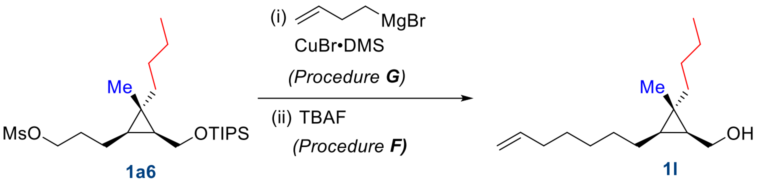

((1*S,2*S**,3*R**)-2-butyl-3-(hept-6-en-1-yl)-2-methylcyclopropyl)methanol**: ***1l***

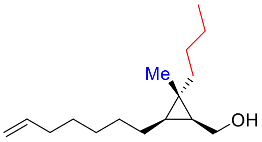

1l

The compound **1l** was obtained as a colorless oil. Overall yield from **1a6**: 58%. *dr* = 92:08:0:0. R_f = 0.3 (hexane/EtOAc, 90:10, v/v). ^{1}H NMR (400 MHz, $CDCl_3$) δ 0.41-0.56 (m, 1H), 0.63-1.00 (m, 8H), 1.02-1.40 (m, 13H), 1.87-2.07 (m, 2H), 2.20 (bs, 1H), 3.39-3.64 (m, 2H), 4.71-5.07 (m, 2H), 5.61-5.87 (m, 1H). ^{13}C NMR (100 MHz, $CDCl_3$) δ 12.10, 14.22, 17.80, 22.13, 23.00, 24.33, 26.90, 28.08, 28.97, 29.20, 30.24, 33.87, 43.09, 59.91, 114.24, 139.05. HRMS (ESI): Mass calcd for $C_{16}H_{30}O$ $[M]^+$: 238.2297; found: 238.2291.

*General procedure for homologation of cyclopropyl aldehydes through Wittig reaction/Hydrolysis (Procedure **I**)*

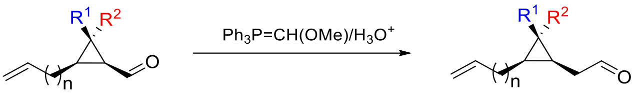

To a solution of phosphonium salt $Ph_3PCH(OMe)Cl$ (820 mg, 2.4 mmol) in THF (20 mL) in a 100 mL three-neck round-bottom flask, *n*BuLi (2.4 mL, 1M solution in hexanes) was added dropwise under argon at -30 °C. The reaction mixture was turned blood red indicating the formation of methoxymethylenetriphenylphosphine (Ph_3P=CHOMe) and was further stirred for 30 min. After it had slowly been warmed up to 0 °C, a solution of aldehyde (2 mmol) in THF (4 mL) was added dropwise and the reaction mixture was left on stirring for 2 h at 0 °C. The reaction mixture was finally diluted with Et_2O (20 mL) and quenched with a saturated aqueous solution of NH_4Cl (20 mL). The two phases were separated and the aqueous phase was extracted with Et_2O (2 x 20 mL). The combined organic layers were dried over $MgSO_4$, filetered and concentrated under vacuum to give the crude methoxymethylenetriphenylphosphine compound from which the hydrolysis step was performed. In a 100 mL one-neck flask, the crude obtained methoxymethylenetriphenylphosphine product was set in a mixture of HCl 1M (5 mL) and acetonitrile (5 mL) and the biphasic solution was stirred for 2.5 h at room temperature. After completion (as indicated by TLC), the reaction mixture was quenched with

saturated aqueous solution of $NaHCO_3$ (15 mL) and the aqueous phase was extracted with Et_2O (3 x 10 mL). The organic phases were combined, dried over $MgSO_4$, filtered and concentrated under vacuum. A column chromatography (2-5% Et_2O in hexane) afforded the desired homologated aldehyde product.

(viii) *Synthesis of the compound **1m**:* The alcohol **1m** was synthesized from the aldehyde **1a1** according to the following scheme:

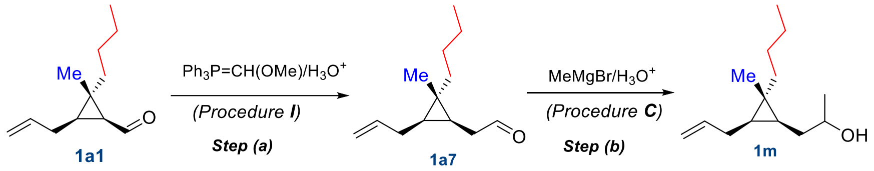

Step (a): The aldehyde **1a7** was synthesized from the aldehyde **1a1** by using the general *Procedure **I***. The aldehyde **1a7** was obtained as a colourless liquid in 62% yield. *dr* > 98:02:0:0. R_f = 0.6 (hexane/EtOAc, 90:10, v/v). ^{1}H NMR (400 MHz, $CDCl_3$) δ 0.59-0.69 (m, 1H), 0.73-0.94 (m, 7H), 1.08-1.40 (m, 6H), 1.82-2.04 (m, 2H), 2.14-2.44 (m, 2H), 4.84-5.05 (m, 2H), 5.66-5.88 (m, 1H), 9.72 (s, 1H). ^{13}C NMR (100 MHz, $CDCl_3$) δ 12.59, 14.34, 19.07, 21.42, 23.13, 24.80, 28.81, 28.90, 39.62, 42.91, 114.76, 138.03, 202.71.

Step (b): The alcohol **1m** was synthesized from the aldehyde **1a7** by using the general *Procedure **C***.

1-((1*S,2*S**,3*R**)-3-allyl-2-butyl-2-methylcyclopropyl)propan-2-ol**: ***1m***

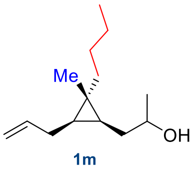

The compound **1m** was obtained as a colorless oil and as a unique diastereomer except secondary alcohol with *dr*: 1:1. Yield: 70%. R_f = 0.3 (hexane/EtOAc, 90:10, v/v). ^{1}H NMR (400 MHz, $CDCl_3$) (*mixture of diastereomers*) δ 0.44-0.61 (m, 2H), 0.78-0.93 (m, 6H), 1.05-1.45 (m, 12H), 1.87-2.05 (m, 2H), 3.71-3.87 (m, 1H), 4.86-5.12 (m, 2H), 5.76-5.94 (m, 1H). ^{13}C NMR (100 MHz, $CDCl_3$) (*mixture of diastereomers*) δ 12.56 & 12.63, 14.36, 21.02 & 21.24, 22.35 & 22.49, 23.18, 23.32 & 23.49, 24.82, 28.96 & 29.03, 34.12, 43.33, 68.80 & 68.98, 114.30 & 114.34, 138.83 & 138.86. HRMS (ESI): Mass calcd for $C_{14}H_{27}O$ $[M+H]^+$: 211.2062; found: 211.2068.

(ix) *Synthesis of the compound **1n**:* The alcohol **1n** was synthesized from the aldehyde **1a7** according to the following scheme:

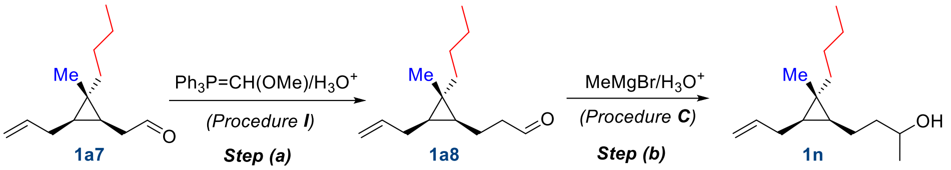

Step (a): The aldehyde **1a8** was synthesized from the aldehyde **1a7** by using the general *Procedure **I*** and was obtained as a colourless liquid in 50% yield. *dr* > 98:02:0:0. R_f = 0.6 (hexane/EtOAc, 90:10, v/v). ^{1}H NMR (400 MHz, $CDCl_3$) δ 0.30-0.53 (m, 2H), 0.70-0.88 (m, 6H), 0.94-1.29 (m, 6H), 1.40-1.63 (m, 2H), 1.85-1.02 (m, 2H), 2.26-2.49 (m, 2H), 4.78-5.04 (m, 2H), 5.64-5.88 (m, 1H), 9.69 (t, *J* = 1.9 Hz, 1H). ^{13}C NMR (100 MHz, $CDCl_3$) δ 12.13, 14.37, 17.48, 21.46, 23.15, 25.15, 28.58, 29.07, 43.17, 44.69, 114.39, 138.76, 202.94. HRMS (ESI): Mass calcd for $C_{14}H_{25}O$ $[M+H]^+$: 209.1905; found: 209.1903.

Step (b): The alcohol **1n** was synthesized from the aldehyde **1a8** by using the general *Procedure **C***.

4-((1S*,2R*,3R*)-3-allyl-2-butyl-2-methylcyclopropyl)butan-2-ol: ***1n***

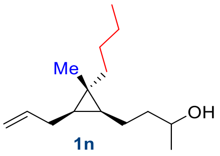

The compound **1n** was obtained as a colorless oil and as a unique diastereomer except secondary alcohol with *dr*: 1:1. Yield: 74%. R_f = 0.3 (hexane/EtOAc, 90:10, v/v). ^{1}H NMR (400 MHz, $CDCl_3$) (*mixture of diastereomers*) δ 0.34-0.56 (m, 2H), 0.80-0.93 (m, 6H), 1.02-1.56 (m, 14H), 1.88-2.09 (m, 2H), 3.69-3.87 (m, 1H), 4.86-5.09 (m, 2H), 5.74-5.94 (m, 1H). ^{13}C NMR (100 MHz, $CDCl_3$) (*mixture of diastereomers*) δ 12.21, 14.41, 20.77, 21.20 & 21.24, 23.21, 23.75 & 23.79, 25.08 & 25.13, 25.80 & 25.85, 28.77, 29.12, 40.10 & 43.38, 68.38, 114.16, 139.16 & 139.18. HRMS (ESI): Mass calcd for $C_{15}H_{29}O$ $[M+H]^+$: 225.2218; found: 225.2213.

(x) *Synthesis of the compound **1o**:* The alcohol **1o** was synthesized from the alcohol **1k** according to the following scheme:

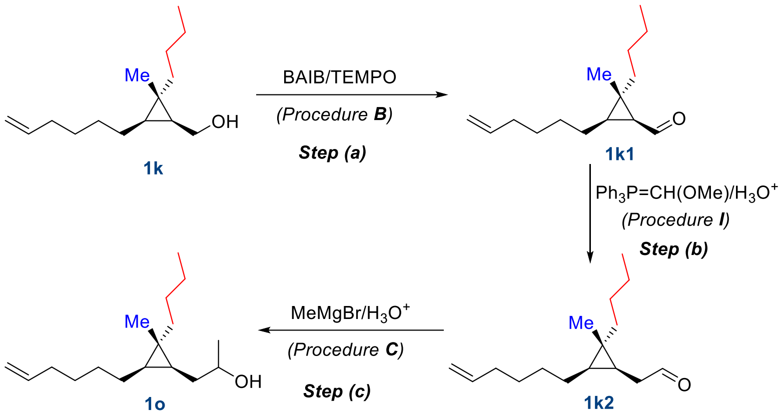

Step (a): The aldehyde **1k1** was synthesized from the alcohol **1k** using the general *Procedure* ***B*** and was obtained as colorless oil in 90% yield. The compound **1k1** was used for the next step without any characterisation.

Step (b): The aldehyde **1k2** was synthesized from the aldehyde **1k1** by using the general *Procedure* ***I***. The desired homologated aldehyde product **1k2** was obtained as a colourless liquid in 60% yield and used for the next step without any purification.

Step (c): The alcohol **1o** was synthesized from the aldehyde **1k2** by using the general *Procedure* ***C***.

1-((1*S,2*S**,3*R**)-2-butyl-3-(hex-5-en-1-yl)-2-methylcyclopropyl)propan-2-ol**: ***1o***

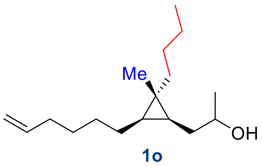

The compound **1o** was obtained as a colorless oil and as a unique diastereomer except secondary alcohol with *dr*: 1:1. Overall yield from **1k**: 51%. R_f = 0.3 (hexane/EtOAc, 90:10, v/v). ^{1}H NMR (400 MHz, $CDCl_3$) (*mixture of diastereomers*) δ 0.29-0.48 (m, 2H), 0.70-0.88 (m, 6H), 0.99-1.62 (m, 18H), 1.89-2.07 (m, 2H), 3.63-3.85 (m, 1H), 4.78-5.02 (m, 2H), 5.63-5.84 (m, 1H). ^{13}C NMR (100 MHz, $CDCl_3$) (*mixture of diastereomers*) δ 12.59 & 12.70, 14.40, 21.11 & 21.30, 22.62 & 22.67, 23.23, 23.35 & 23.47, 24.59 & 24.68, 25.99 & 26.03, 29.14 & 29.19, 29.83, 34.00 & 34.27, 43.52, 68.98 & 69.14, 114.39, 139.34. HRMS (ESI): Mass calcd for $C_{17}H_{33}O$ $[M+H]^+$: 253.2516; found: 253.2531.

(xi) *Synthesis of the compound* ***1p***: The compound **1p** was obtained by the reduction of the cyclopropyl ester **S1l** using the general *Procedure* ***A*** according to the following scheme:

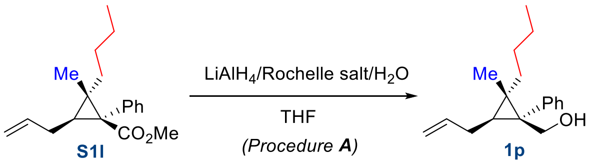

((1*S,2*S**,3*S**)-3-allyl-2-butyl-2-methyl-1-phenylcyclopropyl)methanol**: ***1p***

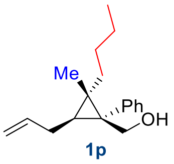

The compound **1p** was obtained as a colorless oil. Yield: 95%. *dr* > 98:02:0:0. R_f = 0.3 (hexane/EtOAc, 90:10, v/v). ^{1}H NMR (400 MHz, $CDCl_3$) δ 0.39-0.54 (m, 1H), 0.72 (t, *J* = 7.3 Hz, 3H), 0.94-1.39 (m, 10H), 2.15-2.39 (m, 2H), 3.71 (d, *J* = 11.7 Hz, 1H), 3.90 (d, *J* = 11.7 Hz, 1H), 4.94-5.20 (m, 2H), 5.85-6.08 (m, 1H), 7.09-7.35 (m, 5H). ^{13}C NMR (100 MHz, $CDCl_3$) δ 13.21, 14.27, 23.05, 27.81, 29.05, 29.74, 31.49, 39.25, 39.62, 64.74, 115.09, 126.58, 128.43, 130.56, 138.81, 142.53. HRMS (ESI): Mass calcd for $C_{17}H_{23}$ [M-CH_2OH]$^+$: 227.1800; found: 227.1810.

(xii) *Synthesis of the compound* ***1q****:* The compound **1q** was synthesized from the cyclopropyl ester **S1k** according to following scheme:

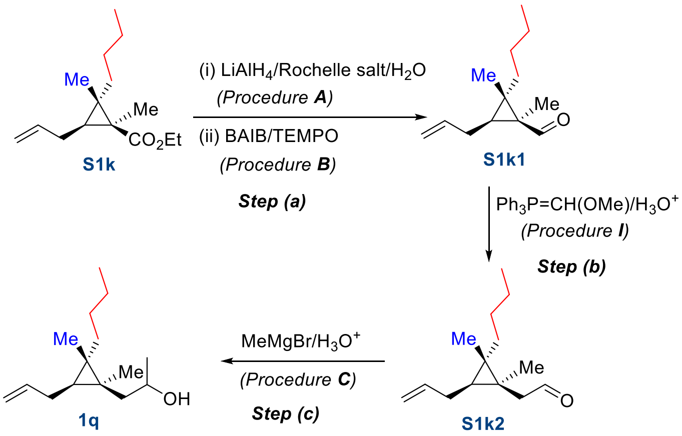

Step (a): The aldehyde **S1k1** was synthesized by the general *Procedure* ***A*** followed by the general *Procedure* ***B***. The aldehyde product **S1k1** was obtained in 96% yield as a colorless oil. *dr* > 98:02:0:0. R_f = 0.5 (hexane/EtOAc, 90:10, v/v). ^{1}H NMR (400 MHz, $CDCl_3$) δ 0.87 (t, *J* = 7.1 Hz, 3H), 1.11 (dd, *J* = 8.3, 7.1 Hz, 1H), 1.18-1.52 (m, 12H), 2.33-2.62 (m, 2H), 4.86-5.09 (m, 2H), 5.68-5.91 (m, 1H), 9.40 (s, 1H). ^{13}C NMR (100 MHz, $CDCl_3$) δ 13.93, 14.23, 14.65, 23.11, 28.73, 28.90, 36.10, 37.83, 38.31, 41.91, 115.46, 137.10, 204.45.

Step (b): The aldehyde **S1k2** was synthesized from the aldehyde **S1k1** by using the general *Procedure **I*** and was obtained as a colourless liquid in 61% yield. *dr* > 98:02:0:0. R_f = 0.6 (hexane/EtOAc, 90:10, v/v). ^{1}H NMR (400 MHz, $CDCl_3$) δ 0.35 (t, *J* = 7.5 Hz, 1H), 0.82 (t, *J* = 7.0 Hz, 3H), 0.90 (s, 3H), 1.10 (s, 3H), 1.14-1.42 (m, 6H), 1.87-2.03 (m, 2H), 2.14-2.34 (m, 2H), 4.81-5.02 (m, 2H), 5.64-5.87 (m, 1H), 9.68 (t, *J* = 2.3 Hz, 1H). ^{13}C NMR (100 MHz, $CDCl_3$) δ 14.33, 14.72, 21.79, 21.90, 23.36, 25.69, 29.43, 29.62, 32.64, 37.81, 46.35, 114.65, 138.15, 203.69.

Step (c): The alcohol **1q** was synthesized from the aldehyde **S1k2** by using the general *Procedure **C***.

1-((1*S,2*S**,3*S**)-3-allyl-2-butyl-1,2-dimethylcyclopropyl)propan-2-ol**: ***1q***

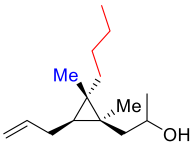

1q

The compound **1q** was obtained as a colorless oil and as a unique diastereomer except secondary alcohol with *dr*: 6:4. Yield: 73%. R_f = 0.3 (hexane/EtOAc, 90:10, v/v). ^{1}H NMR (400 MHz, $CDCl_3$) (*mixture of diastereomers*) δ 0.15-0.36 (m, 1H), 0.69-1.04 (m, 6H), 1.05-1.60 (m, 15H), 1.86-2.17 (m, 2H), 3.87-4.14 (m, 1H), 4.83-5.15 (m, 2H), 5.73-5.97 (m, 1H). ^{13}C NMR (100 MHz, $CDCl_3$) (*mixture of diastereomers*) δ 14.33 & 14.37, 14.72 & 15.16, 21.01 & 21.06, 22.90 & 23.38, 23.44, 23.69 & 23.87, 25.38 & 25.86, 29.24 & 29.49, 29.60, 33.15, 38.18 & 38.26, 40.31 & 40.40, 65.77 & 66.81, 114.19 & 114.25, 138.91 & 139.09. HRMS (ESI): Mass calcd for $C_{15}H_{29}O$ $[M+H]^+$: 225.2218; found: 225.2217.

(xiii) *Synthesis of compound **1r**:* The compound **1r** was synthesized from the cyclopropyl ester **S1j** according to the following scheme:

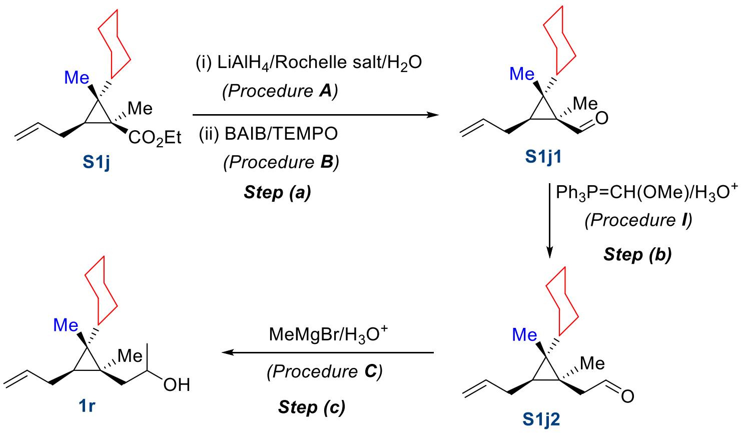

Step (a): The aldehyde **S1j1** was synthesized from cyclopropyl ester **S1j** by using the general *Procedure **A*** followed by the general *Procedure **B*** and was obtained in 90% yield as colorless oil. *dr* > 98:02:0:0. R_f = 0.5 (hexane/EtOAc, 90:10, v/v). ^{1}H NMR (400 MHz, $CDCl_3$) δ 0.91-1.39 (m, 12H), 1.43-1.54 (m, 2H), 1.58-1.82 (m, 3H), 2.37-2.57 (m, 2H), 4.93-5.07 (m, 2H), 5.67-5.85 (m, 1H), 9.41 (s, 1H). ^{13}C NMR (100 MHz, $CDCl_3$) δ 9.57, 14.10, 26.61, 26.67, 27.15, 28.94, 29.72, 29.81, 38.61, 40.04, 42.22, 45.85, 115.62, 137.16, 204.50.

Step (b): The aldehyde **S1j2** was synthesized from the aldehyde **S1j1** by using the general *Procedure **I*** and was obtained as a colourless oil. Yield: 65%. *dr* > 98:02:0:0. R_f = 0.5 (hexane/EtOAc, 90:10, v/v). ^{1}H NMR (400 MHz, $CDCl_3$) δ 0.29 (t, *J* = 7.4 Hz, 1H), 0.74-0.91 (m, 4H), 0.95-1.28 (m, 8H), 1.42-1.72 (m, 5H), 1.86-2.05 (m, 2H), 2.18-2.33 (m, 2H), 4.78-5.05 (m, 2H), 5.61-5.84 (m, 1H), 9.67 (t, *J* = 2.4 Hz, 1H). ^{13}C NMR (100 MHz, $CDCl_3$) δ 10.09, 21.25, 22.56, 26.83, 26.93, 27.37, 29.69, 29.76, 30.19, 30.28, 32.93, 45.14, 46.40, 114.76, 138.23, 203.78.

Step (c): The alcohol **1r** was synthesized from the aldehyde **S1j2** by using the general *Procedure **C***.

1-((1*S,2*S**,3*R**)-3-allyl-2-cyclohexyl-1,2-dimethylcyclopropyl)propan-2-ol**: ***1r***

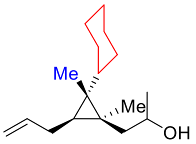

1r

The compound **1r** was obtained as a colorless oil and as a unique diastereomer except secondary alcohol with *dr*: 6:4. Yield: 67%. R_f = 0.3 (hexane/EtOAc, 90:10, v/v). ^{1}H NMR (400 MHz, $CDCl_3$) (*mixture of diastereomers*) δ 0.13-0.26 (m, 1H), 0.78-0.97 (m, 4H), 1.00-1.34 (m, 12H), 1.35-1.78 (m, 7H), 1.88-2.11 (m, 2H), 3.88-4.10 (m, 1H), 4.84-5.08 (m, 2H), 5.66-5.89 (m, 1H). ^{13}C NMR (100 MHz, $CDCl_3$) (*mixture of diastereomers*) δ 9.90 & 10.36, 20.47 & 20.51, 23.48 & 23.74, 23.85 & 24.20, 26.81 & 26.87, 26.95 & 27.00, 27.39 & 27.44, 29.37 & 29.65, 29.88 & 30.42, 30.30 & 30.34, 33.49 & 33.61, 40.43 & 40.68, 45.38 & 45.46, 65.68 & 66.92, 114.30 & 114.34, 138.99 & 139.18. HRMS (ESI): Mass calcd for $C_{17}H_{31}O$ $[M+H]^+$: 251.2375; found: 251.2359.

(xiv) *Synthesis of the compound **1s**:* The compound **1s** were synthesized from the cyclopropyl ester **S1k** according to the following scheme:

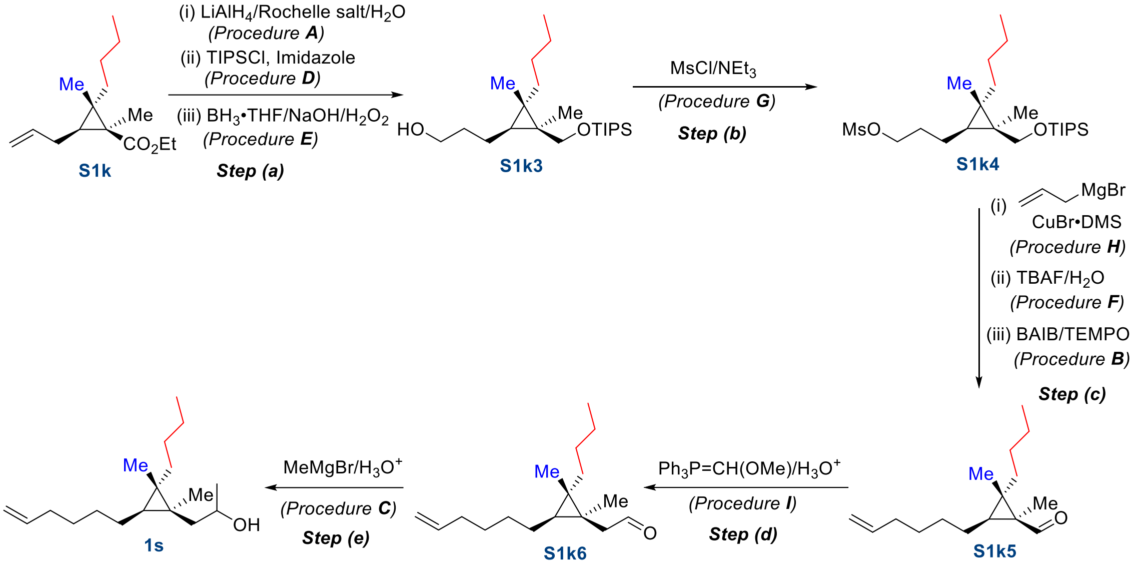

Step (a): The alcohol **S1k3** was synthesized from the cyclopropyl ester **S1k** by using *Procedure **A*** followed by the general *Procedure **D*** and *Procedure **E***. The alcohol product **S1k3** was obtained in 85% overall yield from ester **S1k** and was further used for the next step without any characterisation.

Step (b): The mesylate product **S1k4** was synthesized from the alcohol **S1k3** using the general *Procedure **G*** and was obtained as a colorless oil in 95% yield. R_f = 0.5 (20% ethyl acetate in hexane). ^{1}H NMR (400 MHz, $CDCl_3$) δ 0.13-0.25 (m, 1H), 0.87 (t, *J* = 7.1 Hz, 3H), 0.96 (s, 3H), 0.98-1.50 (m, 32H), 1.69-1.86 (m, 2H), 2.97 (s, 3H), 3.54-3.63 (m, 2H), 4.11-4.25 (m, 2H). ^{13}C NMR (100 MHz, $CDCl_3$) δ 12.25, 13.80, 14.41, 18.32, 19.35, 21.20, 23.33, 26.11, 28.07, 29.61, 30.07, 33.60, 37.59, 38.07, 65.47, 70.26.

Step (c): The aldehyde **S1k5** was synthesized from the mesylate **S1k4** by using the general *Procedure **H*** followed by *Procedure **F*** and *Procedure **B***. The aldehyde **S1k5** was obtained as a colorless oil. Overall yield from **S1k4**: 55%. ^{1}H NMR (400 MHz, $CDCl_3$) δ 0.87 (t, *J* = 6.9 Hz, 3H), 1.01 (t, *J* = 7.4 Hz, 1H), 1.19 (s, 3H), 1.22-1.51 (m, 13H), 1.60-1.81 (m, 2H), 1.91-2.10 (m, 2H), 4.81-5.06 (m, 2H), 5.66-5.86 (m, 1H), 9.38 (s, 1H). ^{13}C NMR (100 MHz, $CDCl_3$) δ 14.04, 14.25, 14.55, 21.13, 24.66, 28.72, 28.94, 29.69, 33.80, 36.42, 38.35, 38.40, 43.78, 114.66, 138.88, 204.96.

Step (d): The homologated aldehyde product **S1k6** was synthesized from the aldehyde **S1k5** by using the general *Procedure **I*** and was obtained as a colourless liquid in 65% yield which was used for the next step without any characterisation.

Step (e): The alcohol **1s** was synthesized from the aldehyde **S1k6** by using the general *Procedure **C***.

1-((1*S,2*S**,3*S**)-2-butyl-3-(hex-5-en-1-yl)-1,2-dimethylcyclopropyl)propan-2-ol**: ***1s***

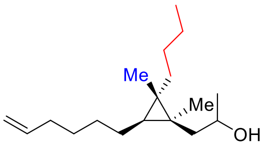

1s

The compound **1s** was obtained as a colorless oil and as a unique diastereomer except secondary alcohol with *dr*: 6:4. Yield: 95%. R_f = 0.3 (hexane/EtOAc, 90:10, v/v). ^{1}H NMR (400 MHz, $CDCl_3$) (*mixture of diastereomers*) δ 0.06-0.15 (m, 1H), 0.77-0.97 (m, 6H), 1.00-1.55 (m, 21H), 1.94-2.07 (m, 2H), 3.87-4.09 (m, 1H), 4.81-5.04 (m, 2H), 5.68-5.88 (m, 1H). ^{13}C NMR (100 MHz, $CDCl_3$) (*mixture of diastereomers*) δ 14.34 & 14.38, 14.77 & 15.18, 21.19 & 21.22, 22.88, 23.46, 23.61 & 23.82, 24.81 & 25.15, 25.50 & 25.87, 29.17, 29.69 & 29.93, 34.01, 34.55 & 34.66, 38.23 & 38.30, 40.47 & 40.56, 65.80 & 67.06, 114.36 & 114.38, 139.29 & 139.32. HRMS (ESI): Mass calcd for $C_{18}H_{33}O$ $[M+H]^+$: 267.2688; found: 267.2687.

(xv) *Synthesis of the compound* ***1a**$_{anti}$*: The alcohol **1a**$_{anti}$ was obtained from cyclopropyl ester **S1m by** using the general *Procedure **A*** according to the following scheme:

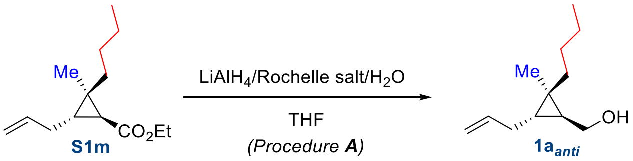

((1*S,2*R**,3*S**)-3-allyl-2-butyl-2-methylcyclopropyl)methanol**: ***1a**$_{anti}$*

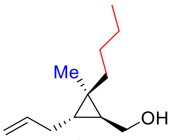

1a$_{anti}$

The compound **1a**$_{anti}$ was obtained as a colorless oil. Yield: 73%. *dr* = 90:10:0:0. R_f = 0.2 (hexane/EtOAc, 90:10, v/v). ^{1}H NMR (400 MHz, $CDCl_3$) δ 0.39-0.48 (m, 1H), 0.56-0.65 (m, 1H), 0.87 (t, *J* = 7.0 Hz, 3H), 1.03 (s, 3H), 1.12-1.38 (m, 7H), 1.94-2.17 (m, 2H), 3.60 (d, *J* = 7.0 Hz, 2H), 4.87-5.10 (m, 2H), 5.76-5.94 (m, 1H). ^{13}C NMR (100 MHz, $CDCl_3$) δ 14.35, 19.24, 23.30, 24.77, 28.41, 29.49, 33.38, 33.68, 35.88, 63.63, 114.58, 138.71. HRMS (ESI): Mass calcd for $C_{12}H_{22}O$ $[M]^+$: 182.1671; found: 182.1714.

(xvi) *Synthesis of the compound **5**:* The deuterated alcohol **5** was synthesized from the cyclopropyl ester **S1a** according to following scheme:

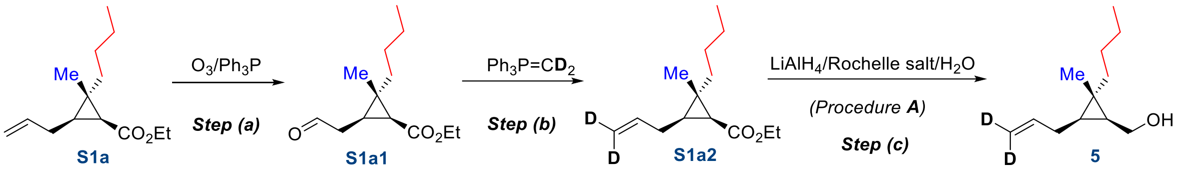

Step (a): To a solution of cyclopropyl ester **S1a** (448 mg, 2 mmol) in DCM (10 mL), the ozone gas was bubbled through for 5 min at -78 °C (until pale blue coloration was observed). Then, triphenylphosphine (2.1 g, 8 mmol) was added and the reaction mixture was stirred for 12 h at room temperature. After completion (as indicated by TLC), the solvent was removed under vacuum and pentane (10 mL) was added. The obtained solid was filtered off and the residue was subjected to a column chromatography using 5-10% Et_2O in pentane as eluent to give aldehyde product **S1a1** as a colorless liquid in 72% yield. *dr* > 98:02:0:0. R_f = 0.4 (hexane/EtOAc, 90:10, v/v). ^{1}H NMR (400 MHz, $CDCl_3$) δ 0.84 (t, *J* = 7.2 Hz, 3H), 1.09 (s, 3H), 1.14-1.42 (m, 10H), 1.53 (d, *J* = 8.7 Hz, 1H), 2.74-2.97 (m, 2H), 3.94-4.11 (m, 2H), 9.74 (s, 1H).^{13}C NMR (100 MHz, $CDCl_3$) δ 11.78, 14.23, 14.47, 22.90, 25.28, 27.83, 28.67, 28.93, 38.73, 42.49, 60.17, 171.93, 201.76.

Step (b): To a solution of (methyl)triphenylphosphonium iodide-*d*$_3$ (1.22 g, 3 mmol) in THF (30 mL) in a three-neck round-bottom flask, *n*BuLi (3 mL, 1M in hexanes) was added at -30 °C and reaction mixture was allowed to stir for 30 min while increasing the temperature from -30 °C to 0 °C. After cooling down the reaction to -30 °C, compound **S1a1** (337 mg, 1.5 mmol), dissolved in THF (3 mL) was added dropwise and the reaction mixture was stirred for 1 h at -30 °C at room temperature. After completion (as indicated by TLC), the reaction mixture was quenched with saturated aqueous solution of NH_4Cl (15 mL) and extracted with Et_2O (3 x 15 mL). The combined organic layers were dried over $MgSO_4$, filtered and evaporated to give crude product **S1a2** which was purified by column chromatography using 2-5% Et_2O in pentane as eluent. The compound **S1a2** was obtained as a colorless oil in 70% yield. *dr* > 98:02:0:0. R_f = 0.5 (hexane/EtOAc, 90:10, v/v). ^{1}H NMR (400 MHz, $CDCl_3$) δ 0.86 (t, *J* = 7.1 Hz, 3H), 1.06-1.46 (m, 14H), 2.28-2.46 (m, 2H), 3.92-4.17 (m, 2H), 5.68-5.86 (m, 1H).^{13}C NMR (100 MHz, $CDCl_3$) δ 11.69, 14.31, 14.58, 22.99, 27.50, 28.09, 28.81, 29.58, 31.96, 42.95, 59.88, 113.70-114.50 (m, *C*D_2), 137.89, 172.03.

Step (c): The alcohol **5** was obtained by the reduction of the cyclopropyl ester **S1a2** by using the general *Procedure* ***A***.

((1*S,2*S**,3*R**)-3-(allyl-3,3-*d*$_2$)-2-butyl-2-methylcyclopropyl)methanol**: ***5***

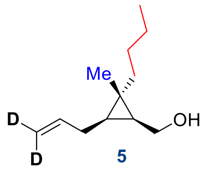

The compound **5** was obtained as a colorless oil. Yield: 90%. *dr* > 98:02:0:0. $R_f = 0.2$ (hexane/EtOAc, 90:10, v/v). ^1H NMR (400 MHz, $CDCl_3$) δ 0.71 (q, *J* = 8.0 Hz, 1H), 0.81-0.92 (m, 4H), 0.97 (s, 3H), 1.11-1.37 (m, 7H), 1.95-2.23 (m, 2H), 3.52-3.71 (m, 2H), 5.79-5.95 (m, 1H). ^{13}C NMR (100 MHz, $CDCl_3$) δ 12.38, 14.35, 22.47, 23.06, 25.79, 28.02, 28.54, 29.00, 43.01, 60.21, 113.36-114.68 (m, CD_2), 138.82. HRMS (ESI): Mass calcd for $C_{12}H_{19}D_2$ $[M-OH]^+$: 167.1769; found: 167.1768.

(xvii) *Synthesis of the compound* ***6****:* The compound **6** was synthesized from the alcohol **5** according to following scheme:

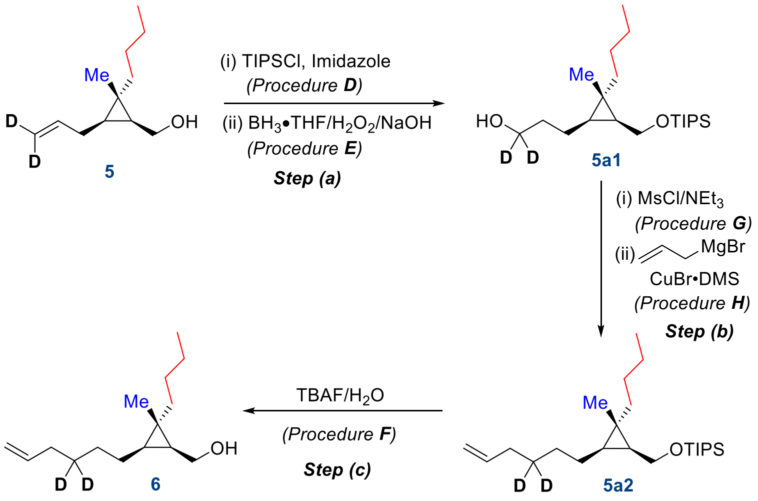

Step (a): The alcohol **5a1** was synthesized from the alcohol **5** by using the general procedure *Procedure* ***D*** followed by the general *Procedure* ***E*** and was obtained as a colorless oil in 95% overall yield from the alcohol **5**. *dr* > 98:02:0:0. $R_f = 0.4$ (hexane/EtOAc, 80/20, v/v). ^1H NMR (400 MHz, $CDCl_3$) δ 0.41-0.55 (m, 1H), 0.71-0.93 (m, 7H), 0.98-1.77 (m, 31H), 2.31 (bs, 1H), 3.57-3.67 (m, 1H), 3.72-3.81 (m, 1H). ^{13}C NMR (100 MHz, $CDCl_3$) δ 12.27, 12.39, 14.43, 18.24, 20.07, 21.53, 23.06, 26.38, 28.26, 29.06, 33.25, 43.17, 60.67, 61.05-61.52 (m, CD_2).

Step (b): The silyl ether **5a2** was synthesized from the alcohol **5a1** by using the general *Procedure* ***G*** followed by the general *Procedure* ***H***. The silyl ether product **5a2** was obtained in 60% yield and used for the next step without any characterization.

Step (c): The alcohol **6** was synthesized from the alcohol **5a2** by using the general *Procedure* ***F***.

((1*S,2*S**,3*R**)-2-butyl-3-(hex-5-en-1-yl-3,3-*d*2)-2-methylcyclopropyl)methanol**: ***6***

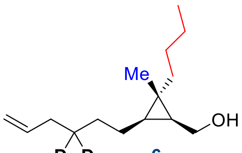

6

The compound **6** was obtained as a colorless oil. Yield: 95%. *dr* > 98:02:0:0. R_f = 0.4 (hexane/EtOAc, 80/20, v/v). ^{1}H NMR (400 MHz, $CDCl_3$) δ 0.49-0.61 (m, 1H), 0.76 (q, *J* = 8.2 Hz, 1H), 0.86 (t, *J* = 7.0 Hz, 3H), 0.94 (s, 3H), 1.07-1.38 (m, 10H), 2.00 (d, *J* = 6.7 Hz, 2H), 3.49-3.71 (m, 2H), 4.79-5.05 (m, 2H), 5.67-5.89 (m, 1H). ^{13}C NMR (100 MHz, $CDCl_3$) δ 12.32, 14.38, 22.43, 23.12, 24.32, 27.16, 27.87-28.79 (m, *C*D_2 + *1 peak at* 28.35), 29.12, 29.79, 33.75, 43.16, 60.48, 114.44, 139.22. HRMS (ESI): Mass calcd for $C_{15}H_{25}D_2$ $[M-OH]^+$: 209.2238; found: 209.2235.

(xviii) *Synthesis of the compounds* ***7-8***: The alcohols **7** and **8** were synthesized from cyclopropyl esters **S1i** and **S1h** by using the general *Procedure **A*** according to the following scheme:

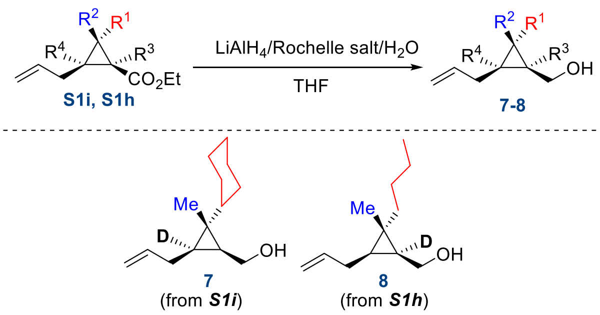

((1*S,2*S**,3*R**)-3-allyl-2-cyclohexyl-2-methylcyclopropyl-3-*d*)methanol**: ***7***

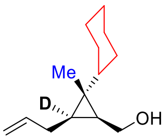

7

The compound **7** was obtained as a colorless oil. Yield: 97%. *dr* > 98:02:0:0. R_f = 0.2 (hexane/EtOAc, 90:10, v/v). ^{1}H NMR (400 MHz, $CDCl_3$) δ 0.29-0.42 (m, 1H), 0.76-0.92 (m, 4H), 1.00-1.33 (m, 6H), 1.50-1.65 (m, 3H), 1.66-1.79 (m, 2H), 1.96-2.17 (m, 2H), 3.54-3.69 (m, 2H), 4.87-5.18 (m, 2H), 5.75-5.94 (m, 1H). ^{13}C NMR (100 MHz, $CDCl_3$) δ 8.64, 25.66 (t, *J* = 24.2 Hz, *C*D), 26.83, 27.05, 28.15, 28.65, 29.53, 29.78, 50.63, 60.13, 114.61, 139.07. HRMS (ESI): Mass calcd for $C_{14}H_{24}O$ $[M+H]^+$: 210.1968; found: 210.1968.

((1*S,2*S**,3*R**)-3-allyl-2-butyl-2-methylcyclopropyl-1-*d*)methanol**: ***8***

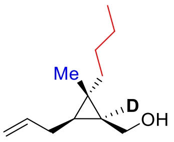

8

The compound **8** was obtained as a colorless oil. Yield: 85%. *dr* > 98:02:0:0. R_f = 0.2 (hexane/EtOAc, 90:10, v/v). ^{1}H NMR (400 MHz, $CDCl_3$) δ ^{1}H NMR (400 MHz, $CDCl_3$) δ 0.71 (t, *J* = 7.4 Hz, 1H), 0.86 (t, *J* = 7.0 Hz, 3H), 0.96 (s, 3H), 1.13-1.36 (m, 7H), 1.96-2.20 (m, 2H), 3.55-3.68 (m, 2H), 4.91-5.15 (m, 2H), 5.81-5.96 (m, 1H). ^{13}C NMR (100 MHz, $CDCl_3$) δ 12.40, 14.37, 22.40, 23.08, 25.68, 27.62 (t, *J* = 24.0 Hz, *C*D), 28.65, 29.02, 42.95, 60.19, 114.55, 139.06. HRMS (ESI): Mass calcd for $C_{12}H_{21}OD$ $[M]^+$: 183.1733; found: 183.1734.

(xix) *Synthesis of the compound* ***9***: The deuterated alcohol **9** was obtained from the cyclopropyl ester **S1a** by using the general *Procedure* ***A*** with $LiAlD_4$ instead of $LiAlH_4$ according to the following scheme:

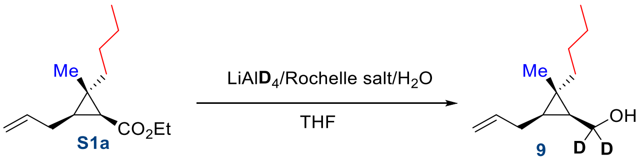

((1*S,2*S**,3*R**)-3-allyl-2-butyl-2-methylcyclopropyl)methan-*d*$_2$-ol**: ***9***

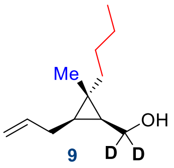

The compound **9** was obtained as a colorless oil. Yield: 70%. *dr* > 98:02:0:0. R_f = 0.2 (hexane/EtOAc, 90:10, v/v). ^{1}H NMR (400 MHz, $CDCl_3$) δ 0.70 (q, *J* = 8.3 Hz, 1H), 0.79-0.90 (m, 4H), 0.96 (s, 3H), 1.13-1.36 (m, 7H), 1.97-2.18 (m, 2H), 4.93-5.12 (m, 2H), 5.82-5.95 (m, 1H). ^{13}C NMR (100 MHz, $CDCl_3$) δ 12.36, 14.33, 22.40, 23.05, 25.72, 27.81, 28.64, 28.98, 42.98, 59.47 (m, J = 20.7 Hz, *C*D_2), 114.51, 139.01. HRMS (ESI): Mass calcd for $C_{12}H_{19}D_2$ $[M-OH]^+$: 167.1769; found: 167.1770.

(xx) *Synthesis of the compound* ***10****:* The compound **10** was synthesized from the alcohol **8** according to the following scheme:

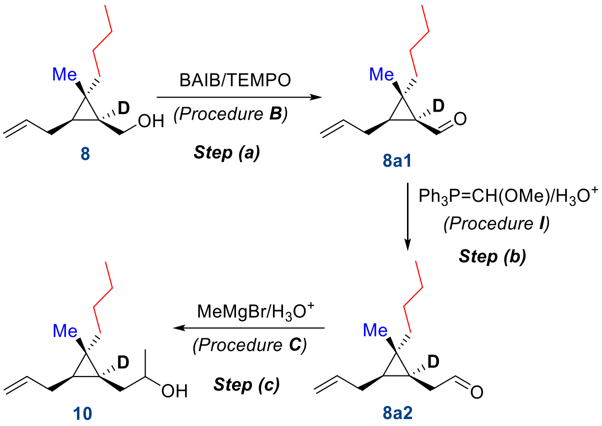

Step (a): The aldehyde **8a1** was synthesized from the alcohol **8** by using the general *Procedure* ***B*** and was obtained as a colorless oil in 85% yield. *dr* > 98:02:0:0. R_f = 0.6 (hexane/EtOAc, 90:10, v/v). ^{1}H NMR (400 MHz, $CDCl_3$) δ 0.86 (t, *J* = 7.1 Hz, 3H), 1.16-1.47 (m, 10H), 2.33-2.57 (m, 2H), 4.79-5.15 (m, 2H), 5.67-5.89 (m, 1H), 9.50 (s, 1H). ^{13}C NMR (100 MHz, $CDCl_3$) δ 12.58, 14.23, 22.87, 28.34, 28.63, 34.08, 35.81, 37.35, 37.59 (t, *J* = 24.1 Hz, *C*D), 37.84, 42.77, 115.48, 137.12, 201.94.

Step (b): The aldehyde **8a2** was synthesized from the aldehyde **8a1** by using the general *Procedure* ***I*** and was obtained in 58% yield which was used for the next step without any characterisation.

Step (c): The alcohol **10** was synthesized from the aldehyde **8a2** by using the general *Procedure* ***C***.

1-((1*S,2*S**,3*R**)-3-allyl-2-butyl-2-methylcyclopropyl-1-*d*)propan-2-ol**: ***10***

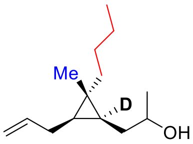

10

The compound **10** was obtained as a colorless oil and as a unique diastereomer except secondary alcohol with *dr*: 1:1. Yield: 85%. R_f = 0.3 (hexane/EtOAc, 90:10, v/v). ^{1}H NMR (400 MHz, $CDCl_3$) δ 0.46-0.60 (m, 1H), 0.78-0.94 (m, 6H), 1.08-1.47 (m, 12H), 1.86-2.08 (m, 2H), 3.69-3.91 (m, 1H), 4.85-5.11 (m, 2H), 5.72-5.94 (m, 1H). ^{13}C NMR (100 MHz, $CDCl_3$) δ 12.56 & 12.63, 14.36, 20.94 & 21.15, 21.61-22.56 (m, *C*D), 23.19, 23.35 & 23.52, 24.73, 28.97 & 29.04, 34.05, 43.27, 68.80 & 68.96, 114.31 & 114.35, 138.85 & 138.87. HRMS (ESI): Mass calcd for $C_{14}H_{26}OD$ $[M+H]^+$: 212.2125; found: 212.2157.

Supplementary Table 1. *Optimization of the reaction conditions of compound **2a** (the Heck arylation and ring opening)*

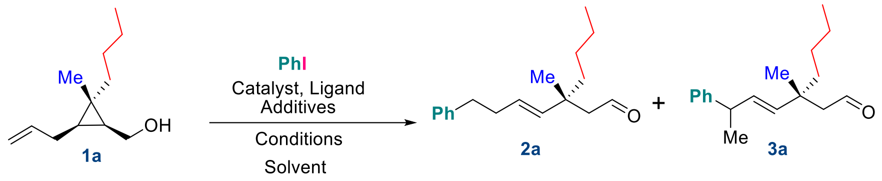

Catalyst (5 mol%)	Ligand (15 mol%)	Additives	Solvent[a]	Conditions	Conversion (GC)	Products ratio (GC) 2a:3a
$Pd(OAc)_2$	2,2-Dipyridyl	$NaHCO_3$(2.5 eq)<br>TBA-Cl (2 eq)	THF	2d, 85 °C	55%	88:12
$Pd(OAc)_2$	2,2-Dipyridyl	LiOAc (2.5 eq)<br>LiCl (2.5 eq)<br>TBA-Cl (2 eq)	THF or DMF or DMSO	2d, 85 °C	<5%	-
$PdCl_2$	XPhos or dppf	$NaHCO_3$(2.5 eq)<br>TBA-Cl (2 eq)	THF or DMF or Dioxane	24 h, 90 °C	>98%	80:20
$PdCl_2$	2,2-Dipyridyl	$CsCO_3$ (2.5 eq)<br>TBA-Cl (2 eq)	THF	24 h, 105 °C	90%	84:16
$Pd(PPh_3)_4$	2,2-Dipyridyl	$NaHCO_3$(2.5 eq)<br>TBA-Cl (2 eq)	DMSO	24 h at 90 °C	>98%	72:28
$Pd(OAc)_2$	2,2-Dipyridyl[c]	$NaHCO_3$(2.5 eq)<br>TBA-Cl (2 eq)	DMSO	24 h, rt	60%	80:20
$Pd(OAc)_2$	2,2-Dipyridyl	$NaHCO_3$(2.5 eq)<br>TBA-Cl (2 eq)	THF + Water	24 h, 90 °C	>98%	**50:50**
$Pd(OAc)_2$	None	$NaHCO_3$(2.5 eq)<br>TBA-Cl (2 eq)	THF	24 h at 90 °C	>98%	85:15
**$Pd(OAc)_2$**	**($p$$CF_3$-Ph)$_3$P**	**$NaHCO_3$ (2.5 eq)<br>TBA-Cl (2 eq)**	**THF**	**24 h at 90 °C**	>98%	**90:10**
$Pd(OAc)_2$	($p$$CF_3$-Ph)$_3$P	$NaHCO_3$(2.5 eq)<br>TBA-Cl (2 eq)	Toluene	24 h at 90 °C	>98%	88:12
$Pd(OAc)_2$	($p$$CF_3$-Ph)$_3$P	$NaHCO_3$(2.5 eq)<br>TBA-Cl (2 eq)	THF	24 h at 90°C	>98%	90:10[b]
$Pd(OAc)_2$	($p$$CF_3$-Ph)$_3$P	$NaHCO_3$(2.5 eq)<br>TBA-Cl (2 eq)	THF	24 h at 90 °C	0%	--
$Pd(OAc)_2$	($p$$CF_3$-Ph)$_3$P	*(No base)*<br>TBA-Cl (2 eq)	THF	24 h at 90 °C	0%	--
$Pd(OAc)_2$	($p$$CF_3$-Ph)$_3$P	$NaHCO_3$(2.5 eq)<br>*(No TBA-Cl)*	THF	24 h at 90 °C	0%	--

[a]*0.4 mL of solvent is used per 0.2 mmol of substrate;* [b]*PhBr is used instead of PhI.* [c]*22.5 mol% of ligand is used instead of 5 mol%*

Synthesis of the compound *2a-2z, 2aa-2ap, 11-16*

The compound **2a-2z, 2aa-2ap** and **11-16** were synthesized from alcohols **1a-1s** and **5-10** using following reaction scheme:

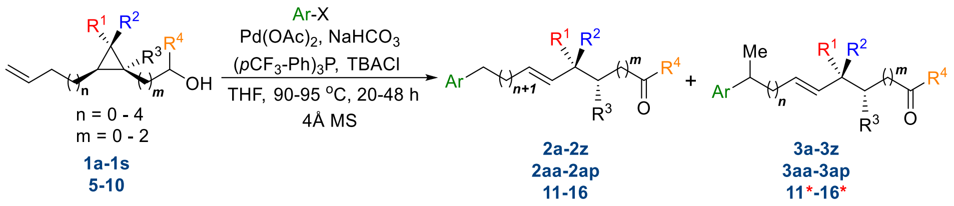

General procedure: A reaction vial (10 mL capacity) with magnetic stir bar, was charged with the given alcohol **1a-1s/5-10** (0.2 mmol), aryl bromide/iodide (0.24 mmol), $Pd(OAc)_2$ (2.24 mg, 5 mol%), $(pCF_3\text{-}Ph)_3P$ (14 mg, 15 mol%), $NaHCO_3$ (42 mg, 2.5 mmol), pre-activated molecular sieves (4Å size, powdered, 30 mg) and tetrabutylammonium chloride (TBACl) (2 mmol) followed by addition of dry THF (0.4 mL). The reaction mixture was stirred for 10 minutes at room temperature to obtain a homogeneous solution and was then placed on a pre-heated oil bath at 90-95 °C for 24-48 h. After completion of the reaction (as monitored by TLC), the reaction mixture was cooled down to room temperature, diluted with Et_2O (5 mL) and filtered. The obtained residue was concentrated and subjected to a column chromatography using 3-8% Et_2O in hexane as eluent to afford product **2a-2z**, **2aa-2ap** and **11-16** as a mixture with their corresponding inseparable regioisomers **3a-3z**, **3aa-3ap** and **11*-16***.

(*R*,E*)-3-butyl-3-methyl-7-phenylhept-4-enal: ***2a***

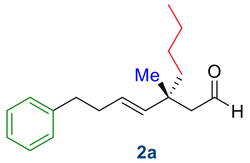

2a

The compound **2a** was prepared from the alcohol **1a** and iodobenzene. The compound **2a** was isolated as a major isomer along with its regioisomer **3a** in a ratio of 10:1. Combined Yield: 73%. *E/Z* > 99:01. R_f = 0.5 (hexane/EtOAc, 90:10, v/v). ^{1}H NMR (600 MHz, $CDCl_3$) (*major regioisomer*) δ 0.84 (t, *J* = 7.3 Hz, 3H), 1.04 (s, 3H), 1.06-1.14 (m, 2H), 1.17-1.25 (m, 2H), 1.25-1.35 (m, 2H), 2.18 (dd, *J* = 14.3, 3.6 Hz, 1H), 2.27 (dd, *J* = 15.0, 2.9 Hz, 1H), 2.29-2.39 (m, 2H), 2.64 (t, *J* = 7.3 Hz, 2H), 5.25-5.57 (m, 2H), 7.04-7.19 (m, 3H), 7.20-7.30 (m, 2H), 9.54 (t, *J* = 3.2 Hz, 1H). ^{13}C NMR (100 MHz, $CDCl_3$) (*major regioisomer*) δ 14.26, 23.44, 23.36, 26.19, 34.69, 36.20, 38.29, 41.97, 53.90, 126.01, 127.95, 128.46, 128.70, 138.10, 141.89, 204.26. HRMS (ESI): Mass calcd for $C_{18}H_{26}ONa$ $[M+Na]^+$: 281.1881; found: 281.1879.

(*R,*E*)-3-butyl-7-(4-methoxyphenyl)-3-methylhept-4-enal**: ***2b***

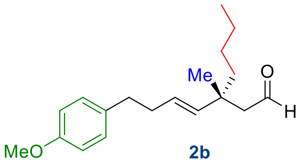

2b

The compound **2b** was prepared from the alcohol **1a** and 4-bromoanisole. The compound **2b** was isolated as a major isomer along with its regioisomer **3b** in a ratio of 4.9:1. Combined Yield: 58%. *E/Z* > 99:01. R_f = 0.3 (hexane/EtOAc, 90:10, v/v). ^{1}H NMR (400 MHz, $CDCl_3$) (*major regioisomer*) δ 0.85 (t, *J* = 7.2 Hz, 3H), 1.02-1.40 (m, 9H), 2.09-2.41 (m, 4H), 2.60 (t, *J* = 7.6 Hz, 2H), 3.76 (s, 3H), 5.29-5.56 (m, 2H), 6.72-6.86 (m, 2H), 6.99-7.09 (m, 2H), 9.58 (t, *J* = 3.2 Hz, 1H). ^{13}C NMR (100 MHz, $CDCl_3$) (*major regioisomer*) δ 14.24, 23.44, 24.39, 26.21, 34.94, 35.31, 38.30, 41.99, 53.89, 55.45, 113.90, 128.06, 129.56, 134.02, 138.02, 157.97, 204.24. HRMS (ESI): Mass calcd for $C_{19}H_{29}O_2$ $[M+H]^+$: 289.2193; found: 259.2162.

(*R,*E*)-3-butyl-7-(2-methoxyphenyl)-3-methylhept-4-enal**: ***2c***

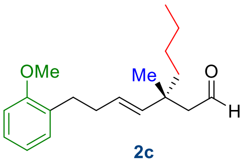

2c

The compound **2c** was prepared from the alcohol **1a** and 2-bromoanisole. The compound **2c** was isolated as a major isomer along with its regioisomer **3c** in a ratio of 5.3:1. Combined Yield: 79%. *E/Z* > 98:02. R_f = 0.4 (hexane/EtOAc, 90:10, v/v).^{1}H NMR (400 MHz, $CDCl_3$) (*major regioisomer*) δ 0.85 (t, *J* = 7.2 Hz, 3H), 0.98-1.39 (m, 9H), 2.07-2.45 (m, 4H), 2.66 (t, *J* = 7.5 Hz, 2H), 3.80 (s, 3H), 5.26-5.60 (m, 2H), 6.72-6.95 (m, 2H), 6.99-7.21 (m, 2H), 9.57 (t, *J* = 3.0 Hz, 1H). ^{13}C NMR (100 MHz, $CDCl_3$) (*major regioisomer*) δ 14.26, 23.46, 24.40, 26.19, 30.51, 33.13, 38.25, 42.02, 53.94, 55.42, 110.40, 120.49, 127.24, 128.58, 130.15, 130.30, 137.56, 157.64, 204.47. HRMS (ESI): Mass calcd for $C_{19}H_{29}O_2$ $[M+H]^+$: 289.2193; found: 289.2156.

(*R,*E*)-3-butyl-7-(4-chlorophenyl)-3-methylhept-4-enal**: ***2d***

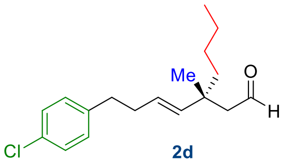

2d

The compound **2d** was prepared from the alcohol **1a** and 4-chloro-1-bromobenzene. The compound **2d** was isolated as a major isomer along with its regioisomer **3d** in a ratio of 7.3:1. Combined Yield:

61%. *E/Z* >98:02. R$_f$ = 0.5 (hexane/EtOAc, 90:10, v/v). ^{1}H NMR (400 MHz, CDCl$_3$) (*major regioisomer*) δ 0.85 (t, *J* = 7.3 Hz, 3H), 1.00-1.36 (m, 9H), 2.14-2.39 (m, 4H), 2.63 (t, *J* = 7.5 Hz, 2H), 5.27-5.49 (m, 2H), 7.06 (d, *J* = 8.5 Hz, 2H), 7.21 (d, *J* = 8.5 Hz, 2H), 9.60 (t, *J* = 3.1 Hz, 1H). ^{13}C NMR (100 MHz, CDCl$_3$) (*major regioisomer*) δ 14.24, 23.41, 24.33, 26.19, 34.54, 35.51, 38.34, 41.95, 53.82, 127.50, 128.54, 130.05, 131.71, 138.44, 140.30, 204.02. HRMS (ESI): Mass calcd for $C_{18}H_{24}OCl$ [M-H]$^-$: 291.1516; found: 291.1510.

(*R,*E*)-3-butyl-7-(2-chlorophenyl)-3-methylhept-4-enal**: ***2e***

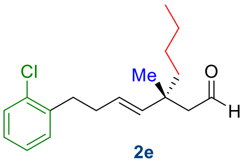

2e

The compound **2e** was prepared from the alcohol **1a** and 2-chloro-1-iodobenzene. The compound **2e** was isolated as a major isomer along with its regioisomer **3e** in a ratio of 7.3:1. Combined Yield: 74%. *E/Z* > 99:01. R$_f$ = 0.5 (hexane/EtOAc, 90:10, v/v). ^{1}H NMR (400 MHz, CDCl$_3$) (*major regioisomer*) δ 0.85 (t, *J* = 7.3 Hz, 3H), 1.02-1.38 (m, 9H), 2.04-2.51 (m, 4H), 2.79 (t, *J* = 7.5 Hz, 2H), 5.24-5.59 (m, 2H), 6.99-7.40 (m, 4H), 9.57 (t, *J* = 3.2 Hz, 1H). ^{13}C NMR (100 MHz, CDCl$_3$) (*major regioisomer*) δ 14.25, 23.42, 24.31, 26.17, 33.03, 33.77, 33.28, 41.92, 53.85, 126.81, 127.53, 129.63, 130.78, 134.13, 138.34, 139.39, 204.18. HRMS (ESI): Mass calcd for $C_{18}H_{24}OCl$ [M-H]$^-$: 291.1516; found: 291.1548.

(*R,*E*)-3-butyl-7-(4-fluorophenyl)-3-methylhept-4-enal**: ***2f***

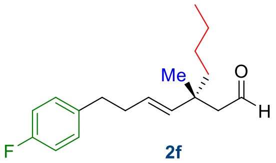

2f

The compound **2f** was prepared from the alcohol **1a** and 4-fluoro-1-iodobenzene. The compound **2f** was isolated as a major isomer along with its regioisomer **3f** in a ratio of 5.7:1. Combined Yield: 63%. *E/Z* > 99:01. R$_f$ = 0.5 (hexane/EtOAc, 90:10, v/v). ^{1}H NMR (400 MHz, CDCl$_3$) (*major regioisomer*) δ 0.85 (t, *J* = 7.3 Hz, 3H), 0.99-1.40 (m, 9H), 2.11-2.41 (m, 4H), 2.63 (t, *J* = 7.0 Hz, 2H), 5.23-5.57 (m, 2H), 6.83-7.16 (m, 4H), 9.60 (t, *J* = 3.1 Hz, 1H). ^{13}C NMR (100 MHz, CDCl$_3$) (*major regioisomer*) δ 14.23, 23.41, 24.33, 26.18, 34.76, 35.33, 38.31, 41.96, 53.82, 115.16 (d, *J* = 21.2 Hz), 127.63, 129.99 (d, *J* = 7.7 Hz), 137.46 (d, *J* = 3.0 Hz), 138.33, 161.42 (d, *J* = 242.5 Hz), 204.06. HRMS (ESI): Mass calcd for $C_{18}H_{25}OFNa$ [M+Na]$^+$: 299.1787; found: 299.1782.

(*R,*E*)-3-butyl-7-(2-fluorophenyl)-3-methylhept-4-enal**: ***2g***

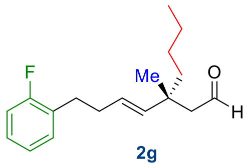

2g

The compound **2g** was prepared from the alcohol **1a** and 2-fluoro1-iodobenzene. The compound **2g** was isolated as a major isomer along with its regioisomer **3g** in a ratio of 5.7:1. Combined Yield: 66%. *E/Z* > 99:01. R_f = 0.5 (hexane/EtOAc, 90:10, v/v). ^{1}H NMR (400 MHz, $CDCl_3$) (*major regioisomer*) δ 0.84 (t, *J* = 7.2 Hz, 3H), 0.98-1.40 (m, 9H), 2.03-2.48 (m, 4H), 2.70 (t, *J* = 7.3 Hz, 2H), 5.26-5.62 (m, 2H), 6.92-7.21 (m, 2H), 9.55 (t, *J* = 3.2 Hz, 1H). ^{13}C NMR (100 MHz, $CDCl_3$) (*major regioisomer*) δ 14.24, 23.42, 24.31, 26.14, 29.31, 33.40, 38.25, 41.92, 53.85, 115.33 (d, *J* = 21.6 Hz), 124.02 (d, *J* = 3.5 Hz), 127.60, 127.74 (d, *J* = 8.1 Hz), 128.64 (d, *J* = 15.9 Hz), 130.94 (d, *J* = 5.2 Hz), 138.35, 161.33 (d, *J* = 241.9 Hz), 204.19. HRMS (ESI): Mass calcd for $C_{18}H_{24}OF$ $[M-H]^-$: 275.1811; found: 275.1816.

(*R,*E*)-7-(4-acetylphenyl)-3-butyl-3-methylhept-4-enal**: ***2h***

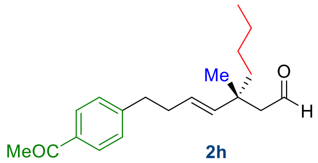

2h

The compound **2h** was prepared from the alcohol **1a** and 4'-iodoacetophenone. The compound **2h** was isolated as a major isomer along with its regioisomer **3h** in a ratio of 46:1. Combined Yield: 47%. *E/Z > 99:01*. R_f = 0.2 (hexane/EtOAc, 90:10, v/v). ^{1}H NMR (400 MHz, $CDCl_3$) (*major regioisomer*) δ 0.84 (t, *J* = 7.3 Hz, 3H), 0.98-1.38 (m, 9H), 2.15-2.42 (m, 4H), 2.56 (s, 3H), 2.72 (t, *J* = 7.5 Hz, 2H), 5.24-5.51 (m, 2H), 7.16-7.29 (m, 2H), 7.86 (d, *J* = 7.8 Hz, 2H), 9.57 (t, *J* = 3.2 Hz, 1H). ^{13}C NMR (100 MHz, $CDCl_3$) (*major regioisomer*) δ 14.24, 23.41, 24.34, 26.20, 26.75, 34.25, 36.19, 38.35, 41.92, 53.82, 127.37, 128.69, 128.94, 135.33, 138.61, 147.73, 198.03, 203.90. HRMS (ESI): Mass calcd for $C_{20}H_{29}O_2$ $[M+H]^+$: 301.2162; found: 301.2206.

(*R,*E*)-3-butyl-3-methyl-7-(thiophen-2-yl)hept-4-*enal***: ***2i***

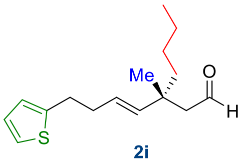

2i

The compound **2i** was prepared from the alcohol **1a** and 2-bromothiophene. The compound **2i** was isolated as a major isomer along with its regioisomer **3i** in a ratio of 3.5:1. Combined Yield: 49%. *E/Z*

> 99:01. R_f = 0.5 (hexane/EtOAc, 90:10, v/v). ^{1}H NMR (400 MHz, $CDCl_3$) (*major regioisomer*) δ 0.85 (t, *J* = 7.3 Hz, 3H), 1.01-1.46 (m, 9H), 2.11-2.53 (m, 4H), 2.88 (t, *J* = 7.5 Hz, 2H), 5.18-5.59 (m, 2H), 6.69-6.80 (m, 1H), 6.83-6.95 (m, 1H), 7.03-7.16 (m, 1H), 9.58 (t, *J* = 3.3 Hz, 1H). ^{13}C NMR (100 MHz, $CDCl_3$) (*major regioisomer*) δ 14.27, 23.44, 24.30, 26.19, 30.24, 34.95, 38.34, 41.93, 53.87, 123.23, 124.50, 126.86, 127.36, 138.67, 144.69, 204.23. HRMS (ESI): Mass calcd for $C_{16}H_{24}OSNa$ $[M+Na]^+$: 287.1446; found: 287.1442.

(*S,*E*)-3-cyclohexyl-3-methyl-7-phenylhept-4-enal**: ***2j***

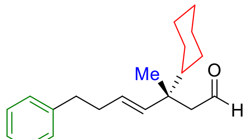

2j

The compound **2j** was prepared from the alcohol **1b** and iodobenzene. The compound **2j** was isolated as a major isomer along with its regioisomer **3j** in a ratio of 5.7:1. Combined Yield: 73%. *E/Z* > 99:01. R_f = 0.4 (hexane/EtOAc, 90:10, v/v).^{1}H NMR (400 MHz, $CDCl_3$) (*major regioisomer*) δ 0.74-0.92 (m, 2H), 0.95-1.27 (m, 7H), 1.55-1.80 (m, 5H), 2.12-2.48 (m, 4H), 2.67 (t, *J* = 7.5 Hz, 2H), 5.21-5.60 (m, 2H), 7.06-7.20 (m, 3H), 7.21-7.38 (m, 2H), 9.54 (t, *J* = 3.2 Hz, 1H). ^{13}C NMR (100 MHz, $CDCl_3$) (*major regioisomer*) δ 20.84, 26.69, 27.09, 27.24, 27.46, 34.71, 36.14, 41.01, 47.69, 51.97, 125.97, 128.44, 128.56, 128.69, 137.28, 141.84, 204.84. HRMS (ESI): Mass calcd for $C_{20}H_{29}O$ $[M+H]^+$: 285.2213; found: 285.2255.

(*S,*E*)-3-butyl-3-isobutyl-7-phenylhept-4-enal**: ***2k***

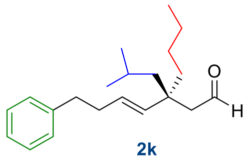

2k

The compound **2k** was prepared from the alcohol **1c** and iodobenzene. The compound **2k** was isolated as a major isomer along with its regioisomer **3k** in a ratio of 5.7:1. Combined Yield: 67%. *E/Z > 99:01*. R_f = 0.7 (hexane/EtOAc, 90:10, v/v). ^{1}H NMR (400 MHz, $CDCl_3$) (*major regioisomer*) δ 0.68-0.99 (m, 9H), 1.06-1.47 (m, 8H), 1.53-1.64 (m, 1H), 2.20-2.44 (m, 4H), 2.66 (t, *J* = 7.7 Hz, 2H), 5.21-5.52 (m, 2H), 7.07-7.30 (m, 5H), 9.61 (t, *J* = 3.1 Hz, 1H). ^{13}C NMR (100 MHz, $CDCl_3$) (*major regioisomer*) δ 14.27, 23.50, 24.14, 25.24, 25.27, 25.89, 34.87, 36.17, 38.07, 41.64, 47.93, 50.25, 126.03, 127.73, 128.49, 128.66, 138.55, 141.92, 204.34. HRMS (ESI): Mass calcd for $C_{21}H_{33}O$ $[M+H]^+$: 301.2526; found: 301.2543.

(*E*)-3,3-dimethyl-7-phenylhept-4-enal: ***2l***

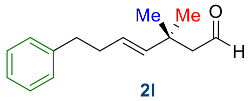

2l

The compound **2l** was prepared from the alcohol **1d** and iodobenzene. The compound **2l** was isolated as a major isomer along with its regioisomer **3l** in a ratio of 7.3:1. Combined Yield: 59%. *E/Z* > 99:01. R_f = 0.6 (hexane/EtOAc, 90:10, v/v). ^{1}H NMR (400 MHz, $CDCl_3$) (*major regioisomer*) δ 1.08 (s, 6H), 2.19-2.42 (m, 4H), 2.66 (t, *J* = 8.4 Hz, 2H), 5.30-5.63 (m, 2H), 7.09-7.20 (m, 3H), 7.22-7.30 (m, 2H), 9.56 (t, *J* = 3.2 Hz, 1H). ^{13}C NMR (100 MHz, $CDCl_3$) (*major regioisomer*) δ 28.15, 34.61, 35.37, 36.18, 55.36, 126.02, 126.96, 128.46, 128.71, 139.09, 141.93, 203.92. HRMS (ESI): Mass calcd for $C_{15}H_{21}O$ $[M+H]^+$: 217.1587; found: 217.1617.

(*S,*E*)-3-butyl-3,7-diphenylhept-4-enal**: ***2m***

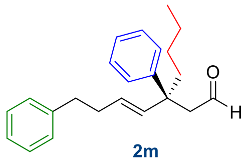

2m

The compound **2m** was prepared from the alcohol **1e** and iodobenzene. The compound **2m** was isolated as a major isomer along with its regioisomer **3m** in a ratio of 7.3:1. Combined Yield: 81%. *E/Z* > 99:01. R_f = 0.4 (hexane/EtOAc, 90:10, v/v). ^{1}H NMR (400 MHz, $CDCl_3$) (*major regioisomer*) δ 0.80 (t, *J* = 7.3 Hz, 3H), 0.92-1.08 (m, 2H), 1.12-1.26 (m, 2H), 1.63-1.91 (m, 2H), 2.32-2.56 (m, 2H), 2.58-2.90 (m, 4H), 5.39-5.77 (m, 2H), 7.01-7.44 (m, 10H), 9.38 (t, *J* = 3.0 Hz, 1H). ^{13}C NMR (100 MHz, $CDCl_3$) (*major regioisomer*) δ 14.15, 23.37, 26.30, 34.82, 35.98, 40.02, 45.54, 51.34, 126.08, 126.54, 127.10, 128.56, 128.80, 129.11, 136.98, 141.74, 145.14, 203.93. HRMS (ESI): Mass calcd for $C_{23}H_{29}O$ $[M+H]^+$: 321.2213; found: 321.2238.

(*R,*E*)-3-butyl-3-ethyl-7-phenylhept-4-enal**: ***2n***

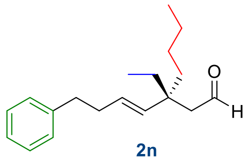

2n

The compound **2n** was prepared from the alcohol **1f** and iodobenzene. The compound **2n** was isolated as a major isomer along with its regioisomer **3n** in a ratio of 6.1:1. Combined Yield: 70%. *E/Z* > 99:01. R_f = 0.6 (hexane/EtOAc, 90:10, v/v). ^{1}H NMR (400 MHz, $CDCl_3$) (*major regioisomer*) δ 0.68-0.95 (m, 6H), 1.04-1.49 (m, 9H), 2.26 (d, *J* = 3.2 Hz, 2H), 2.31-2.46 (m, 2H), 2.67 (t, *J* = 7.1 Hz, 2H), 5.18-

5.60 (m, 2H), 7.07-7.32 (m, 5H), 9.59 (t, *J* = 3.3 Hz, 1H). ^{13}C NMR (100 MHz, $CDCl_3$) (*major regioisomer*) δ 8.02, 14.26, 23.50, 25.71, 30.43, 34.86, 36.30, 37.38, 41.28, 49.97, 126.02, 128.48, 128.56, 128.70, 137.59, 141.90, 204.34. HRMS (ESI): Mass calcd for $C_{19}H_{29}O$ $[M+H]^+$: 273.2319; found: 273.2357.

(*R,*E*)-3-(2-(benzyloxy)ethyl)-3-methyl-7-phenylhept-4-enal: *2o***

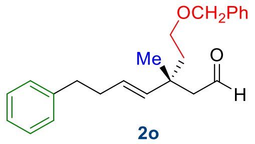

2o

The compound **2o** was prepared from the alcohol **1g** and iodobenzene. The compound **2o** was isolated as a major isomer along with its regioisomer **3o** in a ratio of 6.7:1. Combined Yield: 63%. *E/Z* > 99:01. R_f = 0.2 (hexane/EtOAc, 90:10, v/v). ^{1}H NMR (400 MHz, $CDCl_3$) (*major regioisomer*) δ 1.08 (m, 3H), 1.61-1.83 (m, 2H), 2.13-2.44 (m, 4H), 2.63 (d, *J* = 7.5 Hz, 2H), 3.30-3.50 (m, 2H), 4.40 (s, 2H), 5.28-5.62 (m, 2H), 7.02-7.39 (m, 10H), 9.56 (t, *J* = 3.0 Hz, 1H). ^{13}C NMR (100 MHz, $CDCl_3$) (*major regioisomer*) δ 24.90, 34.59, 36.09, 37.47, 41.06, 53.89, 66.96, 73.21, 126.05, 127.79, 128.28, 128.50, 128.58, 128.69, 137.59, 138.55, 141.80, 203.68. HRMS (ESI): Mass calcd for $C_{23}H_{29}O_2$ $[M+H]^+$: 337.2162; found: 337.2112.

(*R,*E*)-4-butyl-4-methyl-8-phenyloct-5-en-2-one**: ***2p***

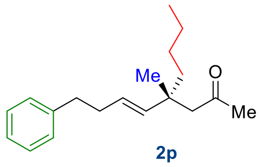

2p

The compound **2p** was prepared from the alcohol **1h** and iodobenzene. The compound **2p** was isolated as a major isomer along with its regioisomer **3p** in a ratio of 5.3:1. Combined Yield: 56%. *E/Z* > 99:01. R_f = 0.5 (hexane/EtOAc, 90:10, v/v). ^{1}H NMR (400 MHz, $CDCl_3$) (*major regioisomer*) δ 0.82 (t, *J* = 7.3 Hz, 3H), 0.95-1.37 (m, 9H), 1.99 (s, 3H), 2.19-2.45 (m, 4H), 2.63 (t, *J* = 7.5 Hz, 2H), 5.21-5.51 (m, 2H), 7.04-7.34 (m, 5H). ^{13}C NMR (100 MHz, $CDCl_3$) (*major regioisomer*) δ 14.31, 23.50, 23.71, 26.47, 32.30, 34.73, 36.60, 38.78, 41.46, 54.47, 125.94, 127.06, 128.44, 128.69, 138.76, 142.09, 208.85. HRMS (ESI): Mass calcd for $C_{19}H_{29}O$ $[M+H]^+$: 273.2218; found: 273.2276.

(*R,*E*)-4-butyl-8-(3-methoxyphenyl)-4-methyloct-5-en-2-one**: ***2q***

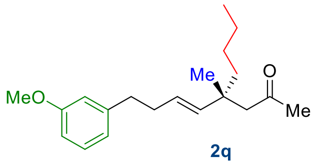

2q

The compound **2q** was prepared from the alcohol **1h** and 3-bromoanisole. The compound **2q** was isolated as a major isomer along with its regioisomer **3q** in a ratio of 10.1:1. Combined Yield: 50%. *E/Z* > 99:01. R_f = 0.4 (hexane/EtOAc, 90:10, v/v). ^{1}H NMR (400 MHz, $CDCl_3$) (*major regioisomer*) δ 0.85 (t, *J* = 7.3 Hz, 3H), 0.96-1.43 (m, 9H), 2.02 (s, 3H), 2.23-2.47 (m, 4H), 2.63 (t, *J* = 7.9 Hz, 2H), 3.77 (s, 3H), 5.17-5.58 (m, 2H), 6.58-6.85 (m, 3H), 7.10-7.20 (m, 1H). ^{13}C NMR (100 MHz, $CDCl_3$) (*major regioisomer*) δ 14.30, 23.49, 23.71, 26.47, 32.60, 34.62, 36.36, 38.79, 41.46, 54.47, 55.31, 111.24, 114.44, 121.13, 127.03, 129.39, 138.76, 143.76, 159.78, 208.87. HRMS (ESI): Mass calcd for $C_{20}H_{31}O_2$ $[M+H]^+$: 303.2324; found: 303.2342.

(*R,*E*)-4-butyl-8-(4-methoxyphenyl)-4-methyloct-5-en-2-one**: ***2r***

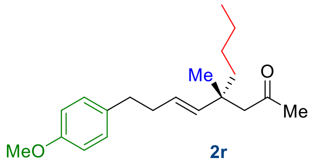

2r

The compound **2r** was prepared from the alcohol **1h** and 4-bromoanisole. The compound **2r** was isolated as a major isomer along with its regioisomer **3r** in a ratio of 6.1:1. Combined Yield: 60%. *E/Z* > 99:01. R_f = 0.4 (hexane/EtOAc, 90:10, v/v). ^{1}H NMR (400 MHz, $CDCl_3$) (*major regioisomer*) δ 0.85 (t, *J* = 7.3 Hz, 3H), 0.96-1.39 (m, 9H), 2.02 (s, 3H), 2.20-2.46 (m, 4H), 2.59 (t, *J* = 7.7 Hz, 2H), 3.76 (s, 3H), 5.21-5.46 (m, 2H), 6.79 (d, *J* = 8.6 Hz, 3H), 7.06 (d, *J* = 8.6 Hz, 3H). ^{13}C NMR (100 MHz, $CDCl_3$) (*major regioisomer*) δ 14.31, 23.50, 23.72, 26.48, 32.62, 34.99, 35.39, 38.79, 41.48, 54.49, 55.44, 113.86, 127.17, 129.56, 134.22, 138.69, 157.91, 208.88. HRMS (ESI): Mass calcd for $C_{20}H_{31}O_2$ $[M+H]^+$: 303.2324; found: 303.2383.

(*S,*E*)-4-butyl-4-isobutyl-8-phenyloct-5-en-2-one**: ***2s***

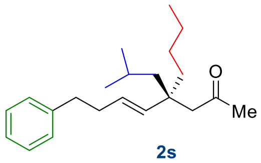

2s

The compound **2s** was prepared from the alcohol **1i** and iodobenzene. The compound **2s** was isolated as a major isomer along with its regioisomer **3s** in a ratio of 7.3:1. Combined Yield: 57%. *E/Z* > 99:01. R_f = 0.5 (hexane/EtOAc, 90:10, v/v). ^{1}H NMR (400 MHz, $CDCl_3$) (*major regioisomer*) δ 0.73-0.91

(m, 9H), 0.98-1.14 (m, 2H), 1.16-1.54 (m, 7H), 2.02 (s, 3H), 2.24-2.49 (m, 4H), 2.65 (t, J = 7.6 Hz, 2H), 5.19-5.43 (m, 2H), 7.09-7.29 (m, 5H). ^{13}C NMR (100 MHz, $CDCl_3$) (*major regioisomer*) δ 14.36, 23.53, 24.31, 25.22, 25.27, 26.18, 32.55, 34.95, 36.27, 36.99, 41.92, 47.15, 50.03, 125.95, 126.68, 128.46, 128.64, 139.50, 142.15, 208.51. HRMS (ESI): Mass calcd for $C_{20}H_{35}O$ $[M+H]^+$: 315.2682; found: 315.2781.

(*S,*E*)-4-butyl-4-isobutyl-8-(3-methoxyphenyl)oct-5-en-2-one**: ***2t***

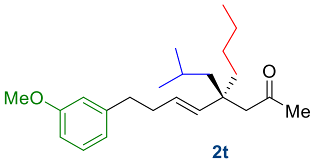

2t

The compound **2t** was prepared from the alcohol **1i** and 3-bromoanisole. The compound **2t** was isolated as a major isomer along with its regioisomer **3t** in a ratio of 13.3:1. Combined Yield: 61%. *E/Z* > 99:01. R_f = 0.5 (hexane/EtOAc, 90:10, v/v). ^{1}H NMR (400 MHz, $CDCl_3$) (*major regioisomer*) δ 0.73-0.91 (m, 9H), 0.98-1.14 (m, 2H), 1.16-1.54 (m, 7H), 2.03 (s, 3H), 2.27-2.51 (m, 4H), 2.63 (t, J = 7.6 Hz, 2H), 3.77 (m, 3H), 5.15-5.45 (m, 2H), 6.63-6.82 (m, 3H), 7.11-7.22 (m, 1H).^{13}C NMR (100 MHz, $CDCl_3$) (*major regioisomer*) δ 14.34, 23.53, 24.31, 25.21, 25.27, 26.17, 32.55, 34.83, 36.32, 36.99, 41.92, 47.15, 50.04, 55.31, 111.24, 114.41, 121.08, 126.64, 129.41, 139.50, 143.80, 159.80, 208.50. HRMS (ESI): Mass calcd for $C_{23}H_{37}O_2$ $[M+H]^+$: 345.2788; found: 345.2799.

(*R,*E*)-3-butyl-3-methyl-8-phenyloct-4-enal**: ***2u***

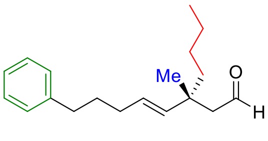

2u

The compound **2u** was prepared from the alcohol **1j** and iodobenzene. The compound **2u** was isolated as a major isomer along with its regioisomer **3u** in a ratio of 10.1:1. Combined Yield: 57%. *E/Z* > 99:01. R_f = 0.4 (hexane/EtOAc, 90:10, v/v). ^{1}H NMR (400 MHz, $CDCl_3$) (*major regioisomer*) δ 0.87 (t, J = 6.7 Hz, 3H), 1.01 (s, 3H), 1.14-1.41 (m, 6H), 1.60-1.75 (m, 2H), 1.98-2.13 (m, 2H), 2.19-2.40 (m, 2H), 2.58 (t, J = 7.6 Hz, 2H), 5.18-5.53 (m, 2H), 7.06-7.34 (m, 5H), 9.70 (t, J = 3.1 Hz, 1H). ^{13}C NMR (100 MHz, $CDCl_3$) (*major regioisomer*) δ 14.28, 23.46, 24.43, 26.30, 31.54, 32.47, 35.56, 38.35, 42.00, 53.89, 125.91, 128.49, 128.57, 128.63, 137.71, 142.61, 204.17. HRMS (ESI): Mass calcd for $C_{19}H_{29}O$ $[M+H]^+$: 273.2213; found: 273.2254.

(*R,*E*)-3-butyl-8-(4-methoxyphenyl)-3-methyloct-4-enal**: ***2v***

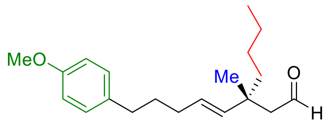

2v

The compound **2v** was prepared from the alcohol **1j** and 4-bromoanisole. The compound **2v** was isolated as a major isomer along with its regioisomer **3v** in a ratio of 5:1. Combined Yield: 64%. *E/Z > 99:01*. R_f = 0.4 (hexane/EtOAc, 90:10, v/v). ^{1}H NMR (400 MHz, $CDCl_3$) (*major regioisomer*) δ 0.87 (t, *J* = 7.0 Hz, 3H), 1.09 (s, 3H), 1.15-1.38 (m, 6H), 1.59-1.71 (m, 2H), 1.96-2.13 (m, 2H), 2.20-2.39 (m, 2H), 2.52 (t, *J* = 7.3 Hz, 2H), 3.77 (s, 3H), 5.13-5.53 (m, 2H), 6.81 (d, *J* = 8.5 Hz, 2H), 7.07 (d, *J* = 8.5 Hz, 2H), 9.70 (t, *J* = 3.0 Hz, 1H). ^{13}C NMR (100 MHz, $CDCl_3$) (*major regioisomer*) δ 14.28, 23.45, 24.42, 26.28, 31.76, 32.40, 34.60, 38.33, 42.00, 53.89, 55.45, 113.90, 128.62, 129.48, 134.67, 137.63, 157.88, 204.16. HRMS (ESI): Mass calcd for $C_{20}H_{31}O_2$ $[M+H]^+$: 303.2319; found: 303.2333.

(*R,*E*)-3-butyl-8-(2-fluorophenyl)-3-methyloct-4-enal**: ***2w***

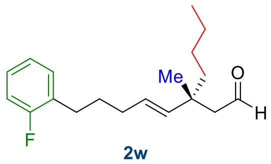

2w

The compound **2w** was prepared from the alcohol **1j** and 2-fluoro-1-iodobenzene. The compound **2w** was isolated as a major isomer along with its regioisomer **2w** in a ratio of 5.3:1. Combined Yield: 79%. *E/Z > 99:01*. R_f = 0.5 (hexane/EtOAc, 90:10, v/v). ^{1}H NMR (400 MHz, $CDCl_3$) (*major regioisomer*) δ 0.86 (t, *J* = 7.1 Hz, 3H), 1.09 (s, 3H), 1.13-1.42 (m, 6H), 1.59-1.77 (m, 2H), 1.99-2.41 (m, 4H), 2.61 (t, *J* = 7.6 Hz, 2H), 5.20-5.56 (m, 2H), 6.90-7.20 (m, 4H), 9.70 (t, *J* = 3.2 Hz, 1H). ^{13}C NMR (100 MHz, $CDCl_3$) (*major regioisomer*) δ 14.26, 23.45, 24.41, 26.27, 28.61, 30.23, 32.48, 38.34, 41.97, 53.88, 115.36 (d, *J* = 22.6 Hz), 124.05 (d, *J* = 3.3 Hz), 127.58 (d, *J* = 8.1 Hz), 128.37, 129.35 (d, *J* = 16.4 Hz), 130.81 (d, *J* = 4.8 Hz), 137.83, 161.32 (d, *J* = 240.2 Hz), 204.15. HRMS (ESI): Mass calcd for $C_{19}H_{27}OFNa$ $[M+Na]^+$: 313.1944; found: 313.1942.

(*R,*E*)-3-butyl-8-(2-chlorophenyl)-3-methyloct-4-enal**: ***2x***

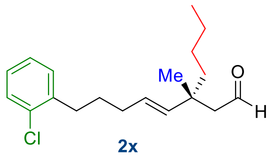

2x

The compound **2x** was prepared from the alcohol **1j** and 2-chloro-1-iodobenzene. The compound **2x** was isolated as a major isomer along with its regioisomer **3x** in a ratio of 7.3:1. Combined Yield: 64%.

E/Z > 99:01. R_f = 0.5 (hexane/EtOAc, 90:10, v/v). ^{1}H NMR (400 MHz, $CDCl_3$) (*major regioisomer*) δ 0.87 (t, *J* = 7.1 Hz, 3H), 1.10 (s, 3H), 1.15-1.40 (m, 6H), 1.61-1.73 (m, 2H), 2.02-2.19 (m, 2H), 2.20-2.40 (m, 2H), 2.69 (t, *J* = 7.7 Hz, 2H), 5.17-5.53 (m, 2H), 7.05-7.22 (m, 3H), 7.27-7.36 (m, 1H), 9.71 (t, *J* = 3.2 Hz, 1H). ^{13}C NMR (100 MHz, $CDCl_3$) (*major regioisomer*) δ 14.27, 23.46, 24.44, 26.28, 29.81, 32.59, 33.25, 38.35, 41.98, 53.89, 126.88, 127.41, 128.41, 129.64, 130.55, 134.09, 137.87, 140.18, 204.15. HRMS (ESI): Mass calcd for $C_{19}H_{26}OCl$ $[M-H]^-$: 305.1672; found: 305.1670.

(*R,*E*)-3-butyl-3-methyl-10-phenyldec-4-enal**: ***2y***

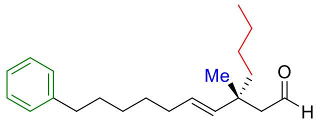

2y

The compound **2y** was prepared from the alcohol **1k** and iodobenzene. The compound **2y** was isolated as a major isomer along with its regioisomer **3y** in a ratio of 4.6:1. Combined Yield: 84%. *E/Z* > 99:01. R_f = 0.4 (hexane/EtOAc, 90:10, v/v). ^{1}H NMR (400 MHz, $CDCl_3$) (*major regioisomer*) δ 0.87 (t, *J* = 7.2 Hz, 3H), 1.08 (s, 3H), 1.12-1.42 (m, 10H), 1.55-1.65 (m, 2H), 1.94-2.06 (m, 2H), 2.18-2.36 (m, 2H), 2.58 (t, *J* = 7.5 Hz, 2H), 5.25-5.44 (m, 2H), 7.10-7.30 (m, 5H), 9.68 (t, *J* = 3.2 Hz, 1H). ^{13}C NMR (100 MHz, $CDCl_3$) (*major regioisomer*) δ 14.30, 23.46, 24.41, 26.28, 28.91, 29.62, 31.50, 32.83, 36.12, 38.29, 42.03, 53.92, 125.80, 128.44, 128.59, 128.94, 137.29, 142.98, 204.29. HRMS (ESI): Mass calcd for $C_{21}H_{33}O$ $[M+H]^+$: 301.2526; found: 301.2584.

(*R,*E*)-3-butyl-10-(3-methoxyphenyl)-3-methyldec-4-enal**: ***2z***

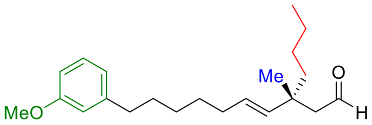

2z

The compound **2z** was prepared from the alcohol **1k** and 3-bromoanisole. The compound **2z** was isolated as a major isomer along with its regioisomer **3z** in a ratio of 8.1:1. Combined Yield: 54%. *E/Z* > 99:01. R_f = 0.4 (hexane/EtOAc, 90:10, v/v). ^{1}H NMR (400 MHz, $CDCl_3$) (*major regioisomer*) δ 0.86 (t, *J* = 7.2 Hz, 3H), 1.08 (s, 3H), 1.12-1.42 (m, 10H), 1.54-1.65 (m, 2H), 1.92-2.06 (m, 2H), 2.15-2.37 (m, 2H), 2.55 (t, *J* = 7.7 Hz, 2H), 3.78 (s, 3H), 5.20-5.46 (m, 2H), 6.65-6.83 (m, 3H), 7.11-7.20 (m, 1H), 9.68 (t, *J* = 3.2 Hz, 1H). ^{13}C NMR (100 MHz, $CDCl_3$) (*major regioisomer*) δ 14.29, 23.46, 24.40, 26.28, 28.92, 29.62, 31.36, 32.83, 36.16, 38.28, 42.02, 53.92, 55.32, 111.00, 114.41, 121.04, 129.93, 129.37, 137.29, 144.64, 159.77, 204.29. HRMS (ESI): Mass calcd for $C_{22}H_{34}O_2Na$ $[M+Na]^+$: 353.2454; found: 353.2457.

(*R,*E*)-3-butyl-10-(4-methoxyphenyl)-3-methyldec-4-enal**: ***2aa***

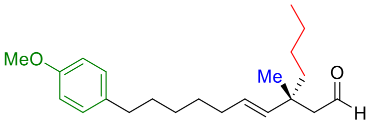

2aa

The compound **2aa** was prepared from the alcohol **1k** and 4-bromoanisole. The compound **2aa** was isolated as a major isomer along with its regioisomer **3aa** in a ratio of 5.7:1. Combined Yield: 74%. *E/Z* > 99:01. R_f = 0.4 (hexane/EtOAc, 90:10, v/v). ^{1}H NMR (400 MHz, $CDCl_3$) (*major regioisomer*) δ 0.87 (t, *J* = 7.0 Hz, 3H), 1.08 (s, 3H), 1.11-1.40 (m, 10H), 1.50-1.62 (m, 2H), 1.91-2.07 (m, 2H), 2.17-2.37 (m, 2H), 2.52 (t, *J* = 7.7 Hz, 2H), 3.77 (s, 3H), 5.13-5.57 (m, 2H), 6.80 (d, *J* = 8.4 Hz, 2H), 7.07 (d, *J* = 8.4 Hz, 2H), 9.68 (t, *J* = 3.2 Hz, 1H). ^{13}C NMR (100 MHz, $CDCl_3$) (*major regioisomer*) δ 14.29, 23.46, 24.41, 26.28, 28.85, 29.62, 31.72, 32.84, 35.17, 38.28, 42.03, 53.92, 55.45, 113.86, 128.96, 129.43, 135.07, 137.26, 157.81, 204.29. HRMS (ESI): Mass calcd for $C_{22}H_{35}O_2$ $[M+H]^+$: 331.2632; found: 331.2636.

(*R,*E*)-3-butyl-11-(3-methoxyphenyl)-3-methylundec-4-enal**: ***2ab***

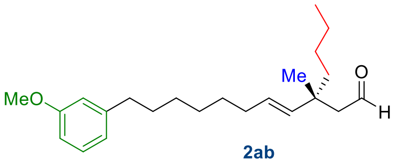

2ab

The compound **2ab** was prepared from the alcohol **1l** and 3-bromoanisole. The compound **2ab** was isolated as a major isomer along with its regioisomer **3ab** in a ratio of 19:1. Combined Yield: 40%. *E/Z* > 99:01. R_f = 0.4 (hexane/EtOAc, 90:10, v/v). ^{1}H NMR (400 MHz, $CDCl_3$) (*major regioisomer*) δ 0.86 (t, *J* = 7.1 Hz, 3H), 1.08 (s, 3H), 1.13-1.39 (m, 12H), 1.56-1.65 (m, 2H), 1.91-2.06 (m, 2H), 2.18-2.38 (m, 2H), 2.56 (t, *J* = 7.5 Hz, 2H), 3.78 (s, 3H), 5.24-5.45 (m, 2H), 6.62-6.82 (m, 3H), 7.12-7.22 (m, 1H), 9.69 (t, *J* = 3.2 Hz, 1H). ^{13}C NMR (100 MHz, $CDCl_3$) (*major regioisomer*) δ 14.28, 23.46, 24.43, 26.28, 29.17, 29.33, 29.70, 31.53, 32.91, 36.22, 38.29, 42.04, 53.93, 55.32, 111.01, 114.41, 121.06, 129.03, 129.35, 137.22, 144.71, 159.77, 204.28. HRMS (ESI): Mass calcd for $C_{23}H_{37}O_2$ $[M+H]^+$: 345.2788; found: 345.2803.

(*R,*E*)-3-butyl-11-(4-methoxyphenyl)-3-methylundec-4-enal**: ***2ac***

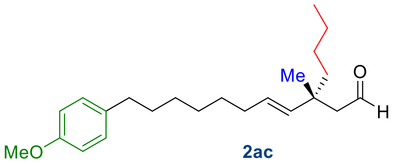

2ac

The compound **2ac** was prepared from the alcohol **1l** and 4-bromoanisole. The compound **2ac** was isolated as a major isomer along with its regioisomer **3ac** in a ratio of 7.3:1. Combined Yield: 66%. *E/Z* > 99:01. R_f = 0.4 (hexane/EtOAc, 90:10, v/v). ^{1}H NMR (400 MHz, $CDCl_3$) (*major regioisomer*) δ 0.86 (t, *J* = 7.1 Hz, 3H), 1.08 (s, 3H), 1.12-1.38 (m, 12H), 1.48-1.59 (m, 2H), 1.90-2.04 (m, 2H), 2.16-2.36 (m, 2H), 2.52 (t, *J* = 7.5 Hz, 2H), 3.77 (s, 3H), 5.23-5.45 (m, 2H), 6.80 (d, *J* = 8.4 Hz, 2H), 6.80 (d, *J* = 8.4 Hz, 2H), 7.07 (d, *J* = 8.4 Hz, 2H), 9.69 (t, *J* = 3.2 Hz, 1H). ^{13}C NMR (100 MHz, $CDCl_3$) (*major regioisomer*) δ 14.28, 23.46, 24.43, 26.28, 29.18, 29.26, 29.71, 31.90, 32.92, 35.22, 38.29, 42.05, 53.93, 55.45, 113.86, 129.05, 129.43, 135.15, 137.21, 157.81, 204.28. HRMS (ESI): Mass calcd for $C_{23}H_{37}O_2$ $[M+H]^+$: 345.2788; found: 345.2824.

(*R,*E*)-5-butyl-5-methyl-9-phenylnon-6-en-2-one**: ***2ad***

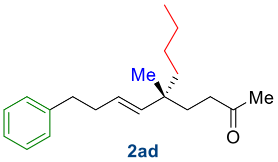

The compound **2ad** was prepared from the alcohol **1m** and iodobenzene. The compound **2ad** was isolated as a major isomer along with its regioisomer **3ad** in a ratio of 7.3:1. Combined Yield: 42%. *E/Z* > 99:01. R_f = 0.4 (hexane/EtOAc, 90:10, v/v). ^{1}H NMR (400 MHz, $CDCl_3$) (*major regioisomer*) δ 0.77-0.94 (m, 6H), 0.99-1.30 (m, 6H), 1.38-1.57 (m, 2H), 2.07 (s, 3H), 2.12-2.42 (m, 4H), 2.66 (t, *J* = 7.5 Hz, 2H), 5.10-5.36 (m, 2H), 7.04-7.35 (m, 5H). ^{13}C NMR (100 MHz, $CDCl_3$) (*major regioisomer*) δ 14.32, 23.00, 23.66, 26.39, 30.19, 34.72, 34.83, 36.35, 38.25, 39.25, 41.52, 125.90, 127.18, 128.41, 128.74, 139.18, 142.12, 209.93. HRMS (ESI): Mass calcd for $C_{20}H_{31}O$ $[M+H]^+$: 287.2351; found: 287.2375.

(*R,*E*)-5-butyl-9-(4-methoxyphenyl)-5-methylnon-6-en-2-one**: ***2ae***

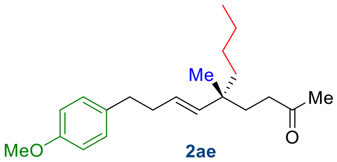

The compound **2ae** was prepared from the alcohol **1m** and 4-bromoanisole. The compound **2ae** was isolated as a major isomer along with its regioisomer **3ae** in a ratio of 8.1:1. Combined Yield: 52%. *E/Z* > 99:01. R_f = 0.3 (hexane/EtOAc, 90:10, v/v). ^{1}H NMR (400 MHz, $CDCl_3$) (*major regioisomer*) δ 0.76-0.94 (m, 6H), 1.01-1.32 (m, 6H), 1.37-1.57 (m, 2H), 2.07 (s, 3H), 2.12-2.35 (m, 4H), 2.60 (t, *J* = 7.3 Hz, 2H), 3.75 (s, 3H), 5.09-5.36 (m, 2H), 6.79 (d, *J* = 8.4 Hz, 2H), 7.05 (d, *J* = 8.4 Hz, 2H). ^{13}C NMR (100 MHz, $CDCl_3$) (*major regioisomer*) δ 14.32, 23.00, 23.67, 26.40, 30.14, 34.87, 34.96, 41.53,

55.39, 113.81, 127.29, 129.60, 134.20, 139.11, 157.85, 209.98. HRMS (ESI): Mass calcd for $C_{21}H_{33}O_2$ $[M+H]^+$: 317.2481; found: 317.2475.

(*R,*E*)-6-butyl-6-methyl-10-phenyldec-7-en-2-one**: ***2af***

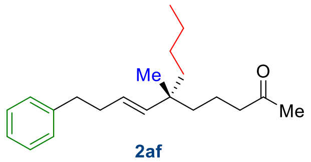

2af

The compound **2af** was prepared from the alcohol **1n** and iodobenzene. The compound **2af** was isolated as a major isomer along with its regioisomer **3af** in a ratio of 7.7:1. Combined Yield: 43%. *E/Z* > 99:01. R_f = 0.5 (hexane/EtOAc, 90:10, v/v). ^{1}H NMR (400 MHz, $CDCl_3$) (*major regioisomer*) δ 0.78-0.93 (m, 6H), 0.99-1.28 (m, 8H), 1.34-1.48 (m, 2H), 2.09 (s, 3H), 2.21-2.44 (m, 4H), 2.65 (t, *J* = 7.5 Hz, 2H), 5.15-5.34 (m, 2H), 7.08-7.30 (m, 5H). ^{13}C NMR (100 MHz, $CDCl_3$) (*major regioisomer*) δ 14.34, 18.87, 23.44, 23.70, 26.41, 30.02, 34.86, 36.50, 38.63, 41.02, 41.24, 44.74, 125.82, 16.54, 128.36, 128.71, 139.78, 142.28, 209.48. HRMS (ESI): Mass calcd for $C_{21}H_{33}O$ $[M+H]^+$: 301.2531; found: 301.2548.

(*R,*E*)-5-butyl-5-methyl-12-phenyldodec-6-en-2-one**: ***2ag***

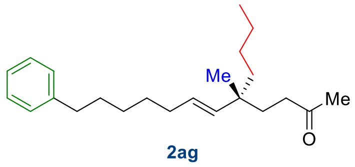

2ag

The compound **2ag** was prepared from the alcohol **1o** and iodobenzene. The compound **2ag** was isolated as a major isomer along with its regioisomer **3ag** in a ratio of 7.3:1. Combined Yield: 53%. *E/Z* > 99:01. R_f = 0.6 (hexane/EtOAc, 90:10, v/v). ^{1}H NMR (600 MHz, $CDCl_3$) (*major regioisomer*) δ 0.74-0.97 (m, 6H), 1.04-1.67 (m, 14H), 1.86-2.01 (m, 2H), 2.08 (s, 3H), 2.28 (t, *J* = 8.8 Hz, 2H), 2.57 (t, *J* = 8.5 Hz, 2H), 5.02-5.38 (m, 2H), 6.94-7.44 (m, 5H). ^{13}C NMR (100 MHz, $CDCl_3$) (*major regioisomer*) δ 14.35, 23.10, 23.69, 26.49, 29.00, 29.84, 30.19, 31.54, 32.95, 34.87, 36.16, 38.26, 39.39, 41.54, 125.79, 128.17, 128.43, 128.58, 138.39, 143.01, 209.90. HRMS (ESI): Mass calcd for $C_{23}H_{37}O$ $[M+H]^+$: 329.2844; found: 329.2863.

(3*S,*E*)-3-butyl-3-methyl-2,7-diphenylhept-4-enal**: ***2ah***

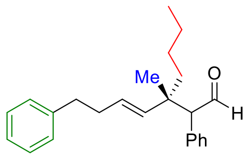

2ah

The compound **2ah** was prepared from the alcohol **1p** and iodobenzene. The compound **2ah** was isolated as a major isomer (*mixture of diastereomers*) along with its regioisomer **3ah** in a ratio of 6.7:1. Combined Yield: 62%. *E/Z* > 99:01. *dr* ~ 52:48. R_f = 0.5 (hexane/EtOAc, 90:10, v/v). ^{1}H NMR (400 MHz, $CDCl_3$) (*major regioisomer as a mixture of diastereomers d1 and d2 in 1:1 ratio*) δ 0.62-0.82 (m, 6H, *d1& d2*), 0.87 (s, 3H, *d1*), 0.99-1.40 (m, 15H, *d1& d2*), 2.20-2.40 (m, 4H, *d1*), 2.49-2.78 (m, 4H, *d2*), 3.15 (d, *J* = 4.4 Hz, 1H, *d1*), 3.27 (d, *J* = 3.6 Hz, 1H, *d2*), 5.11-5.57 (m, 4H, *d1& d2*), 6.80-7.45 (m, 20H, *d1& d2*), 9.67 (d, *J* = 4.4 Hz, 1H, *d1*), 9.76 (d, *J* = 3.6 Hz, 1H, *d2*).^{13}C NMR (100 MHz, $CDCl_3$, (*major regioisome as a mixture of two diastereomers, ^{13}C NMR value written as "d1 & d2"*) δ 14.29 & 14.30, 21.25 & 21.96, 23.44 & 23.47, 25.93 & 26.13, 34.80 & 30.89, 36.02 & 36.08, 39.41 & 39.93, 42.00 & 42.88, 67.53 & 67.59, 126.06 & 126.10, 127.36 & 127.41, 128.26 & 128.36, 128.52 (*d1,d2*), 128.68 & 128.74, 129.31 & 129.81, 130.51 & 130.78, 135.03 & 135.11, 135.87 & 136.26, 141.95 (*d1,d2*), 202.80 & 203.22. HRMS (ESI): Mass calcd for $C_{24}H_{31}O$ $[M+H]^+$: 335.2369; found: 335.2377.

(3*S,*E*)-3-butyl-7-(4-methoxyphenyl)-3-methyl-2-phenylhept-4-enal**: ***2ai***

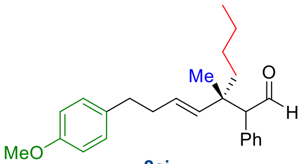

2ai

The compound **2ai** was prepared from the alcohol **1p** and iodobenzene. The compound **2ai** was isolated as a major isomer (*mixture of diastereomers*) along with its regioisomer **3ai** in a ratio of 8:1. Combined Yield: 55%. *E/Z* > 99:01. *dr* ~ 55:45. R_f = 0.5 (hexane/EtOAc, 90:10, v/v). ^{1}H NMR (400 MHz, $CDCl_3$) (*major regioisomer as a mixture of diastereomers d1 and d2 in 55:45 ratio*) δ 0.64-0.81 (m, 6H, *d1& d2*), 0.86 (s, 3H, *d1*), 0.92-1.43 (m, 15H, *d1& d2*), 2.16-2.42 (m, 4H, *d1*), 2.45-2.68 (m, 4H, *d2*), 3.14 (d, *J* = 4.4 Hz, 1H, *d1*), 3.26 (d, *J* = 3.7 Hz, 1H, *d2*), 3.69 & 3.70 (2 x s, 6H, *d1& d2*), 5.13-5.55 (m, 4H, *d1& d2*), 6.76 (d, *J* = 7.6 Hz, 4H, *d1& d2*), 6.91-7.11 (m, 8H, *d1& d2*), 7.14-7.28 (m, 6H, *d1& d2*), 9.69 (d, *J* = 4.4 Hz, 1H, *d1*), 9.75 (d, *J* = 3.7 Hz, 1H, *d2*).^{13}C NMR (100 MHz, $CDCl_3$, (*major regioisome as a mixture of two diastereomers, ^{13}C NMR value written as "d1 & d2"*) δ 14.28, 21.24 & 21.99, 23.43 & 23.46, 25.93 & 26.14, 35.05, 35.12 & 35.17, 39.42 & 39.94, 42.00 & 42.88, 55.45, 67.53 & 67.58, 113.95, 127.37 & 127.41, 128.25 & 128.35, 129.43 & 129.60, 129.55 & 129.60, 130.51 & 130.78, 134.03, 135.06 & 135.13, 135.79 & 136.19, 158.00 & 158.04, 202.83 & 203.24. HRMS (ESI): Mass calcd for $C_{25}H_{33}O_2$ $[M+H]^+$: 365.2475; found: 365.2477.

(4*S,5*R**,*E*)-5-butyl-9-(3-methoxyphenyl)-4,5-dimethylnon-6-en-2-one**: ***2aj***

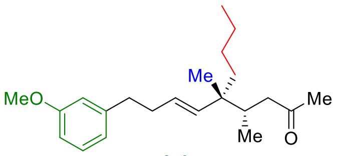

2aj

The compound **2aj** was prepared from the alcohol **1q** and 3-bromoanisole. The compound **2aj** was isolated as a major isomer along with its regioisomer **3aj** in a ratio of 11.5:1. Combined Yield: 65%. *E/Z* > 99:01. dr > 98:02. R_f = 0.3 (hexane/EtOAc, 90:10, v/v). ^{1}H NMR (400 MHz, $CDCl_3$) (*major regioisomer*) δ 0.68-0.91 (m, 9H), 1.00-1.11 (m, 2H), 1.12-1.28 (m, 4H), 1.80-2.11 (m, 5H), 2.24-2.42 (m, 3H), 2.52-2.74 (m, 2H), 3.77 (s, 3H), 5.11-5.42 (m, 2H), 6.60-6.80 (m, 3H), 7.16 (dd, *J* = 9.0, 7.1 Hz, 1H). ^{13}C NMR (100 MHz, $CDCl_3$) (*major regioisomer*) δ 14.31, 14.54, 18.41, 23.73, 26.41, 30.64, 34.63, 36.39, 36.56, 39.67, 41.37, 47.14, 55.29, 111.17, 114.51, 121.16, 127.71, 129.37, 138.95, 143.74, 159.77, 209.84. HRMS (ESI): Mass calcd for $C_{22}H_{35}O_2$ $[M+H]^+$: 331.2632; found: 331.2633.

(4*S,5*R**,*E*)-5-butyl-9-(4-methoxyphenyl)-4,5-dimethylnon-6-en-2-one**: ***2ak***

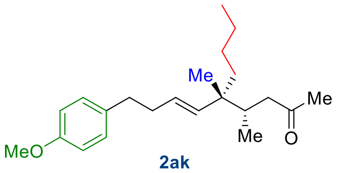

2ak

The compound **2ak** was prepared from the alcohol **1q** and 4-bromoanisole. The compound **2ak** was isolated as a major isomer along with its regioisomer **3ak** in a ratio of 4.6:1. Combined Yield: 44%. *E/Z* > 99:01. dr > 98:02. R_f = 0.3 (hexane/EtOAc, 90:10, v/v). ^{1}H NMR (600 MHz, $CDCl_3$) (*major regioisomer*) δ 0.71-0.89 (m, 9H), 1.01-1.31 (m, 7H), 1.82-1.98 (m, 2H), 2.03 (s, 3H), 2.25-2.34 (m, 2H), 2.52-2.66 (m, 2H), 3.75 (s, 3H), 5.14-5.43 (m, 2H), 6.78 (d, *J* = 8.5 Hz, 2H), 7.05 (d, *J* = 8.4 Hz, 2H). ^{13}C NMR (100 MHz, $CDCl_3$) (*major regioisomer*) δ 14.32, 14.53, 18.36, 23.73, 26.43, 30.62, 34.96, 35.39, 36.55, 39.68, 41.35, 47.14, 55.37, 113.83, 127.82, 129.57, 134.15, 138.92, 157.86, 209.86. HRMS (ESI): Mass calcd for $C_{22}H_{34}O_2Na$ $[M+Na]^+$: 353.2459; found: 353.2456.

(4*S,5*R**,*E*)-5-cyclohexyl-4,5-dimethyl-9-phenylnon-6-en-2-one**: ***2al***

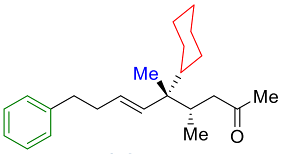

2al

The compound **2al** was prepared from the alcohol **1r** and bromobenzene. The compound **2al** was isolated as a major isomer along with its regioisomer **3al** in a ratio of 8.1:1. Combined Yield: 57%. *E/Z* > 99:01. dr > 98:02. R_f = 0.5 (hexane/EtOAc, 90:10, v/v). ^{1}H NMR (400 MHz, $CDCl_3$) (*major

regioisomer) δ 0.64-0.91 (m, 7H), 0.93-1.31 (m, 6H), 1.49-1.76 (m, 4H), 1.90-2.45 (m, 8H), 2.67 (t, *J* = 7.4 Hz, 2H), 5.03-5.53 (m, 2H), 7.00-7.39 (m, 5H). ^{13}C NMR (100 MHz, $CDCl_3$) (*major regioisomer*) δ 14.36, 15.97, 26.93, 27.26, 27.37, 27.41, 27.74, 30.80, 33.30, 34.81, 36.34, 43.97, 44.07, 46.98, 125.91, 128.43, 128.73, 128.93, 136.50, 142.07, 209.91. HRMS (ESI): Mass calcd for $C_{23}H_{35}O$ $[M+H]^+$: 327.2682; found: 327.2649.

(4*S,5*R**,*E*)-5-cyclohexyl-9-(3-methoxyphenyl)-4,5-dimethylnon-6-en-2-one**: ***2am***

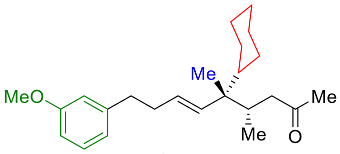

2am

The compound **2am** was prepared from the alcohol **1r** and 3-bromoanisole. The compound **2am** was isolated as a major isomer along with its regioisomer **3am** in a ratio of 4.9:1. Combined Yield: 56%. *E/Z* > 99:01. *dr* > 98:02. R_f = 0.4 (hexane/EtOAc, 90:10, v/v). ^{1}H NMR (400 MHz, $CDCl_3$) (*major regioisomer*) δ 0.63-0.91 (m, 7H), 0.95-1.28 (m, 6H), 1.49-1.78 (m, 4H), 1.91-2.18 (m, 5H), 2.22-2.43 (m, 3H), 2.64 (t, *J* = 7.2 Hz, 2H), 3.76 (s, 3H), 5.07-5.49 (m, 2H), 6.59-6.84 (m, 3H) 7.15 (t, *J* = 7.7 Hz, 1H). ^{13}C NMR (100 MHz, $CDCl_3$) (*major regioisomer*) δ 14.34, 15.96, 26.92, 27.25, 27.38, 27.40, 27.74, 30.75, 33.31, 34.69, 36.37, 43.97, 44.07, 46.97, 55.27, 111.15, 114.51, 121.16, 128.87, 129.36, 136.51, 143.71, 159.77, 209.93. HRMS (ESI): Mass calcd for $C_{24}H_{37}O_2$ $[M+H]^+$: 357.2788; found: 357.2785.

(4*S,5*R**,*E*)-9-(4-chlorophenyl)-5-cyclohexyl-4,5-dimethylnon-6-en-2-one**: ***2an***

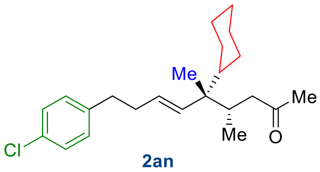

2an

The compound **2an** was prepared from the alcohol **1r** and 1-iodo-4-chlorobenzene. The compound **2an** was isolated as a major isomer along with its regioisomer **3an** in a ratio of 7.3:1. Combined Yield: 47%. *E/Z* > 99:01. *dr* > 98:02. R_f = 0.5 (hexane/EtOAc, 90:10, v/v). ^{1}H NMR (400 MHz, $CDCl_3$) (*major regioisomer*) δ 0.58-0.88 (m, 7H), 0.90-1.26 (m, 6H), 1.46-1.77 (m, 4H), 1.89-2.40 (m, 8H), 2.64 (t, *J* = 7.3 Hz, 2H), 5.05-5.37 (m, 2H), 7.06 (d, *J* = 8.0 Hz, 1H), 7.20 (d, *J* = 8.0 Hz, 1H). ^{13}C NMR (100 MHz, $CDCl_3$) (*major regioisomer*) δ 14.38, 15.96, 26.90, 27.27, 27.34, 27.37, 27.74, 30.83, 33.26, 34.64, 35.58, 44.00, 44.04, 46.94, 128.45, 128.52, 130.11, 131.61, 136.95, 140.47, 209.82. HRMS (ESI): Mass calcd for $C_{23}H_{34}ClO$ $[M+H]^+$: 361.2293; found: 361.2285.

(4*S,5*R**,*E*)-5-butyl-4,5-dimethyl-12-phenyldodec-6-en-2-one**: ***2ao***

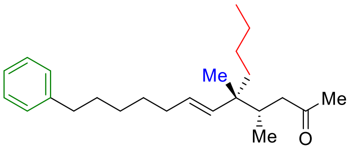

2ao

The compound **2ao** was prepared from the alcohol **1s** and bromobenzene. The compound **2ao** was isolated as a major isomer along with its regioisomer **3ao** in a ratio of 8.1:1. Combined Yield: 51%. *E/Z* > 99:01. *dr* > 98:02. R_f = 0.5 (hexane/EtOAc, 90:10, v/v). ^{1}H NMR (600 MHz, $CDCl_3$) (*major regioisomer*) δ 0.76-0.83 (m, 6H), 0.86 (t, *J* = 7.3 Hz, 3H), 1.08-1.40 (m, 9H), 1.55-1.64 (m, 2H), 1.87-2.05 (m, 5H), 2.07 (s, 3H), 2.48 (dd, *J* = 16.1, 2.5 Hz, 1H), 2.58 (t, *J* = 7.8 Hz, 2H), 5.13-5.34 (m, 2H), 7.12-7.18 (m, 3H), 7.22-7.28 (m, 2H). ^{13}C NMR (100 MHz, $CDCl_3$) (*major regioisomer*) δ 14.36, 14.68, 18.49, 23.77, 26.54, 29.03, 29.88, 30.69, 31.55, 33.02, 36.16, 36.61, 39.68, 41.37, 47.27, 125.79, 128.44, 128.57, 128.76, 138.11, 143.00, 209.78. HRMS (ESI): Mass calcd for $C_{24}H_{39}O$ $[M+H]^+$: 343.2995; found: 343.3012.

(4*S,5*R**,*E*)-5-butyl-12-(3-methoxyphenyl)-4,5-dimethyldodec-6-en-2-one**: ***2ap***

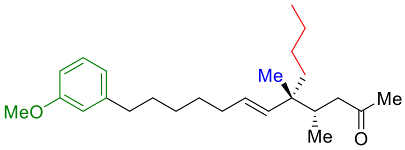

2ap

The compound **2ap** was prepared from the alcohol **1s** and 3-bromoanisole. The compound **2ap** was isolated as a major isomer along with its regioisomer **3ap** in a ratio of 9:1. Combined Yield: 45%. *E/Z > 99:01*. *dr* > 98:02. R_f = 0.4 (hexane/EtOAc, 90:10, v/v). ^{1}H NMR (600 MHz, $CDCl_3$) (*major regioisomer*) δ 0.76-0.91 (m, 9H), 1.01-1.40 (m, 10H), 1.55-1.61 (m, 2H), 1.88-2.12 (m, 7H), 2.48 (dd, *J* = 16.6, 2.6 Hz, 1H), 2.55 (t, *J* = 8.5 Hz, 2H), 3.77 (s, 3H), 5.11-5.36 (m, 2H), 6.67-6.77 (m, 3H), 7.17 (dd, J = 9.0, 7.3 Hz, 1H). ^{13}C NMR (100 MHz, $CDCl_3$) (*major regioisomer*) δ 14.35, 14.67, 18.46, 23.75, 26.52, 29.03, 29.87, 30.68, 31.41, 33.00, 36.19, 36.59, 39.59, 39.66, 41.35, 47.26, 55.31, 110.99, 114.40, 121.03, 128.75, 129.36, 138.10, 144.66, 159.77, 209.81. HRMS (ESI): Mass calcd for $C_{25}H_{41}O_2$ $[M+H]^+$: 373.3101; found: 373.3102.

(*R,*E*)-3-butyl-3-methyl-7-phenylhept-4-enal-7,7-*d*$_2$**: ***11***

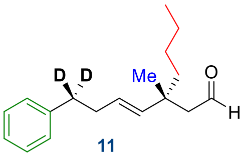

11

The compound **11** was prepared from the alcohol **5** and iodobenzene. The compound **11** was isolated as a major isomer along with its regioisomer **11*** in a ratio of 6.7:1. Combined Yield: 67%. *E/Z* > 99:01. R_f = 0.5 (hexane/EtOAc, 90:10, v/v). ^{1}H NMR (400 MHz, $CDCl_3$) (*major regioisomer*) δ 0.86 (t, *J* = 7.2 Hz, 3H), 0.99-1.42 (m, 9H), 2.09-2.47 (m, 4H), 5.25-5.59 (m, 2H), 7.08-7.20 (m, 3H), 7.22-7.36 (m, 2H), 9.57 (t, *J* = 3.1 Hz, 1H). ^{13}C NMR (100 MHz, $CDCl_3$) (*major regioisomer*) δ 14.24, 23.42, 24.36, 26.18, 34.51, 34.96-35.97 (m, *C*D_2), 38.27, 41.95, 53.88, 125.99, 127.91, 128.44, 128.68, 130.09, 141.81, 204.16. HRMS (ESI): Mass calcd for $C_{18}H_{24}D_2ONa$ $[M+Na]^+$: 283.2007; found: 283.2004.

(3*R,*E*)-3-butyl-3-methyl-10-phenyldec-4-enal-7,8-*d*2: *12***

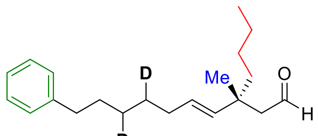

12

The compound **12** was prepared from the alcohol **6** and iodobenzene. The compound **12** was isolated as a major isomer along with its regioisomer **12*** in a ratio of 4.6:1. Combined Yield: 78%. *E/Z* > 99:01. R_f = 0.6 (hexane/EtOAc, 90:10, v/v). ^{1}H NMR (400 MHz, $CDCl_3$) (*major regioisomer*) δ 0.87 (t, *J* = 7.1 Hz, 3H), 1.08 (s, 3H), 1.12-1.38 (m, 8H), 1.57-1.66 (m, 2H), 2.00 (t, *J* = 7.2 Hz, 2H), 2.18-2.37 (m, 2H), 2.58 (t, *J* = 7.7 Hz, 2H), 5.24-5.49 (m, 2H), 7.05-7.30 (m, 5H), 9.68 (t, *J* = 3.1 Hz, 1H). ^{13}C NMR (100 MHz, $CDCl_3$) (*major regioisomer*) δ 14.29, 23.47, 24.42, 26.28, 28.40 (t, *J* = 19.2 Hz), 29.11 (t, *J* = 19.2 Hz), 31.37, 32.71, 36.10, 38.29, 42.02, 53.92, 125.80, 128.44, 128.59, 128.94, 137.29, 142.98, 204.27. HRMS (ESI): Mass calcd for $C_{21}H_{30}D_2ONa$ $[M+Na]^+$: 325.2476; found: 325.2469.

(*R,*E*)-3-cyclohexyl-3-methyl-7-phenylhept-4-enal-4-*d*: *13***

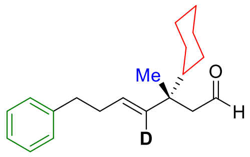

13

The compound **13** was prepared from the alcohol **7** and iodobenzene. The compound **13** was isolated as a major isomer along with its regioisomer **13*** in a ratio of 5.7:1. Combined Yield: 70%. *E/Z* > 99:01. R_f = 0.6 (hexane/EtOAc, 90:10, v/v). ^{1}H NMR (400 MHz, $CDCl_3$) (*major regioisomer*) δ 0.74-0.92 (m, 2H), 0.95-1.27 (m, 7H), 1.51-1.82 (m, 5H), 2.15-2.46 (m, 4H), 2.67 (t, *J* = 7.4 Hz, 2H), 5.33 (t, *J* = 7.2 Hz, 1H), 7.01-7.40 (m, 5H), 9.54 (t, *J* = 3.2 Hz, 1H). ^{13}C NMR (100 MHz, $CDCl_3$) (*major regioisomer*) δ 20.83, 26.69, 27.10, 27.24, 27.47, 34.66, 36.15, 40.93, 47.68, 51.96, 125.98, 128.45,

128.60, 128.69, 136.90 (t, J = 22.8 Hz), 141.85, 204.87. HRMS (ESI): Mass calcd for $C_{20}H_{28}DO$ $[M+H]^+$: 286.2281; found: 286.2261.

Compound ***14***: Data of the compound **14** was found identical with the compound **2a**.

(*R,*E*)-3-butyl-3-methyl-7-phenylhept-4-enal-1-*d*: *15***

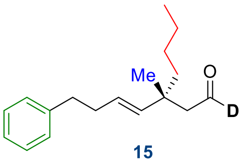

15

The compound **15** was prepared from the alcohol **9** and iodobenzene. The compound **15** was isolated as a major isomer along with its regioisomer **15*** in a ratio of 7.3:1. Combined Yield: 77%. *E/Z* > 99:01. R_f = 0.5 (hexane/EtOAc, 90:10, v/v). ^{1}H NMR (400 MHz, $CDCl_3$) (*major regioisomer*) δ 0.81 (t, J = 7.1 Hz, 3H), 0.94-1.37 (m, 9H), 2.03-2.42 (m, 4H), 2.61 (t, J = 7.3 Hz, 2H), 5.24-5.57 (m, 2H), 7.01-7.29 (m, 5H). ^{13}C NMR (100 MHz, $CDCl_3$) (*major regioisomer*) δ 14.24, 23.42, 24.34, 26.18, 34.67, 36.18, 38.26, 41.96, 53.73 (t, J = 2.9 Hz), 125.99, 127.92, 128.45, 128.69, 138.08, 141.87, 203.89 (t, J = 25.9 Hz). HRMS (ESI): Mass calcd for $C_{18}H_{26}DO$ $[M+H]^+$: 260.2125; found: 260.2106.

(4*S,5*R**,*E*)-5-butyl-9-(4-methoxyphenyl)-5-methylnon-6-en-2-one-4-*d*: *16***

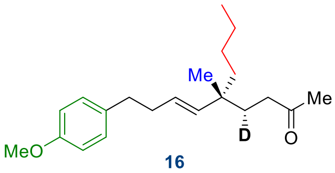

16

The compound **16** was prepared from the alcohol **10** and 4-bromoanisole. The compound **16** was isolated as a major isomer along with its regioisomer **16*** in a ratio of 7.7:1. Combined Yield: 48%. *E/Z > 99:01*. dr > 98:02. R_f = 0.3 (hexane/EtOAc, 90:10, v/v). ^{1}H NMR (400 MHz, $CDCl_3$) (*major regioisomer*) δ 0.78-0.94 (m, 6H), 1.01-1.32 (m, 6H), 1.43-1.51 (m, 1H), 2.07 (s, 3H), 2.12-2.35 (m, 4H), 2.60 (t, J = 7.3 Hz, 2H), 3.75 (s, 3H), 5.08-5.43 (m, 2H), 6.79 (d, J = 8.4 Hz, 2H), 7.05 (d, J = 8.4 Hz, 2H). ^{13}C NMR (100 MHz, $CDCl_3$) (*major regioisomer*) δ 14.31, 23.01, 23.66, 26.41, 30.12, 34.50 (t, J = 19.2 Hz, *C*D), 34.95, 35.44, 38.18, 39.18, 41.49, 55.39, 113.82, 127.28, 129.59, 134.21, 139.12, 157.87, 209.93. HRMS (ESI): Mass calcd for $C_{21}H_{32}DO_2$ $[M+H]^+$: 318.2543; found: 318.2540.

(xxiii) *Synthesis of compound* **4**: The compound **4** was synthesized from the compound **2aj** according to following scheme:

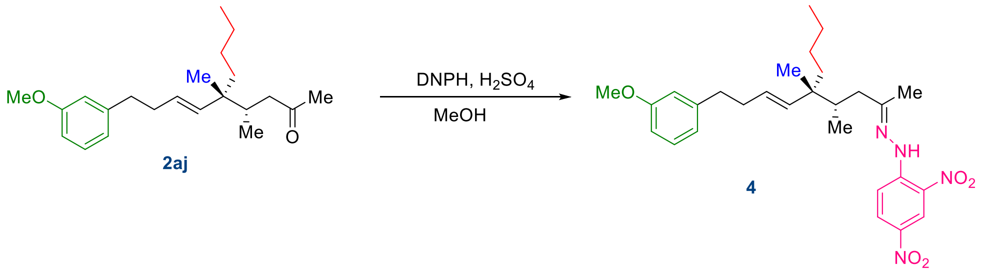

Procedure: To a solution of ketone **2aj** (15 mg, 0.045 mmol) and 2,4-dinitrophenylhydrazine (DNPH) (9 mg, 0.045 mmol) in EtOH (2 mL), conc. H_2SO_4 (1 drop) was added and the reaction mixture was stirred at 70 ^oC for overnight. After completion of the reaction (as monitored by TLC), water (5 mL) was added and the reaction mixture was extracted by ethyl acetate (3 x 5 mL). The combined organic layer was dried over $MgSO_4$ and evaporated to give crude product which was further purified by column chromatography using 5-10% diethyl ether in hexane as eluent.

1-((4*S*,5*R*,*E*)-5-butyl-9-(3-methoxyphenyl)-4,5-dimethylnon-6-en-2-ylidene)-2-(2,4-dinitrophenyl)hydrazine: ***4***

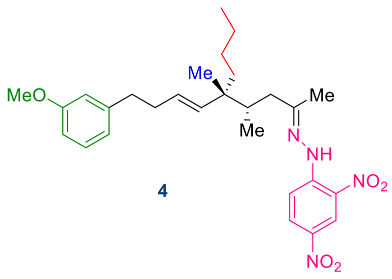

The compound **4** was obtained as yellow-orange solid in 90% yield. *E/Z* > 99:01. *dr* > 98:02. R_f = 0.4 (hexane/EtOAc, 90:10, v/v). ^{1}H NMR (400 MHz, $CDCl_3$) δ 0.79 (d, *J* = 6.6 Hz, 3H), 0.82-0.92 (m, 6H), 1.07-1.34 (m, 6H), 1.60-1.72 (m, 1H), 1.84-1.99 (m, 4H), 2.29-2.46 (m, 3H), 2.57-2.75 (m, 2H), 3.75 (s, 3H), 5.20-5.40 (m, 2H), 6.57-6.80 (m, 3H), 7.15 (t, *J* = 7.9 Hz, 1H), 7.94 (d, *J* = 9.6 Hz, 1H), 8.28 (dd, *J* = 9.5, 2.6 Hz, 1H), 9.11 (d, *J* = 2.5 Hz, 1H), 11.00 (s, 1H). ^{13}C NMR (100 MHz, $CDCl_3$) δ 13.84, 14.36, 16.15, 18.37, 23.76, 26.48, 34.74, 36.42, 38.47, 39.82, 41.78, 41.99, 55.31, 111.11, 114.64, 116.70, 121.19, 123.81, 128.09, 129.16, 129.41, 130.22, 137.81, 138.82, 143.72, 145.42, 158.95, 159.82. HRMS (ESI): Mass calcd for $C_{28}H_{39}N_4O_5$ $[M+H]^+$: 511.2915; found: 511.2905.

Supplementary Note 1

Crystal structure determination of the compound product **4**. Single crystals suitable for X-ray diffraction analysis were grown by dissolving the compound **4** (5 mg) in acetonitrile (1 mL) and allowing it to evaporate slowly at room temperature. The single-crystal material was immersed in Paratone–N oil and mounted on a Kappa CCD diffractometer at temperature 200(2) K. Data collection was performed using monochromated Mo Kα radiation, λ = 0.71073 Å, using φ and ω scans to cover the Ewald sphere.[3] Accurate cell parameters were obtained with the amount of indicated reflections.[4] The structure was solved by direct methods (SHELXS-97)[5] and refined by full-matrix least-squares methods against F^2 (SHELXL-97).[6] All non-hydrogen atoms were refined with anisotropic displacement parameters. The hydrogen atoms were refined isotropically on calculated positions using a riding model with their U_{iso} values constrained to 1.5 times the U_{eq} of their pivot atoms for terminal sp^3 carbon atoms and 1.2 times for all other carbon atoms. Software used for molecular graphics: Mercury 3.7.[7]

Supplementary Table 2. *Crystal data and structure refinement of compound* **4**

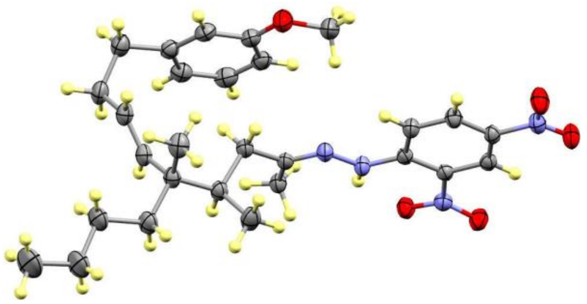

Crystal data	*Compound* **4**
**Empirical formula**	$C_{28}H_{38}N_4O_5$
**Formula weight**	510.62
**Temperature (K)**	200(2)
**Wavelength (Å)**	0.71073
**Crystal system,**	Monoclinic
**space group**	P 21/c
**a (Å)**	8.53(2)
**b (Å)**	32.62(3) A
**c (Å)**	10.37 (2)
**alpha**	90
**beta**	103.24(3)
**gamma**	90
**Volume (Å^3)**	2851(2)
**Z**	4
**Calculated density (mg/m^3)**	1.190
**Absorption coefficient (mm^{-1})**	0.082
**F(000)**	1096
**Crystal size (mm)**	0.15x0.21x0.36
**Theta range**	1.25 – 24.14
**Reflection collected/unique**	4193 / 4193
**Rint**	0.0710
**Completeness (%)**	99.1
**Absorption correction**	semi-empirical
**Data/restraints/ parameters**	4193/0/339
**Goodness-of-fit on F^2**	1.088
**R1, wR2 [I>2sigma(I)]**	0.0578, 0.1466
**R1, wR2 (all data)**	0.0854, 0.1636
**Largest diff. peak and hole**	0.303 , -0.289
**Diffractometer**	Nonius KappaCCD

Supplementary References

1. Liao, L. *et. al* An efficient and general method for resolving cyclopropene carboxylic acids. *Tetrahedron* **60**, 1803-1816 (2004).

2. Didier, D. *et. al.* Modulable and highly diastereoselective carbometalation of cyclopropenes. *Chem. Eur. J.* **20**, 1038-1048 (2014).

3. Kappa CCD Server Software, Nonius BV, Delft, The Netherlands (1997).

4. Otwinowski, Z. & Minor, W. Processing of X-ray diffraction data collected in oscillation mode. *Methods Enzymol.* **276**, 307-326 (1997).

5. Sheldrick, G. M. *Acta Crystallogr., Sect. A: Fundam. Crystallogr.* **46**, 467-473 (1990).

6. ORTEP, TEXSAN Structure Analysis Package, Molecular Structure Corp., The Woodlands, TX (1999).

7. Mercury Software from CCDC: http://www.ccdc.cam.ac.uk/ Solutions/CSDSystem/Pages/Mercury.aspx.
